# Supplementary material for: Breaking photoswitch activation depth limit using ionising radiation stimuli adapted to clinical application
Source: Nat Commun. 2022 Jul 14;13:4102. doi: 10.1038/s41467-022-30917-0 (PMC9283480; doi:10.1038/s41467-022-30917-0)
Supplement: Supplementary file 1 — Supplementary Information [file 41467_2022_30917_MOESM1_ESM.pdf]

Supplementary Information for

Breaking photoswitch activation depth limit using ionising radiation stimuli adapted to clinical application

Authors: Alban Guesdon-Vennerie, Patrick Couvreur, Fatoumia Ali, Frédéric Pouzoulet, Christophe Roulin, Immaculada Martínez-Rovira, Guillaume Bernadat, François-Xavier Legrand, Claudie Bourgaux, Cyril Lucien Mazars, Sergio Marco, Sylvain Trépout, Simona Mura, Sébastien Mériaux, Guillaume Bort.

This PDF file includes:

Materials and Methods

Supplementary Figs. 1 to 93

Supplementary Tables 1 to 11

Captions for Supplementary Videos 1 to 3

Other supplementary material for this manuscript includes the following:

Videos 1 to 3

Source data file

Table of Contents

Supplementary Section 1 - General analytical procedures p. 4

Supplementary Section 2 - Syntheses of Azo and GdAzo p. 6

Supplementary Section 3 - Isomerisation monitoring p. 22
- 3.1 - Absorbance and ^{1}H NMR monitoring p. 22
- 3.2 - Thermal back relaxation of *cis*-GdAzo p. 23

Supplementary Section 4 - Irradiation sources p. 24

Supplementary Section 5 - Characterisation of *cis*-GdAzo activation upon ionising radiations p. 25
- 5.1 - Quantification of *cis*-GdAzo activation upon ionising radiations p. 25
- 5.2 - Confirmation of isomerisation by LC-MS p. 52
- 5.3 - Confirmation of isomerisation reversibility p. 61
- 5.4 - Determination of *G*-values and comparison of the radiation sources p. 62
- 5.5 - Determination of activation constants p. 64

Supplementary Section 6 – Monte Carlo simulations p. 68

Supplementary Section 7 - Investigation of activation mechanism using various converter species
- 7.1 - Protocol p. 69
- 7.2 - Results p. 69

Supplementary Section 8 - Investigation of activation mechanism and role of hydroxyl radicals p. 77
- 8.1 - *cis*-GdAzo activation under N_2 and N_2O gas saturation p. 77
- 8.2 - *cis*-GdAzo activation using Fenton chemistry p. 79

Supplementary Section 9 - Investigation of activation mechanism and role of Gd p. 81
- 9.1 - Quantification of hydroxyl radicals and role of Gd p. 81
- 9.2 - Impact of Gd on *cis*-Azo activation upon ionising radiations p. 85

Supplementary Section 10 - Investigation of activation mechanism by variation of GdAzo concentration p. 87

Supplementary Section 11 - Physicochemical characterisation p. 93
- 11.1 - Micelle characterisation by fluorescence labelling p. 93
- 11.2 - Relaxivity measurement p. 94

Supplementary Section 12 - Computational details for electronic structure calculation p. 96

12.1 - Structures of GdAzo derivatives and local surface polarities p. 96

12.2 - Dipole moments p. 98

12.3 - Total electron densities p. 100

12.4 - Molecular models of GdAzo derivatives p. 100

Supplementary Section 13 - Characterisation of *trans*-GdAzo aggregation and interaction with cell membrane models p. 120

13.1 - Sample preparation and methods for SAXS and DSC experiments p. 120

13.2 - SAXS characterisation of *trans*- GdAzo micelles p. 121

13.3 - Study of *trans*-GdAzo/DPPC bilayer interactions using SAXS and DSC p. 122

Supplementary Section 14 - *In vitro* characterisation on cancer cells p. 128

14.1 - Toxicity of *cis*-GdAzo (cell viability by MTT assay) p. 129

14.2 - Cell permeabilisation of *trans*-GdAzo without irradiation (confocal microscopy) p. 130

14.3 - Cell permeabilisation of *cis*-GdAzo upon ionising radiation (confocal microscopy) p. 138

14.4 - Cell permeabilisation of *cis*-GdAzo upon ionising radiation (flow cytometry) p. 141

14.5 - Intracellular distribution by EELS-TEM p. 143

14.6 - Cytotoxicity of *cis*-GdAzo upon ionising radiation p. 144

14.7 - Gem cellular uptake in the presence of *trans*-GdAzo p. 147

Abbreviations p. 149

References p. 150

<u>Supplementary Section 1</u>

<u>General analytical procedures</u>

High-performance liquid chromatography (HPLC)

Method A

HPLC was carried out on Binary HPLC pump 1565 (Waters), Photodiode Array Detector (PAD) 2998 (Waters), with a reverse phase column Agilent Eclipse XDB-C18 (length: 250 mm, diameter: 4.6 mm, stationary phase: 5 μm) using a gradient system of elution water-0.05% TFA/acetonitrile-0.05% TFA; 0’ (98/2), 5’ (98/2), 25’ (0/100), 27’ (0/100), 29’ (98/2), 35’ (98/2) at a 1 $mL.min^{-1}$ flow rate (30 μL injected). Data were treated on Empower.

Method B

HPLC was carried out on Binary HPLC pump 1565 (Waters), PAD 2998 (Waters), with a reverse phase column XBridge C18 (length: 100 mm, diameter: 3.0 mm, stationary phase: 3.5 μm) using an isocratic system of elution water-0.05% TFA/acetonitrile 63:37 at a 0.75 $mL.min^{-1}$ flow rate (10 μL injected). The extracted wavelengths were 319 nm and 320 nm for **Azo** and **GdAzo** compounds respectively (isobestic points). Data were treated on Empower.

Method C

HPLC was carried out on Binary HPLC pump 2525 (Waters), 515 HPLC Pump (Waters), 2996 PDA (Waters), with a reverse phase column XBridge C18 (length: 100 mm, diameter: 3.0 mm, stationary phase: 3.5 μm) using an isocratic system of elution water-0.05% TFA/acetonitrile 63:37 at a 0.75 $mL.min^{-1}$ flow rate (5, 10 or 20 μL injected). The extracted wavelengths were 319 nm and 320 nm for **Azo** and **GdAzo** compounds respectively (isobestic points). Data were treated on MassLynx.

High-performance liquid chromatography-mass spectrometry (LC-MS)

Method D

LC-MS was carried out on Alliance 2695 (Waters), 2996 PDA (Waters), with a reverse phase column XBridge C18 (length: 150 mm, diameter: 2.1 mm, stationary phase: 3.5 µm) using a gradient system of elution water-0.1% formic acid/acetonitrile; 0’ (95/5), 20’ (0/100) at a 0.25 mL.min^{-1} flow rate (10 µL injected). The Mass analyser was a TOF LCT Premier (Waters). The capillary tension was 2.8 kV for ES+. The cone tension was 35 V. The temperature of the source was 120 °C and the temperature of desolvation was 280 °C. Data were treated on MassLynx.

Method E

LC-MS was carried out on 2695 separations module (Waters), 2998 PDA (Waters), with a reverse phase column XBridge C18 (length: 100 mm, diameter: 3.0 mm, stationary phase: 3.5 µm) at 20 °C using a gradient system of elution water-0.1% formic acid/acetonitrile-0.1% formic acid; 0’ (98/2), 1’ (98/2), 7’ (0/100), 9’ (0/100), 13’ (98/2) at a 0.75 mL.min^{-1} flow rate (10 µL injected). The Mass analyser was a MicromassZQ (Waters). The cone tension was 35 V. The temperature of the source was 120 °C and the temperature of desolvation was 400 °C. Data were treated on MassLynx.

<u>Supplementary Section 2</u>

<u>Syntheses of Azo and GdAzo</u>

General information

All reagents were purchased from Sigma Aldrich or Alfa Aesar commercial sources with the highest grade commercially available and used without further purification. 2,2′,2”-(10-(2,6-dioxotetrahydro-2H-pyran-3-yl)-1,4,7,10-tetraazacyclododecane-1,4,7-triyl)triacetic acid (DOTAGA anhydride) was purchased from CheMatech® (Dijon, France) and used without further purification. Silica gel (Aldrich 717185 Si 60, 40-63 μm) used for flash column chromatography was purchased from VWR. Flash column chromatographies with RP-18 were carried out using a CombiFlash device (Biotage). Thin layer chromatographies (TLC) were performed using aluminium-backed sheets coated with 60F254 silica gel (visualisation by UV lamp at 254 nm). ^{1}H and ^{13}C nuclear magnetic resonance (NMR) spectra were acquired on Bruker 300, 400 or 500 MHz spectrometers at room temperature (rt). Chemical shifts δ are given in ppm with reference to tetramethylsilane as the solvent signal. Coupling patterns are described by abbreviations: d (doublet), dt (doublet of triplets), m (multiplet), t (triplet). 2D NMR experiments were performed for assigning signals in the NMR spectra, such as homonuclear correlation spectroscopy (COSY), nuclear overhauser effect spectroscopy (NOESY), heteronuclear single quantum coherence spectroscopy (HSQC) and heteronuclear multiple bond correlation (HMBC). High-resolution mass spectrometry (HRMS) analyses were performed in water:methanol 3:7 mixture from ionisation by electrospray on positive mode, unless otherwise specified, using a TOF mass analyser on a Waters TOF-LCT Premier mass spectrometer. The high-performance liquid chromatography (HPLC) and liquid chromatography-mass spectroscopy (LC-MS) methods are described in the Supplementary Section 1. The purity of intermediates was measured by ^{1}H NMR or reverse-phase HPLC. The purity of the final compounds was determined by reverse-phase HPLC and was confirmed > 95%.

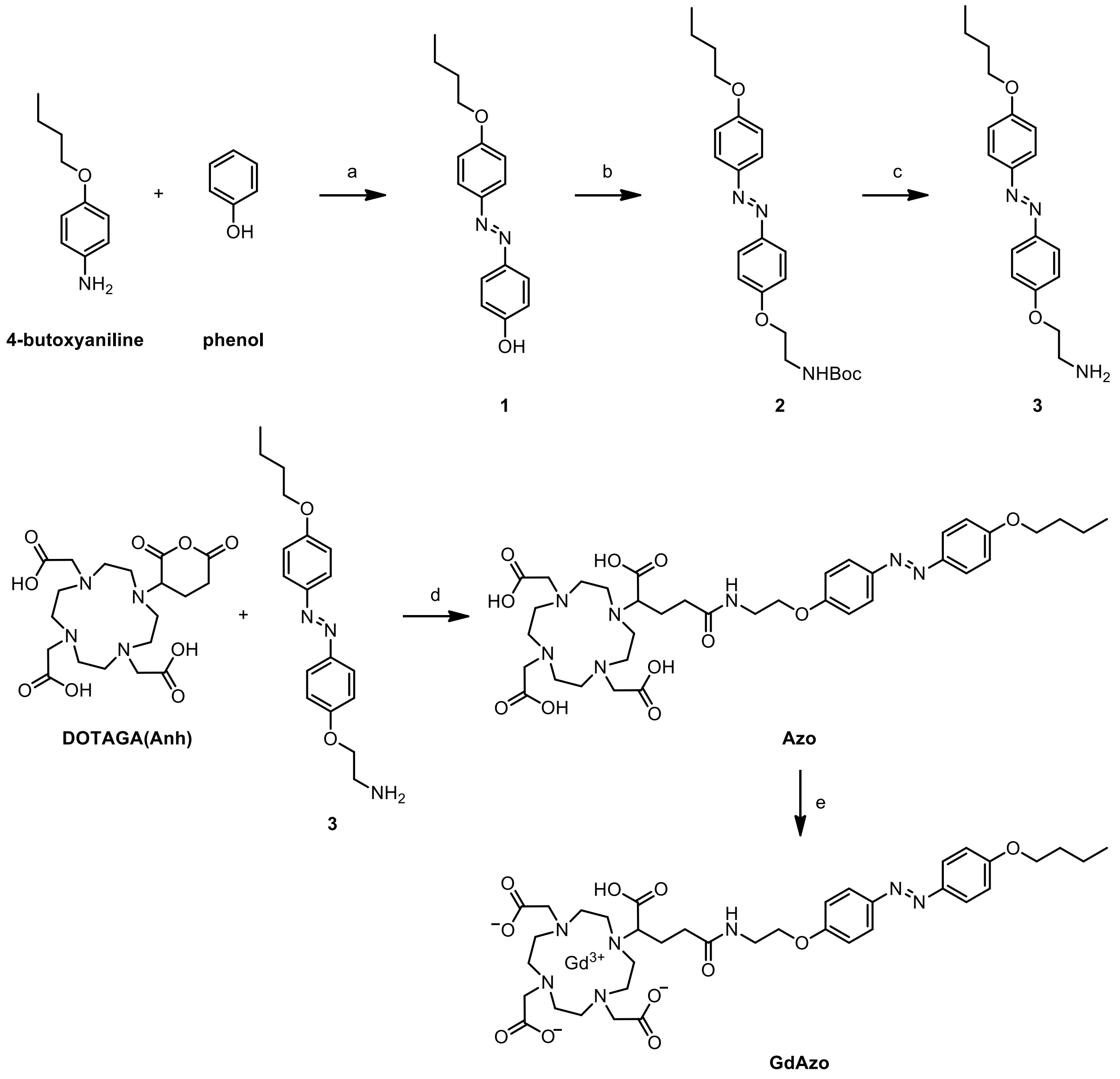

Fig. 1. Synthesis pathway to Azo and GdAzo. a. (1) 4-butoxyaniline (2.00 mL, 12.0 mmol), $NaNO_2$ (0.854 g, 12.0 mmol, 1.0 equiv), ice (12 g), HCl cc (2.6 mL) ethanol:water 1:1 (24 mL); (2) phenol (1.14 g, 12.0 mmol, 1.0 equiv), NaOH (0.960 g, 24.0 mmol, 2.0 equiv), water (6.3 mL), 20 min at 0 °C and 70 min at rt, 85% (2.76 g, 10.2 mmol). b. **1** (2.00 g, 7.40 mmol), K_2CO_3 (1.53 g, 11.1 mmol, 1.5 equiv), 2-(Boc-amino)ethyl bromide (4.98 g, 22.2 mmol, 3.0 equiv), acetone, 18 h, reflux, 74% (2.27 g, 5.49 mmol). c. **2** (1.26 g, 3.04 mmol), DCM:TFA 4:1 (63 mL), 1.5 h, rt, 96% (1.25 g, 2.93 mmol, TFA salt). d. **3** (0.259 g, 0.606 mmol), triethylamine (253 µL, 1.81 mmol, 3.0 equiv), DOTAGA anhydride (278 mg, 0.606 mmol, 1.0 equiv), anh DMF (4.3 mL), 21 h, 70 °C, 63% (294 mg, 0.381 mmol). e. **Azo** (0.200 g, 0.259 mmol), $GdCl_3.6H_2O$ (101 mg, 0.272 mmol, 1.05 equiv), water (10 mL), pH 5.5-6.0, 17 h, 50 °C, 88% (212 mg, 0.229 mmol).

Synthesis of 4-hydroxy-4’-butoxyazobenzene (1)

4-butoxyaniline (2.00 mL, 12.0 mmol) and sodium nitrite (0.854 g, 12.0 mmol, 1.0 equiv) were dissolved in ethanol:water 1:1 (24 mL) and the mixture was cooled down to 0 °C. Ice (12 g) was introduced in the mixture before careful addition of HCl cc (2.6 mL). An aqueous solution (6.3 mL) of phenol (1.14 g, 12.0 mmol, 1.0 equiv) and NaOH (0.960 g, 24.0 mmol, 2.0 equiv) previously prepared and cooled down to 0 °C was carefully introduced in the mixture at 0 °C. The mixture was stirred for 20 min at 0 °C and for 70 min at rt. After adjusting the pH to 1 (HCl cc), the mixture was left to stand for 30 min at rt and was then filtrated. The precipitate was washed with water (4x50 mL), solubilised in DCM and the organic solvent was dried over $MgSO_4$ before concentration. **1** (2.76 g, 10.2 mmol, 85%) was isolated as a black amorphous powder.

^{1}H NMR (400 MHz, $CDCl_3$) δ (ppm): 7.87 (d, J = 8.9 Hz, 2H), 7.82 (d, J = 8.8 Hz, 2H), 6.98 (d, J = 8.9 Hz, 2H), 6.91 (d, J = 8.8 Hz, 2H), 4.03 (t, J = 6.5 Hz, 2H), 1.85 – 1.75 (m, 2H), 1.58 – 1.45 (m, 2H), 0.99 (t, J = 7.4 Hz, 3H).

^{13}C NMR (75 MHz, $CDCl_3$) δ (ppm): 161.51, 158.38, 146.89, 146.63, 124.81, 124.56, 116.07, 114.92, 68.22, 31.35, 19.33, 13.96.

HRMS (m/z): calcd for $C_{16}H_{19}N_2O_2$, 271.1447 ($[M+H]^+$). Found 271.1440.

TLC (DCM): Rf = 0.36 (UV).

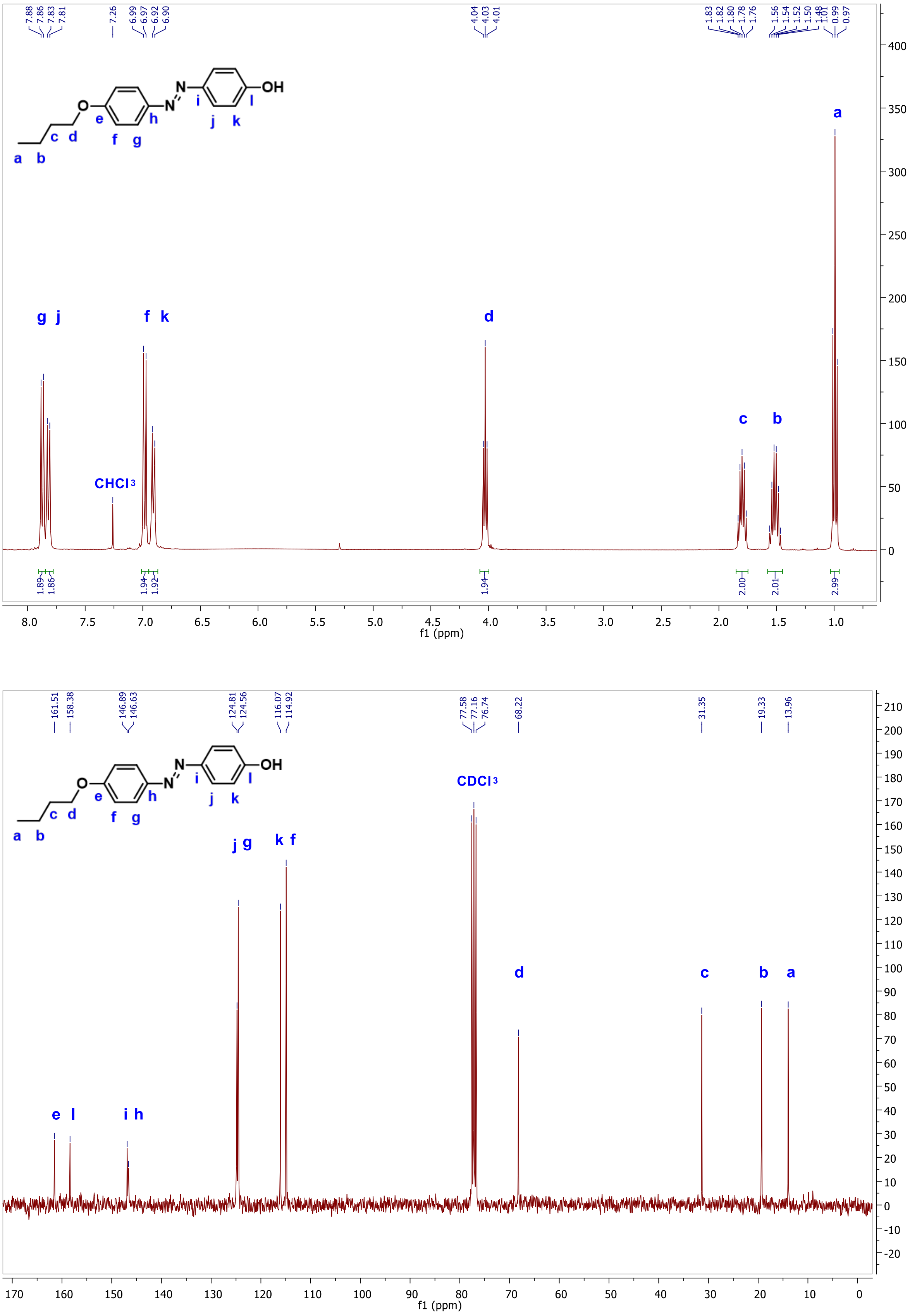

Fig. 2. ^{1}H NMR and ^{13}C NMR spectra of **1**.

Synthesis of 4-(*N*-(*tert*-butyloxycarbonyl)-ethoxyamine)-4'-butoxyazobenzene (2)

1 (2.00 g, 7.40 mmol) and K_2CO_3 (1.53 g, 11.1 mmol, 1.5 equiv) were dissolved in acetone (25 mL). After 30 min stirring at rt under argon, 2-(Boc-amino)ethyl bromide (4.98 g, 22.2 mmol, 3.0 equiv) was introduced in the mixture. After stirring at reflux for 18 h, the warmed mixture was filtrated and washed with hot acetone (75 mL). The filtrate was left to stand at rt for 30 min before filtration and the precipitate A was washed with cold acetone (20 mL). The filtrate was left to stand for 3 h at 0 °C before filtration. The precipitate B was washed with cold acetone (20 mL) and added to precipitate A. The filtrate was concentrated and purified by flash chromatography on silica gel (DCM) to increase the reaction yield. **2** (2.27 g, 5.49 mmol, 74%) was isolated as a yellow amorphous powder (57% by precipitation, 17% by flash chromatography).

^{1}H NMR (400 MHz, $CDCl_3$) δ (ppm): 7.86 (2d, J = 8.9 Hz, 4H), 6.99 (2d, J = 8.9 Hz, 4H), 4.10 (t, J = 5.0 Hz, 2H), 4.04 (t, J = 6.5 Hz, 2H), 3.57 (m, 2H), 1.87 – 1.74 (m, 2H), 1.64 – 1.40 (m, 11H), 1.00 (t, J = 7.4 Hz, 3H).

^{13}C NMR (100 MHz, $CDCl_3$) δ (ppm): 161.46, 160.62, 156.03, 147.51, 147.07, 124.52, 124.48, 114.84, 114.82, 79.79, 68.18, 67.64, 40.27, 31.42, 28.55, 19.38, 13.98.

HRMS (m/z): calcd for $C_{23}H_{32}N_3O_4$, 414.2393 ($[M+H]^+$). Found 414.2396.

TLC (DCM:MeOH, 98:2 v/v): Rf = 0.90 (UV).

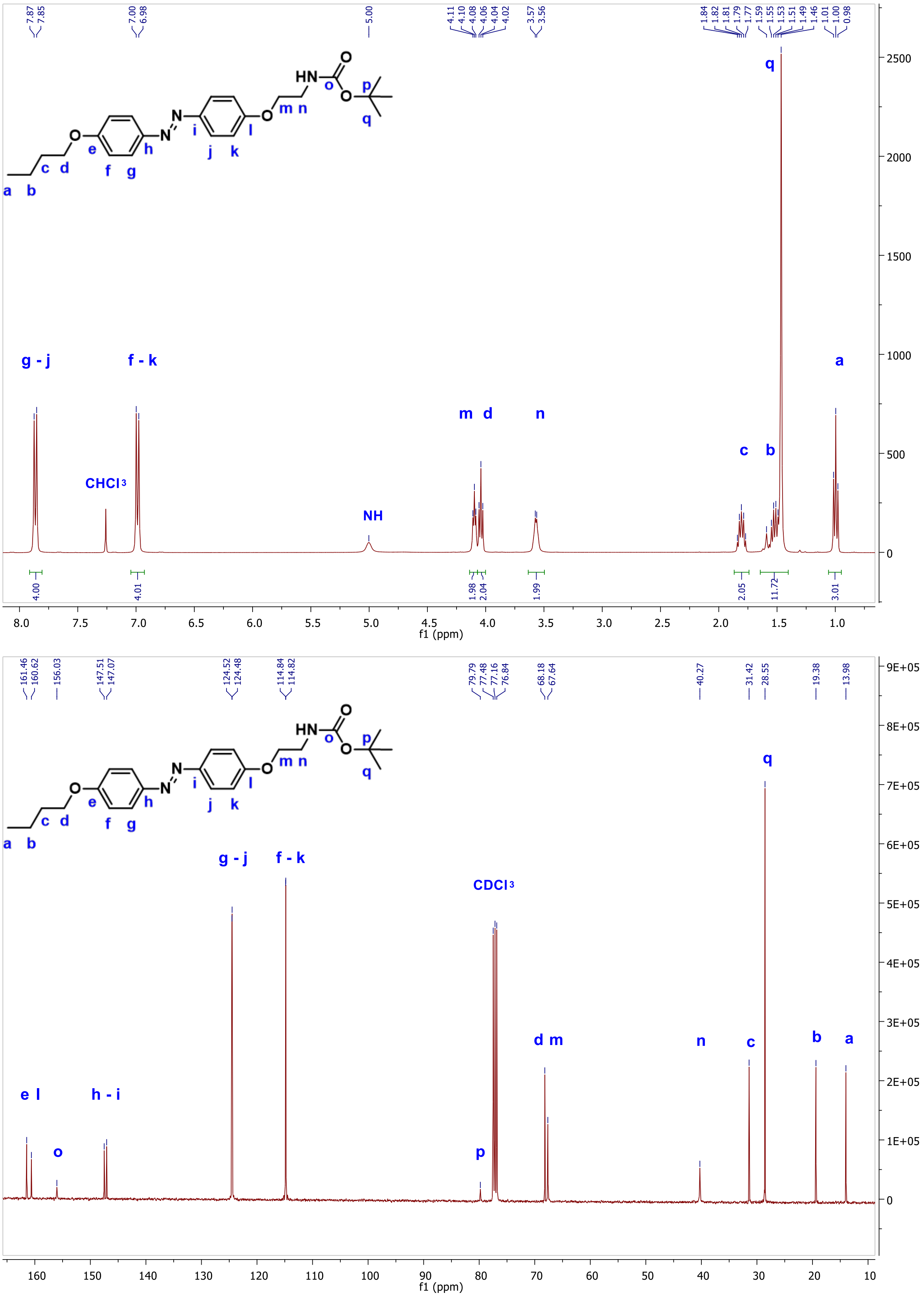

Fig. 3. ^{1}H NMR and ^{13}C NMR spectra of **2**.

Synthesis of 4-aminoethoxy-4'-butoxyazobenzene (3)

2 (1.26 g, 3.04 mmol) was dissolved in DCM:TFA 4:1 (63 mL) and the mixture was stirred for 1.5 h at rt. After concentration, diethyl ether (200 mL) was introduced and the formed precipitate was crushed for 20 min before filtration and washed with diethyl ether (4x50 mL). **3** (1.25 g, 2.93 mmol, 96%, TFA salt) was isolated as a yellow amorphous powder.

^{1}H NMR (400 MHz, MeOD) δ (ppm): 7.88 (d, *J* = 8.9 Hz, 2H), 7.85 (d, *J* = 8.9 Hz, 2H), 7.15 (d, *J* = 8.9 Hz, 2H), 7.04 (d, *J* = 8.9 Hz, 2H), 4.32 (t, *J* = 4.4 Hz, 2H), 4.06 (t, *J* = 6.4 Hz, 2H), 3.41 (t, *J* = 4.4 Hz, 2H), 1.86 – 1.73 (m, 2H), 1.60 – 1.46 (m, 2H), 1.00 (t, *J* = 7.4 Hz, 3H).

^{13}C NMR (75 MHz, MeOD) δ (ppm): 163.03, 161.34, 148.97, 148.10, 125.42, 125.30, 115.99, 115.80, 69.12, 65.59, 40.24, 32.43, 20.28, 14.16.

HRMS (m/z): calcd for $C_{18}H_{24}N_3O_2$, 314.1869 ($[M+H]^+$). Found 314.1864.

HPLC (method A): t_R 17.92 min (*cis*)-20.35 min (*trans*).

TLC (DCM:MeOH, 95:5 v/v): Rf = 0.70 (UV or ninhydrin).

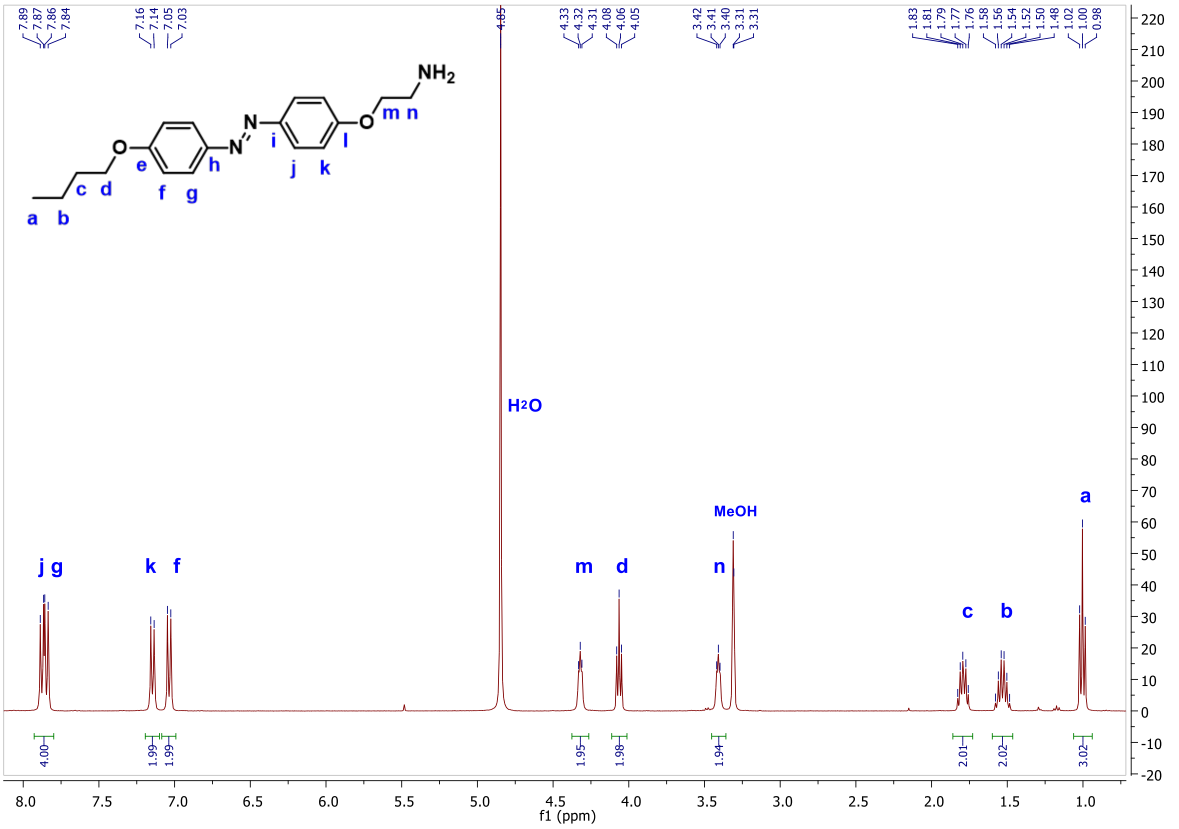

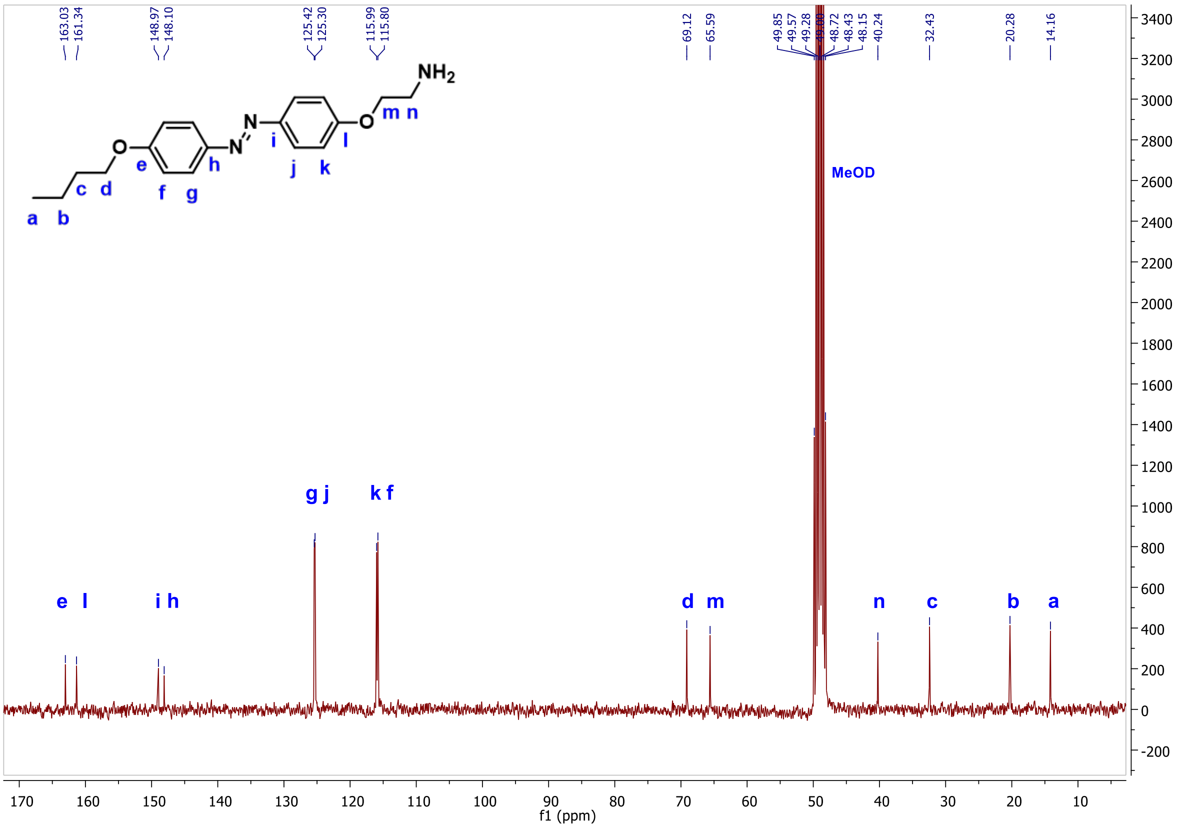

Fig. 4. ^{1}H NMR and ^{13}C NMR spectra of **3**.

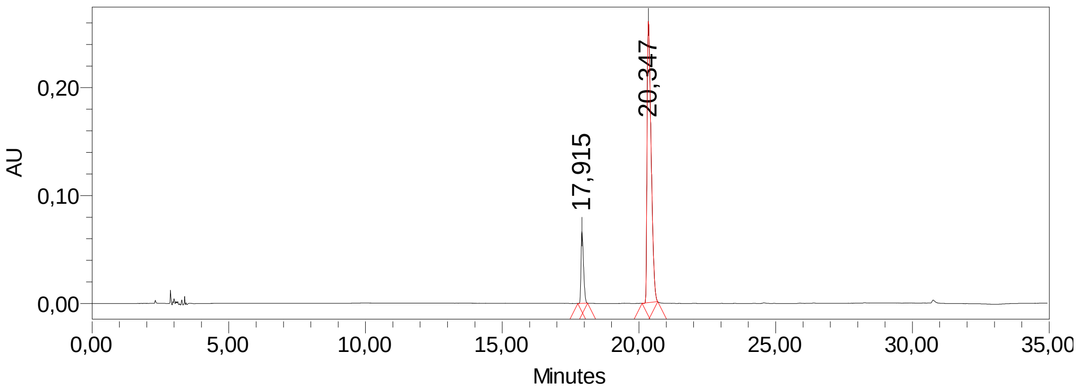

Fig. 5. HPLC chromatogram of **3**. Method A (detection at 418 nm).

Synthesis of 4-(*N*-(1,4,7,10-tetraazacyclododecane-1,4,7-triacetic acid-10-glutaryl)-ethoxyamide)-4'-butoxyazobenzene (Azo)

3 (259 mg, 0.606 mmol, TFA salt) was dissolved in anhydrous DMF (4.3 mL). After introduction of triethylamine (253 µL, 1.81 mmol, 3.0 equiv), the mixture was stirred for 5 min at rt under argon. DOTAGA anhydride (278 mg, 0.606 mmol, 1.0 equiv) was then introduced and the mixture was stirred for 21 h at 70 °C under argon. After concentration, diethyl ether (100 mL) was introduced and the formed precipitate was crushed for 30 min before filtration and washed with diethyl ether (2x50 mL). The crude was purified on reverse phase flash chromatography (RP-18, Biotage, gradient water-0.05% formic acid:acetonitrile-0.05% formic acid 1:0 to 2:8). **Azo** (294 mg, 0.381 mmol, 63%) was isolated by freeze drying as an electrostatic yellow powder.

The regioselectivity of the anhydride-opening reaction was confirmed by NOESY 2D NMR experiment (mixing time = 0.8 s, Fig. 8) which showed that the proton of the amide group (*NH*, Fig. 7) is closer to the CH_2 *p* (signal intensity 2.02, 2H) than CH_2 *q* (signal intensity 0.42, 2H) and CH_2 *t* (signal intensity 0.34, 4H). The correlation of *NH* with CH_2 *n* and CH *r* overlapped and could not be accurately exploited.

^{1}H NMR (500 MHz, DMSO) δ (ppm): 8.53 (s, 1H), 7.82 (d, J = 8.9 Hz, 2H), 7.81 (d, J = 8.9 Hz, 2H), 7.13 (d, J = 9.0 Hz, 2H), 7.09 (d, J = 9.0 Hz, 2H), 4.11 (t, J = 6.2 Hz, 2H), 4.06 (t, J = 6.5 Hz, 2H), 3.56 – 3.37 (m, 8H), 3.37 (t, J = 7.9 Hz, 1H), 3.09 – 2.83 (m, 12H), 2.76 (m, 4H), 2.38 (dt, J = 14.6, 7.2 Hz, 1H), 2.30 – 2.18 (m, 1H), 1.95 – 1.83 (m, 1H), 1.79 – 1.67 (m, 3H), 1.50 – 1.40 (m, 2H), 0.95 (t, J = 7.4 Hz, 3H).

^{13}C NMR (126 MHz, DMSO) δ (ppm): 172.68, 172.37, 170.48, 170.12, 160.95, 160.63, 146.20, 146.08, 124.14, 124.10, 115.06, 114.95, 67.64, 66.46, 62.09, 55.34, 54.48, 50.74, 50.24, 49.77, 46.95, 37.99, 32.13, 30.67, 23.40, 18.72, 13.70.

HRMS (m/z): calcd for $C_{37}H_{54}N_7O_{11}$, 772.3881 ($[M+H]^+$). Found 772.3883.

HPLC (method A): t_R 17.24 min (*cis*)-19.37 min (*trans*).

LC-MS (method D): t_R 12.357 min (*cis*) (m/z 772.99 $[M+H]^+$) - 14.437 min (*trans*) (m/z 772.98 $[M+H]^+$).

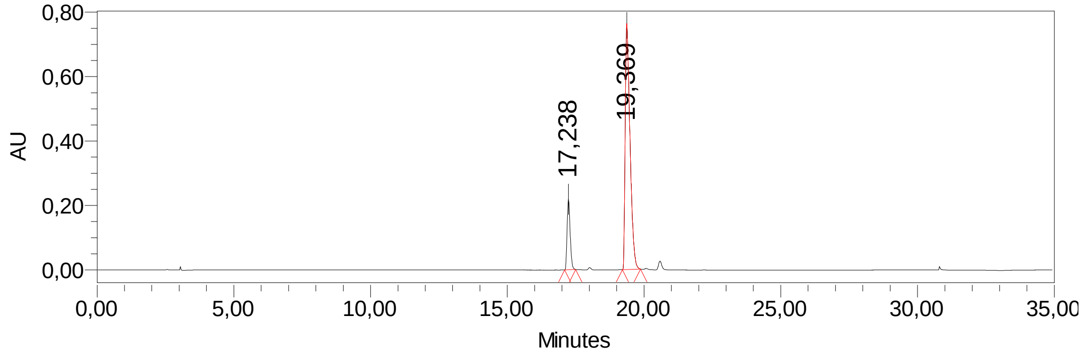

Fig. 6. HPLC chromatogram of **Azo**. Method A (detection at 418 nm).

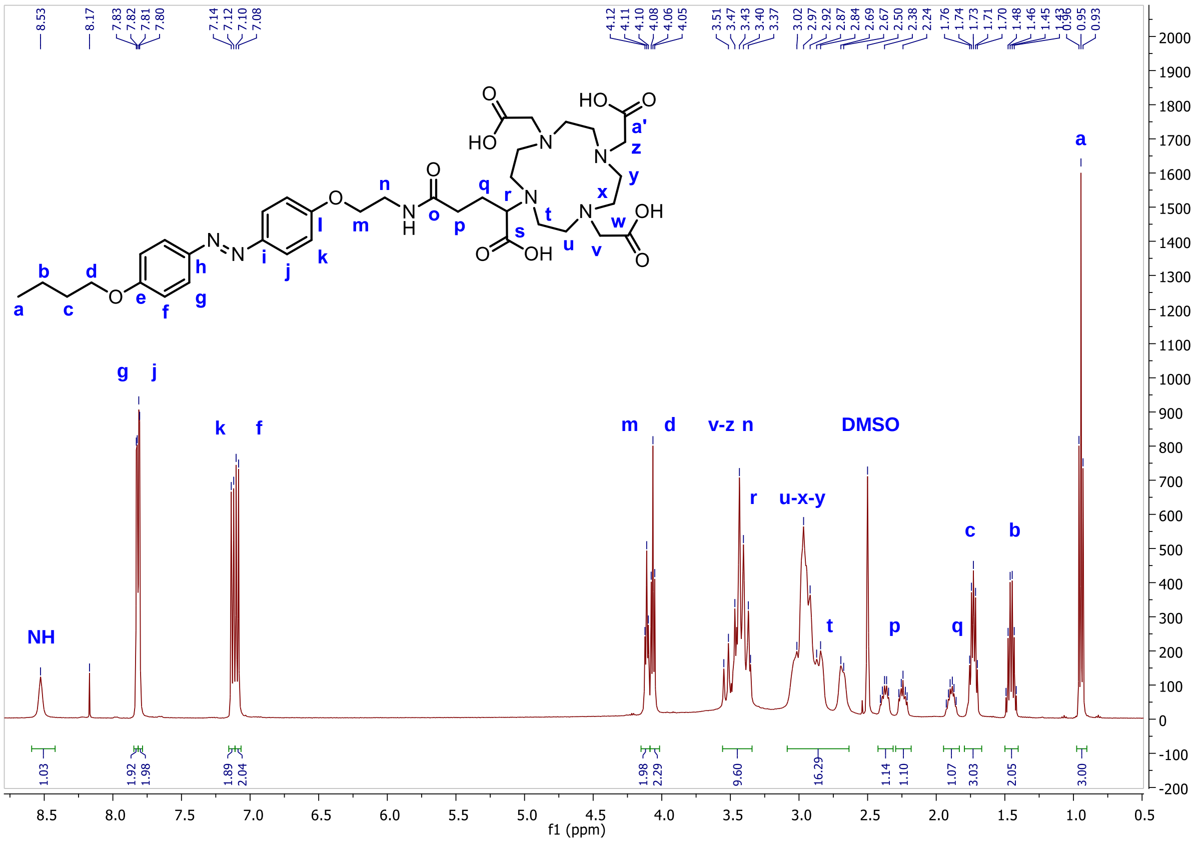

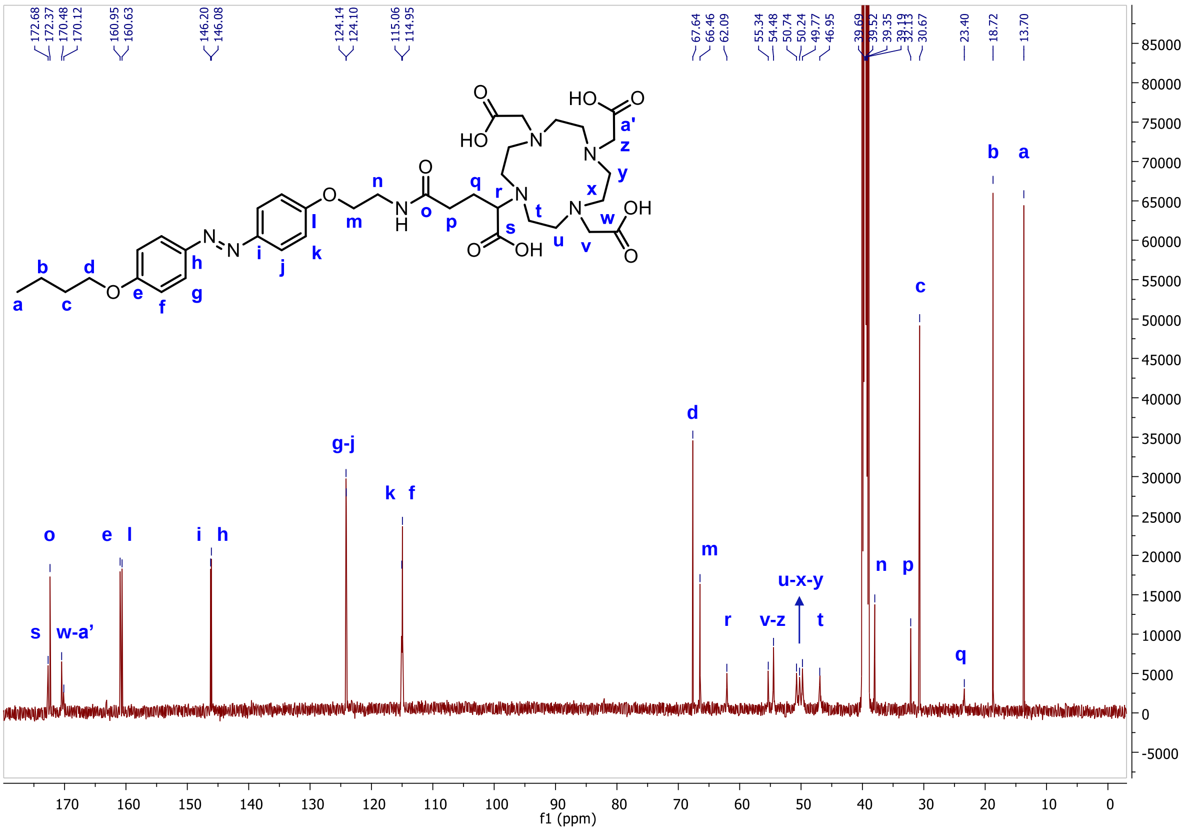

Fig. 7. ^{1}H NMR and ^{13}C NMR spectra of **Azo**.

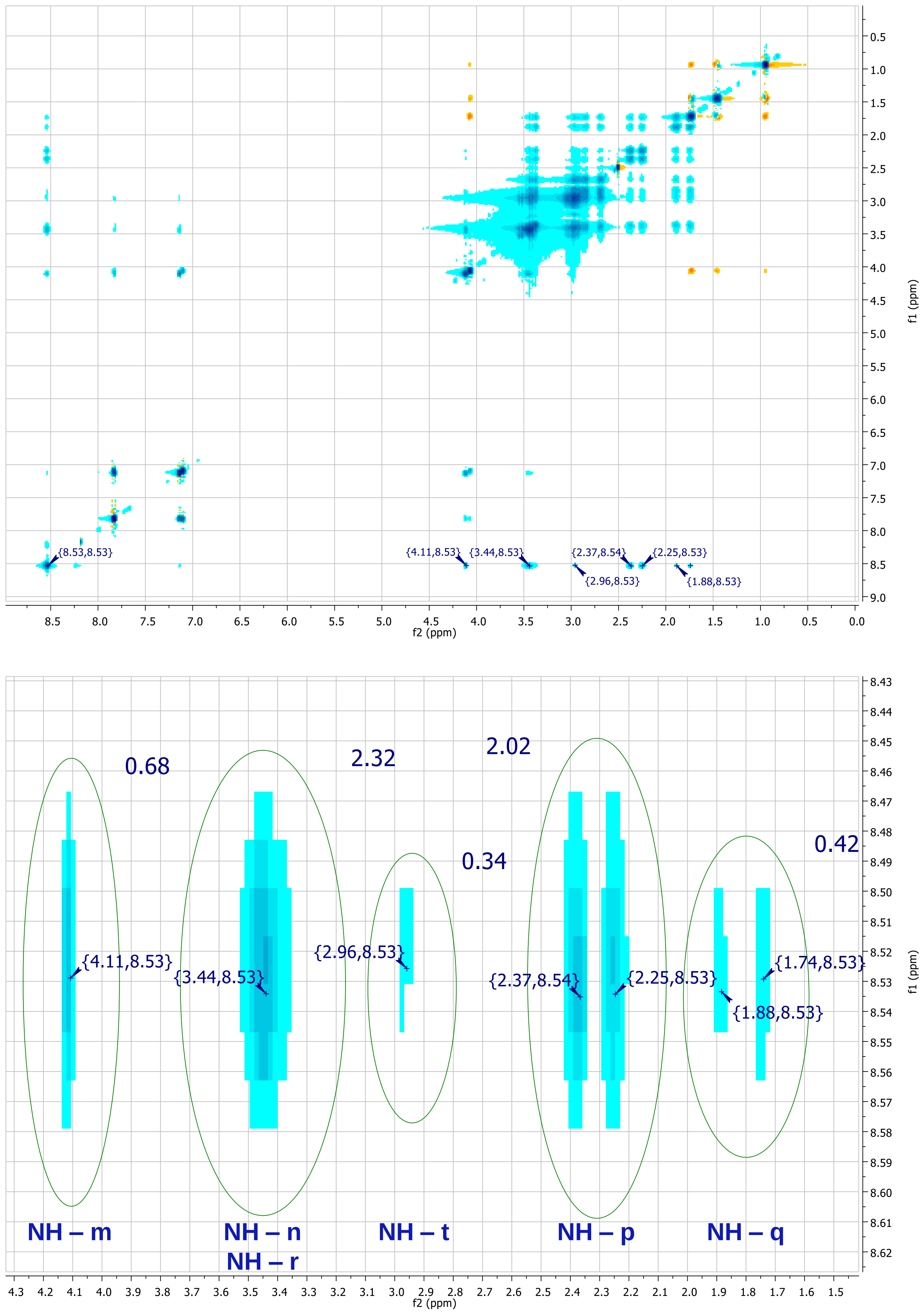

Fig. 8. NOESY spectra of **Azo**. The enlargement of the signals around 8.3 ppm is presented at the bottom. The intensity and correlation are indicated above and below each signal.

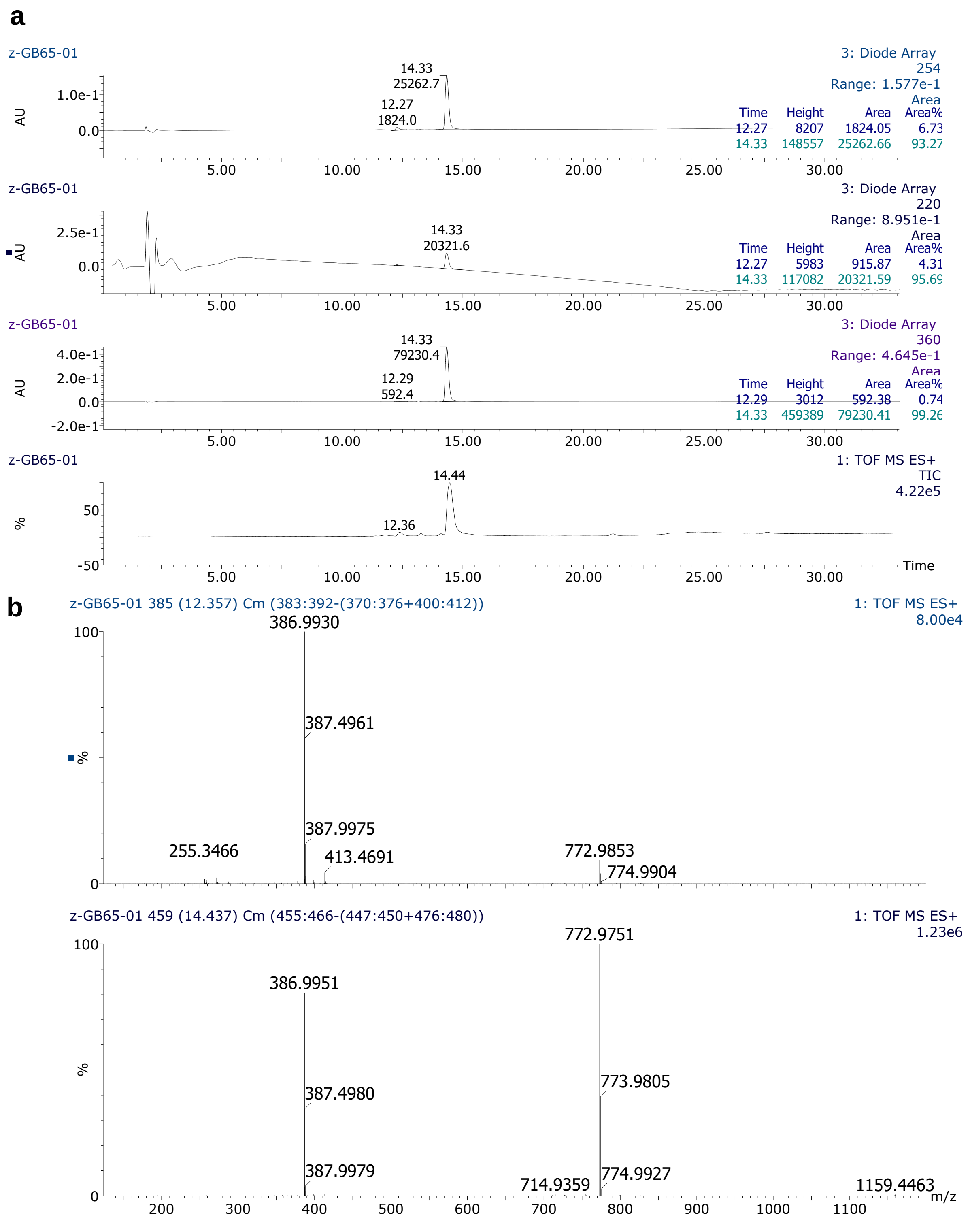

Fig. 9. LC-MS of Azo. a, Chromatograms of **Azo** at 254 nm, 220 nm, 360 nm and total ion current (positive mode) from top to bottom (method D). **b**, Mass spectra (positive mode) of *cis*-**Azo** (top, t_R = 12.357 min; m/z 772.9853 $[M+H]^+$) and *trans*-**Azo** (bottom, t_R = 14.437 min; m/z 772.9751 $[M+H]^+$).

Synthesis of Gadolinium 4-(*N*-(1,4,7,10-tetraazacyclododecane-1,4,7-triacetic acid-10-glutaryl)-ethoxyamide)-4'-butoxyazobenzene (GdAzo)

Azo (200 mg, 0.259 mmol) and $GdCl_3.6H_2O$ (101 mg, 0.272 mmol, 1.05 equiv) were dissolved in MiliQ water (10 mL). After adjusting the pH to 5.5, the mixture was stirred for 17 h at 50 °C controlling that pH remains in the 5.5-6.0 range. Then, the pH was adjusted to 6.5 before introduction of Chelex 100 sodium form resin in the mixture. After 1 h stirring, the Chelex resin was removed by filtration and washed with MeOH (3x30 mL). The filtrate was concentrated and purified on reverse phase flash chromatography (RP-18, Biotage, gradient water/acetonitrile 1:0 to 1:1). **GdAzo** (212 mg, 0.229 mmol, 88%) was isolated by freeze drying as an electrostatic yellow powder.

HRMS (m/z): calcd for $C_{37}H_{49}N_7Na_2O_{11}Gd$, 971.2527 ($[M\text{-}H\text{+}2Na]^+$). Found 971.2529.

HPLC (method A): t_R 17.18 min (*cis*)-20.60 min (*trans*).

LC-MS (method D): t_R 15.956 min (*cis*) (m/z 927.29 $[M+H]^+$) - 19.633 min (*trans*) (m/z 927.29 $[M+H]^+$).

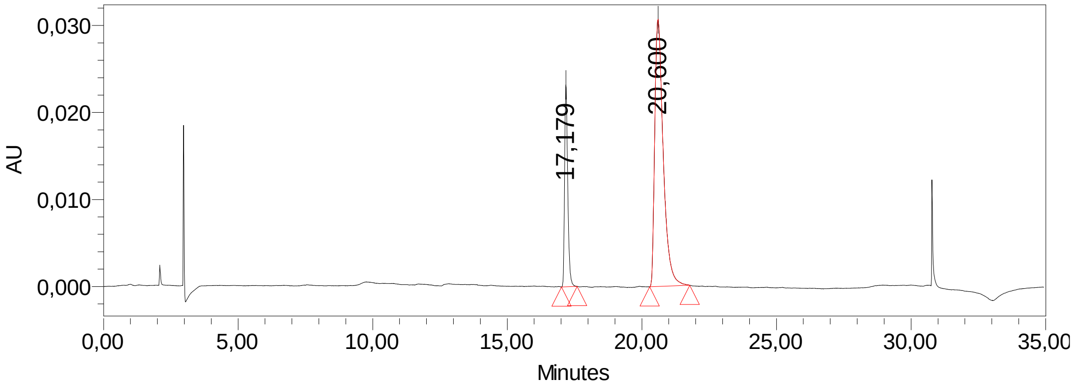

Fig. 10. HPLC chromatogram of **GdAzo**. Method A (detection at 418 nm).

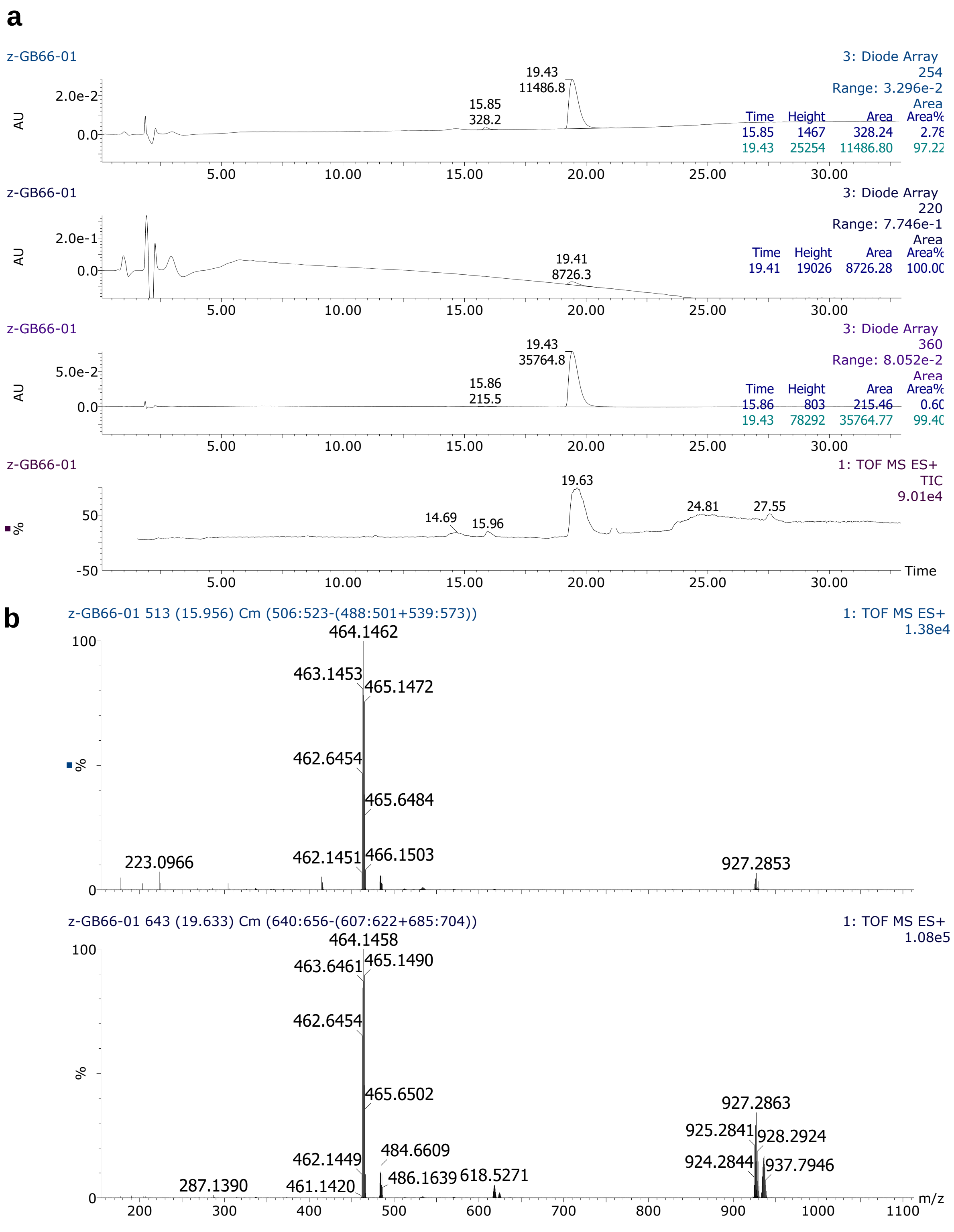

Fig. 11. LC-MS of GdAzo. a, Chromatograms of **GdAzo** at 254 nm, 220 nm, 360 nm and total ion current (positive mode) from top to bottom (method D). **b**, Mass spectra (positive mode) of *cis*-**GdAzo** (top, t_R = 15.956 min; m/z 927.2853 $[M+H]^+$) and *trans*-**GdAzo** (bottom, t_R = 19.633 min; m/z 927.2863 $[M+H]^+$).

Determination of free Gd^{3+} content

A colorometric test with Arsenazo III (Sigma Aldrich) was used to determine the amount of free Gd^{3+} contained in the **GdAzo** compound. **GdAzo** (10 µL, 750 µM in water), MilliQ water (140 µL), buffer (40 µL, triethanolamine 1 M:nitric acid 0.5 M 2:3 pH 7.85) and Arsenazo III (10 µL, 4 mM in water) were introduced in 96-well microplate (triplicate). The Arsenazo III solution has to be introduced at the latest step at the same time in each well. After 30 min at rt in the dark, absorbance (650 nm) was determined in each well. For the standard calibration curve, the **GdAzo** solution was replaced by a Gd standard solutions (0.5-50 µM in water). The **GdAzo** compound contained 1.26 ± 0.08 mol% (mean ± standard deviation) of free Gd^{3+}, a content which was neglected.

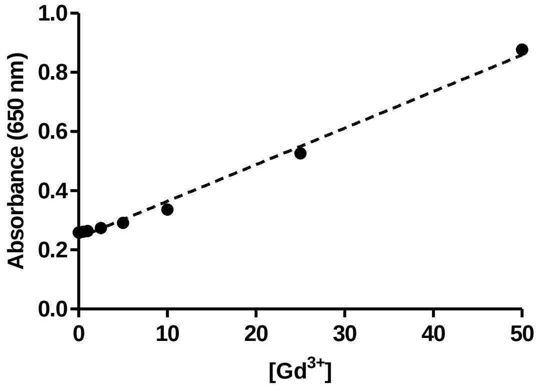

Fig. 12. Standard curve for the determination of free Gd^{3+} content. A linear regression was performed (least-squares method, slope: 0.01236; Y-intercept: 0.2408; r^2: 0.9925) with GraphPad (GraphPad Prism 5.00). The absorbance values measured for the experiment with the **GdAzo** compound were 0.357, 0.352 and 0.366.

Supplementary Section 3

Isomerisation monitoring

3.1 – Absorbance and ¹H NMR monitoring

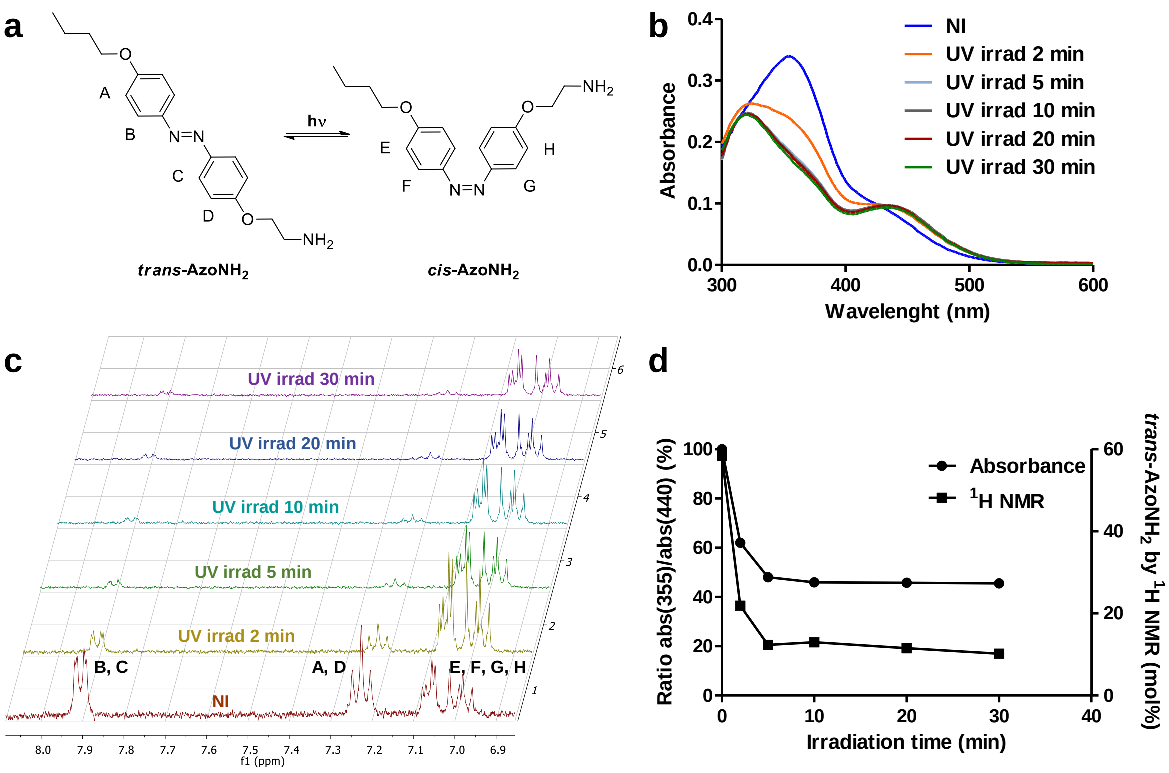

Fig. 13. Isomerisation of AzoNH2 (synthetic precursor of GdAzo) upon UV irradiation observed by UV-vis spectrophotometer and ¹H NMR. a, Structure of *trans*-AzoNH2 and *cis*-AzoNH2, assigned as **3** in Supplementary Section 2. **b**, **c**, Absorbance spectra (**b**) and corresponding ¹H NMR spectra (**c**) of Azo-NH2 (mixture of *cis* and *trans*-isomers) upon UV irradiation (365 nm, 49.0 mJ/cm²/min) in phosphate buffered saline (PBS). NI: non-irradiated. **d**, Azo-NH2 absorbance (mixture of *cis* and *trans*-isomers, left axis) and *trans*-AzoNH2 amount determined by ¹H NMR (mol%, right axis) upon UV irradiation (365 nm, 49.0 mJ/cm²/min) in PBS. The absorbance signal ratio was normalised to the signal of the NI mixture.

3.2 – Thermal back relaxation of *cis*-GdAzo

cis-**GdAzo** was obtained by UV irradiation (365 nm, 0.817 mW.cm^{-2}, 30 min) of *trans*-**GdAzo** (200 µL, 100 µM, PBS, *cis*-**GdAzo** 91%) in 96-well microplate. *cis*-**GdAzo** was maintained in the dark at 37 °C and absorbance (5 µM in quartz cuve, Perkin Elmer Lambda 25 UV/VIS spectrometer) and HPLC (method B) analyses were performed at different time points. The thermal half-life of *cis*-**GdAzo** was determined by HPLC-data treatment using nonlinear regression with one phase decay equation and least-squares fitting method (GraphPad Prism 5.00).

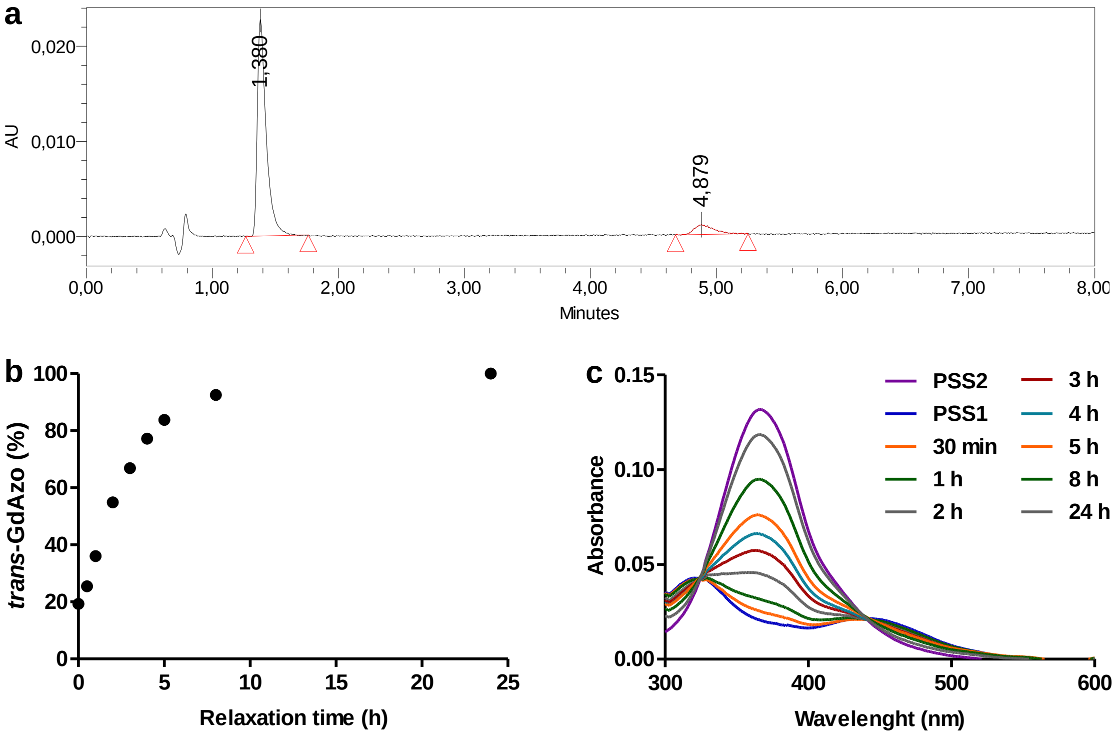

Fig. 14. Thermal back relaxation of *cis*-GdAzo at 37 °C in PBS. a, HPLC chromatogram (method B, 320 nm) of *cis*-**GdAzo** obtained by UV irradiation (365 nm, 0.817 mW.cm^{-2}, 30 min) of *trans*-**GdAzo**. The peak 1 (1.380 min, area 111645, 90.98%) and the peak 2 (4.879 min, area 11189, 9.11%) are *cis*-**GdAzo** and *trans*-**GdAzo** respectively. **b**, **c**, Monitoring of *cis*-**GdAzo** thermal back relaxation by HPLC (**b**) and absorbance (**c**) measurements. The photostationary states (PSS) 1 and 2 refer to media containing a majority of *cis* and *trans*-**GdAzo** isomers respectively. The thermal half-life determined by HPLC was $t_{1/2}$ = 2.3 h (GraphPad Prism 5.00).

Supplementary Section 4

Irradiation sources

UV irradiation. UV irradiation to isomerise the *trans*-isomers into the *cis*-isomers was performed with a CN-15.LC chamber (Vilber Lourmat) equipped with two 15 W tubes (365 nm, black light blue) and delivering an irradiance of 0.817 mW/cm^2 at the position of irradiation (determined by a Cole-Parmer VLX-3W microprocessor-controlled radiometer calibrated for 365 nm, Vilber Lourmat). The terms "*cis*-**GdAzo**" and "*cis*-**Azo**" are used to designate the *cis*-rich isomer mixture for easier reading.

Gamma-ray irradiation. Irradiation upon gamma-rays was performed with a Cesium-137 source (IBL 637) delivering photons at energy of 662 keV with a dose rate about 1 Gy/min. The irradiation dose (± 5%, gafchromic film dosimetry, confirmed by ionisation chamber) is expressed in Gray (1 Gy=1 J/L). It has to be noted that the doses delivered by this source was lowered by 7.2 to 8.1% (2, 5, 10 and 20 Gy refer to the real doses of 1.84, 4.61, 9.26 and 18.56 Gy), but this offsetting was corrected to calculate the *G*-values (for $G(HO^{\bullet})$ and *G*(*trans*-**GdAzo**) in µmol/J).

X-ray irradiation. Irradiation upon X-rays was performed with a X-ray generator (Xrad 320 Dx) delivering photons at mean energy of 80 keV (30-140 keV range) with a dose rate about 1 Gy/min (operated at 200 kV, 20 mA, filtration: 0.3 mm Cu + 1 mm Al). The irradiation dose (± 5%, gafchromic film dosimetry, confirmed by ionisation chamber) is expressed in Gray.

Electron-beam irradiation. Electron-beam irradiation was performed with a linear accelerator (LINAC, Kinetron) delivering electrons at energy of 4.5 MeV with a dose rate about 4 Gy/min. The irradiation dose (±5 %, gafchromic film dosimetry) is expressed in Gray.

Supplementary Section 5

Characterisation of *cis*-GdAzo activation upon ionising radiations

General information

Absorbance was measured in 96-well microplates on a FLUOstar OMEGA plate-reader (OMEGA version 5.70 R2 and MARS version 4.01 R2 software). The irradiation devices used (UV and ionising-radiation sources) are described in the Supplementary Section 4. The high-performance liquid chromatography (HPLC) methods are described in the Supplementary Section 1. Phosphate buffered saline (PBS) was purchased from Sigma Aldrich (France).

5.1 - Quantification of *cis*-GdAzo activation upon ionising radiations

The gadolinium-containing compound (*trans*-**GdAzo**) and the control compound (*trans*-**Azo**) (50 µM, 200 µL, PBS) were introduced in two 96-well microplates (8 wells for each compound). Both microplates were irradiated by UV (365 nm, 0.817 mW.cm^{-2}, 5 min) to obtain the photostationary state 1 (PSS1) containing a majority of the *cis*-isomer (90 ± 3%). One microplate (plate 1) was kept in the dark and used as control (non-irradiated by ionising radiation) whereas the second microplate (plate 2) was irradiated upon incremental doses of ionising radiations (2, 3, 5 and 10 Gy). After each irradiation, absorbance and HPLC (method C) analyses were performed on the non-irradiated (plate 1) and irradiated (plate 2) compounds. A delay of 26 min between the UV irradiation of plate 1 and plate 2 was introduced to analyse the non-irradiated control compounds (plate 1) at the same time as the compounds irradiated by ionising radiations (plate 2) after UV irradiation. The full experiment was performed in about 4.5 h. The absorbance spectra were drawn from a mean of triplicate. The relative amount of each isomer was obtained by running HPLC (method C) at the isobestic-point conditions (mobile phase and detection wavelength) and the molecular activation (%) was determined by the difference in proportion of the *trans*-isomer in the media. The experiment was repeated 3 times independently. It has to be noted that the conversion observed for the non-irradiated compounds was due to thermal back relaxation.

5.1.a - Characterisation by absorbance

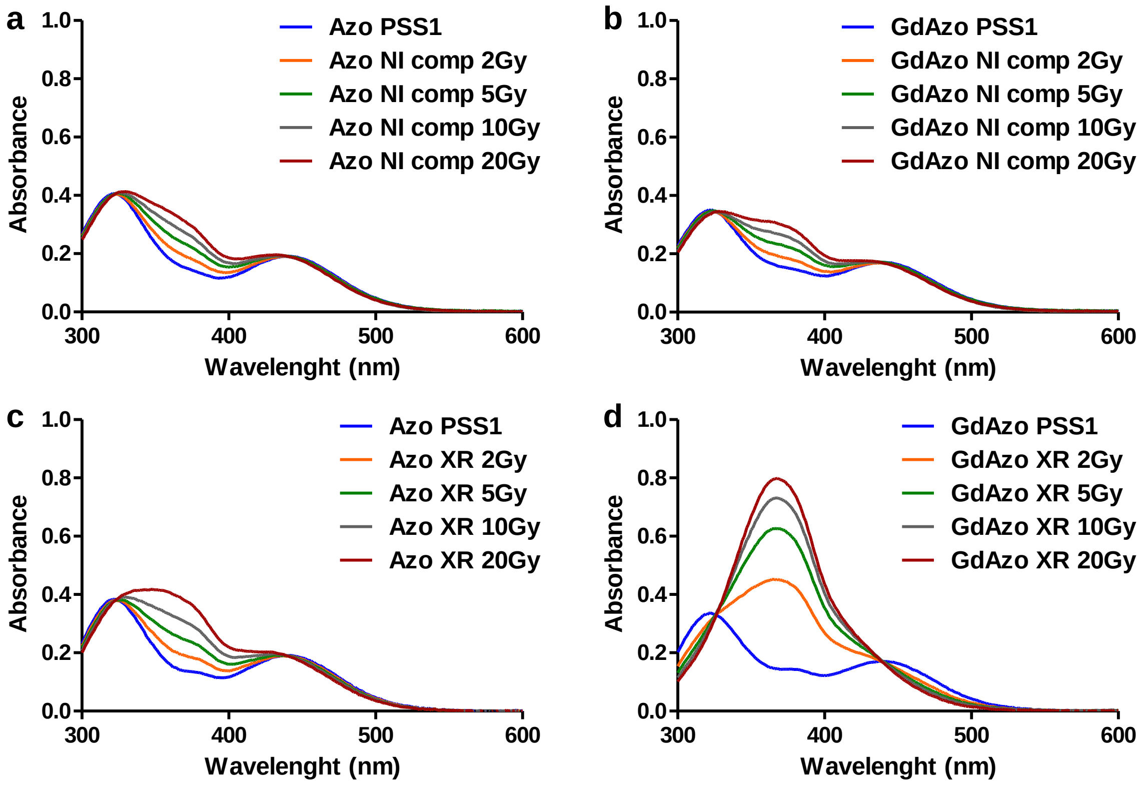

Fig. 15. Absorbance spectra of non-irradiated *cis*-**Azo** compound (**a**), non-irradiated *cis*-**GdAzo** compound (**b**), irradiated *cis*-**Azo** compound (**c**) and irradiated *cis*-**GdAzo** compound (**d**) (50 µM in PBS, X-ray (XR) irradiation). The spectra were drawn from a mean of triplicate. The photostationary state 1 (PSS1), containing a majority of the *cis*-isomer, was obtained by UV irradiation (365 nm, 0.817 mW/cm^2, 5 min). "NI comp" (**a** and **b**) refers to a non-irradiated compound analysed at the same time as the XR-irradiated compound after UV irradiation at the mentioned dose. The presence of isobestic points means that only two species contribute to the absorption (the *cis* and *trans* isomers).

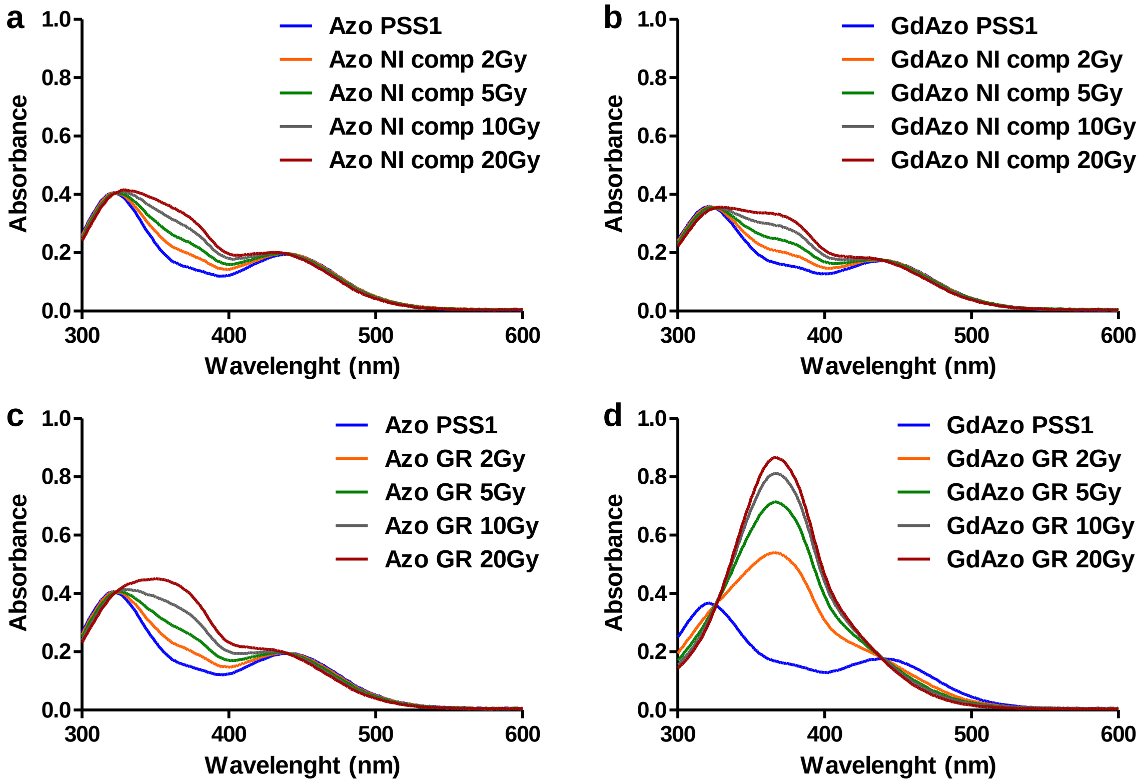

Fig. 16. Absorbance spectra of non-irradiated *cis*-**Azo** compound (**a**), non-irradiated *cis*-**GdAzo** compound (**b**), irradiated *cis*-**Azo** compound (**c**) and irradiated *cis*-**GdAzo** compound (**d**) (50 µM in PBS, gamma-ray (GR) irradiation). The spectra were drawn from a mean of triplicate. The photostationary state 1 (PSS1), containing a majority of the *cis*-isomer, was obtained by UV irradiation (365 nm, 0.817 mW/cm^2, 5 min). "NI comp" (**a** and **b**) refers to a non-irradiated compound analysed at the same time as the GR-irradiated compound after UV irradiation at the mentioned dose. The presence of isobestic points means that only two species contribute to the absorption (the *cis* and *trans* isomers).

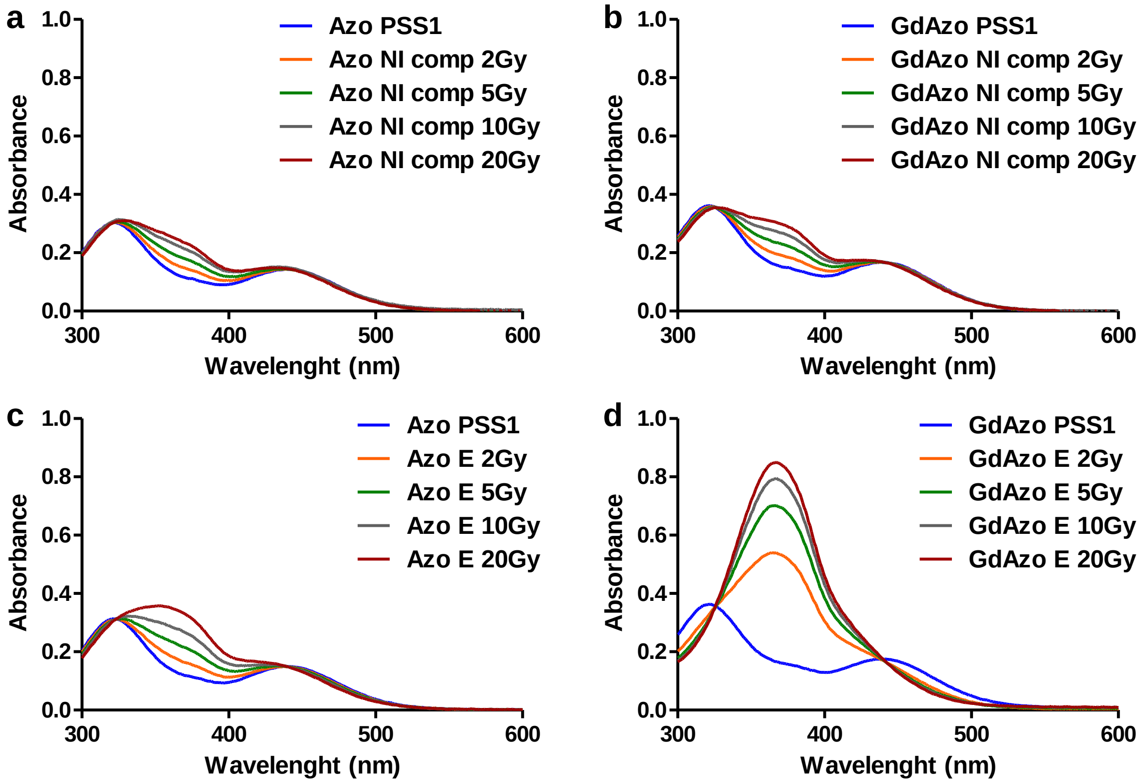

Fig. 17. Absorbance spectra of non-irradiated *cis*-**Azo** compound (**a**), non-irradiated *cis*-**GdAzo** compound (**b**), irradiated *cis*-**Azo** compound (**c**) and irradiated *cis*-**GdAzo** compound (**d**) (50 µM in PBS, electron-beam (E) irradiation). The spectra were drawn from a mean of triplicate. The photostationary state 1 (PSS1), containing a majority of the *cis*-isomer, was obtained by UV irradiation (365 nm, 0.817 mW/cm^2, 5 min). "NI comp" (**a** and **b**) refers to a non-irradiated compound analysed at the same time as the E-irradiated compound after UV irradiation at the mentioned dose. The presence of isobestic points means that only two species contribute to the absorption (the *cis* and *trans* isomers).

5.1.b - Characterisation by HPLC

5.1.b.1 - X-ray irradiation

Figs. 18-20. HPLC chromatograms of *cis*-Azo upon X-ray irradiation. Figs. 18, 19 and 20 are the repetitions of three independent experiments. **a**, HPLC chromatograms (319 nm, isobestic point, method C) of a solution of *cis*-**Azo** (50 µM in PBS, first chromatogram at the top) at the time of the corresponding experiments (**b**) upon X-ray irradiations (controls of 2, 3, 5 and 10 Gy irradiations from the second to the fifth chromatogram) (non-irradiated plate 1). **b**, HPLC chromatograms (319 nm, isobestic point, method C) of a solution of *cis*-**Azo** (50 µM in PBS, first chromatogram at the top) after successive X-ray irradiations of 2, 3, 5 and 10 Gy (from the second to the fifth chromatogram) (irradiated plate 2). The cumulative doses in the solutions are 0, 2, 5, 10 and 20 Gy from the top to the bottom chromatogram. The retention times of the *cis*-**Azo** and *trans*-**Azo** compounds are 2.08-2.15 min and 10.38-10.77 min respectively (areas of the peaks are assigned).

Figs. 21-23. HPLC chromatograms of *cis*-GdAzo upon X-ray irradiation. Figs. 21, 22 and 23 are the repetitions of three independent experiments. **a**, HPLC chromatograms (320 nm, isobestic point, method C) of a solution of *cis*-**GdAzo** (50 µM in PBS, first chromatogram at the top) at the time of the corresponding experiments (**b**) upon X-ray irradiations (controls of 2, 3, 5 and 10 Gy irradiations from the second to the fifth chromatogram) (non-irradiated plate 1). **b**, HPLC chromatograms (320 nm, isobestic point, method C) of a solution of *cis*-**GdAzo** (50 µM in PBS, first chromatogram at the top) after successive X-ray irradiations of 2, 3, 5 and 10 Gy (from the second to the fifth chromatogram) (irradiated plate 2). The cumulative doses in the solutions are 0, 2, 5, 10 and 20 Gy from the top to the bottom chromatogram. The retention times of the *cis*-**GdAzo** and *trans*-**GdAzo** compounds are 1.55-1.65 min and 5.93-6.25 min respectively (areas of the peaks are assigned).

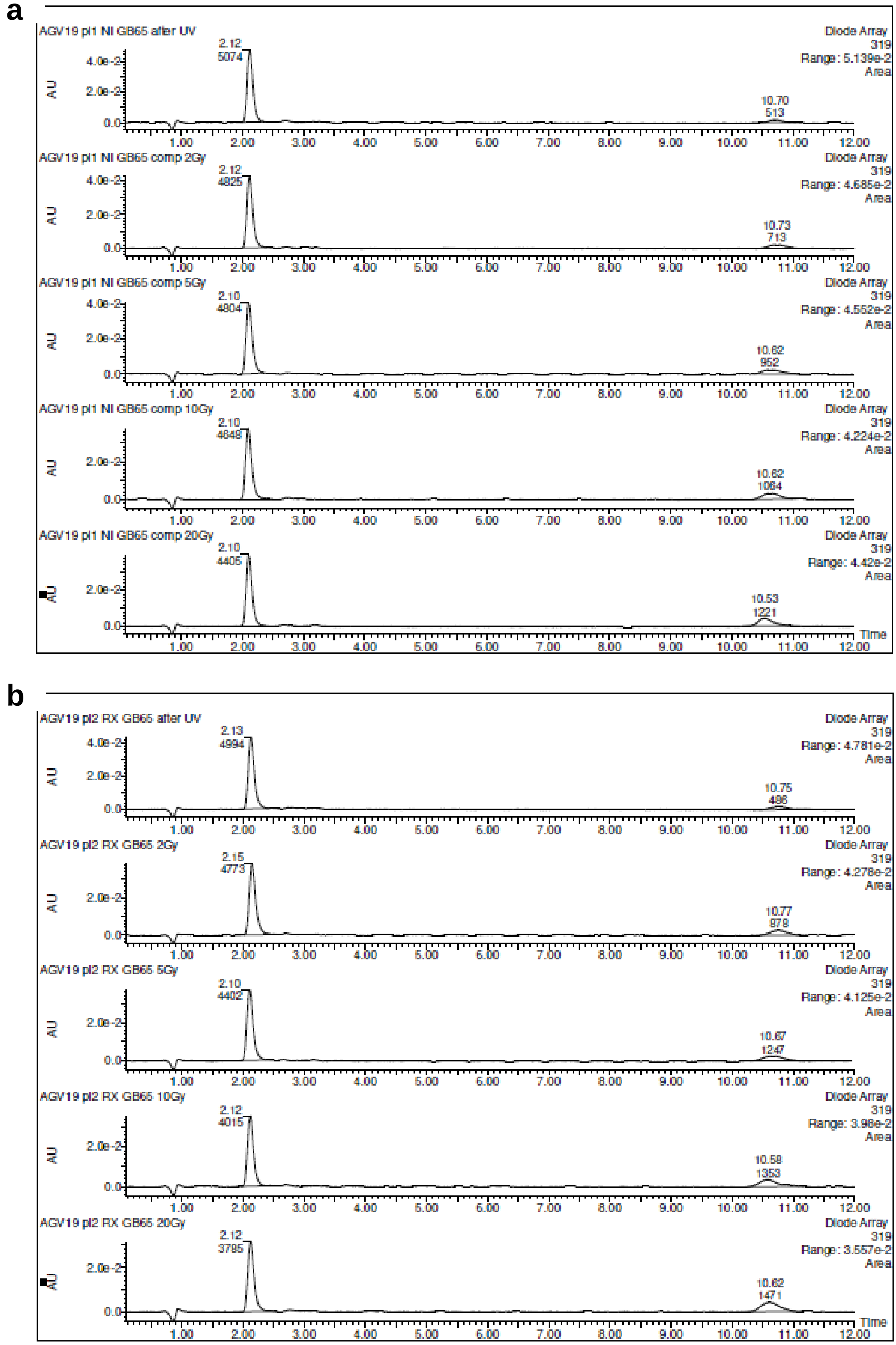

Fig. 18. HPLC chromatograms of *cis*-Azo upon X-ray irradiation. Exp. 1.

a

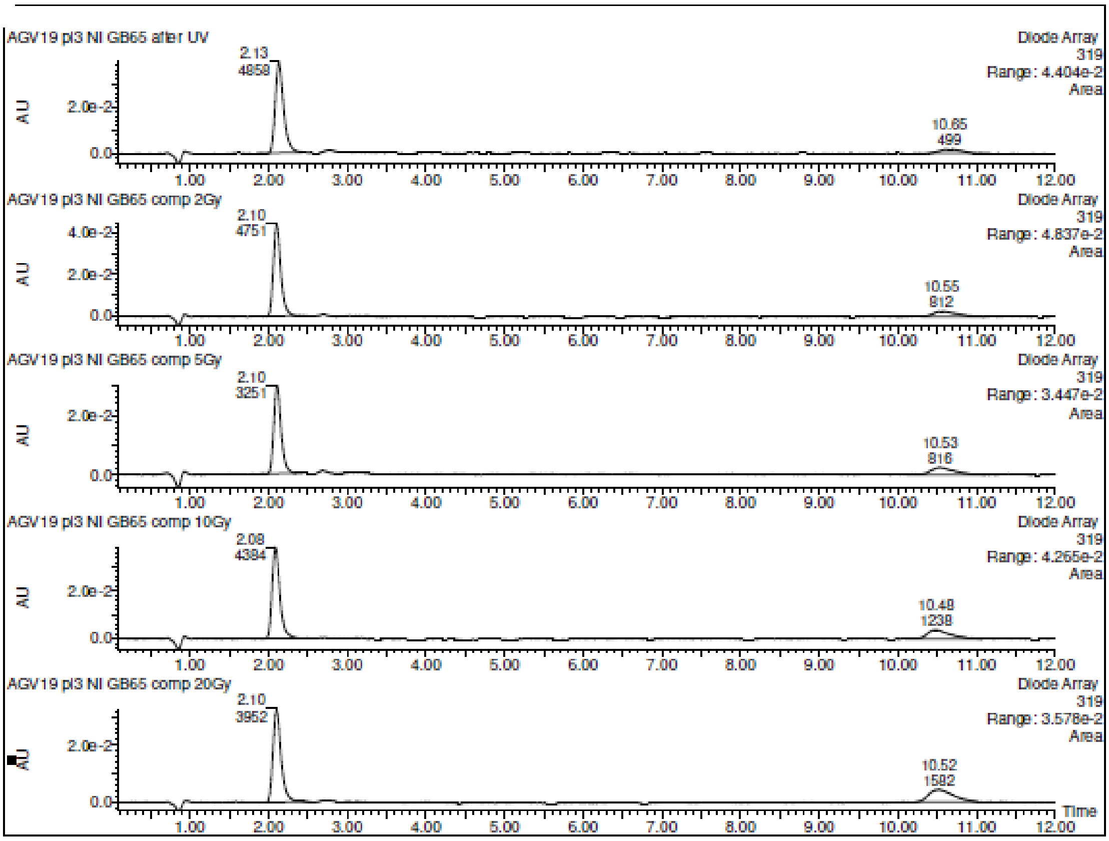

b

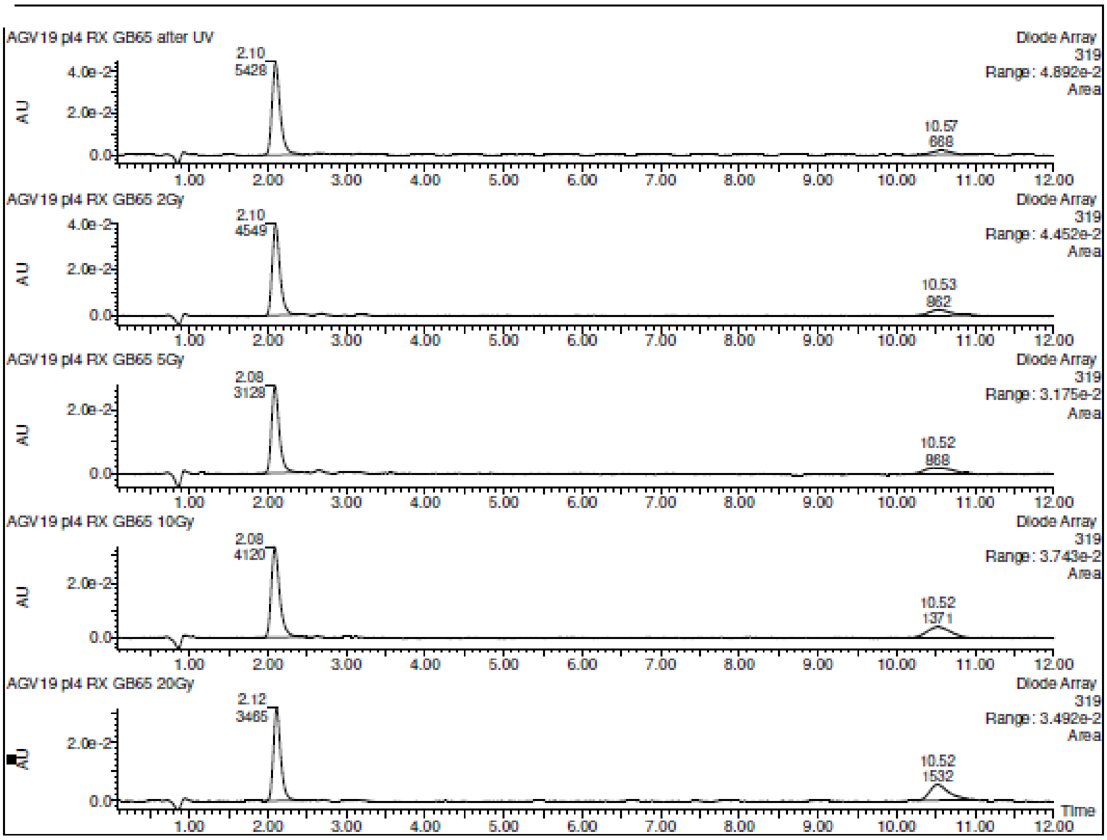

Fig. 19. HPLC chromatograms of *cis*-Azo upon X-ray irradiation. Exp. 2.

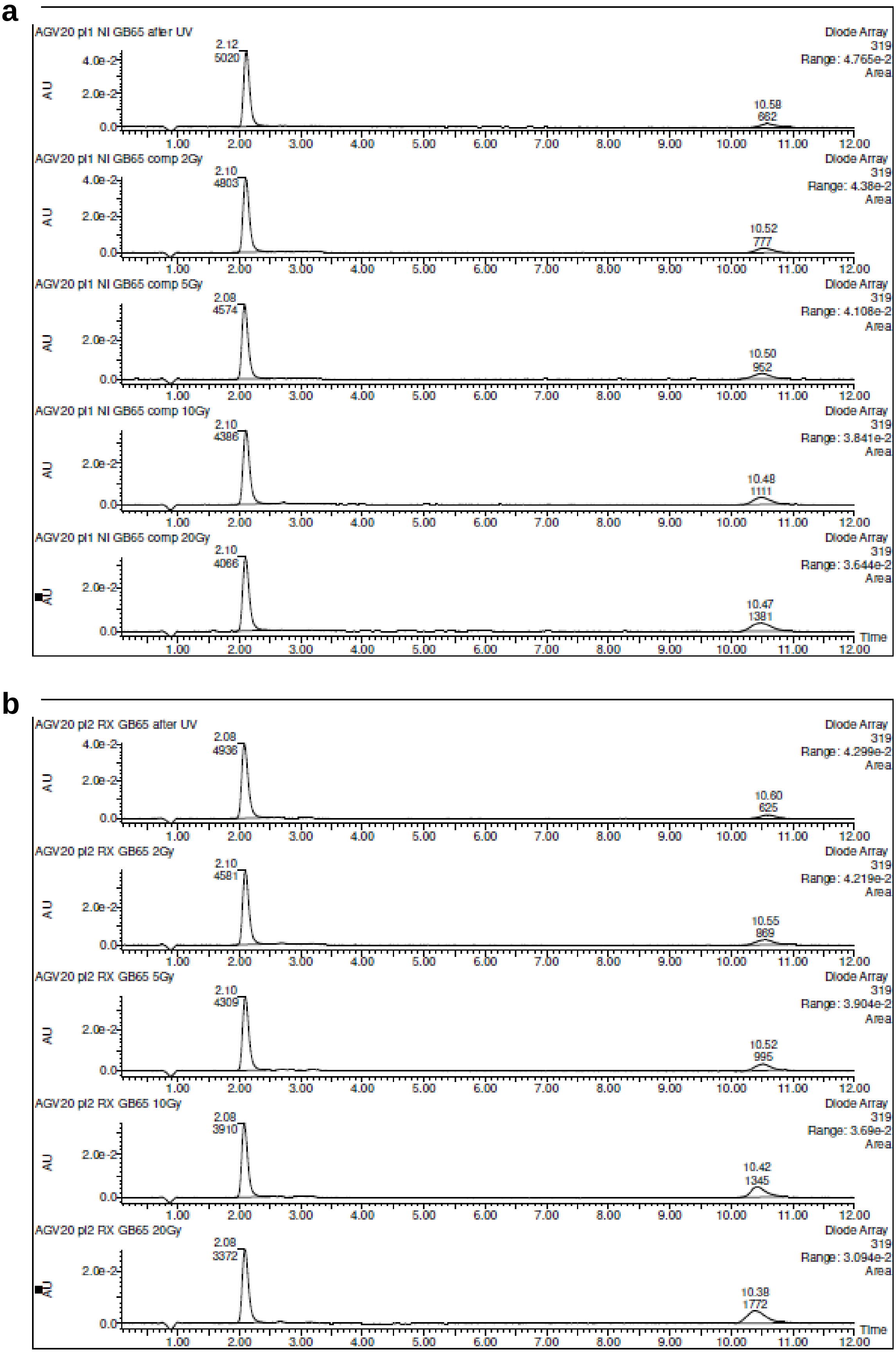

Fig. 20. HPLC chromatograms of *cis*-Azo upon X-ray irradiation. Exp. 3.

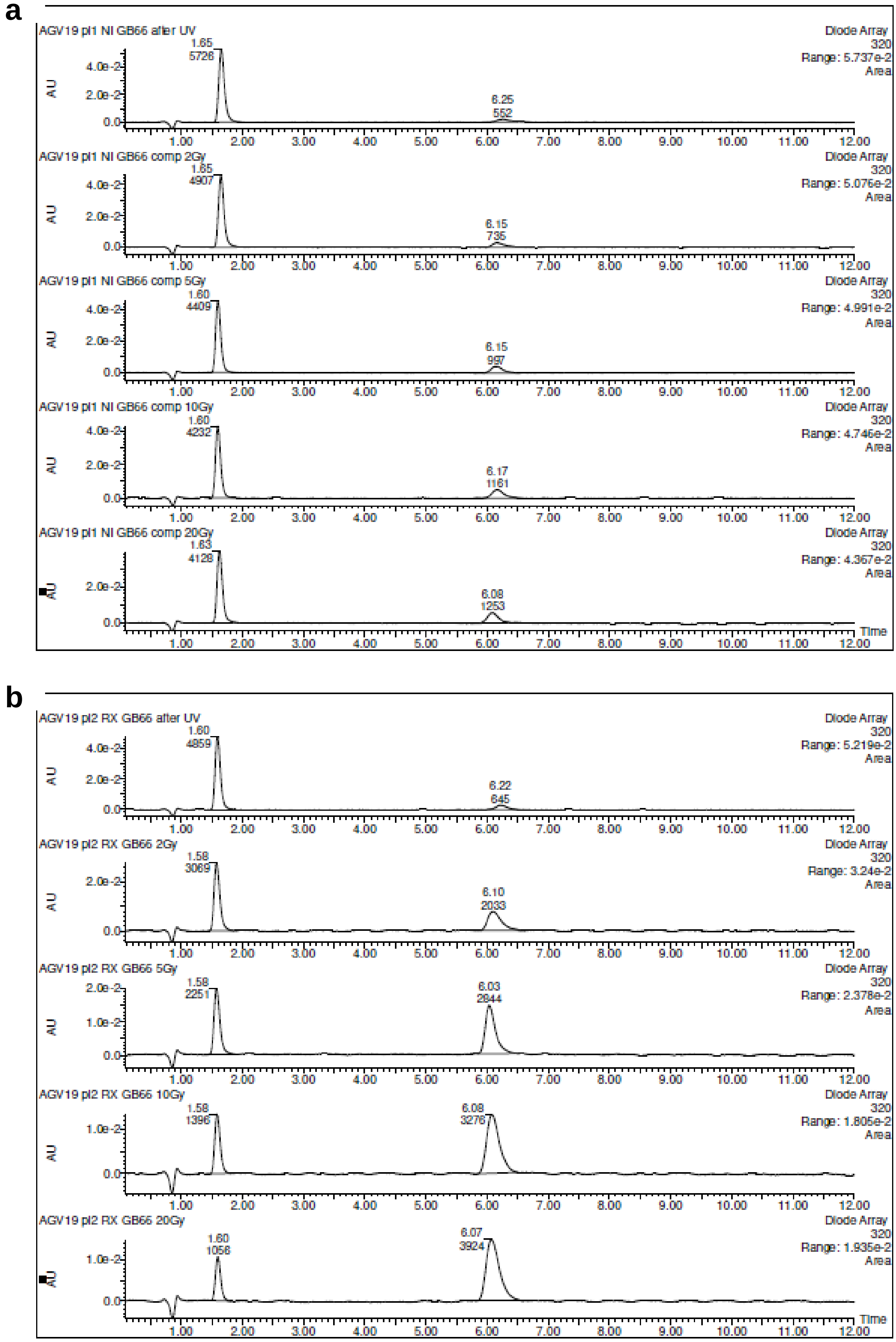

Fig. 21. HPLC chromatograms of *cis*-GdAzo upon X-ray irradiation. Exp. 1.

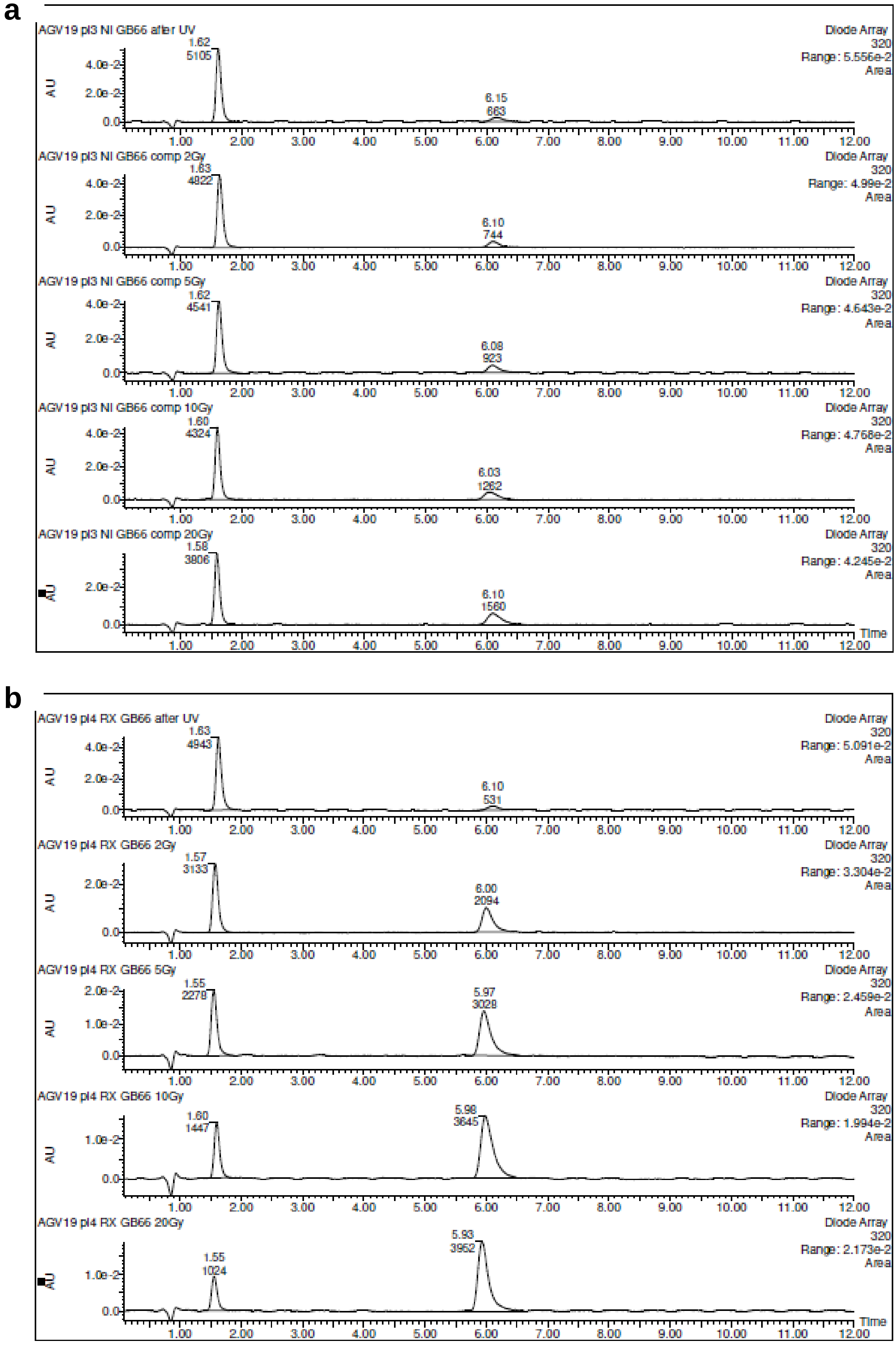

Fig. 22. HPLC chromatograms of *cis*-GdAzo upon X-ray irradiation. Exp. 2.

a

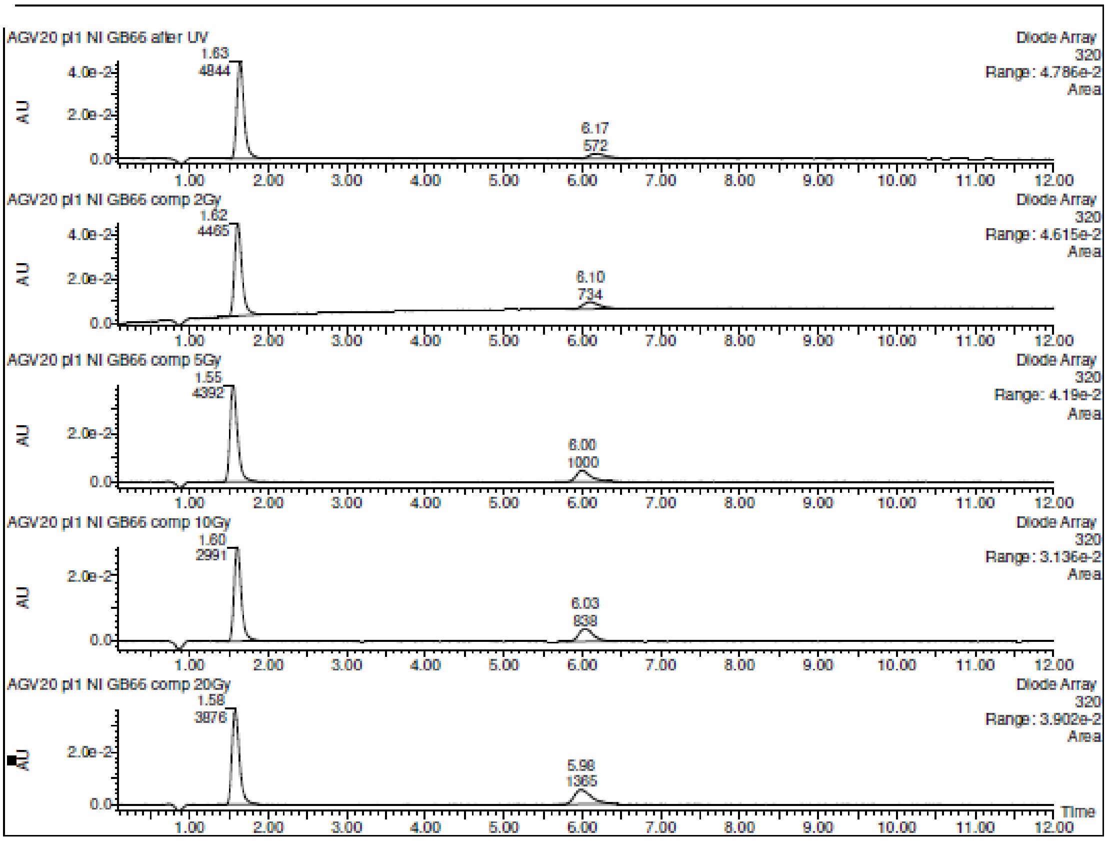

b

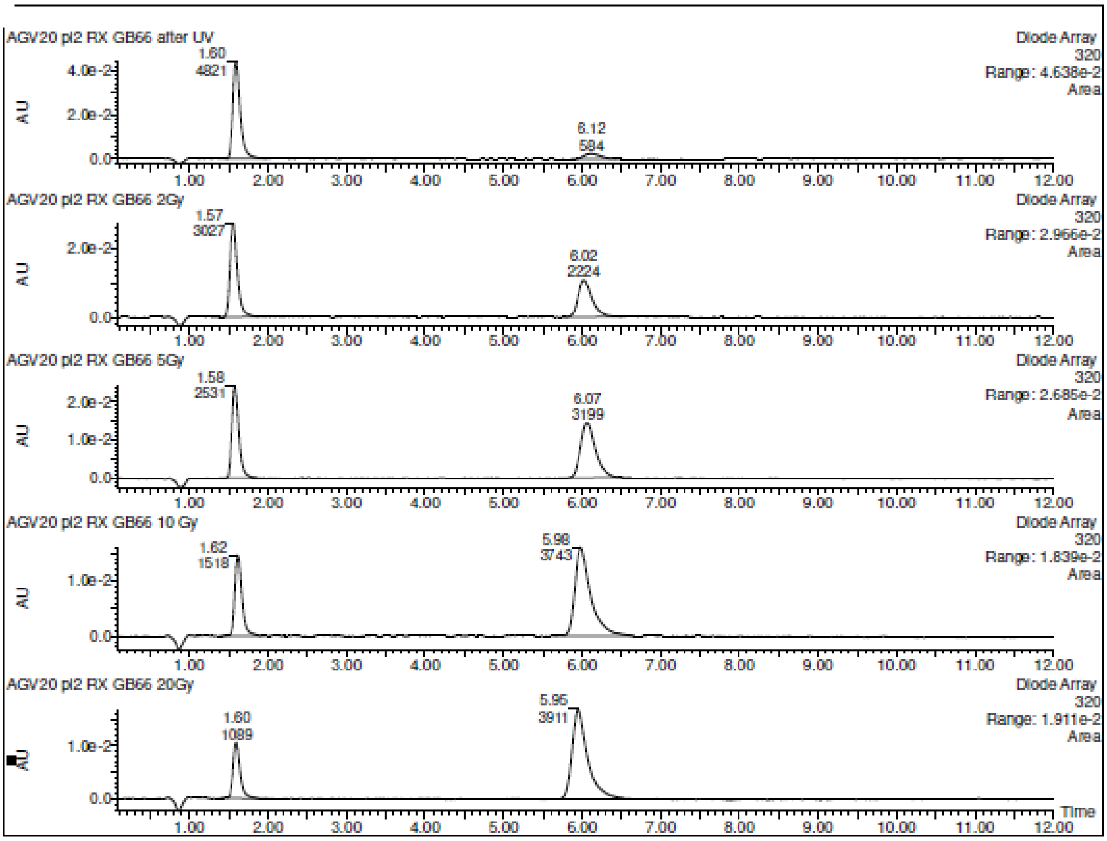

Fig. 23. HPLC chromatograms of *cis*-GdAzo upon X-ray irradiation. Exp. 3.

5.1.b.2 - Gamma-ray irradiation

Figs. 24-26. HPLC chromatograms of *cis*-Azo upon gamma-ray irradiation. Figs. 24, 25 and 26 are the repetitions of three independent experiments. **a**, HPLC chromatograms (319 nm, isobestic point, method C) of a solution of *cis*-**Azo** (50 µM in PBS, first chromatogram at the top) at the time of the corresponding experiments (**b**) upon gamma-ray irradiations (controls of 2, 3, 5 and 10 Gy irradiations from the second to the fifth chromatogram) (non-irradiated plate 1). **b**, HPLC chromatograms (319 nm, isobestic point, method C) of a solution of *cis*-**Azo** (50 µM in PBS, first chromatogram at the top) after successive gamma-ray irradiations of 2, 3, 5 and 10 Gy (from the second to the fifth chromatogram) (irradiated plate 2). The cumulative doses in the solutions are 0, 2, 5, 10 and 20 Gy from the top to the bottom chromatogram. The retention times of the *cis*-**Azo** and *trans*-**Azo** compounds are 2.02-2.08 min and 9.87-10.32 min respectively (areas of the peaks are assigned).

Figs. 27-29. HPLC chromatograms of *cis*-GdAzo upon gamma-ray irradiation. Figs. 27, 28 and 29 are the repetitions of three independent experiments. **a**, HPLC chromatograms (320 nm, isobestic point, method C) of a solution of *cis*-**GdAzo** (50 µM in PBS, first chromatogram at the top) at the time of the corresponding experiments (**b**) upon gamma-ray irradiations (controls of 2, 3, 5 and 10 Gy irradiations from the second to the fifth chromatogram) (non-irradiated plate 1). **b**, HPLC chromatograms (320 nm, isobestic point, method C) of a solution of *cis*-**GdAzo** (50 µM in PBS, first chromatogram at the top) after successive gamma-ray irradiations of 2, 3, 5 and 10 Gy (from the second to the fifth chromatogram) (irradiated plate 2). The cumulative doses in the solutions are 0, 2, 5, 10 and 20 Gy from the top to the bottom chromatogram. The retention times of the *cis*-**GdAzo** and *trans*-**GdAzo** compounds are 1.48-1.63 min and 5.60-5.97 min respectively (areas of the peaks are assigned).

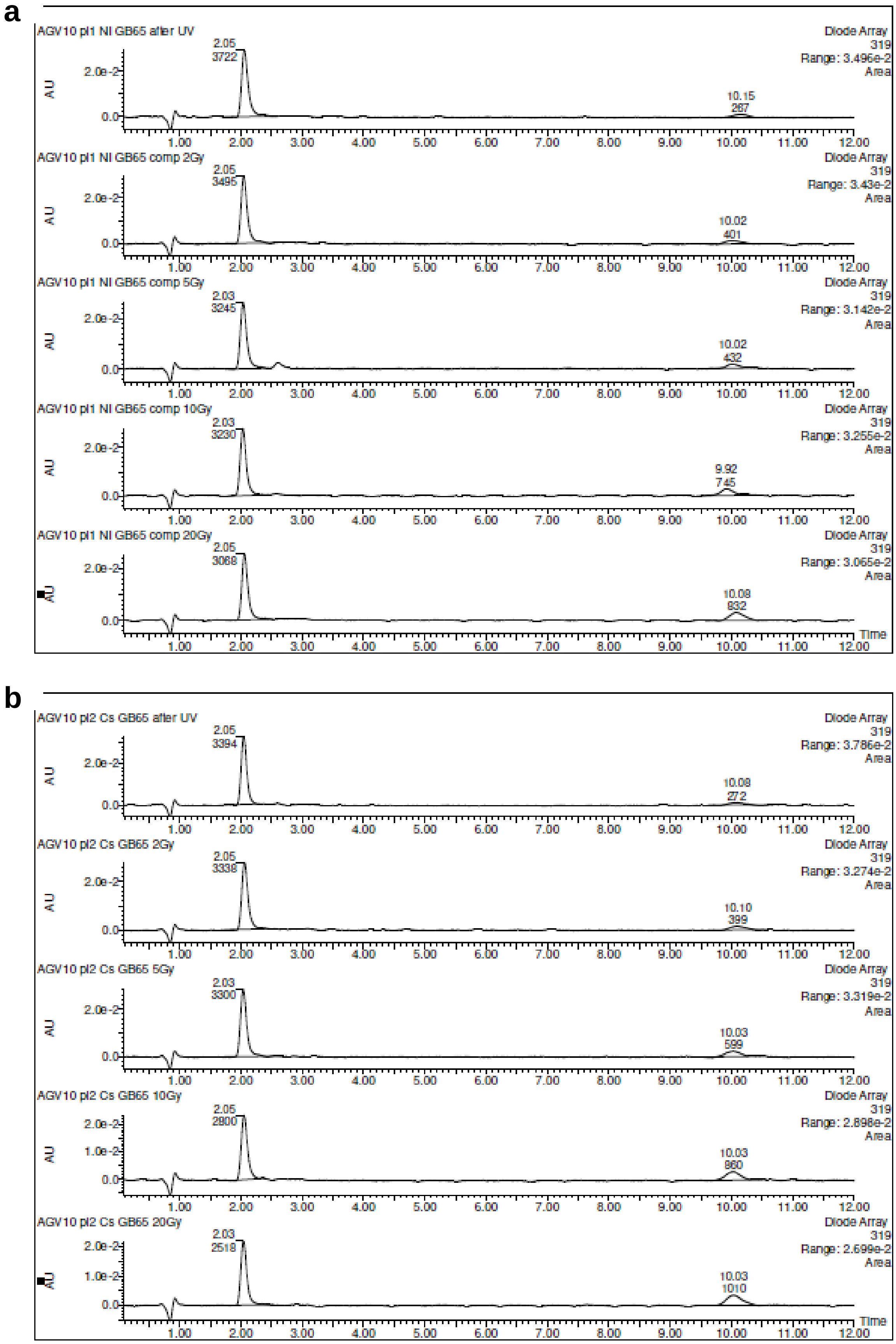

Fig. 24. HPLC chromatograms of *cis*-Azo upon gamma-ray irradiation. Exp. 1.

a

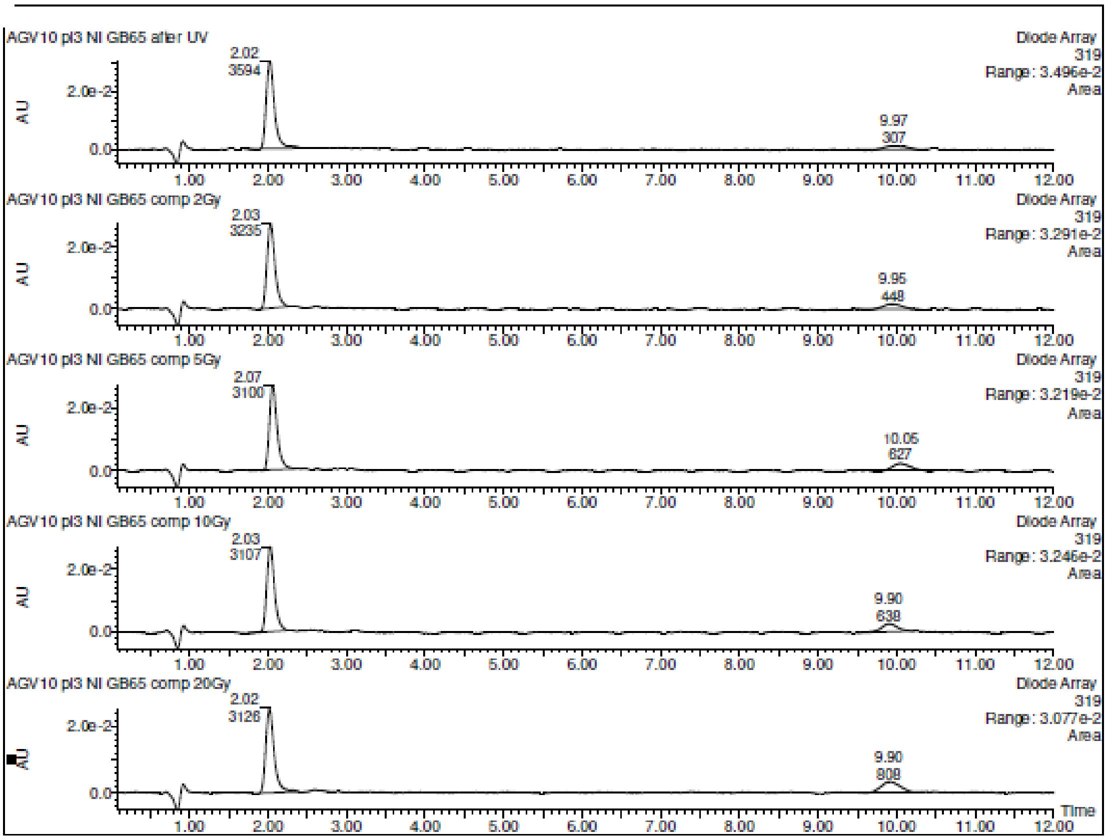

b

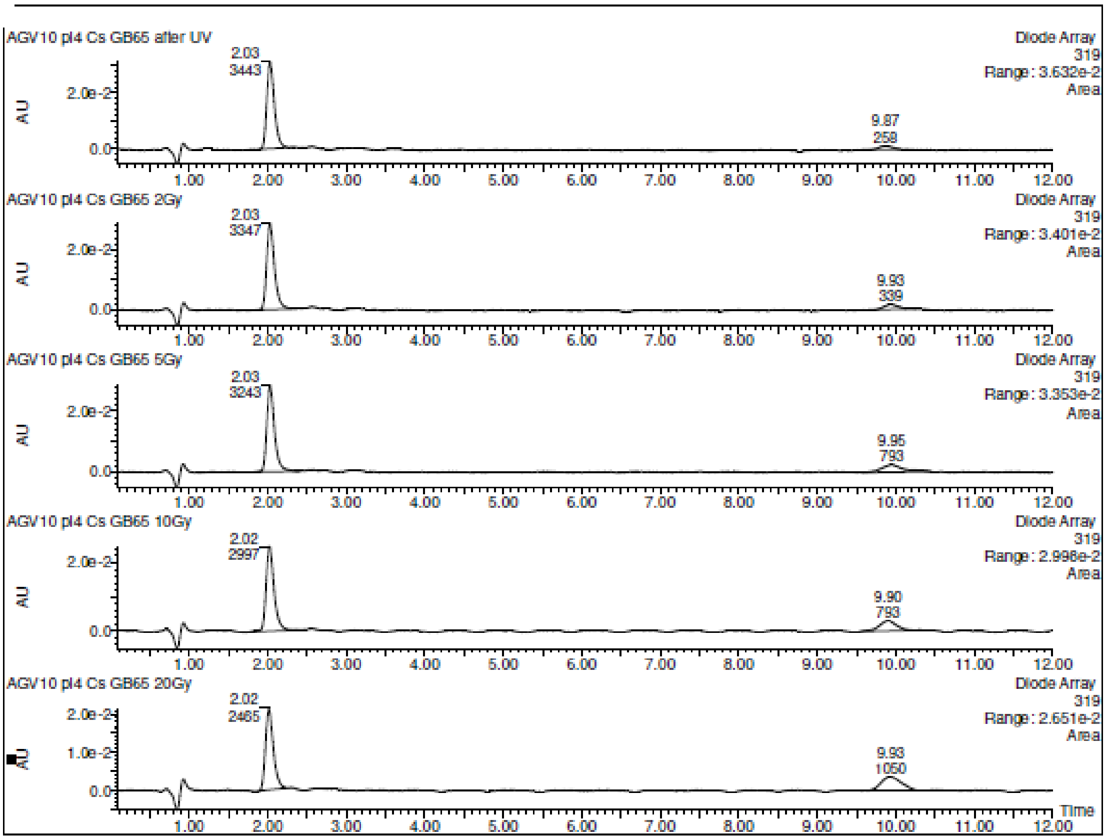

Fig. 25. HPLC chromatograms of *cis*-Azo upon gamma-ray irradiation. Exp. 2.

a

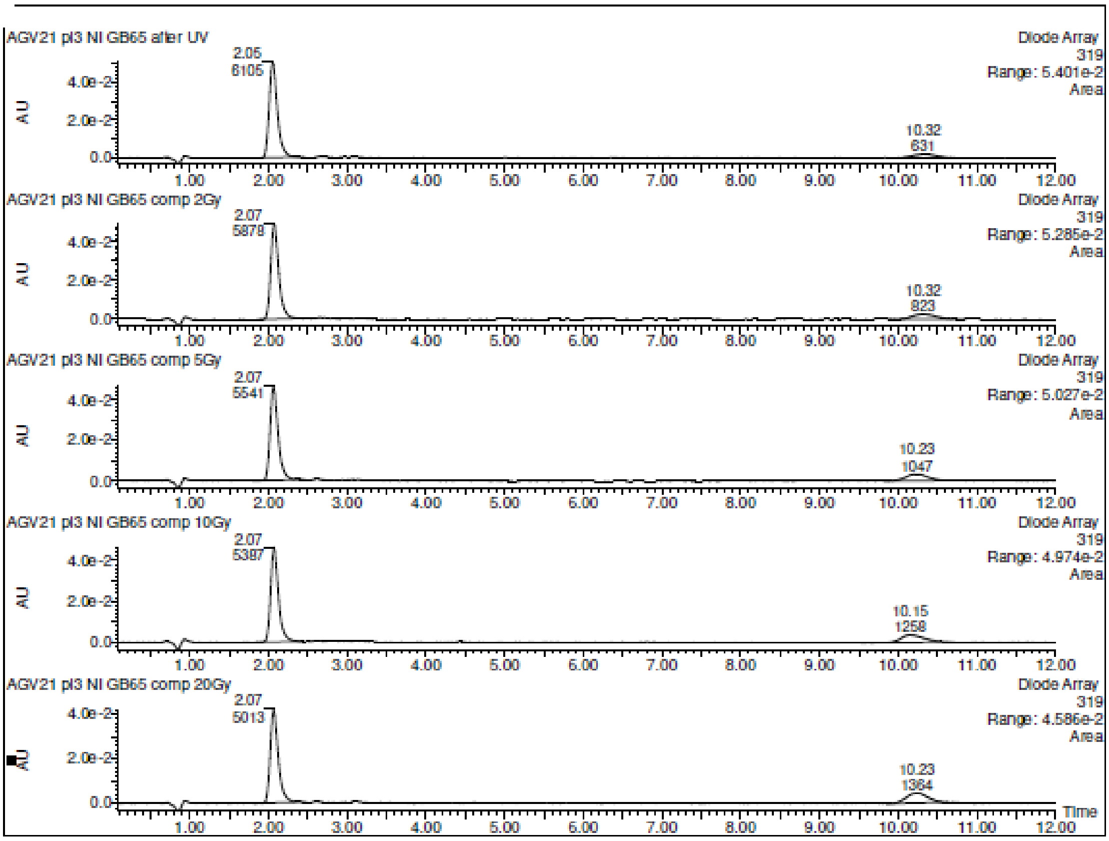

b

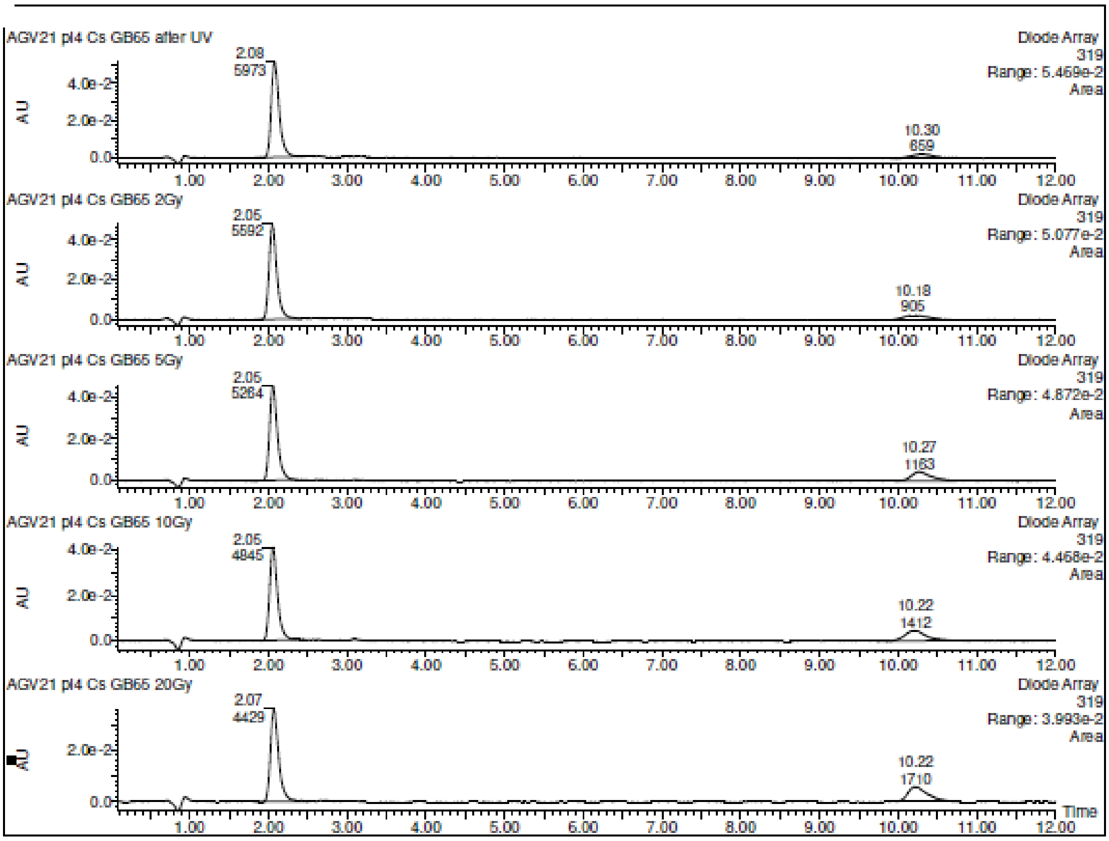

Fig. 26. HPLC chromatograms of *cis*-Azo upon gamma-ray irradiation. Exp. 3.

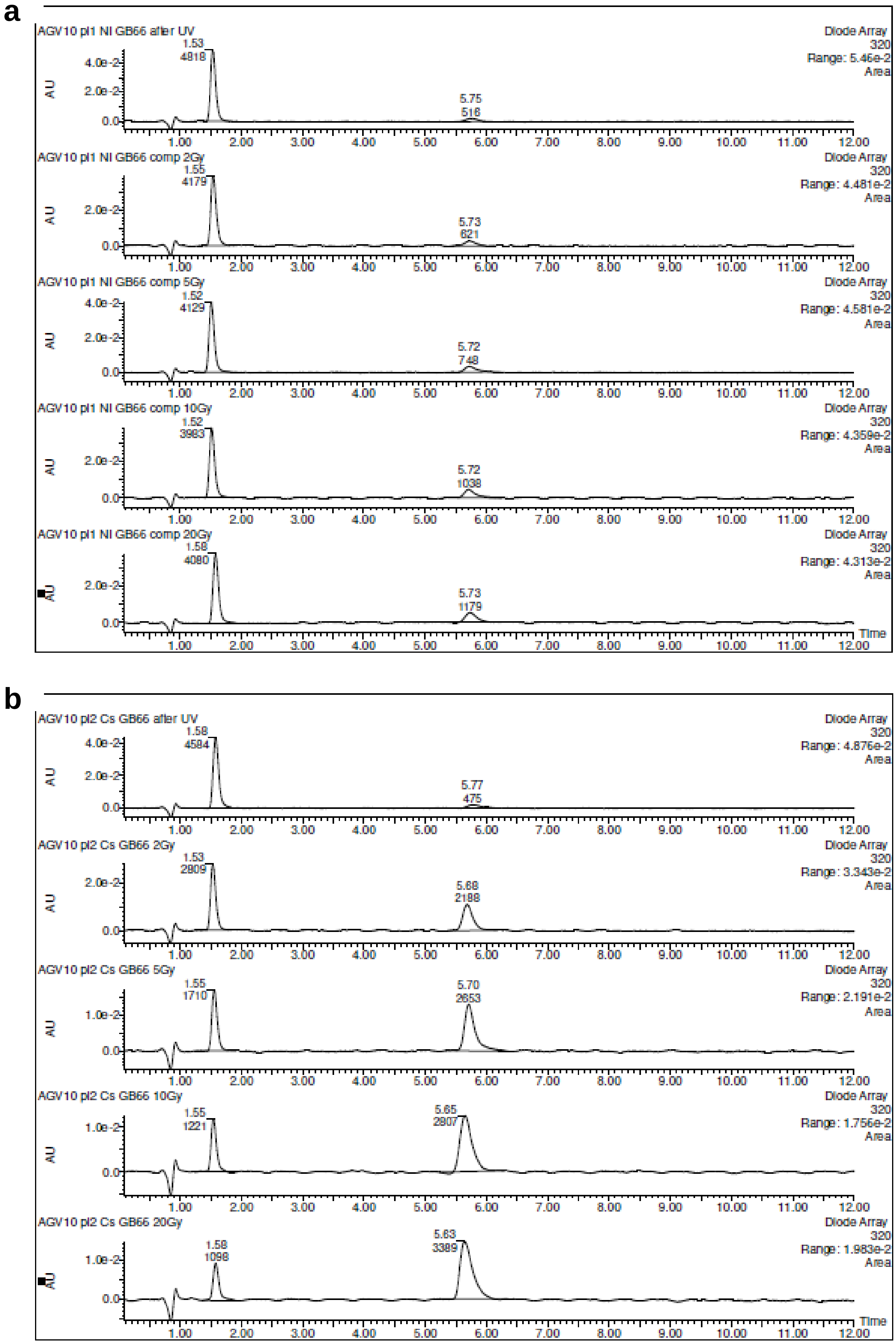

Fig. 27. HPLC chromatograms of *cis*-GdAzo upon gamma-ray irradiation. Exp. 1.

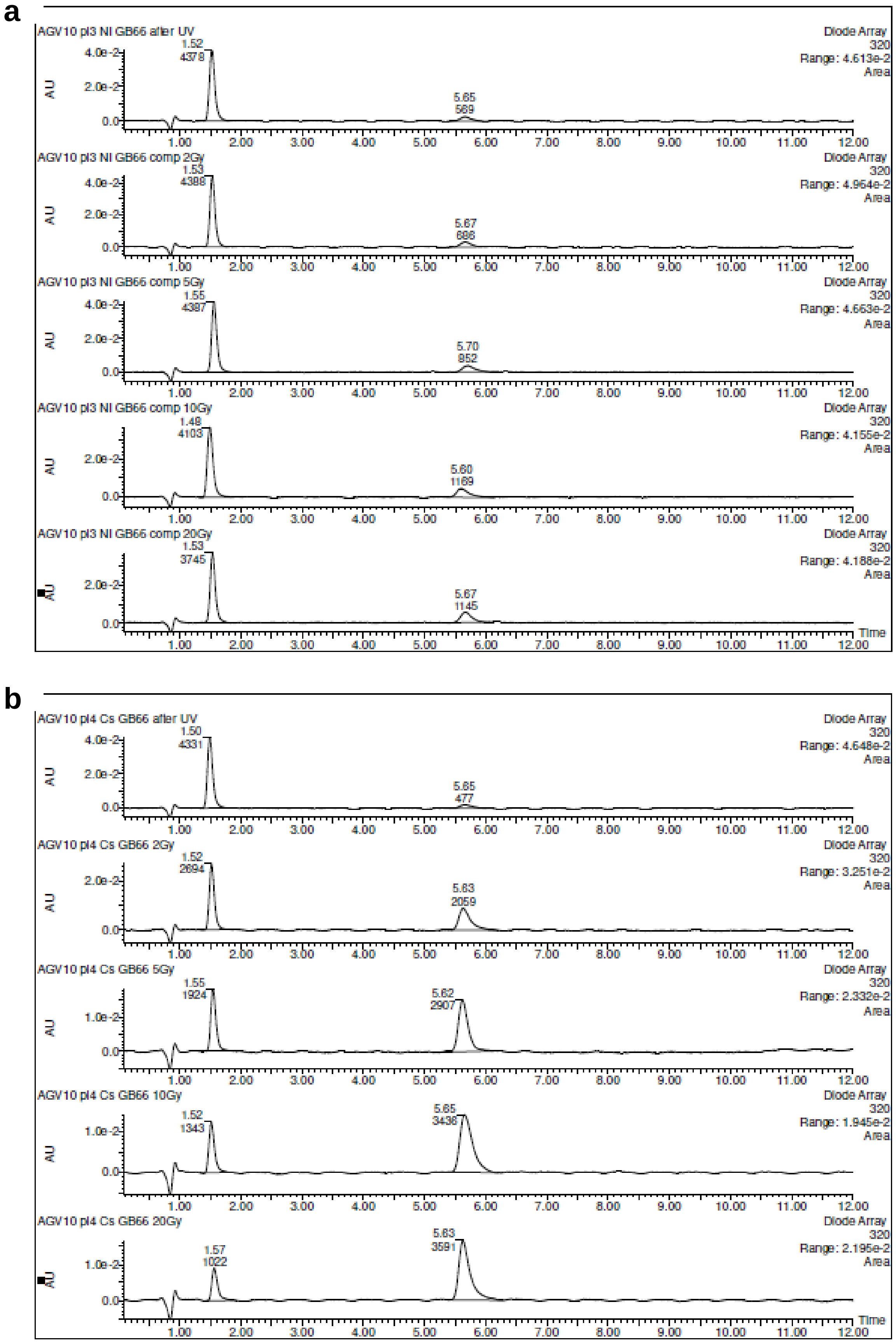

Fig. 28. HPLC chromatograms of *cis*-GdAzo upon gamma-ray irradiation. Exp. 2.

a

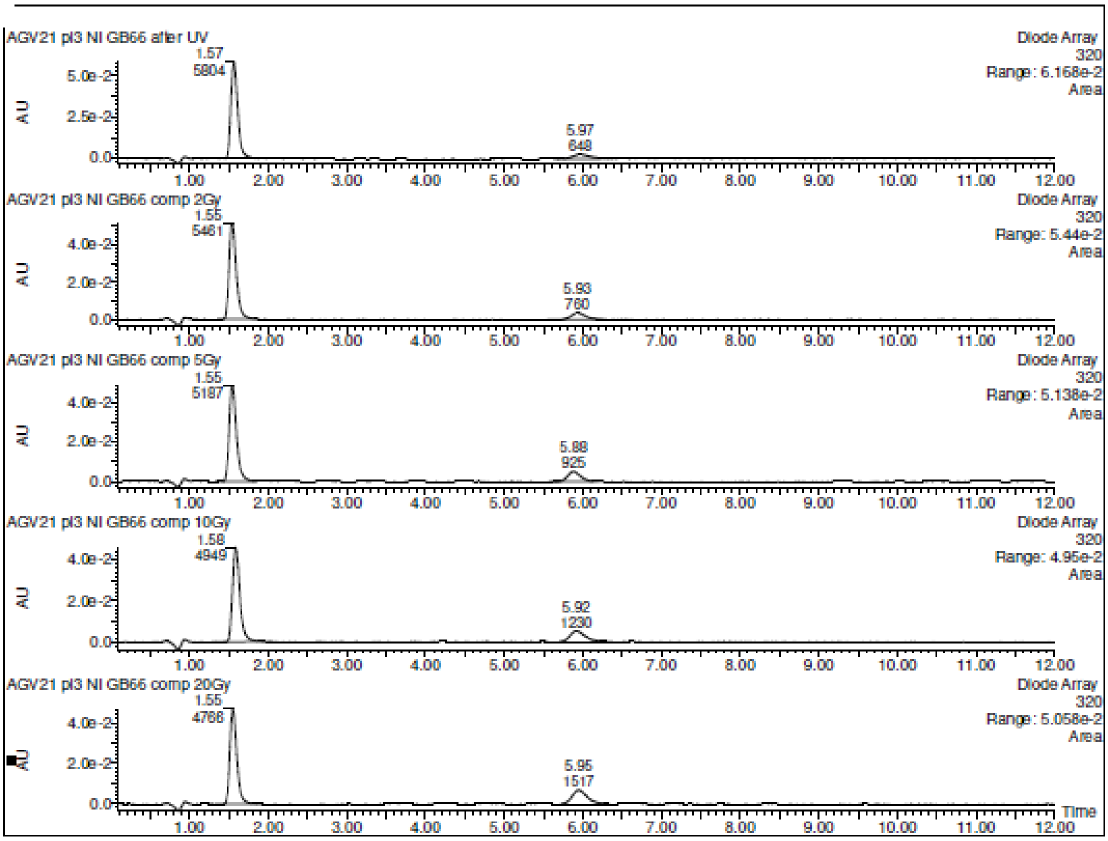

b

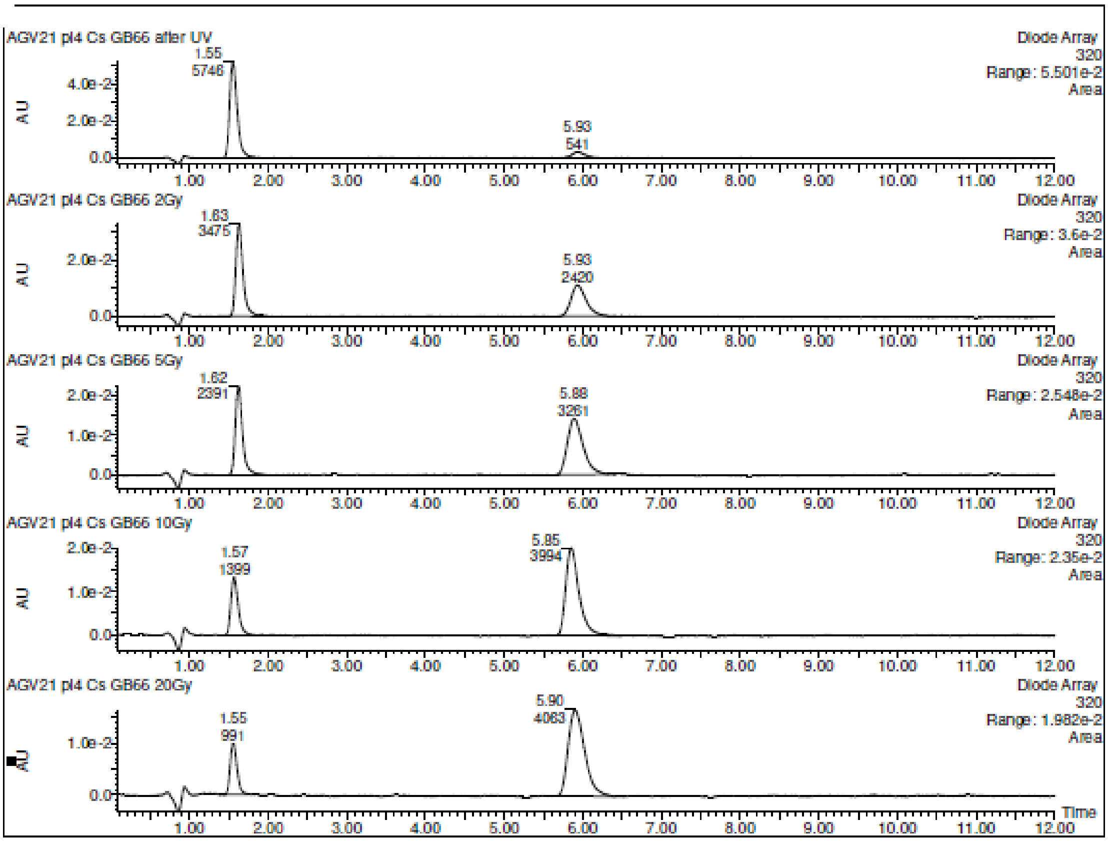

Fig. 29. HPLC chromatograms of *cis*-GdAzo upon gamma-ray irradiation. Exp. 3.

5.1.b.3 - Electron-beam irradiation

Figs. 30-32. HPLC chromatograms of *cis*-Azo upon electron-beam irradiation. Figs. 30, 31 and 32 are the repetitions of three independent experiments. **a**, HPLC chromatograms (319 nm, isobestic point, method C) of a solution of *cis*-**Azo** (50 µM in PBS, first chromatogram at the top) at the time of the corresponding experiments (**b**) upon electron-beam irradiations (controls of 2, 3, 5 and 10 Gy irradiations from the second to the fifth chromatogram) (non-irradiated plate 1). **b**, HPLC chromatograms (319 nm, isobestic point, method C) of a solution of *cis*-**Azo** (50 µM in PBS, first chromatogram at the top) after successive electron-beam irradiations of 2, 3, 5 and 10 Gy (from the second to the fifth chromatogram) (irradiated plate 2). The cumulative doses in the solutions are 0, 2, 5, 10 and 20 Gy from the top to the bottom chromatogram. The retention times of the *cis*-**Azo** and *trans*-**Azo** compounds are 2.08-2.18 min and 10.50-11.13 min respectively (areas of the peaks are assigned).

Figs. 33-35. HPLC chromatograms of *cis*-GdAzo upon electron-beam irradiation. Figs. 33, 34 and 35 are the repetitions of three independent experiments. **a**, HPLC chromatograms (320 nm, isobestic point, method C) of a solution of *cis*-**GdAzo** (50 µM in PBS, first chromatogram at the top) at the time of the corresponding experiments (**b**) upon electron-beam irradiations (controls of 2, 3, 5 and 10 Gy irradiations from the second to the fifth chromatogram) (non-irradiated plate 1). **b**, HPLC chromatograms (320 nm, isobestic point, method C) of a solution of *cis*-**GdAzo** (50 µM in PBS, first chromatogram at the top) after successive electron-beam irradiations of 2, 3, 5 and 10 Gy (from the second to the fifth chromatogram) (irradiated plate 2). The cumulative doses in the solutions are 0, 2, 5, 10 and 20 Gy from the top to the bottom chromatogram. The retention times of the *cis*-**GdAzo** and *trans*-**GdAzo** compounds are 1.55-1.63 min and 5.95-6.28 min respectively (areas of the peaks are assigned).

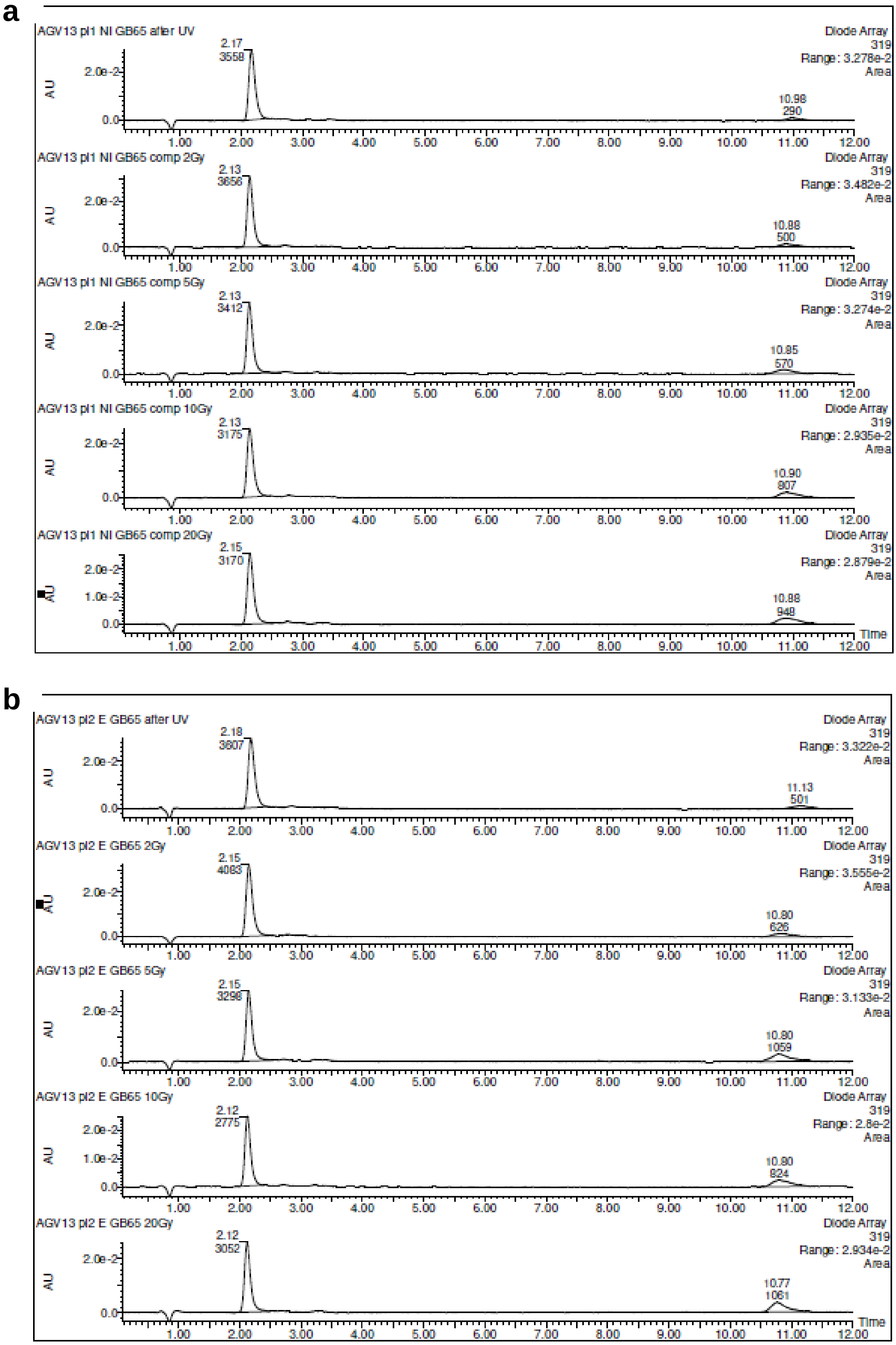

Fig. 30. HPLC chromatograms of *cis*-Azo upon electron-beam irradiation. Exp. 1.

a

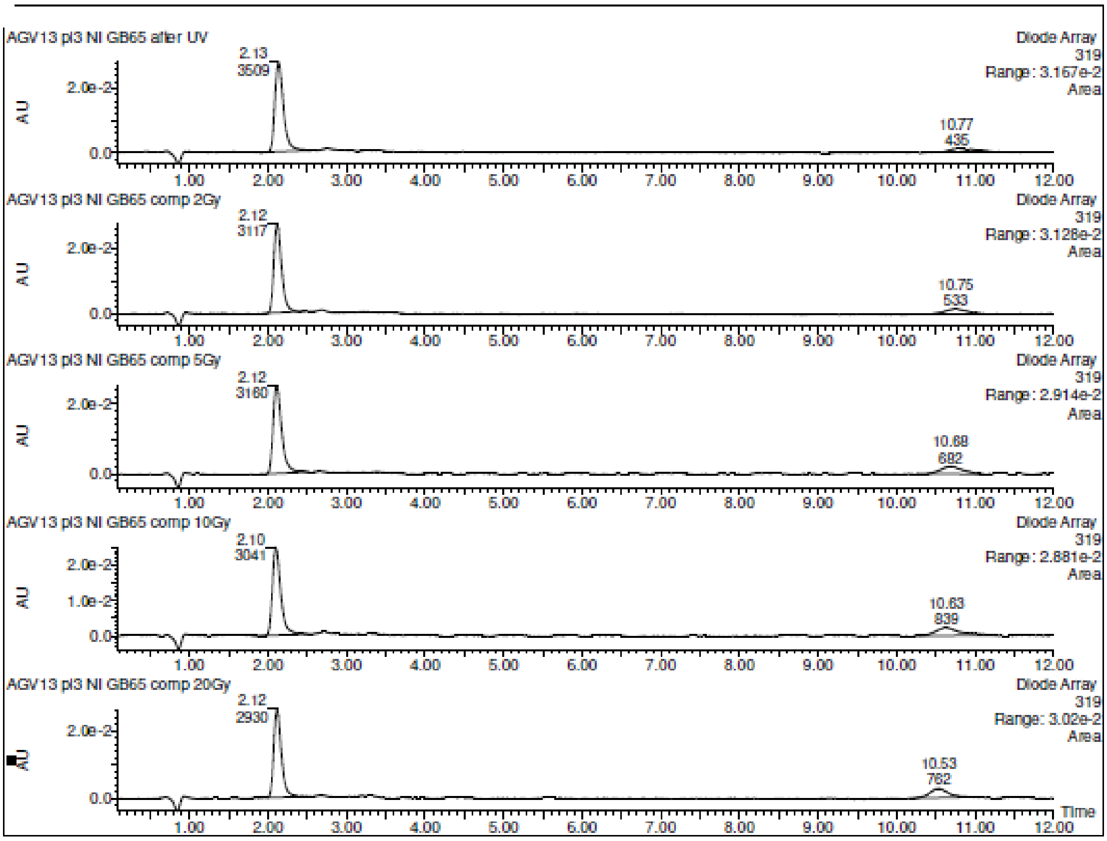

b

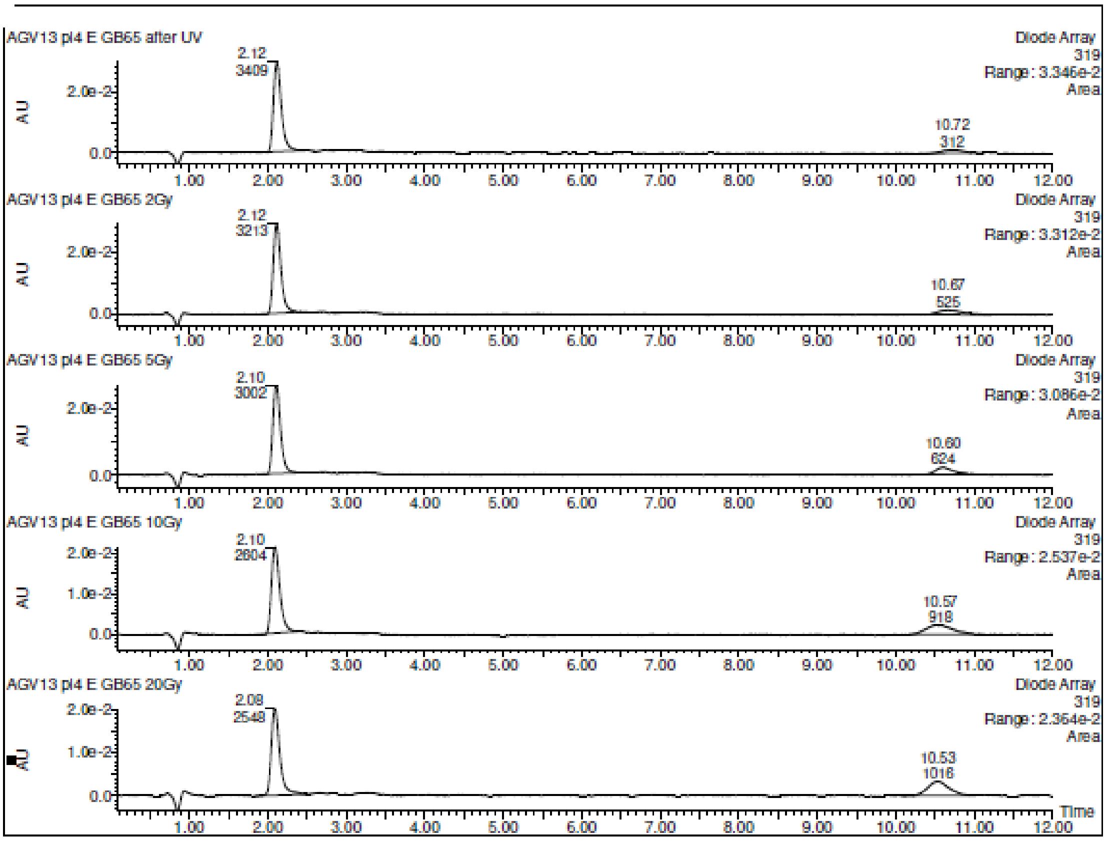

Fig. 31. HPLC chromatograms of *cis*-Azo upon electron-beam irradiation. Exp. 2.

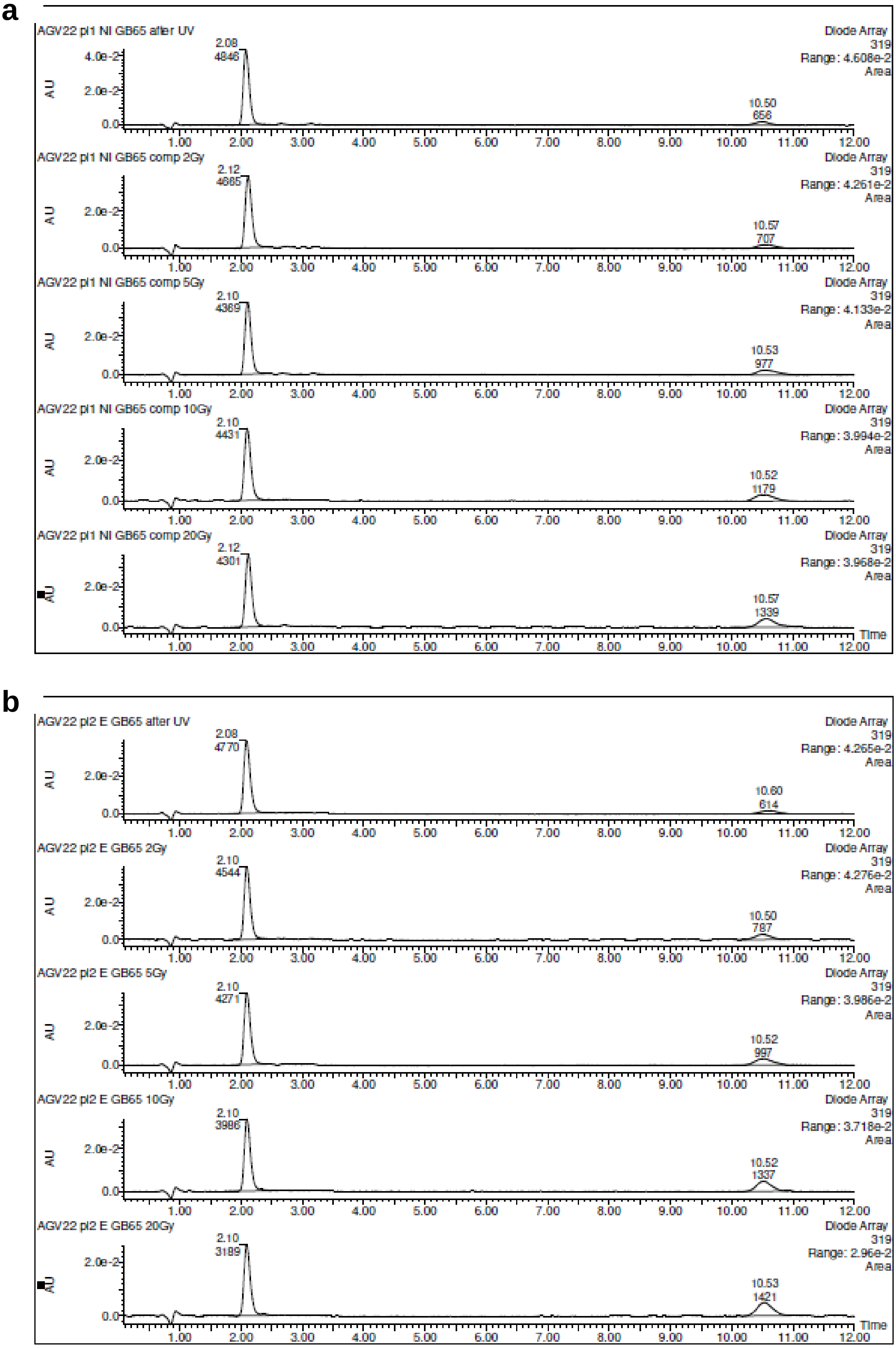

Fig. 32. HPLC chromatograms of *cis*-Azo upon electron-beam irradiation. Exp. 3.

a

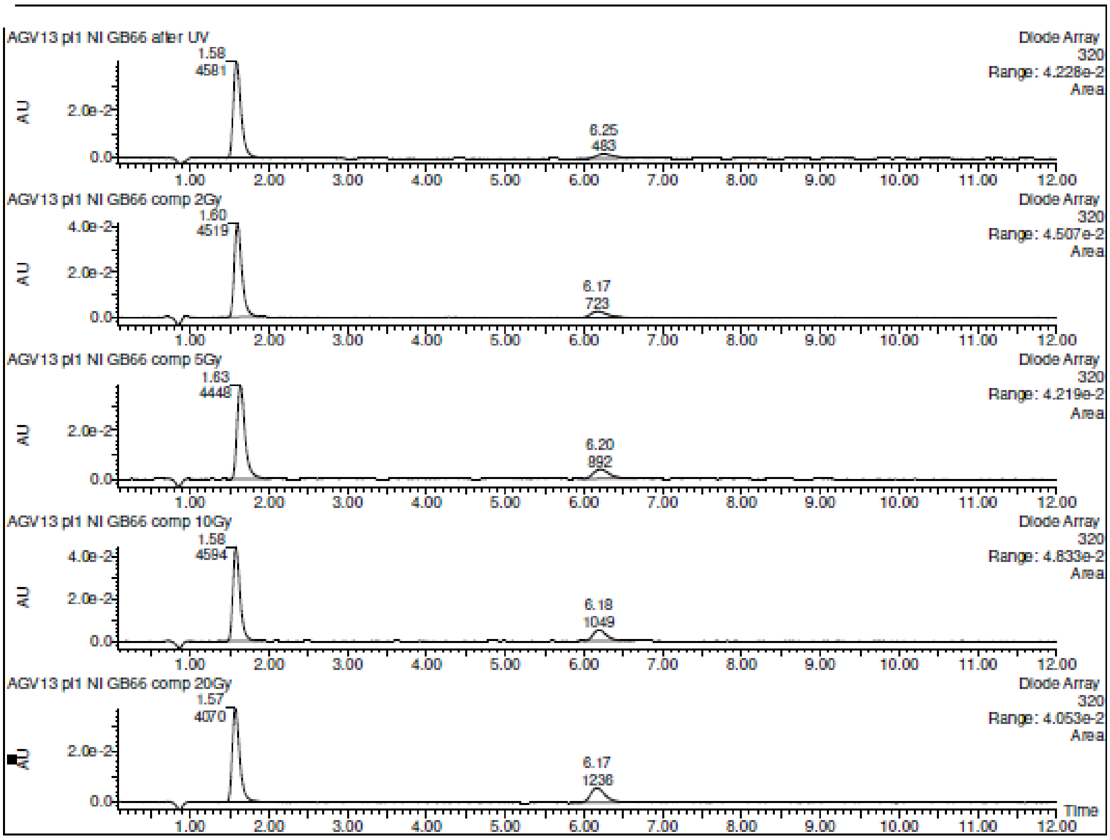

b

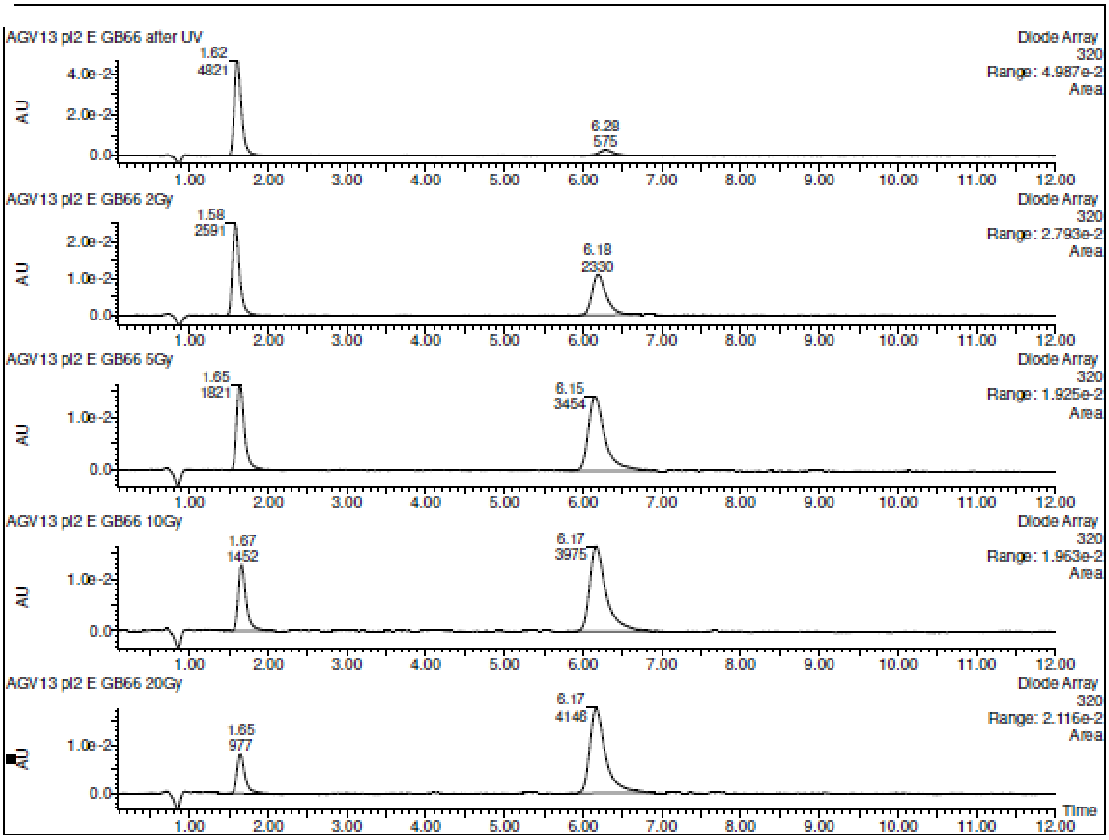

Fig. 33. HPLC chromatograms of *cis*-GdAzo upon electron-beam irradiation. Exp. 1.

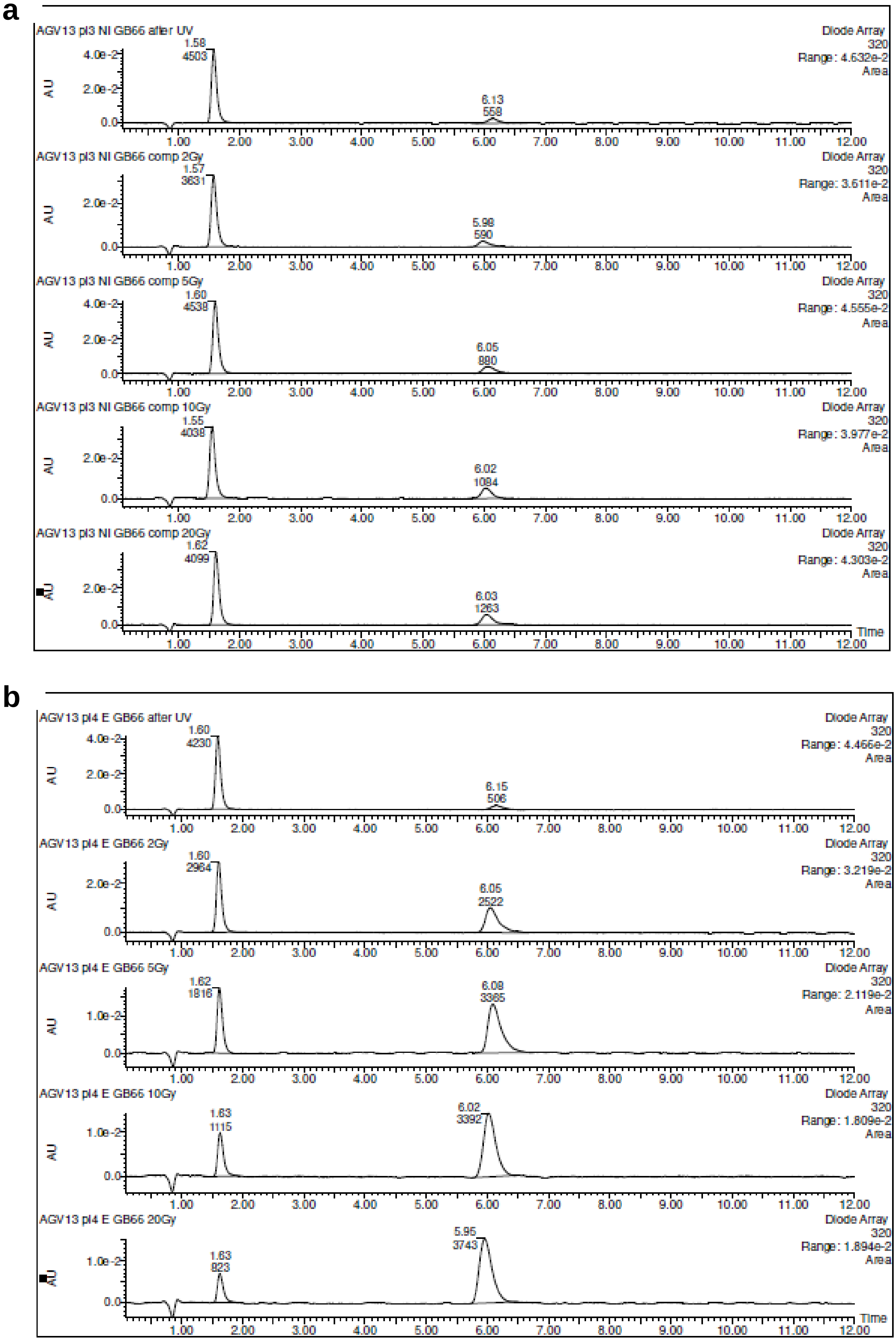

Fig. 34. HPLC chromatograms of *cis*-GdAzo upon electron-beam irradiation. Exp. 2.

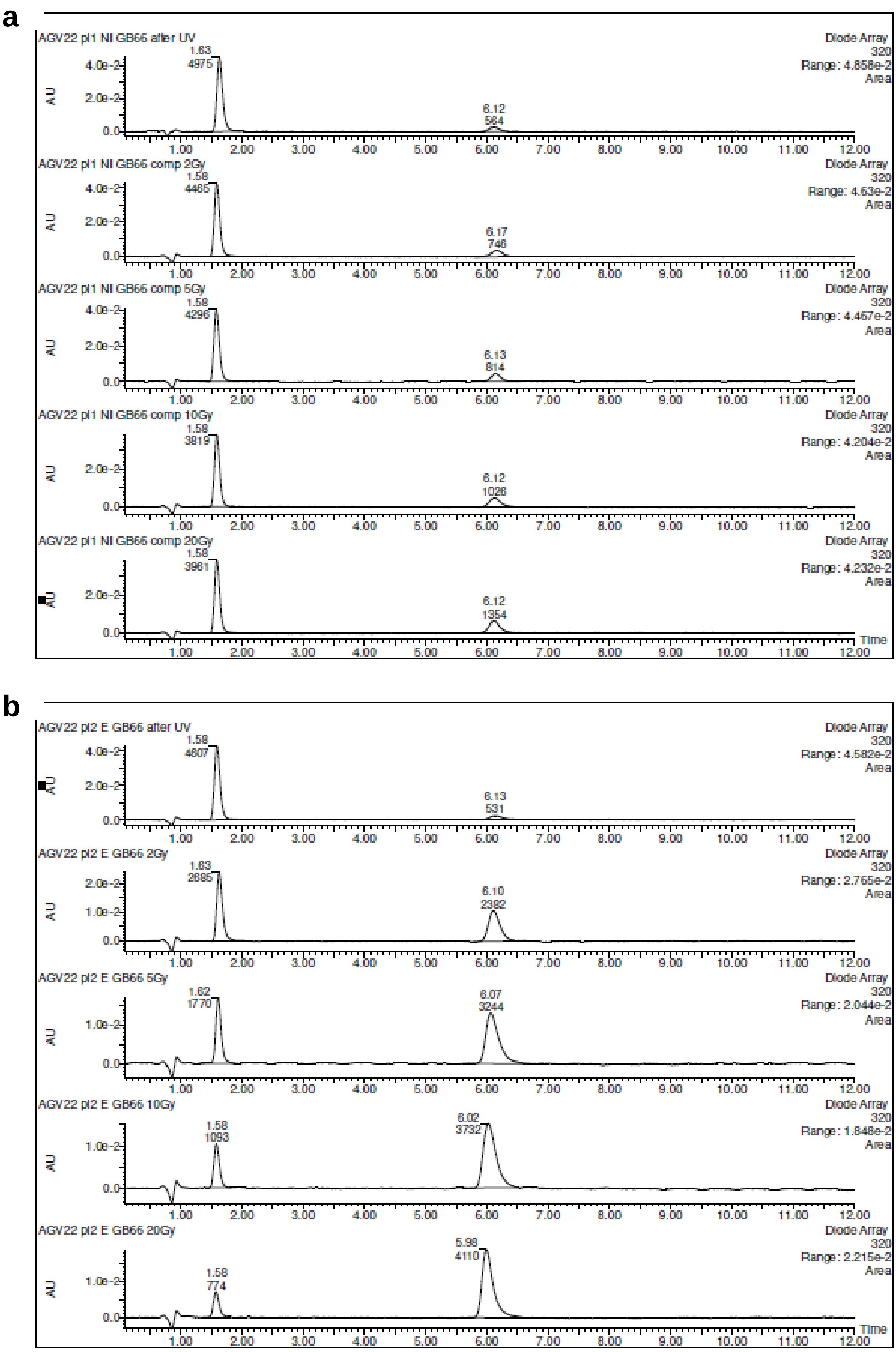

Fig. 35. HPLC chromatograms of *cis*-GdAzo upon electron-beam irradiation. Exp. 3.

a	No irradition (plate 1)						X-ray irradiation (plate 2)					
	GdAzo			Azo			GdAzo			Azo		
**Exp 1**	*trans* area	*cis* area	*trans* (%)	*trans* area	*cis* area	*trans* (%)	*trans* area	*cis* area	*trans* (%)	*trans* area	*cis* area	*trans* (%)
UV	552	5726	8,8	513	5074	9,2	645	4859	11,7	486	4994	8,9
2 Gy	735	4907	13,0	713	4825	12,9	2033	3069	39,8	878	4773	15,5
5Gy	997	4409	18,4	952	4804	16,5	2844	2251	55,8	1247	4402	22,1
10Gy	1161	4232	21,5	1064	4648	18,6	3276	1396	70,1	1353	4015	25,2
20Gy	1253	4128	23,3	1221	4405	21,7	3924	1056	78,8	1471	3785	28,0
**Exp 2**	*trans* area	*cis* area	*trans* (%)	*trans* area	*cis* area	*trans* (%)	*trans* area	*cis* area	*trans* (%)	*trans* area	*cis* area	*trans* (%)
UV	663	5105	11,5	499	4858	9,3	531	4943	9,7	668	5428	11,0
2 Gy	744	4822	13,4	812	4751	14,6	2094	3133	40,1	862	4549	15,9
5Gy	923	4541	16,9	816	3251	20,1	3028	2278	57,1	868	3128	21,7
10Gy	1262	4324	22,6	1238	4384	22,0	3645	1447	71,6	1371	4120	25,0
20Gy	1560	3806	29,1	1582	3952	28,6	3952	1024	79,4	1532	3465	30,7
**Exp 3**	*trans* area	*cis* area	*trans* (%)	*trans* area	*cis* area	*trans* (%)	*trans* area	*cis* area	*trans* (%)	*trans* area	*cis* area	*trans* (%)
UV	572	4844	10,6	662	5020	11,7	584	4821	10,8	625	4936	11,2
2 Gy	734	4465	14,1	777	4803	13,9	2224	3027	42,4	869	4581	15,9
5Gy	1000	4392	18,5	952	4574	17,2	3199	2531	55,8	995	4309	18,8
10Gy	838	2991	21,9	1111	4386	20,2	3743	1518	71,1	1345	3910	25,6
20Gy	1365	3876	26,0	1381	4066	25,4	3911	1089	78,2	1772	3372	34,4

b	No irradition (plate 1)						gamma-ray irradiation (plate 2)					
	GdAzo			Azo			GdAzo			Azo		
**Exp 1**	*trans* area	*cis* area	*trans* (%)	*trans* area	*cis* area	*trans* (%)	*trans* area	*cis* area	*trans* (%)	*trans* area	*cis* area	*trans* (%)
UV	516	4818	9,7	267	3722	6,7	475	4584	9,4	272	3394	7,4
2 Gy	621	4179	12,9	401	3495	10,3	2188	2809	43,8	399	3338	10,7
5Gy	748	4129	15,3	432	3245	11,7	2653	1710	60,8	599	3300	15,4
10Gy	1038	3983	20,7	745	3230	18,7	2807	1221	69,7	860	2800	23,5
20Gy	1179	4080	22,4	832	3068	21,3	3389	1098	75,5	1010	2518	28,6
**Exp 2**	*trans* area	*cis* area	*trans* (%)	*trans* area	*cis* area	*trans* (%)	*trans* area	*cis* area	*trans* (%)	*trans* area	*cis* area	*trans* (%)
UV	569	4378	11,5	307	3594	7,9	477	4331	9,9	258	3443	7,0
2 Gy	686	4388	13,5	448	3235	12,2	2059	2694	43,3	339	3347	9,2
5Gy	852	4387	16,3	627	3100	16,8	2907	1924	60,2	793	3243	19,6
10Gy	1169	4103	22,2	638	3107	17,0	3436	1343	71,9	793	2997	20,9
20Gy	1145	3745	23,4	808	3126	20,5	3591	1022	77,8	1050	2465	29,9
**Exp 3**	*trans* area	*cis* area	*trans* (%)	*trans* area	*cis* area	*trans* (%)	*trans* area	*cis* area	*trans* (%)	*trans* area	*cis* area	*trans* (%)
UV	648	5804	10,0	631	6105	9,4	541	5746	8,6	659	5973	9,9
2 Gy	760	5461	12,2	823	5878	12,3	2420	3475	41,1	905	5592	13,9
5Gy	925	5187	15,1	1047	5541	15,9	3261	2391	57,7	1163	5264	18,1
10Gy	1230	4949	19,9	1258	5387	18,9	3994	1399	74,1	1412	4845	22,6
20Gy	1517	4766	24,1	1364	5013	21,4	4063	991	80,4	1710	4429	27,9

c	No irradition (plate 1)						electron-beam irradiation (plate 2)					
	GdAzo			Azo			GdAzo			Azo		
**Exp 1**	*trans* area	*cis* area	*trans* (%)	*trans* area	*cis* area	*trans* (%)	*trans* area	*cis* area	*trans* (%)	*trans* area	*cis* area	*trans* (%)
UV	483	4581	9,5	290	3558	7,5	575	4821	10,7	501	3607	12,2
2 Gy	723	4519	13,8	500	3656	12,0	2330	2591	47,3	626	4083	13,3
5Gy	892	4448	16,7	570	3412	14,3	3454	1821	65,5	1059	3298	24,3
10Gy	1049	4594	18,6	807	3175	20,3	3975	1452	73,2	824	2775	22,9
20Gy	1236	4070	23,3	948	3170	23,0	4146	977	80,9	1061	3052	25,8
**Exp 2**	*trans* area	*cis* area	*trans* (%)	*trans* area	*cis* area	*trans* (%)	*trans* area	*cis* area	*trans* (%)	*trans* area	*cis* area	*trans* (%)
UV	558	4503	11,0	435	3509	11,0	506	4230	10,7	312	3409	8,4
2 Gy	590	3631	14,0	533	3117	14,6	2522	2964	46,0	525	3213	14,0
5Gy	880	4538	16,2	682	3160	17,8	3365	1816	64,9	624	3002	17,2
10Gy	1084	4038	21,2	839	3041	21,6	3392	1115	75,3	918	2604	26,1
20Gy	1263	4099	23,6	762	2930	20,6	3743	823	82,0	1016	2548	28,5
**Exp 3**	*trans* area	*cis* area	*trans* (%)	*trans* area	*cis* area	*trans* (%)	*trans* area	*cis* area	*trans* (%)	*trans* area	*cis* area	*trans* (%)
UV	564	4975	10,2	656	4846	11,9	531	4607	10,3	614	4770	11,4
2 Gy	746	4465	14,3	707	4665	13,2	2382	2685	47,0	787	4544	14,8
5Gy	814	4296	15,9	977	4369	18,3	3244	1770	64,7	997	4271	18,9
10Gy	1026	3819	21,2	1179	4431	21,0	3732	1093	77,3	1337	3986	25,1
20Gy	1354	3961	25,5	1339	4301	23,7	4110	774	84,2	1421	3189	30,8

Table 1. Proportion of *trans*-Azo and *trans*-GdAzo upon ionising radiations determined by HPLC. Assessment of *cis*-**Azo** and *cis*-**GdAzo** isomerisation upon X-ray (**a**), gamma-ray (**b**) and electron-beam (**c**) irradiations. The data from the experiments without ionising radiation (control experiment, plate 1) and from the experiments upon ionising radiations (plate 2) are reported on the left and on the right respectively. Three independent experiments are reported (Exp 1, 2 and 3). The peak areas of both isomers were measured by HPLC (method C) after UV and ionising radiations. The proportions of the *trans*-isomer were determined by the area ratios.

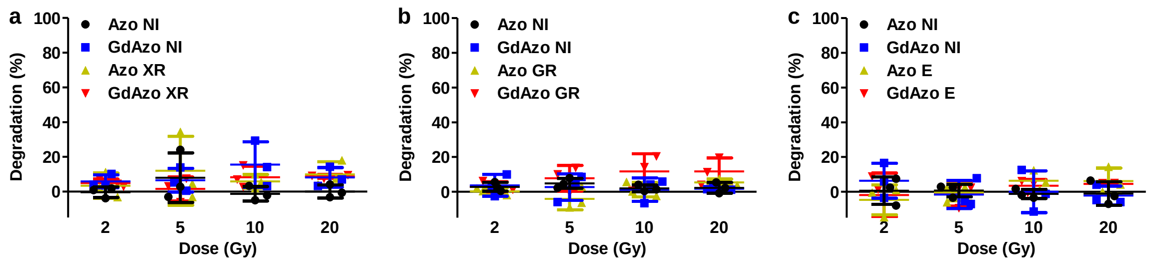

Fig. 36. Degradation of *cis*-Azo and *cis*-GdAzo upon ionising radiations. Assessment of *cis*-**Azo** and *cis*-**GdAzo** degradation upon X-ray (**a**), gamma-ray (**b**) and electron-beam (**c**) irradiations determined by HPLC (method C) (3 independent experiments). The degradation was determined from the formulae: (signal area after UV irradiation - signal area after ionising radiation)/(signal area after UV irradiation)x100 with the signal area corresponding to the addition of the *cis* and *trans*-isomer signal areas. A positive value represents a loss of compound. The data are represented as the means ± standard deviations. A random variation of the degradation values was observed with some fluctuations in the same range for compounds irradiated or non-irradiated by ionising radiation. No compound degradation due to ionising radiation could be observed in our experimental conditions.

5.2 - Characterisation by LC-MS

The liquid chromatography-mass spectrometry (LC-MS) method is described in the Supplementary Section 1 (method E). The compounds **Azo** and **GdAzo** were analysed before (Figs. 37-38) and after (Figs. 39-44) 20 Gy ionising radiations (X-ray, gamma-ray and electron beam).

5.2.a - Before irradiation

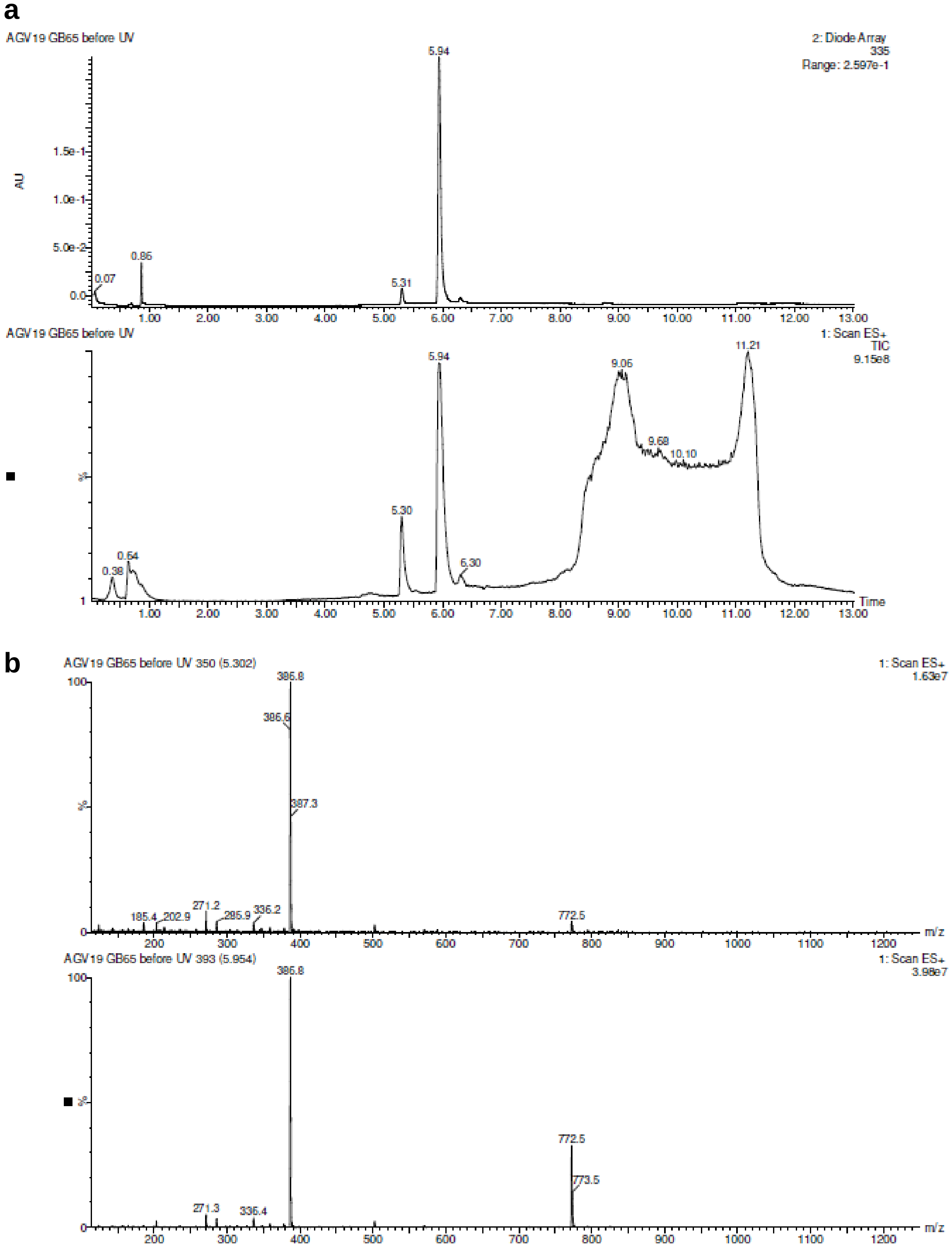

Fig. 37. LC-MS of Azo before irradiation. a, Absorbance (335 nm) and total ion current (positive mode) chromatograms from top to bottom. **b**, Mass spectra (positive mode) of *cis*-**Azo** (top, t_R = 5.302 min) and *trans*-**Azo** (bottom, t_R = 5.954 min).

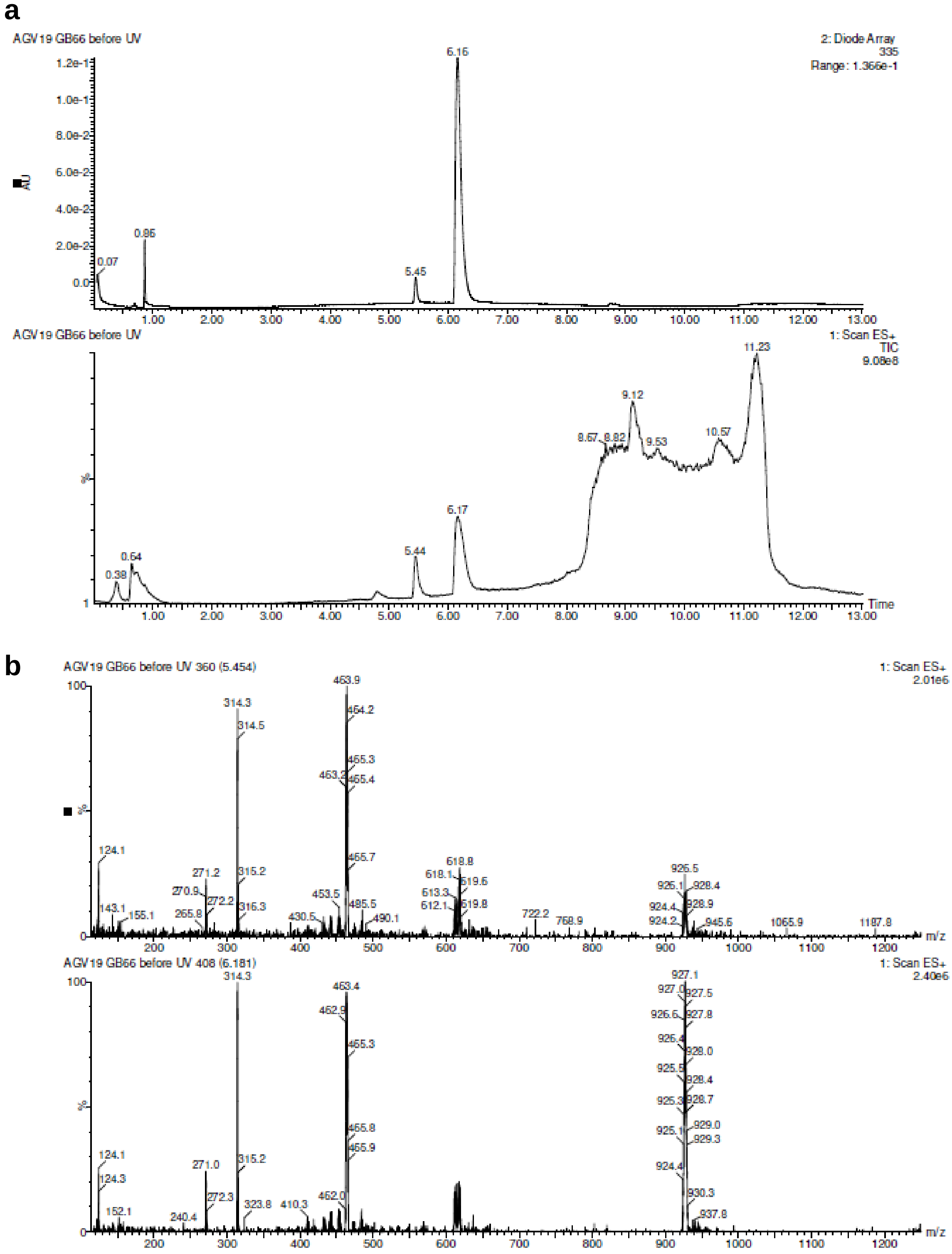

Fig. 38. LC-MS of GdAzo before irradiation. a, Absorbance (335 nm) and total ion current (positive mode) chromatograms from top to bottom. **b**, Mass spectra (positive mode) of *cis*-**GdAzo** (top, t_R = 5.454 min) and *trans*-**GdAzo** (bottom, t_R = 6.181 min).

5.2.b - After 20 Gy X-ray irradiation

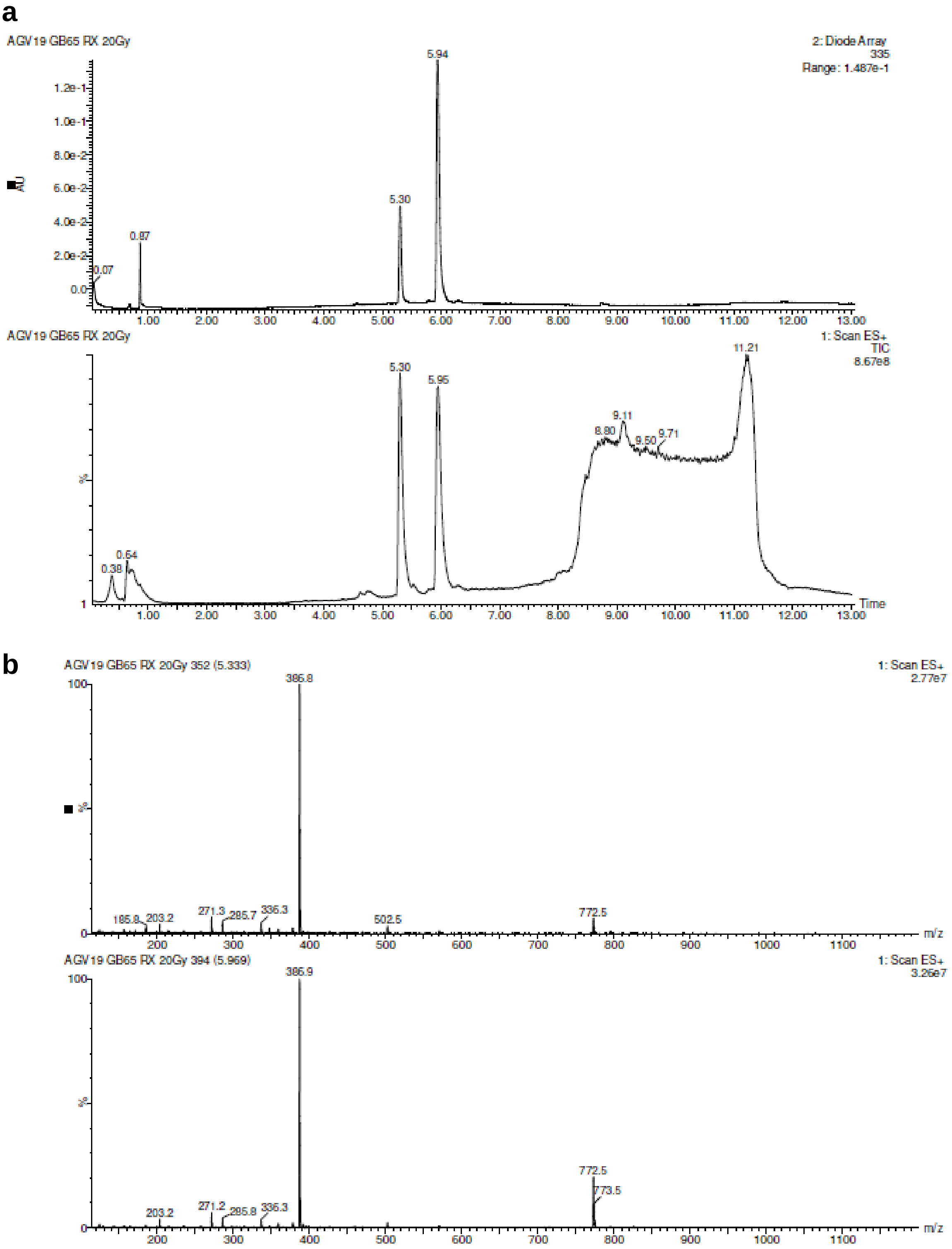

Fig. 39. LC-MS of Azo after 20 Gy X-ray irradiation. a, Absorbance (335 nm) and total ion current (positive mode) chromatograms from top to bottom. **b**, Mass spectra (positive mode) of *cis*-**Azo** (top, t_R = 5.333 min) and *trans*-**Azo** (bottom, t_R = 5.969 min).

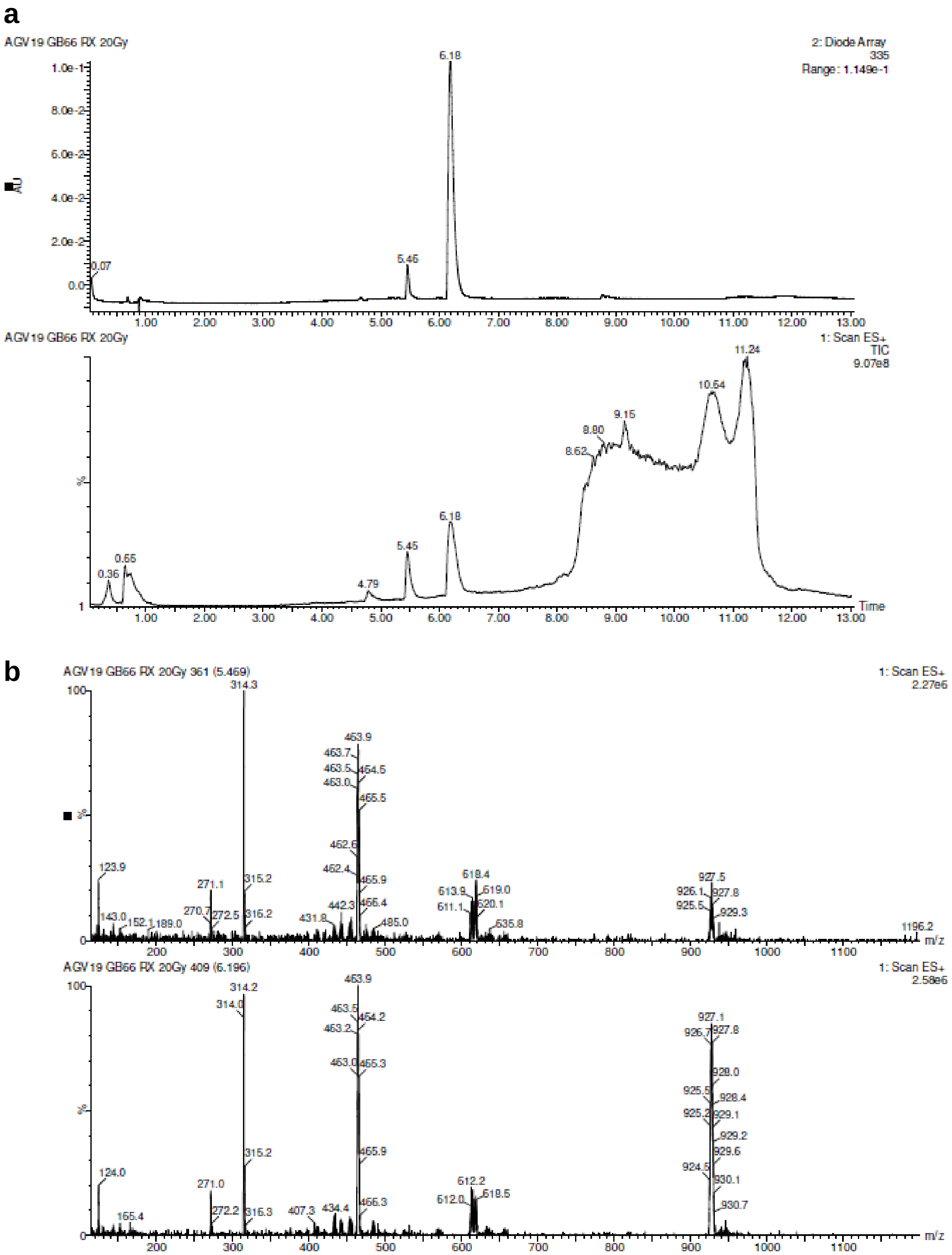

Fig. 40. LC-MS of GdAzo after 20 Gy X-ray irradiation. a, Absorbance (335 nm) and total ion current (positive mode) chromatograms from top to bottom. **b**, Mass spectra (positive mode) of *cis*-**GdAzo** (top, t_R = 5.469 min) and *trans*-**GdAzo** (bottom, t_R = 6.196 min).

5.2.c - After 20 Gy gamma-ray irradiation

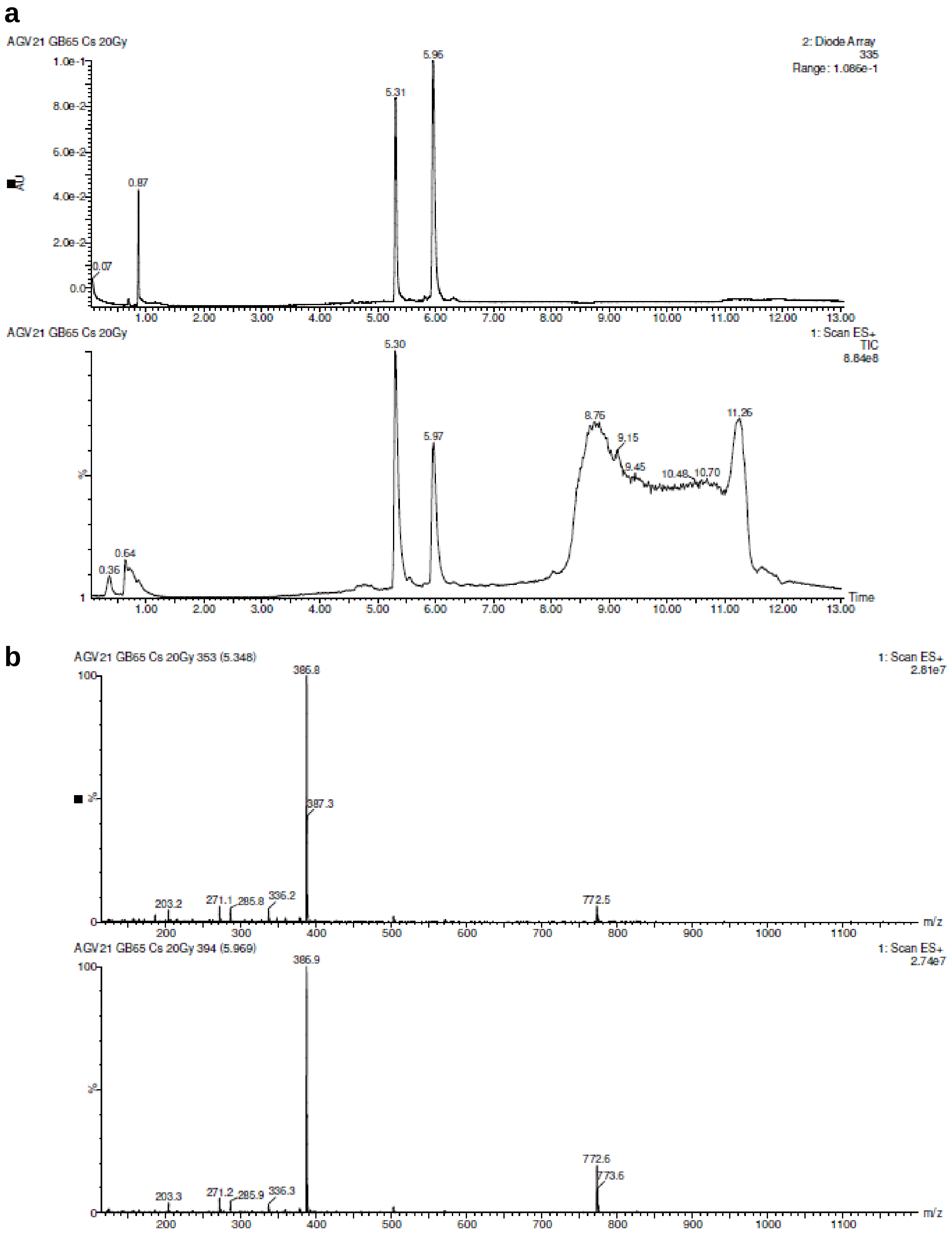

Fig. 41. LC-MS of Azo after 20 Gy gamma-ray irradiation. a, Absorbance (335 nm) and total ion current (positive mode) chromatograms from top to bottom. **b**, Mass spectra (positive mode) of *cis*-**Azo** (top, t_R = 5.348 min) and *trans*-**Azo** (bottom, t_R = 5.969 min).

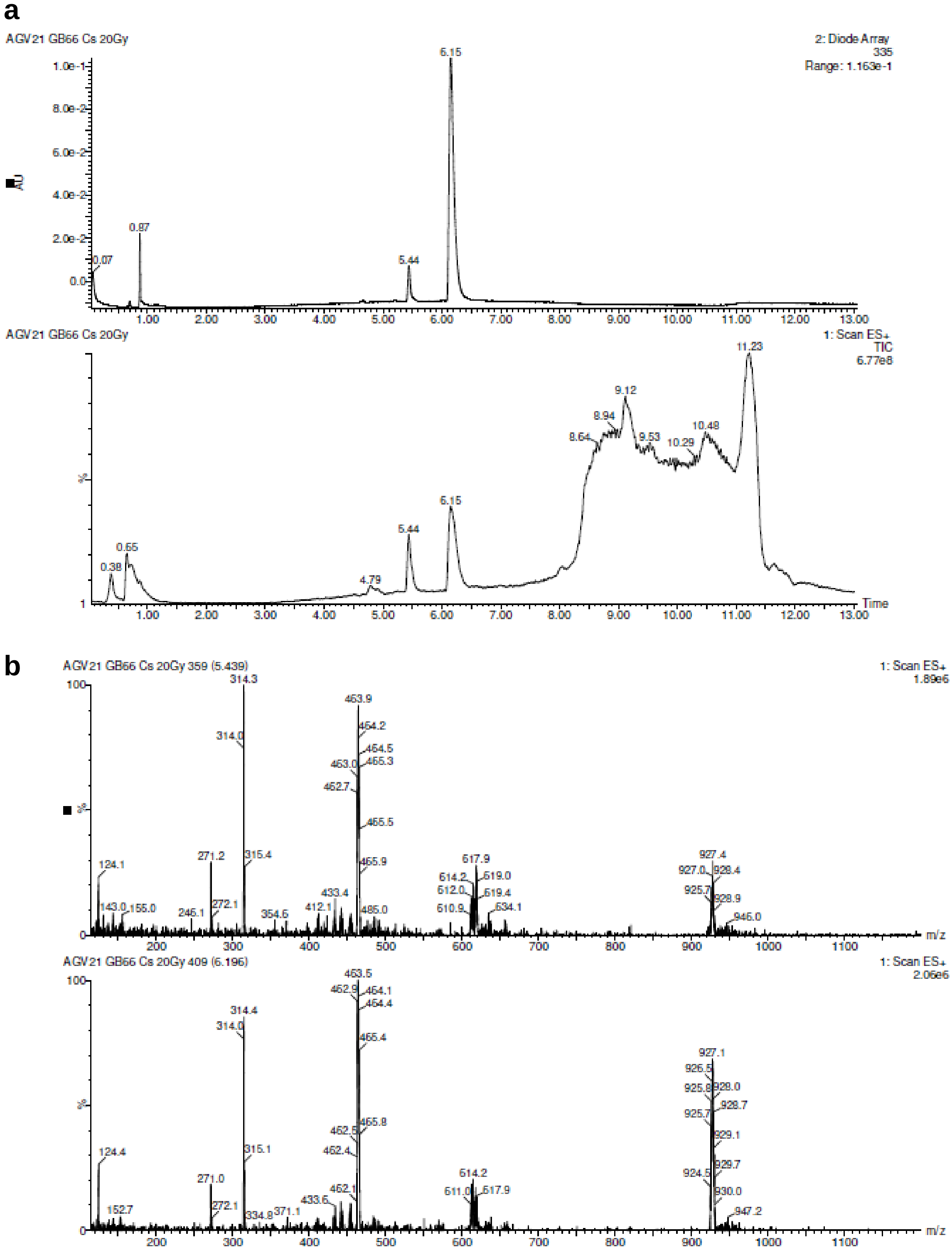

Fig. 42. LC-MS of GdAzo after 20 Gy gamma-ray irradiation. a, Absorbance (335 nm) and total ion current (positive mode) chromatograms from top to bottom. **b**, Mass spectra (positive mode) of *cis*-**GdAzo** (top, t_R = 5.439 min) and *trans*-**GdAzo** (bottom, t_R = 6.196 min).

5.2.d - After 20 Gy electron-beam irradiation

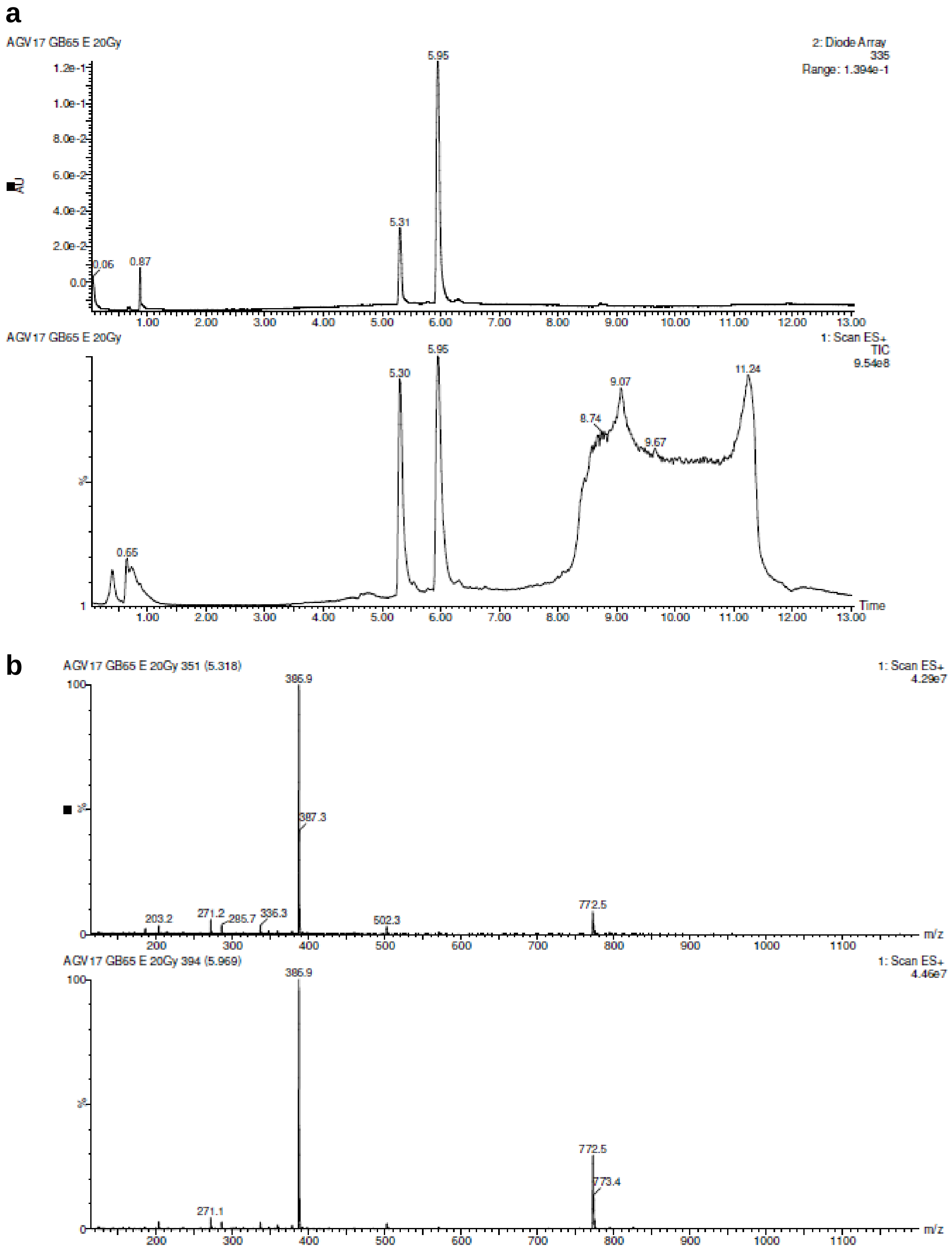

Fig. 43. LC-MS of Azo after 20 Gy electron-beam irradiation. a, Absorbance (335 nm) and total ion current (positive mode) chromatograms from top to bottom. **b**, Mass spectra (positive mode) of *cis*-**Azo** (top, t_R = 5.318 min) and *trans*-**Azo** (bottom, t_R = 5.969 min).

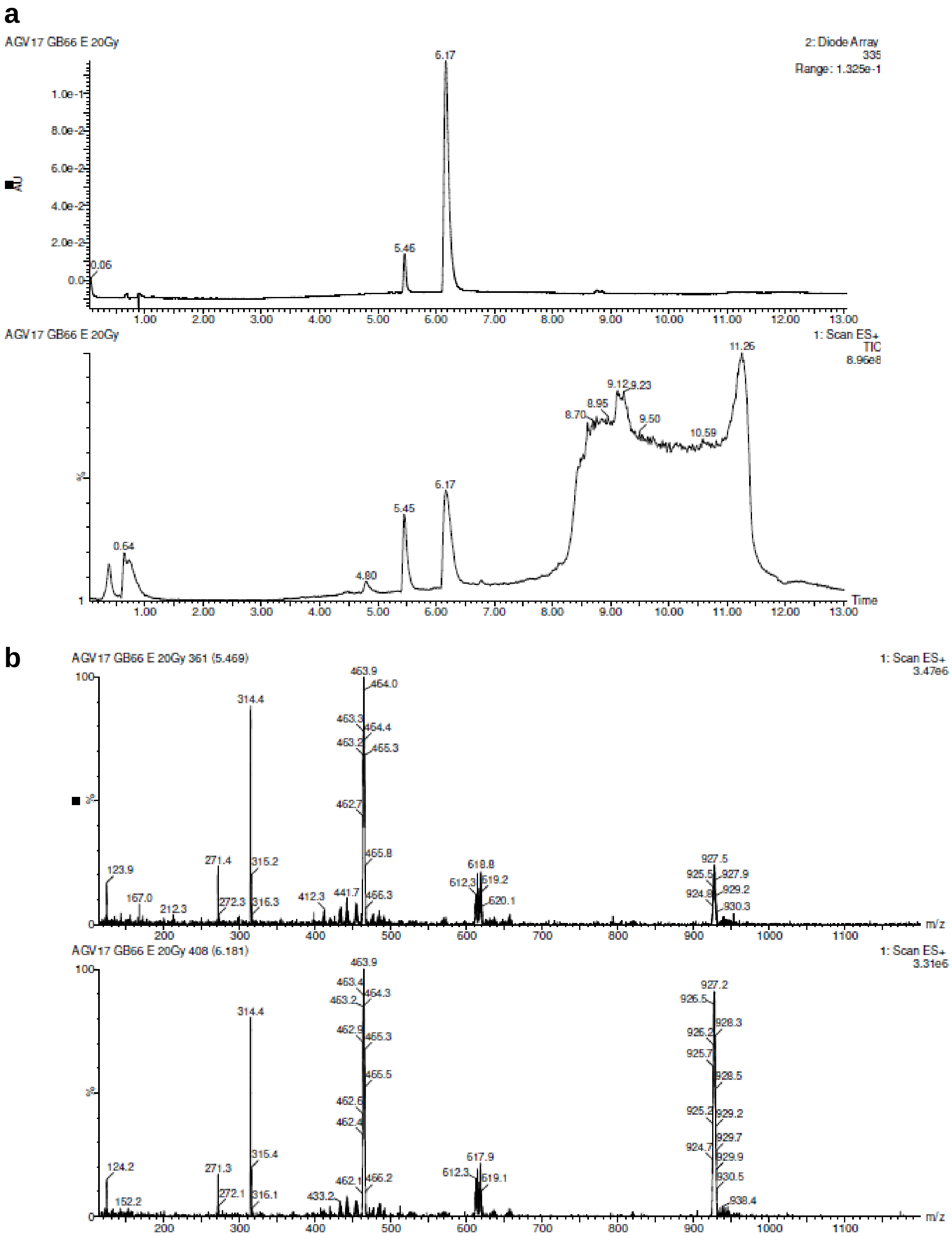

Fig. 44. LC-MS of GdAzo after 20 Gy electron-beam irradiation. a, Absorbance (335 nm) and total ion current (positive mode) chromatograms from top to bottom. **b**, Mass spectra (positive mode) of *cis*-**GdAzo** (top, t_R = 5.469 min) and *trans*-**GdAzo** (bottom, t_R = 6.181 min).

5.3 - Confirmation of isomerisation reversibility

The compound (*trans*-**Azo** or *trans*-**GdAzo**, 50 µM, 200 µL, PBS) was introduced in a 96-well microplate which was successively irradiated by UV (365 nm, 0.817 mW.cm^{-2}, 5 min) and gamma-rays (2 Gy) (4 repetitions). After each irradiation, absorbance was monitored (triplicate).

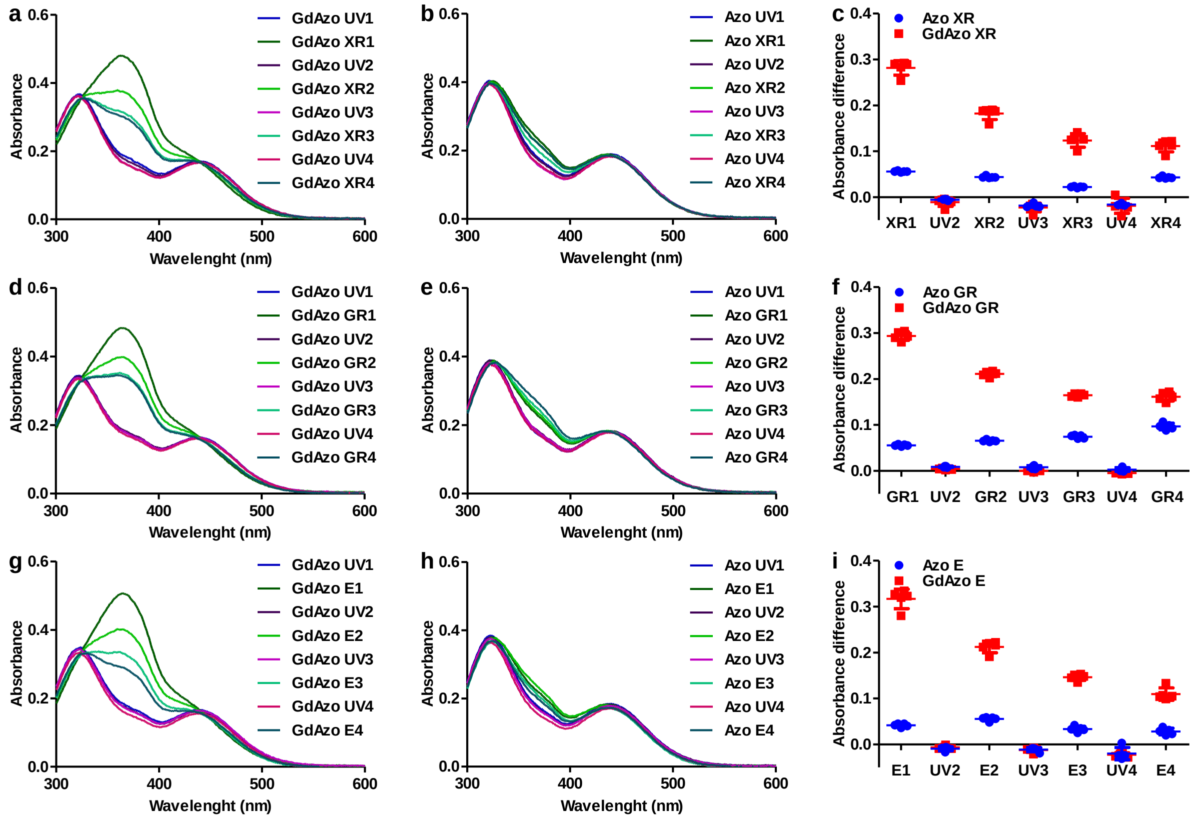

Fig. 45. Confirmation of isomerisation reversibility. Absorbance spectra of **GdAzo** compound (**a**, **d**, **g**) and **Azo** compound (**b**, **e**, **h**) (50 µM in PBS) successively irradiated by UV (365 nm, 0.817 mW.cm^{-2}, 5 min) and X-rays (XR, **a-c**), gamma-rays (GR, **d-f**) or electron beam (E, **g-i**) (2 Gy) over 4 cycles. **c**, **f**, **i**, Absorbance difference (365 nm) between the first UV irradiation (UV1) and the other irradiation treatments (successively ionising radiation and UV, quintuplicate). Means ± standard deviations are reported. This experiment confirmed that *cis-trans* isomerisation is the activation process occurring upon ionising radiation. The decrease of efficacy over the cycles could be explained by the reduction of the amount of chloride ions Cl^- over cycles (137 mM NaCl in PBS). Indeed chloride ions generate the oxidative $Cl_2^{\bullet -}$ radicals upon ionising radiations whose presence may impact *cis*-**GdAzo** activation efficacy (cf. Supplementary Section 7).

5.4 - Determination of *G*-values and comparison of the radiation sources

Compound	Source	Ionising radiation				Control without radiation			
		2 Gy	5 Gy	10 Gy	20 Gy	Comp 2 Gy	Comp 5 Gy	Comp 10 Gy	Comp 20 Gy
cis-Azo	XR	5.5 ± 1.1	10.5 ± 2.9	14.9 ± 1.3	20.7 ± 2.3	3.8 ± 1.6	7.9 ± 2.7	10.2 ± 2.2	15.2 ± 3.7
	GR	3.2 ± 0.9	9.6 ± 2.7	14.2 ± 1.8	20.7 ± 2.6	3.6 ± 0.7	6.9 ± 2.0	10.3 ± 1.6	13.1 ± 1.4
	E	3.4 ± 2.3	9.5 ± 2.4	14.0 ± 3.5	17.7 ± 3.6	3.1 ± 1.7	6.6 ± 0.3	10.8 ± 1.9	12.3 ± 3.0
cis-GdAzo	XR	30.0 ± 1.8	45.5 ± 1.7	60.2 ± 1.8	68.1 ± 1.5	3.2 ± 1.3	7.7 ± 2.2	11.7 ± 0.9	15.9 ± 1.6
	GR	33.4 ± 1.0	50.3 ± 1.2	62.6 ± 2.7	68.6 ± 2.9	2.5 ± 0.7	5.2 ± 0.5	10.5 ± 0.6	12.9 ± 1.1
	E	36.2 ± 0.9	54.5 ± 0.3	64.7 ± 2.3	71.8 ± 1.9	3.8 ± 0.8	6.0 ± 1.1	10.1 ± 1.0	13.9 ± 1.4

Table 2. Molecular activation of *cis*-Azo and *cis*-GdAzo upon ionising radiation. The molecular activation (%) was determined by HPLC (method C) and represents the increase in proportion of the *trans*-isomer in the media from PSS1 (3 independent experiments). “Comp” refers to the non-irradiated compound analysed concurrently with the irradiated compound at the mentioned dose. The means ± standard deviations are reported. XR : X-ray irradiation ; GR : gamma-ray irradiation ; E: electron-beam irradiation.

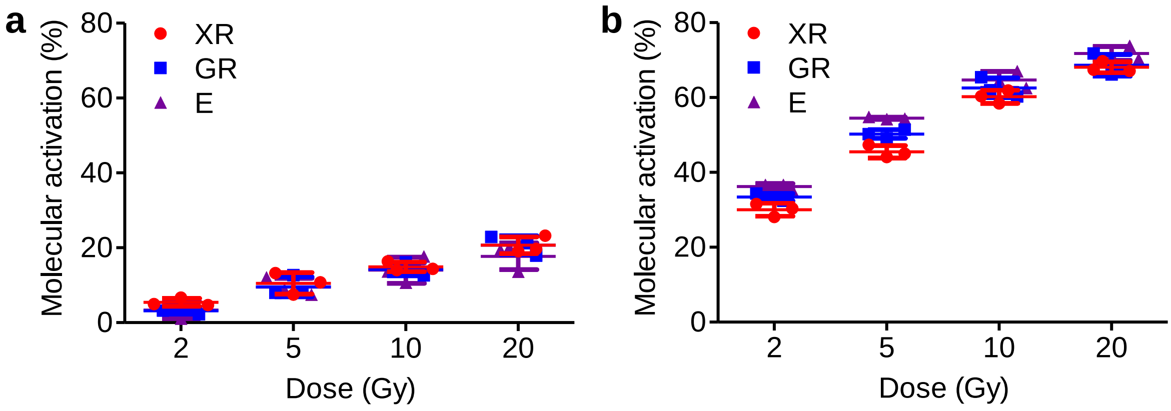

Fig. 46. Comparison of XR, GR and E sources using molecular activation. Molecular activation of *cis*-**Azo** (**a**) and *cis*-**GdAzo** (**b**) determined by HPLC (method C) and reported as the difference of the *trans*-isomer proportion before and after ionising radiation (3 independent experiments). The means ± standard deviations are reported. Two-way Anova (Bonferroni post-test) was used for statistical analyses. Even if the molecular activation of *cis*-**GdAzo** upon X-ray (XR), gamma-ray (GR) and electron-beam (E) irradiations were in the same order of magnitude (compared to the *cis*-**Azo** activation), small significant differences were observed depending on the irradiation dose: XR *vs* GR (* at 5 Gy), XR *vs* E (*** at 2 Gy, *** at 5 Gy, * at 10 Gy) and GR *vs* E (* at 5 Gy). * $P < 0.05$, *** $P < 0.001$.

Compound	Source	Ionising radiation			
		2 Gy	5 Gy	10 Gy	20 Gy
cis-Azo	XR	0.43 ± 0.45	0.26 ± 0.30	0.23 ± 0.15	0.14 ± 0.12
	GR	-0.12 ± 0.43	0.30 ± 0.12	0.21 ± 0.05	0.20 ± 0.06
	E	0.07 ± 0.80	0.29 ± 0.22	0.16 ± 0.24	0.14 ± 0.17
cis-GdAzo	XR	6.70 ± 0.64	3.78 ± 0.39	2.42 ± 0.13	1.31 ± 0.01
	GR	8.42 ± 0.16	4.89 ± 0.11	2.81 ± 0.18	1.50 ± 0.06
	E	8.11 ± 0.03	4.84 ± 0.08	2.73 ± 0.07	1.45 ± 0.04

Table 3. *G*-values of *cis*-Azo and *cis*-GdAzo upon ionising radiation. The *G*-value defines the yield of activation upon ionising radiation (µmol/J). It was determined by HPLC (method C) and represents the molar amount of *trans*-**GdAzo** activation corrected from thermal back relaxation (activation due to thermal relaxation determined by control experiment) and divided by the energy received by the medium (3 independent experiments). The means ± standard deviations are reported. XR : X-ray irradiation ; GR : gamma-ray irradiation ; E: electron-beam irradiation.

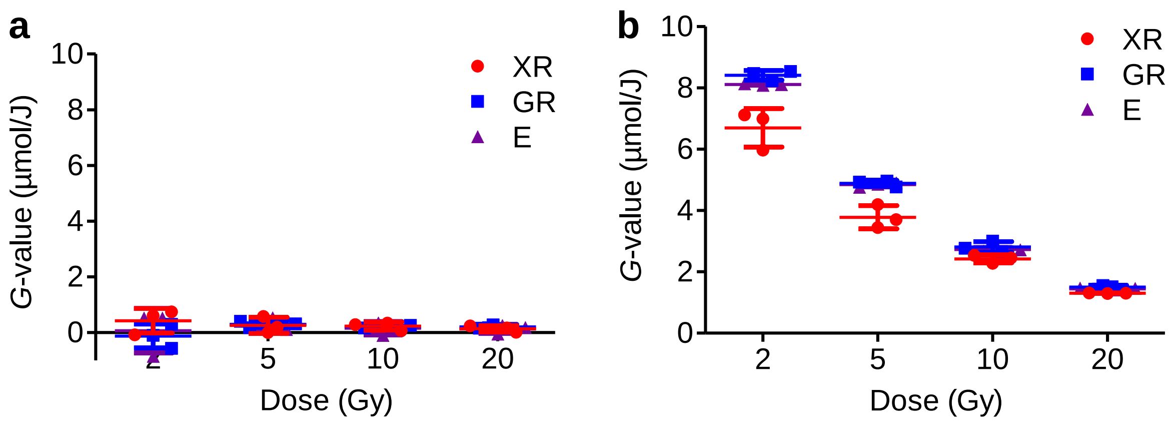

Fig. 47. Comparison of XR, GR and E sources using *G*-value. *G*-values of *cis*-**Azo** (**a**) and *cis*-**GdAzo** (**b**) determined by HPLC (method C) and reported as the molar amount of *trans*-**GdAzo** activation corrected from thermal back relaxation and divided by the energy received by the medium (3 independent experiments). The means ± standard deviations are reported. Two-way Anova (Bonferroni post-test) was used for statistical analyses. Similarly as for the molecular activations, the *G*-values of *cis*-**GdAzo** upon X-ray (XR), gamma-ray (GR) and electron-beam (E) irradiations were in the same order of magnitude (compared to the *G*-values of *cis*-**Azo**), even if small significant differences were observed depending on the irradiation dose: XR *vs* GR (*** at 2 Gy, *** at 5 Gy) and XR *vs* E (*** at 2 Gy, *** at 5 Gy). ** $P < 0.01$, *** $P < 0.001$.

5.5 - Determination of activation constants

The compound (*trans*-**GdAzo** or *trans*-**Azo**, photostationary state 2, PSS2) was introduced in a 96-well microplate (50 µM, 200 µL, PBS) which was irradiated by UV (365 nm, 0.817 mW.cm^{-2}, 5 min) to obtain the *cis*-isomer (90 ± 3%, photostationary state 1, PSS1). Then the microplate was irradiated (X-ray, gamma-ray or electron beam) upon incremental doses of ionising radiations (0.5 Gy increment in the 0.5-2 Gy range and 1 Gy increment in the 2-8 Gy). After each irradiation, absorbance was recorded (triplicate). Ln[(A0-Ainf)/(At-Ainf)] (with A0 the absorbance of PSS1, Ainf the absorbance of PSS2 and At the absorbance after irradiation by ionising radiations) *versus* the irradiation dose was plotted for the 0.5-2 Gy dose range in order to determine an activation constant for each compound considering first-order reaction (Figs. 48-50). The experiment (in the 0.5-2 Gy dose range) was performed in 20 min for ignoring thermal back relaxation. The experiment was repeated 3 times independently.

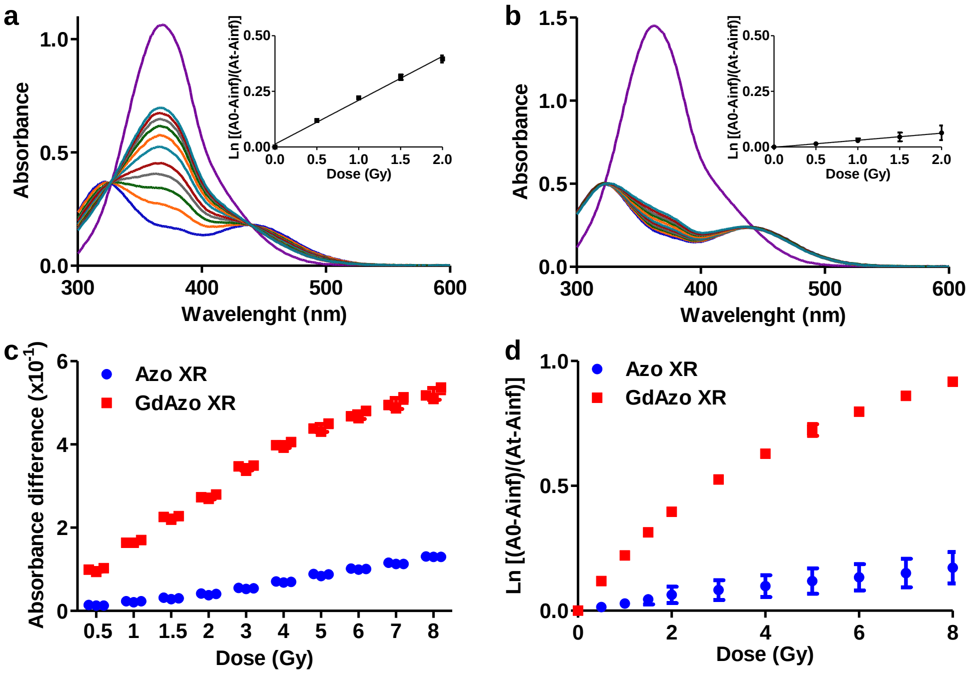

Fig. 48. Determination of *cis*-GdAzo and *cis*-Azo activation constants upon X-ray irradiation. a, **b**, Absorbance spectra of medium containing **GdAzo** compound (**a**) or **Azo** compound (**b**) (50 µM in PBS). The blue (lowest absorbance at 365 nm) and purple (highest absorbance at 365 nm) curves were obtained from the photostationary state 1 (PSS1, *cis*-isomer major) and photostationary state 2 (PSS2, *trans*-isomer) respectively. The intermediate curves were obtained upon X-ray (XR) irradiation of the *cis*-isomers (PSS1) by 0.5 Gy increment in the 0.5-2 Gy range and 1 Gy increment in the 2-8 Gy range. The graphs Ln[(A0-Ainf)/(At-Ainf)] by the irradiation dose (in the 0.5-2 Gy range) are plotted on the top-right corners, with A0 the absorbance of PSS1, Ainf the absorbance of PSS2 and At the absorbance at a specific irradiation dose. Linear regressions (least-squares method, r^2: 0.9919 and 0.7009 for *cis*-**GdAzo** and *cis*-**Azo** respectively, GraphPad Prism 5.00) were used to determine activation constants at low dose (< 2 Gy). The activation constants upon XR irradiation are $k = (1.97 \pm 0.05)*10^{-1}$ Gy^{-1} for *cis*-**GdAzo** compound and $k = (3.2 \pm 0.6)*10^{-2}$ Gy^{-1} for *cis*-**Azo** compound (triplicate, n=3, *F*-values = 1583 and 30.46 for *cis*-**GdAzo** and *cis*-**Azo** respectively, $P < 0.0001$). The irradiation experiment in the 0.5-2 Gy range was performed in less than 20 min in order to neglect the thermal back relaxation of the *cis*-isomers. **c**, Absorbance difference (365 nm) of media containing *cis*-**GdAzo** or *cis*-**Azo** compound (PSS1, 50 µM in PBS) before and after XR irradiation (triplicate). **d**, Plot of Ln[(A0-Ainf)/(At-Ainf)] by the XR-irradiation dose (0-8 Gy) for the **GdAzo** and **Azo** compounds (50 µM in PBS) (triplicate, n=3). Means ± standard deviations are reported.

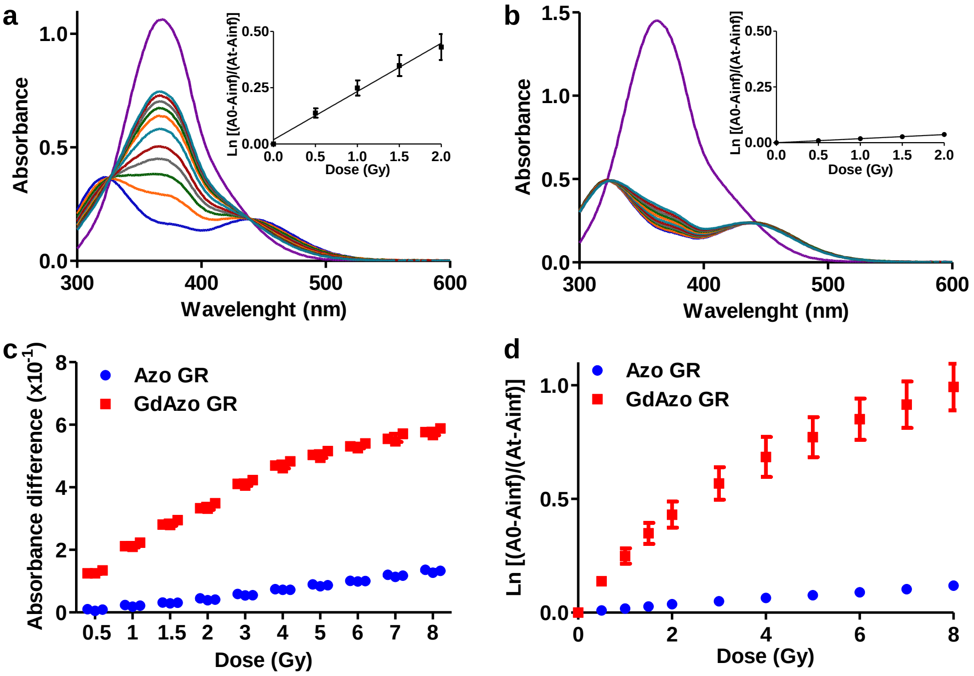

Fig. 49. Determination of *cis*-GdAzo and *cis*-Azo activation constants upon gamma-ray irradiation. a, **b**, Absorbance spectra of medium containing **GdAzo** compound (**a**) or **Azo** compound (**b**) (50 µM in PBS). The blue (lowest absorbance at 365 nm) and purple (highest absorbance at 365 nm) curves were obtained from the photostationary state 1 (PSS1, *cis*-isomer major) and photostationary state 2 (PSS2, *trans*-isomer) respectively. The intermediate curves were obtained upon gamma-ray (GR) irradiation of the *cis*-isomers (PSS1) by 0.5 Gy increment in the 0.5-2 Gy range and 1 Gy increment in the 2-8 Gy range. The graphs Ln[(A0-Ainf)/(At-Ainf)] by the irradiation dose (in the 0.5-2 Gy range) are plotted on the top-right corners, with A0 the absorbance of PSS1, Ainf the absorbance of PSS2 and At the absorbance at a specific irradiation dose. Linear regressions (least-squares method, r^2: 0.9521 and 0.9836 for *cis*-**GdAzo** and *cis*-**Azo** respectively, GraphPad Prism 5.00) were used to determine activation constants at low dose (< 2 Gy). The activation constants upon GR irradiation are $k = (2.1 \pm 0.2)*10^{-1}$ Gy^{-1} for *cis*-**GdAzo** compound and $k = (1.83 \pm 0.07)*10^{-2}$ Gy^{-1} for *cis*-**Azo** compound (triplicate, n=3, F-values = 258.3 and 778.7 for *cis*-**GdAzo** and *cis*-**Azo**, $P < 0.0001$). The irradiation experiment in the 0.5-2 Gy range was performed in less than 20 min in order to neglect the thermal back relaxation of the *cis*-isomers. **c**, Absorbance difference (365 nm) of media containing *cis*-**GdAzo** or *cis*-**Azo** compound (PSS1, 50 µM in PBS) before and after GR irradiation (triplicate). **d**, Plot of Ln[(A0-Ainf)/(At-Ainf)] by the GR-irradiation dose (0-8 Gy) for the **GdAzo** and **Azo** compounds (50 µM in PBS) (triplicate, n=3). Means ± standard deviations are reported.

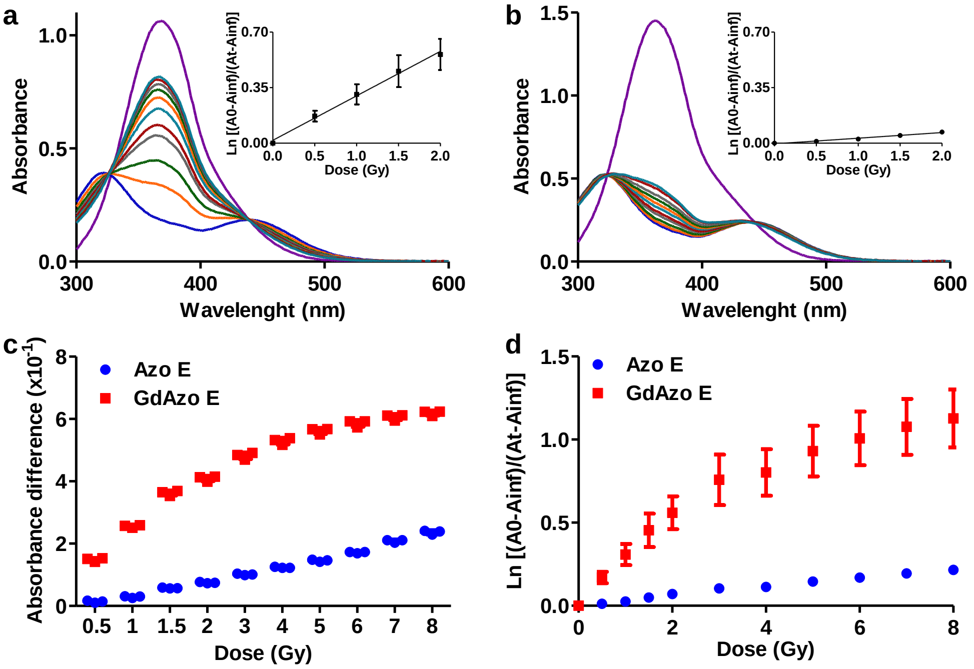

Fig. 50. Determination of *cis*-GdAzo and *cis*-Azo activation constants upon electron-beam irradiation. a, **b**, Absorbance spectra of medium containing **GdAzo** compound (**a**) or **Azo** compound (**b**) (50 µM in PBS). The blue (lowest absorbance at 365 nm) and purple (highest absorbance at 365 nm) curves were obtained from the photostationary state 1 (PSS1, *cis*-isomer major) and photostationary state 2 (PSS2, *trans*-isomer) respectively. The intermediate curves were obtained upon electron-beam (E) irradiation of the *cis*-isomers (PSS1) by 0.5 Gy increment in the 0.5-2 Gy range and 1 Gy increment in the 2-8 Gy range. The graphs Ln[(A0-Ainf)/(At-Ainf)] by the irradiation dose (in the 0.5-2 Gy range) are plotted on the top-right corners, with A0 the absorbance of PSS1, Ainf the absorbance of PSS2 and At the absorbance at a specific irradiation dose. Linear regressions (least-squares method, r^2: 0.9168 and 0.9525 for *cis*-**GdAzo** and *cis*-**Azo** respectively, GraphPad Prism 5.00) were used to determine activation constants at low dose (< 2 Gy). The activation constants upon E irradiation are $k = (2.8 \pm 0.3)*10^{-1}$ Gy^{-1} for *cis*-**GdAzo** compound and $k = (3.6 \pm 0.3)*10^{-2}$ Gy^{-1} for *cis*-**Azo** compound (triplicate, n=3, F-values = 143.2 and 260.6 for *cis*-**GdAzo** and *cis*-**Azo** respectively, $P < 0.0001$). The irradiation experiment in the 0.5-2 Gy range was performed in less than 20 min in order to neglect the thermal back relaxation of the *cis*-isomers. **c**, Absorbance difference (365 nm) of media containing *cis*-**GdAzo** or *cis*-**Azo** compound (PSS1, 50 µM in PBS) before and after E irradiation (triplicate). **d**, Plot of Ln[(A0-Ainf)/(At-Ainf)] by the E-irradiation dose (0-8 Gy) for the **GdAzo** and **Azo** compounds (50 µM in PBS) (triplicate, n=3). Means ± standard deviations are reported.

Supplementary Section 6

Monte Carlo simulations

In order to provide insights into the *cis*-**GdAzo** activation mechanism upon X-ray (XR), gamma-ray (GR) and electron-beam (E) irradiations, complementary Monte Carlo simulations (PENELOPE code)[1] were performed. PENELOPE allows the simulation of coupled electron-photon transport in a wide energy range (from 1 GeV down to, nominally, 50 eV) in arbitrary materials and complex geometries. In this work, an irradiation of a Gd atom was considered. The three beam sources were modelled with realistic energy distributions (XR from source spectra, GR at 662 keV and E at 4.5 MeV) and directly impinged on the Gd volume. The absorption energies, at which the transport is discontinued, were set to 50 eV for both electrons and photons. The spectra of the electrons exiting the Gd volume, normalised with respect to the total number of outgoing secondary electrons, were scored. In order to optimise the computation time, variance-reduction techniques (interaction forcing) were employed in photon interactions.

Supplementary Section 7

Investigation of activation mechanism using various converter species

General information

Same equipment as described in the Supplementary Section 5 was used. Phosphate buffered saline (PBS), *tert*-butanol (tBuOH), D-mannitol, ethanol (EtOH), cadmium perchlorate hydrate ($Cd(ClO_4)_2$), dimethylsulfoxyde (DMSO), sodium azide (NaN_3) and sodium selenate decahydrate (Na_2SeO_4) were purchased from Sigma Aldrich (France).

7.1 - Protocol

A solution of *trans*-**GdAzo** (50 µM, 200 µL, media) was introduced in two 96-well microplates. Both microplates were irradiated by UV (365 nm, 0.817 $mW.cm^{-2}$, 5 min). One microplate (plate 1) was kept in the dark and used as control (non-irradiated by ionising radiation (IR)) whereas the second microplate (plate 2) was irradiated (X-ray, gamma-ray or electron beam) upon incremental doses of IRs (2, 3, 5 and 10 Gy). After each irradiation, absorbance analyses were performed on the non-irradiated (plate 1) and IR-irradiated (plate 2) compounds (triplicate or duplicate). Histograms showing absorbance difference (365 nm) of the media before and after IRs were used for interpretation. The media used were: water (control), aqueous solutions of tBuOH (10, 100 and 524 mM ~5% v/v), D-mannitol (10, 100 and 524 mM), EtOH (10, 100 and 524 mM), NaN_3 (1, 10 and 50 mM), DMSO (15% v/v), $Cd(ClO_4)_2$ (20 mM) and Na_2SeO_4 (1 and 25 mM).

7.2 - Results

Irradiation experiments carried out in the presence of different quenchers/converters revealed some key information about the activation (isomerisation) mechanism upon IR. During IR of water, several types of reactive oxygen species are generated coming from the excitation and ionisation of the water molecules, followed by the dissociation of the excited species.[2,3] For low linear energy transfer irradiations (and for incident photon energy above ~100 keV), the firstly produced species are mainly prehydrated electrons leading to hydrated electrons

(G = 0.280 µmol/J after ~100 ns, with G the radiolytic yield), hydrogen ($H^{\bullet}$) and hydroxyl ($HO^{\bullet}$) radicals (G = 0.062 µmol/J and 0.280 µmol/J respectively after ~100 ns) (Fig. 51). Other more stable oxidant reactive species such as hydrogen peroxide (H_2O_2, G = 0.073 µmol/J) will be formed later on.

a

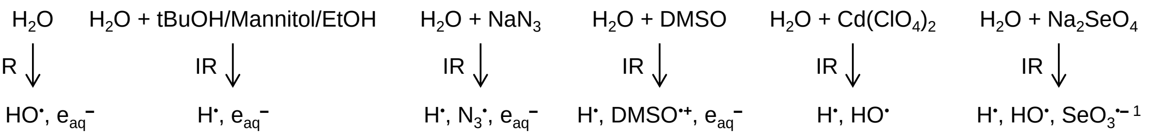

b

Species	Convertion induced upon IR	Chemical effect upon IR	Effect on *cis* -GdAzo activation
tBuOH	$HO^{\bullet}$ > tertiary radical	Quench $HO^{\bullet}$ reactivity	Abolish
Mannitol	$HO^{\bullet}$ > unreactive radical	Quench $HO^{\bullet}$ reactivity	Abolish
EtOH	$HO^{\bullet}$ > reducing radical	Quench $HO^{\bullet}$ reactivity	Abolish
NaN_3	$HO^{\bullet}$ > $N_3^{\bullet}$	Selective electron transfer oxidation	Increase
DMSO	$HO^{\bullet}$ > $DMSO^{\bullet+}$	Oxidation	Increase
$Cd(ClO_4)_2$	e_{aq}^- > Cd^+	-	No effect
Na_2SeO_4	e_{pre}^- and e_{aq}^- > $SeO_3^{\bullet-}$ [1]	Oxidation	Increase

Fig. 51. **a**, Main radicals generated upon water radiolysis in the presence of quenchers/converters. **b**, Effect of radical quenchers/converters on the *cis*-**GdAzo** activation upon ionising radiation (IR, X-ray, gamma-ray and electron beam). tBuOH: *tert*-butanol; EtOH: ethanol; NaN_3: sodium azide; DMSO: dimethylsulfoxyde; $Cd(ClO_4)_2$: cadmium perchlorate; Na_2SeO_4: sodium selenate. e_{aq}^-: hydrated electron. e_{pre}^-: prehydrated electron. >: converted to. [1]: other oxidant species may be released.[4,5]

We observed that tBuOH was able to quench *cis*-**GdAzo** activation (Fig. 52). This chemical species converts the highly reactive hydroxyl radicals $HO^{\bullet}$ generated upon IR into much less reactive tertiary radicals. Therefore, the activation process is mainly dependent on the $HO^{\bullet}$ production (or species generated from $HO^{\bullet}$), which suggests that *cis*-**GdAzo** activation proceeds through an oxidation pathway, meaning an oxidation of the azo double bond ($N{=}N^{\bullet+}$). To confirm this conclusion, the impact of two other specific quenchers of $HO^{\bullet}$, mannitol and EtOH, on *cis*-**GdAzo** activation upon gamma ray was assessed (Fig. 53). As expected, tBuOH, mannitol and EtOH showed a similar, clear and drastic inhibition of *cis*-**GdAzo** activation upon gamma ray. These concordant results demonstrate that *cis*-**GdAzo** activation upon IR results from the presence of $HO^{\bullet}$.

Nevertheless, we were surprised to observe that other chemical species known to convert the $HO^•$ radicals, such as NaN_3 (which also interacts with $H^•$) or DMSO, were actually increasing the *cis*-**GdAzo** activation efficacy (Fig. 55). This behaviour can be explained by the new species generated by these converters after the interaction with $HO^•$, which are the azide radical ($N_3^•$) and DMSO radical ($DMSO^{•+}$) respectively.[6,7] Both these species are oxidants and their impact on *cis*-**GdAzo** activation efficacy consolidates the mechanism hypothesis on an oxidation process. The increased activation efficacy in the presence of these two converters is probably due to the higher ability of the generated radical species to induce azo-bond oxidation. Indeed, the $HO^•$ radical is highly reactive and poorly selective (1.90 V at neutral pH *vs* standard hydrogen electrode (SHE) compared to 1.31 V *vs* SHE for $N_3^•/N_3^−$ system)[8] and is able to induce other types of reactions than electron transfer such as hydrogen atom abstraction (by interacting with $H^•$ radical or by removing $H^•$ from a substrate), addition to unsaturated bond, interaction with an hydrated electron or with another $HO^•$ radical to make $HO^−$ or H_2O_2 respectively. It has to be noted that the decrease of activation efficacy, observed when the concentration of NaN_3 was increased, is certainly due to the recombination of $N_3^•$ radicals to form nitrogen gas.

$Cd(ClO_4)_2$ (20 mM) is known to quench hydrated electrons mainly whereas Na_2SeO_4 (at concentration above 1 mM) is known to also quench prehydrated electrons.[5] Interestingly, $Cd(ClO_4)_2$ has no impact on the *cis*-**GdAzo** activation (Fig. 55). On the other hand, Na_2SeO_4 slightly increases activation at low concentration (1 mM, effect improved at higher IR doses) whereas it induces a major activation increase at high concentration (25 mM) upon 2 Gy (independent from the IR dose) (Fig. 55). These observations led us to conclude that prehydrated and hydrated electrons are not involved in the *cis*-**GdAzo** activation process. The increase in activation at high concentration (25 mM) of Na_2SeO_4 is probably due to the high concentration of oxidant species generated upon IR such as $SeO_3^{•−}$ (1.68 V *vs* SHE).[3-5]

A similar behaviour of *cis*-**GdAzo** activation was observed for the three irradiation sources (X-ray, gamma-ray and electron beam) (Figs. 54-56), which suggests the same activation mechanism upon the different sources. This is in accordance with the similar activation efficacy obtained with the different IR sources. These experiments clearly reveal that the interactions of the primary incident particles with *cis*-**GdAzo** have no influence on the activation process since *cis*-**GdAzo** activation operates similarly upon X-ray (incident

particles at 30-140 keV), gamma-rays (incident particles at 662 keV) and electron beam (incident particles at 4.5 MeV). Moreover, the Čerenkov effect (exclusively upon electron beam) also has low impact. The activation process originates from the low-energy particles and species released during the energy dissipation in water. These results corroborate with the Monte Carlo simulations (Supplementary Section 6) revealing a similar amount of relative low-energy secondary electrons generated by the three different IR sources in the presence of Gd. Indeed, activation of *cis*-**GdAzo** by high-energy particles (662 keV and 4.5 MeV for gamma-rays and electron beam respectively) is possible thanks to their energy loss occurring in the media.

These investigations led us to conclude that *cis*-**GdAzo** activation is happening through the oxidation of the azo double bond by reaction with $HO^{\bullet}$ which is generated by the low-energy particles released upon IR. The different experimental approaches implemented (Monte Carlo simulations, experiments upon different IR sources and in the presence of radical-interacting species) were consistent with this activation pathway. However, we were not able to elucidate the full process leading to *cis*-**GdAzo** activation upon IR. The mechanism pathway is probably more complex. Indeed, the production yield of the $HO^{\bullet}$ radicals in water upon IR is about G = 0.28 µmol/J (upon gamma-rays or electron beam) whereas the activation yield of *cis*-**GdAzo** is about 8.4 ± 0.2 µmol/J (upon 2 Gy gamma-rays, corrected from thermal back relaxation) which is much higher. This can be explained by (i) a higher amount of hydroxyl radicals $HO^{\bullet}$ produced in the presence of *cis*-**GdAzo**, by (ii) other oxidant species involved, (iii) an activation before $HO^{\bullet}$ escape in the bulk solution (within the spurs and before $HO^{\bullet}$ recombination for instance) or by (iv) a catalytic effect (chain reaction) during the reduction step of the oxidised azo bond, required to make the final *trans*-**GdAzo** compound (a substoichiometric amount of *cis*-**GdAzo** would need to be oxidised). This last hypothesis was investigated and was finally rejected (Supplementary Section 10).

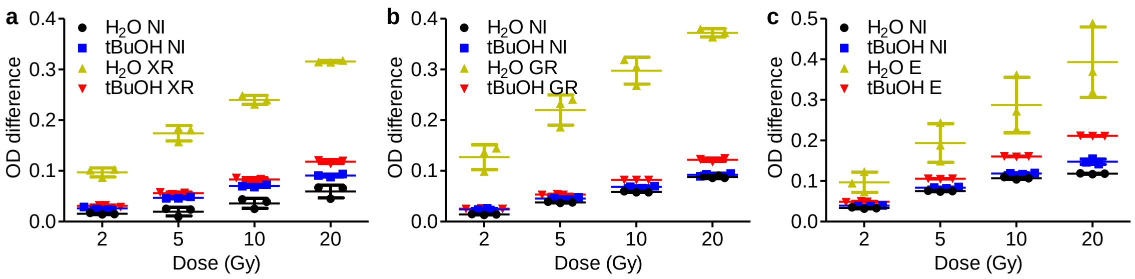

Fig. 52. Absorbance difference (365 nm) of medium containing *cis*-**GdAzo** compound (50 µM) before and after X-ray (XR, **a**), gamma-ray (GR, **b**) or electron-beam (E, **c**) irradiations at different doses (triplicate). The compound was dissolved in MilliQ water or in aqueous solution of *tert*-butanol (tBuOH, 5% v/v, 524 mM). The non-irradiated (NI) control samples are presented on each graph. The values ± standard deviations are reported. OD: optical density.

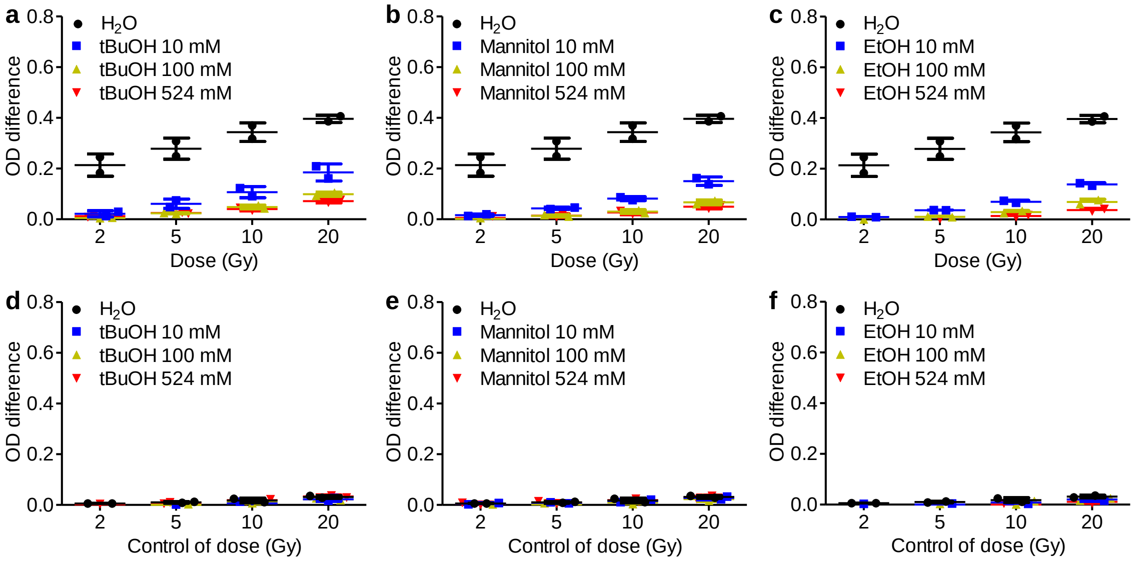

Fig. 53. Absorbance difference (365 nm) of medium containing *cis*-**GdAzo** compound (50 µM) before and after gamma-ray at different doses (**a**, **b**, **c**) or non irradiated (**d**, **e**, **f**) (triplicate or duplicate). The compound was dissolved in MilliQ water (control) or in aqueous solution of *tert*-butanol (tBuOH, **a**, **d**, 10, 100, 524 mM), mannitol (**b**, **e**, 10, 100, 524 mM) or ethanol (EtOH, **c**, **f**, 10, 100, 524 mM). The experiments were repeated 2 times independently. The means ± standard deviations are reported. OD: optical density.

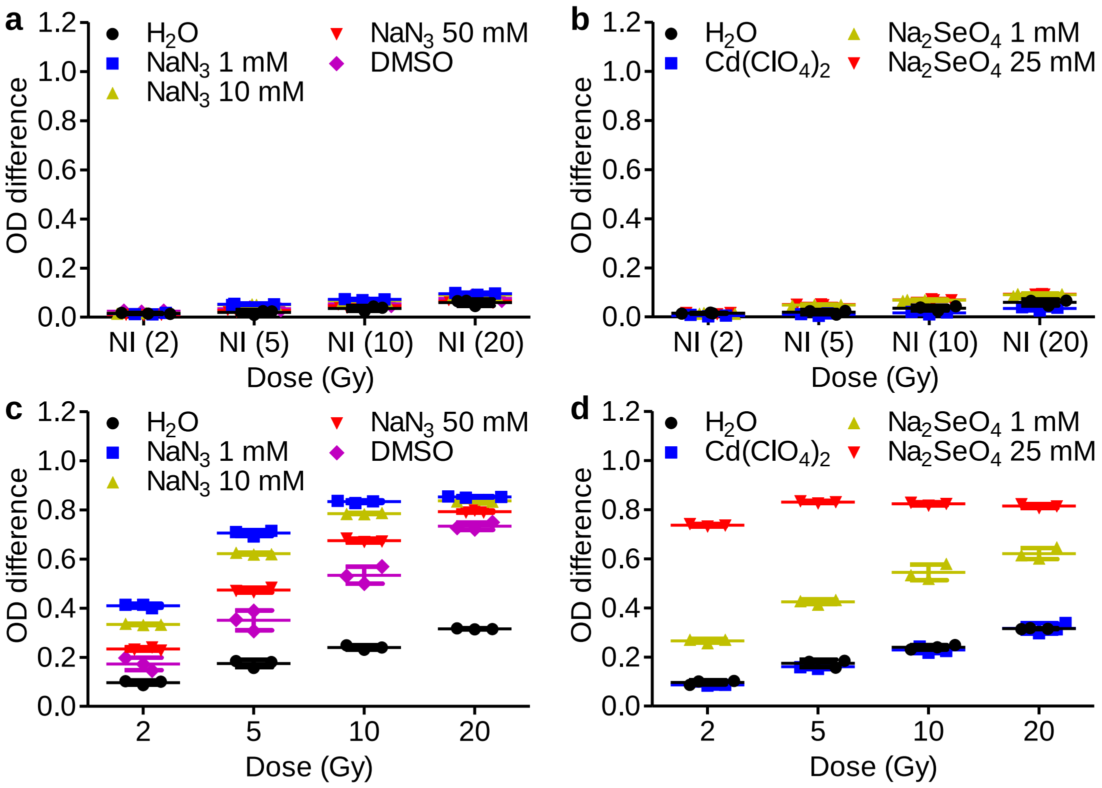

Fig. 54. Absorbance difference (365 nm) of medium containing *cis*-**GdAzo** compound (50 µM) before and after X-rays (**c**, **d**), or after leaving the medium in the dark without IR (**a**, **b**, control experiments) (triplicate). The doses in brackets (**a**, **b**) refer to the non-irradiated (NI) compound analysed concurrently with the IR-irradiated compound (**c**, **d**) at the mentioned doses. Activation efficacy was assessed within eight different media. The data are fractioned into two graphs (**a**, **c** and **b**, **d**) for ease of reading. The data from experiments carried out in water (control experiments) are reported on each graph. The data from experiments carried out in aqueous solutions of sodium azide (NaN_3, 1 mM, 10 mM and 50 mM) and dimethylsulfoxyde (DMSO, 15% v/v) are reported on (**a**) and (**c**) whereas the data from experiments carried out in aqueous solution of cadmium perchlorate ($Cd(ClO_4)_2$, 20 mM) and sodium selenate (Na_2SeO_4, 1 mM and 25 mM) are reported on (**b**) and (**d**). The values ± standard deviations are reported. OD: optical density.

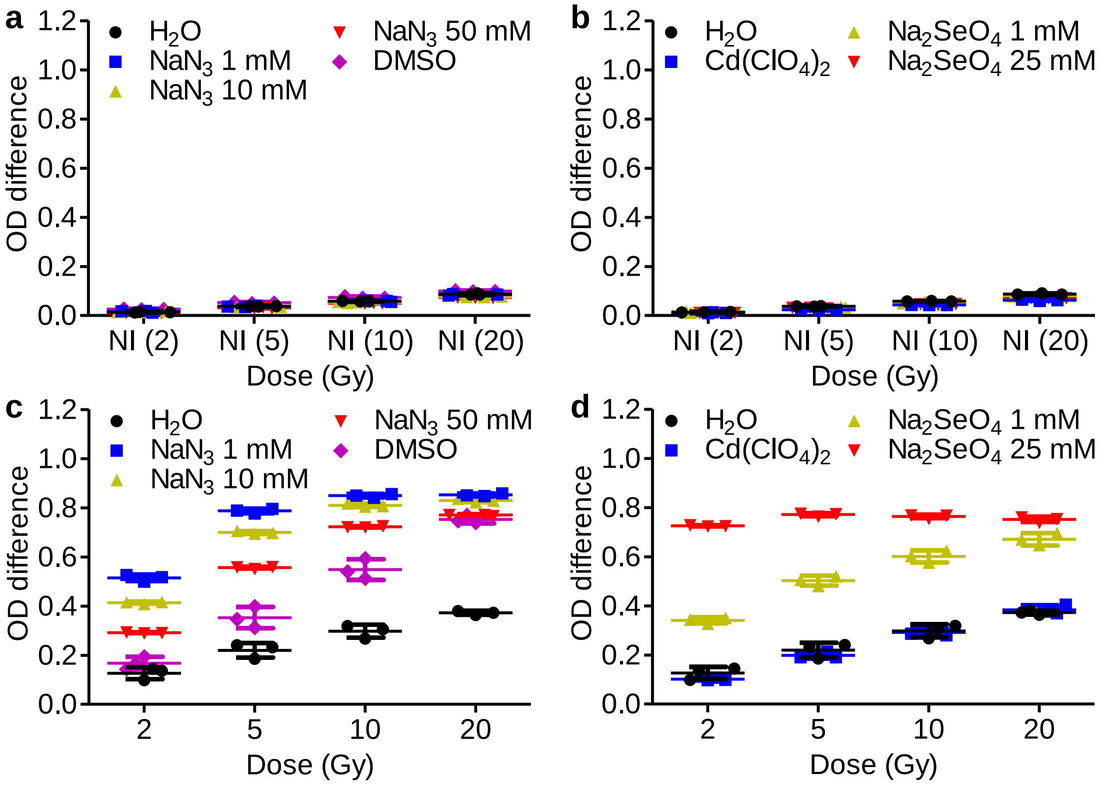

Fig. 55. Absorbance difference (365 nm) of medium containing *cis*-**GdAzo** compound (50 µM) before and after gamma-rays (**c**, **d**), or after leaving the medium in the dark without IR (**a**, **b**, control experiments) (triplicate). The doses in brackets (**a**, **b**) refer to the non-irradiated (NI) compound analysed concurrently with the IR-irradiated compound (**c**, **d**) at the mentioned doses. Activation efficacy was assessed within eight different media. The data are fractioned into two graphs (**a**, **c** and **b**, **d**) for ease of reading. The data from experiments carried out in water (control experiments) are reported on each graph. The data from experiments carried out in aqueous solutions of sodium azide (NaN_3, 1 mM, 10 mM and 50 mM) and dimethylsulfoxyde (DMSO, 15% v/v) are reported on (**a**) and (**c**) whereas the data from experiments carried out in aqueous solution of cadmium perchlorate ($Cd(ClO_4)_2$, 20 mM) and sodium selenate (Na_2SeO_4, 1 mM and 25 mM) are reported on (**b**) and (**d**). The values ± standard deviations are reported. OD: optical density.

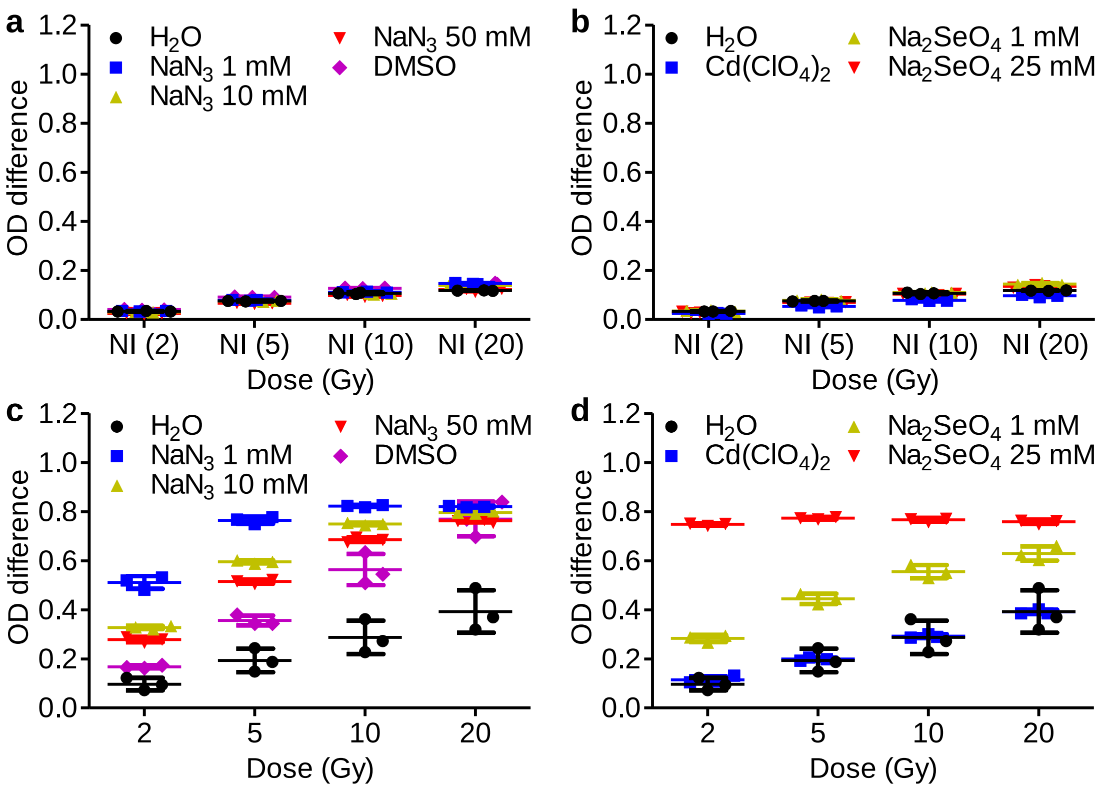

Fig. 56. Absorbance difference (365 nm) of medium containing *cis*-**GdAzo** compound (50 µM) before and after electron-beam irradiation (**c**, **d**), or after leaving the medium in the dark without IR (**a**, **b**, control experiments) (triplicate). The doses in brackets (**a**, **b**) refer to the non-irradiated (NI) compound analysed concurrently with the IR-irradiated compound (**c**, **d**) at the mentioned doses. Activation efficacy was assessed within eight different media. The data are fractioned into two graphs (**a**, **c** and **b**, **d**) for ease of reading. The data from experiments carried out in water (control experiments) are reported on each graph. The data from experiments carried out in aqueous solutions of sodium azide (NaN_3, 1 mM, 10 mM and 50 mM) and dimethylsulfoxyde (DMSO, 15% v/v) are reported on (**a**) and (**c**) whereas the data from experiments carried out in aqueous solution of cadmium perchlorate ($Cd(ClO_4)_2$, 20 mM) and sodium selenate (Na_2SeO_4, 1 mM and 25 mM) are reported on (**b**) and (**d**). The values ± standard deviations are reported. OD: optical density.

Supplementary Section 8

Investigation of activation mechanism and role of hydroxyl radicals

Same equipment as described in the Supplementary Section 5 was used. Ethylenediaminetetraacetic acid disodium salt dihydrate (EDTA), iron(II) chloride tetrahydrate ($FeCl_2$) and hydrogen peroxide solution (H_2O_2, 30% w/w in H_2O) were purchased from Sigma Aldrich (France) and nitrogen (N_2) and nitrous oxide (N_2O) gas were purchased from Air Liquide (France).

8.1 – *cis*-GdAzo activation under N_2 and N_2O gas saturation

A solution of *trans*-**GdAzo** (50 µM, 200 µL, H_2O) was introduced in a 96-well microplate before UV irradiation (365 nm, 0.817 mW.cm^{-2}, 10 min, *cis*-isomer at 90 ± 3%). The compound was then introduced into two sealed glass tubes (700 µL) and the medium was saturated by N_2 or N_2O gas bubbling for 15 min (control experiment without gas bubbling). One of the glass tube was kept in the dark and used as control (non-irradiated tube 1) whereas the second one (tube 2) was irradiated by gamma rays (GRs) upon incremental doses (2, 3, 5 and 10 Gy). After each irradiation, HPLC analyses were performed on the non-irradiated (tube 1) and GR-irradiated (tube 2) compounds. The full experiment was performed in about 1 h 40 min. The relative amount of each isomer was obtained by running HPLC at the isobestic-point conditions (method C, 6 min run, 20 µL injected) and the molecular activation (%) was determined by the difference in proportion of the *trans*-isomer in the media. The experiments were repeated 3 times independently.

**a**	No irradition (tube 1)			gamma-ray irradiation (tube 2)		
	GdAzo			GdAzo		
**Exp 1**	*trans* area	*cis* area	*trans* (%)	*trans* area	*cis* area	*trans* (%)
UV	531	4844	9,9	520	5017	9,4
2 Gy	631	4969	11,3	1778	3857	31,6
5Gy	640	4948	11,5	2423	2872	45,8
10Gy	610	4692	11,5	2732	2396	53,3
20Gy	680	4672	12,7	3088	1881	62,1
**Exp 2**	*trans* area	*cis* area	*trans* (%)	*trans* area	*cis* area	*trans* (%)
UV	568	4365	11,5	518	4325	10,7
2 Gy	608	4301	12,4	1523	2940	34,1
5Gy	615	4267	12,6	2017	2369	46,0
10Gy	641	4207	13,2	2178	1848	54,1
20Gy	627	4304	12,7	2449	1477	62,4
**Exp 3**	*trans* area	*cis* area	*trans* (%)	*trans* area	*cis* area	*trans* (%)
UV	710	5305	11,8	616	5797	9,6
2 Gy	725	5321	12,0	1854	3827	32,6
5Gy	775	5325	12,7	2408	2963	44,8
10Gy	733	5153	12,5	2751	2471	52,7
20Gy	873	5272	14,2	3113	1960	61,4

**b**	No irradition (tube 1)			gamma-ray irradiation (tube 2)		
	GdAzo			GdAzo		
**Exp 1**	*trans* area	*cis* area	*trans* (%)	*trans* area	*cis* area	*trans* (%)
UV	520	4693	10,0	579	4656	11,1
2 Gy	552	4696	10,5	1675	3167	34,6
5Gy	530	4555	10,4	2177	2469	46,9
10Gy	594	4497	11,7	2516	2005	55,7
20Gy	552	4562	10,8	2734	1504	64,5
**Exp 2**	*trans* area	*cis* area	*trans* (%)	*trans* area	*cis* area	*trans* (%)
UV	517	4501	10,3	458	4459	9,3
2 Gy	521	4409	10,6	1383	3129	30,7
5Gy	581	4401	11,7	1975	2406	45,1
10Gy	538	3935	12,0	2325	2018	53,5
20Gy	550	4161	11,7	2451	1559	61,1
**Exp 3**	*trans* area	*cis* area	*trans* (%)	*trans* area	*cis* area	*trans* (%)
UV	510	3981	11,4	502	4149	10,8
2 Gy	582	4057	12,5	1495	2690	35,7
5Gy	530	4074	11,5	2114	2243	48,5
10Gy	585	4105	12,5	2207	1722	56,2
20Gy	534	4133	11,4	2549	1378	64,9

**c**	No irradition (tube 1)			gamma-ray irradiation (tube 2)		
	GdAzo			GdAzo		
**Exp 1**	*trans* area	*cis* area	*trans* (%)	*trans* area	*cis* area	*trans* (%)
UV	638	5411	10,5	665	5281	11,2
2 Gy	825	5372	13,3	2252	3406	39,8
5Gy	840	5413	13,4	2927	2556	53,4
10Gy	835	5227	13,8	3099	2018	60,6
20Gy	818	5279	13,4	3183	1493	68,1
**Exp 2**	*trans* area	*cis* area	*trans* (%)	*trans* area	*cis* area	*trans* (%)
UV	612	4615	11,7	460	4522	9,2
2 Gy	716	4625	13,4	1972	2719	42,0
5Gy	636	4584	12,2	2431	2041	54,4
10Gy	740	4526	14,1	2572	1550	62,4
20Gy	742	4677	13,7	2526	1099	69,7
**Exp 3**	*trans* area	*cis* area	*trans* (%)	*trans* area	*cis* area	*trans* (%)
UV	469	4454	9,5	282	3711	7,1
2 Gy	501	4413	10,2	1851	2377	43,8
5Gy	574	4382	11,6	1948	1841	51,4
10Gy	522	4380	10,6	2210	1478	59,9
20Gy	541	4323	11,1	2120	1104	65,8

Table 4. Proportion of *trans*-GdAzo upon gamma-ray radiations under N_2 and N_2O gas saturation determined by HPLC. Assessment of *cis*-**GdAzo** isomerisation (50 µM, H_2O) upon gamma rays in normal atmosphere (**a**, control) or in medium saturated with N_2 (**b**) or N_2O (**c**). The data from the experiments without ionising radiation (control experiment, tube 1) and from the experiments upon gamma-ray irradiation (tube 2) are reported on the left and on the right respectively. Three independent experiments are reported (Exp 1, 2 and 3). The peak areas of both isomers were measured by HPLC (method C) after UV and gamma-ray radiations. The proportions of the *trans*-isomer were determined by the area ratios.

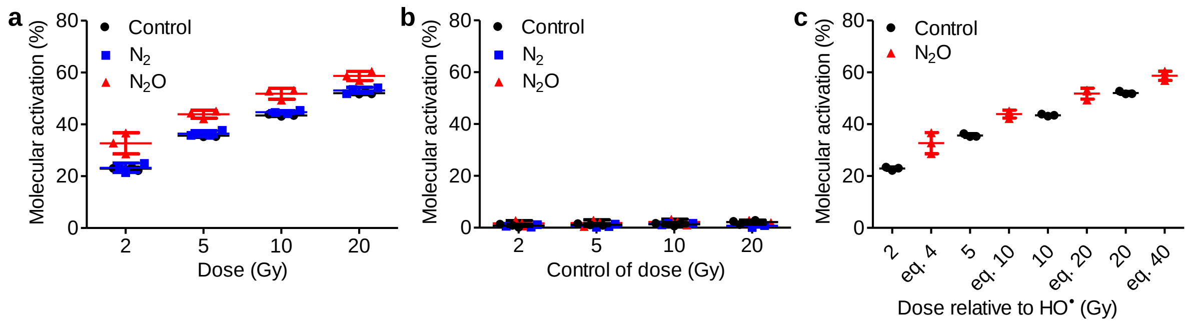

Fig. 57. Molecular activation of *cis*-GdAzo upon gamma-ray radiation under N_2 and N_2O gas saturation. a, **b**, Molecular activation of *cis*-**GdAzo** (50 µM, H_2O) upon gamma rays (**a**) or no irradiation (**b**) in N_2 or N_2O saturated solutions (Control without inerting) determined by HPLC (method C) and reported as the difference of *trans*-isomer proportion before and after gamma-ray irradiation (n=3). The means ± standard deviations are reported. **c**, Comparison of *cis*-**GdAzo** activations in control (no inerting) and N_2O saturated solution in conjunction with the equivalent (eq.) amount of hydroxyl radicals generated depending on doses. N_2O saturation generates two times more hydroxyl radicals at the same dose, thus "eq 4", "eq 10", "eq 20" and "eq 40" relate to the doses of 2, 5, 10 and 20 Gy in N_2O saturated solutions (n=3). The means ± standard deviations are reported.

8.2 – *cis*-GdAzo activation using Fenton chemistry

Activation monitoring by absorbance

cis-**GdAzo** (541.7 µL, final concentration 50 µM, H_2O), EDTA (4.88 µL, final concentration 75 µM, H_2O) and $FeCl_2$ (4.88 µL, final concentration 75 µM, H_2O) were introduced in an 1.5 mL Eppedorf tube (final volume 650 µL, H_2O). The Fenton reaction was started by the addition of H_2O_2 (2.56 µL, final concentration 50 mM, H_2O) into the mixture which was stirred (orbital) in the dark, and the absorbance was acquired 5 min after H_2O_2 addition (96-well microplate, 200 µL/well). Two control mixtures were used: (i) introduction of *cis*-**GdAzo** and H_2O_2 only and (ii) introduction of *cis*-**GdAzo** only. The absorbance values at 365 nm were reported from a mean of triplicate. The experiment was repeated 3 times independently. *cis*-**GdAzo** was obtained by UV irradiation (365 nm, 0.817 mW.cm^{-2}, 10 min) of *trans*-**GdAzo** in 96-well microplate (200 µL, 60 µM, H_2O).

Activation monitoring by HPLC

cis-**GdAzo** (1.000 mL, final concentration 50 µM, H_2O), EDTA (9.00 µL, final concentration 75 µM, H_2O) and $FeCl_2$ (9.00 µL, final concentration 75 µM, H_2O) were introduced in an 1.5 mL Eppedorf tube (final volume 1.200 mL). A first HPLC analysis was run before the introduction of H_2O_2. The Fenton reaction was started by the addition of H_2O_2 (4.41 µL, final concentration 50 mM, H_2O) into the mixture which was stirred (orbital) in the dark. The proportion of *trans*-**GdAzo** was determined by HPLC (method C) 5 min after the addition of H_2O_2. The control experiment was carried out by introducing *cis*-**GdAzo** only into the mixture. The experiment was repeated 3 times independently. *cis*-**GdAzo** was obtained by UV irradiation (365 nm, 0.817 $mW.cm^{-2}$, 10 min) of *trans*-**GdAzo** in 96-well microplate (200 µL, 60 µM, *cis*-**GdAzo** 83% and 94% in EDTA/$FeCl_2$/H_2O_2/H_2O mixture and H_2O only respectively).

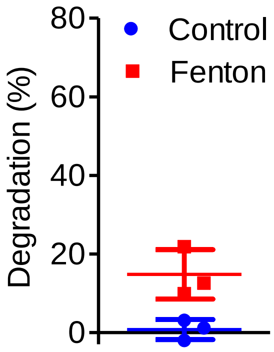

Fig. 58. Degradation *cis*-GdAzo by Fenton-reaction conditions. Assessment of *cis*-**GdAzo** degradation in the dark 5 min after running Fenton reaction determined by HPLC (method C). A control experiment was carried out with *cis*-**GdAzo** in water only (Control). The experiment was repeated 3 times independently. The degradation was determined from the formulae: (signal area after UV irradiation - signal area after ionising radiation)/(signal area after UV irradiation)x100 with the signal area corresponding to the addition of the *cis* and *trans*-isomer signal areas. A positive value represents a loss of compound. The data are represented as the means ± standard deviations. A degradation of *cis*-**GdAzo** was observed using Fenton-reaction conditions.

Supplementary Section 9

Investigation of activation mechanism and role of Gd

Same equipment as described in the Supplementary Section 5 was used. Coumarine (Coum), 7-hydroxycoumarin (7-OH-Coum) and gadolinium(III) chloride hexahydrate ($GdCl_3.6H_2O$) were purchased from Sigma Aldrich (France).

9.1 – Quantification of hydroxyl radicals and role of Gd

9.1.a - Description of the method used for hydroxyl radical quantification

Indirect quantification of hydroxyl radicals ($HO^\bullet$) was based on $HO^\bullet$ scavenging by coumarin (Coum)[9] and quantification of 7-hydroxycoumarin (7-OH-Coum) which is the only fluorescent product released from this scavenging reaction[10]. 7-OH-Coum production proved to be proportional to $HO^\bullet$ concentration and specific for $HO^\bullet$ among other reactive oxygen species[11]. This quantification method seems to be robust and adaptable[12,13]. Nevertheless, it has to be noted that this is an indirect method of quantification which can only give access to the amount of $HO^\bullet$ available to react with Coum in our experimental conditions (temperature, concentrations, etc.). In the conditions used, Coum reacts with $HO^\bullet$ ~10^{-7} s after the initial transfer of energy to water (rate constant of this scavenging reaction is $k = 1.05*10^{10}$ $L.mol^{-1}.s^{-1}$)[14] which results in quantification of the homogeneously distributed $HO^\bullet$ without interfering on the intratrack recombination of radicals occurring in the spurs. The radiochemical yields (*G*-values) of $HO^\bullet$ determined in our study (Fig. 63) confirmed a conversion yield of Coum into 7-OH-Coum of 3.1%, which is in line with the values reported in literature (3.1% reported in ref[11]), with $G(HO^\bullet)$ of 0.200 and 0.280 µmol/J upon X-ray and gamma-ray irradiations respectively[15,16]. The concentration of Coum was considered as constant during the experiment and the radiation dose was considered to not be impacted by the presence of Gd^{3+} in the solutions.

9.1.b - Protocol

Coum (600 µL, final concentration 0.5 mM, H_2O) and $GdCl_3$ (0-168 µL, final concentrations from 0 to 2000 µM, H_2O) were mixed and introduced in a 96-well microplate (200 µL, H_2O, duplicate). The microplate was irradiated upon incremental doses of ionising radiations (final doses of 0, 2, 5, 10, 15, 20, 25, 30 and 40 Gy). After each irradiation, fluorescence (ex: 355 nm, em: 460 nm) was measured. The experiment was repeated 5 times independently. The standard curve of 7-OH-Coum (Fig. 59) led to quantify the production of 7-OH-Coum by increasing the radiation dose at different Gd^{3+} concentrations (Fig 60, 61). The slope of these lines (related to *G*(7-OH-Coum)) were plotted against the Gd^{3+} concentrations to assess the impact of Gd^{3+} on $HO^{\bullet}$ production (Fig. 62). Finally, *G*($HO^{\bullet}$) was determined by applying a conversion yield of Coum into 7-OH-Coum of 3.1% (Fig. 63).

9.1.c - Results

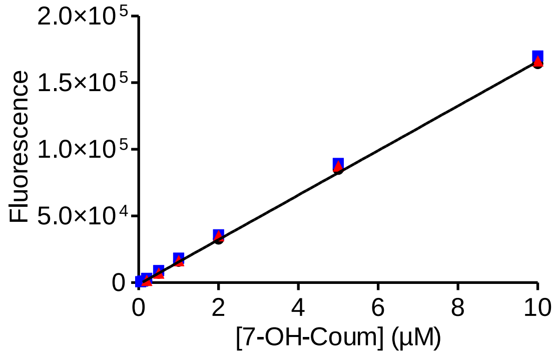

Fig. 59. Standard curve of 7-OH-Coum. Fluorescence (ex: 355 nm, em: 460 nm) of 7-OH-Coum from 0 to 10 µM (9 data points) in water in the presence of Coum (0.5 mM). Representation of three independent experiments. Linear regressions (least-squares method, r^2: 0.9993) resulted in the relation Y = 16943(X) – 530.15 (Excel 2016).

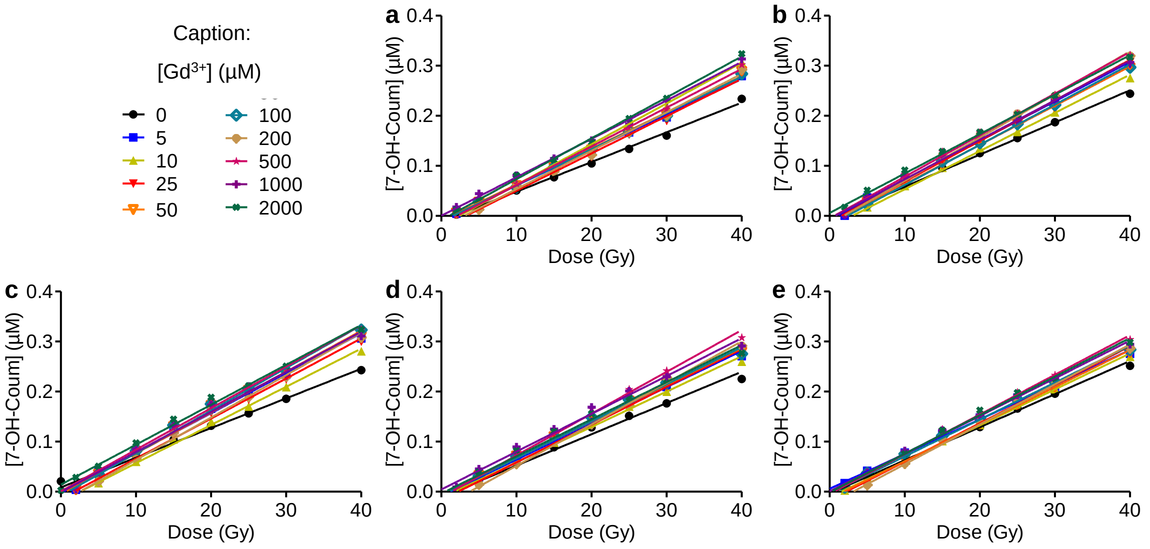

Fig. 60. Production of 7-OH-Coum upon X-ray radiation. Determination of 7-OH-Coum concentration by fluorescence (ex: 355 nm, em: 460 nm) in the presence of different amounts of Gd^{3+} and upon incremental doses of X-rays. **a**, Caption of the graphs. **b-e**, Representation of five independent experiments.

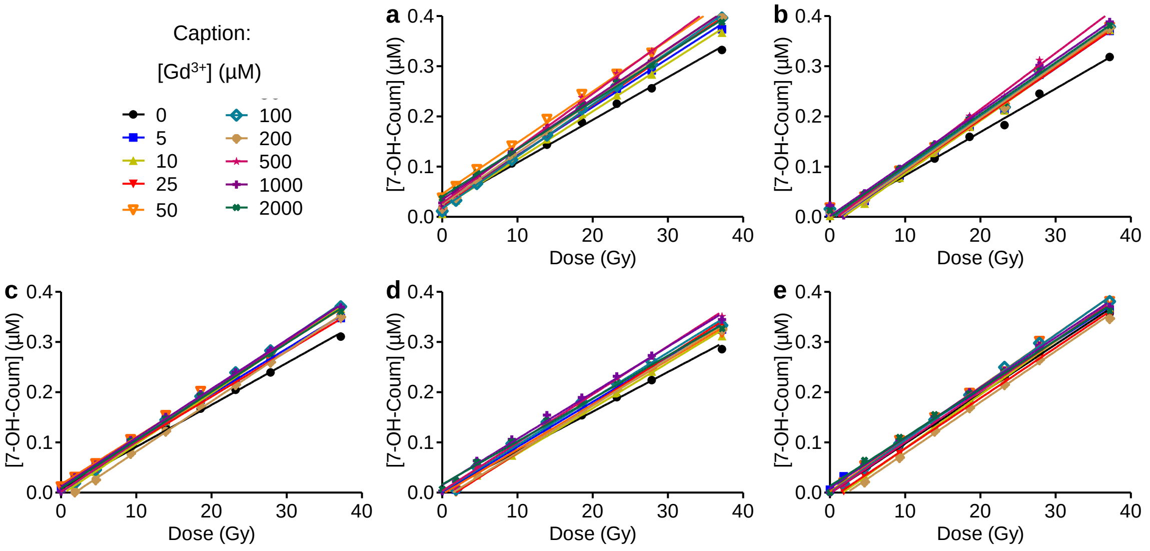

Fig. 61. Production of 7-OH-Coum upon gamma-ray radiation. Determination of 7-OH-Coum concentration by fluorescence (ex: 355 nm, em: 460 nm) in the presence of different amounts of Gd^{3+} and upon incremental doses of gamma-rays. **a**, Caption of the graphs. **b-e**, Representation of five independent experiments.

a

$[Gd^{3+}]$ (µM)	*G* (7-HO-Coum) (nmol/J)						
	Exp 1	Exp 2	Exp 3	Exp 4	Exp 5	Mean	SD
0	5.9	6.4	5.9	6.2	6.6	6.2	0.3
5	7.1	7.9	8.0	7.2	6.9	7.4	0.4
10	8.1	7.6	7.6	7.1	7.2	7.5	0.4
25	7.5	8.1	8.0	7.6	7.7	7.8	0.2
50	7.2	8.0	8.2	7.3	7.4	7.6	0.4
100	7.2	8.0	8.5	7.4	7.4	7.7	0.5
200	7.7	8.6	8.5	8.3	8.0	8.2	0.3
500	7.7	8.3	8.3	8.3	7.9	8.1	0.3
1000	7.7	7.9	8.0	7.6	7.5	7.7	0.2
2000	8.2	7.9	8.0	7.4	7.7	7.8	0.3

c

$[Gd^{3+}]$ (µM)	*G* (7-HO-Coum) (nmol/J)						
	Exp 1	Exp 2	Exp 3	Exp 4	Exp 5	Mean	SD
0	8.5	8.7	8.4	7.9	9.9	8.7	0.7
5	9.6	10.3	9.1	9.0	9.7	9.5	0.5
10	9.6	10.5	10.1	9.1	10.7	10.0	0.6
25	10.1	10.2	9.0	9.6	10.0	9.8	0.4
50	10.2	10.1	9.4	8.9	10.4	9.8	0.6
100	10.4	10.3	10.1	9.2	10.5	10.1	0.5
200	10.3	10.4	10.0	9.3	10.2	10.0	0.4
500	10.9	11.2	10.0	9.6	10.2	10.4	0.6
1000	10.0	10.4	9.8	9.2	10.0	9.9	0.4
2000	9.5	10.3	9.7	8.6	9.7	9.6	0.6

b

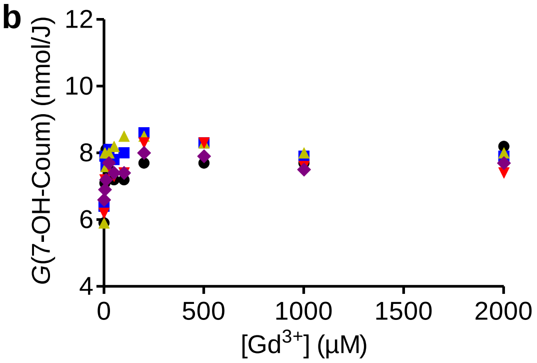

d

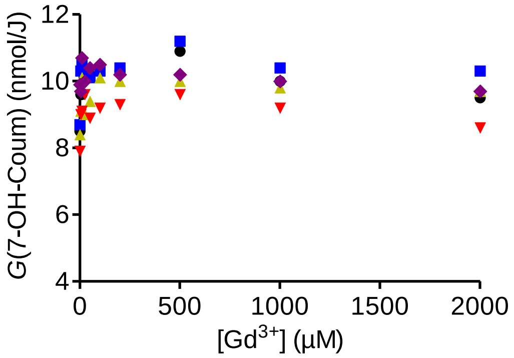

Fig. 62. Determination of *G*(7-OH-Coum) at different concentrations of Gd^{3+}. Tables (**a**, **c**) and corresponding graphs (**b**, **d**) of the 7-OH-Coum production upon X-ray (**a**, **b**) and gamma-ray (**c**, **d**) in the presence of different concentrations of Gd^{3+} (slopes of the lines from Figs. 60, 61). Five independent experiments are represented.

a

[Gd^{3+}] (µM)	*G* (HO•) (nmol/J) Exp 1	Exp 2	Exp 3	Exp 4	Exp 5	Mean	SD
0	190	206	190	200	213	200	9
5	229	255	258	232	223	239	14
10	261	245	245	229	232	243	11
25	242	261	258	245	248	251	7
50	232	252	265	235	239	245	12
100	232	258	274	239	239	248	16
200	248	277	274	268	258	265	11
500	248	268	268	268	255	261	8
1000	248	255	258	245	242	250	6
2000	265	255	258	239	248	253	9

c

[Gd^{3+}] (µM)	*G* (HO•) (nmol/J) Exp 1	Exp 2	Exp 3	Exp 4	Exp 5	Mean	SD
0	274	281	271	255	319	280	21
5	310	332	294	290	313	308	15
10	310	339	326	294	345	323	19
25	326	329	290	310	323	315	14
50	329	326	303	287	335	316	18
100	335	332	326	297	339	326	15
200	332	335	323	300	329	324	13
500	352	361	323	310	329	335	19
1000	323	335	316	297	323	319	13
2000	306	332	313	277	313	308	18

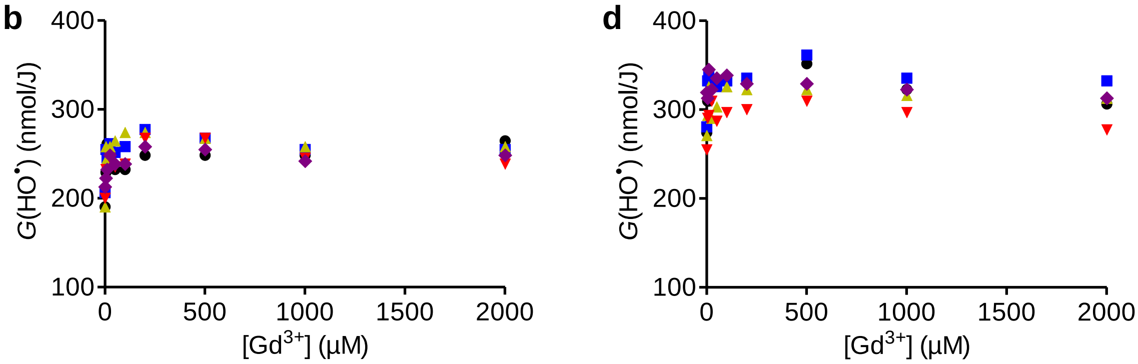

Fig. 63. Determination of *G*(HO•) at different concentrations of Gd^{3+}. Tables (**a**, **c**) and corresponding graphs (**b**, **d**) of the HO• production upon X-ray (**a**, **b**) and gamma-ray (**c**, **d**) in the presence of different concentrations of Gd^{3+} by considering a conversion yield of Coum into 7-OH-Coum of 3.1%. Radiochemical yields *G*(HO•) of 200 and 280 nmol/J upon X-ray and gamma-ray irradiations respectively were obtained without Gd^{3+}, as expected from literature[15,16]. Maximum enhancement factors of 33% ((265-200)/200) at 200 µM [Gd^{3+}] and 20% ((335-280)/280) at 500 µM [Gd^{3+}] were observed upon X-ray and gamma-ray irradiations respectively. Five independent experiments are represented.

9.2 – Impact of Gd on *cis*-Azo activation upon ionising radiations

cis-**GdAzo** or *cis*-**Azo** (final concentration 50 µM, H_2O) and $GdCl_3$ (final concentrations of 0, 20, 50, 200, 500 or 2000 µM, H_2O) were introduced in a 96-well microplate (200 µL, H_2O, triplicate). The microplate was irradiated upon incremental doses of ionising radiations (2, 3, 5, 10 Gy). Before and after each irradiation, absorbance was measured (Fig. 64). The full experiment was performed in about 1.2 h. *cis*-**GdAzo** or *cis*-**Azo** were obtained by UV

irradiation (365 nm, 0.817 mW.cm^{-2}, 5 min) of the *trans*-isomers in a 96-well microplate (200 µL/well). The experiment was repeated 3 times independently.

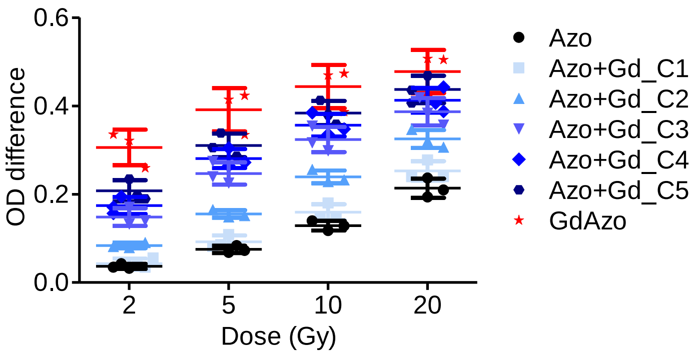

Fig. 64. Impact of the presence of Gd^{3+} for *cis*-Azo activation upon gamma-ray radiation. Absorbance difference (365 nm) of medium containing *cis*-**GdAzo** or *cis*-**Azo** (50 µM, H_2O) in the presence of Gd^{3+} (20, 50, 200, 500 and 2000 µM for C1 to C5 respectively, H_2O) before and after gamma-ray irradiation (triplicate). The experiment was repeated three times independently. The data are represented as the means ± standard deviations.

Supplementary Section 10

Investigation of activation mechanism by variation of GdAzo concentration

Same equipment as described in the Supplementary Section 5 was used.

Quantification of *cis*-GdAzo activation upon gamma-ray radiations at different GdAzo concentrations

The gadolinium-containing compound (*trans*-**GdAzo**) and the control compound (*trans*-**Azo**) (20, 200, 500 and 1000 µM, 200 µL, PBS) were introduced in two 96-well microplates (8 wells for each compound). Both microplates were irradiated by UV (365 nm, 0.817 mW.cm^{-2}, 15 min) to obtain the photostationary state 1 (PSS1) containing a majority of the *cis*-isomer (88 ± 4%). One microplate (plate 1) was kept in the dark and used as control (non-irradiated by ionising radiation) whereas the second microplate (plate 2) was irradiated upon incremental doses of ionising radiations (2, 3, 5 and 10 Gy). After each irradiation, HPLC (method C) analyses were performed on the non-irradiated (plate 1) and irradiated (plate 2) compounds. A delay of 26 min between the UV irradiation of plate 1 and plate 2 was introduced to analyse the non-irradiated control compounds (plate 1) at the same time as the compounds irradiated by ionising radiations (plate 2) after UV irradiation. The full experiment was performed in about 4.5 h. The relative amount of each isomer was obtained by running HPLC (method C) at the isobestic-point conditions (mobile phase and detection wavelength) and the molecular activation (%) was determined by the difference in proportion of the *trans*-isomer in the media (Fig. 65). The experiments were repeated 3 times independently.

a	No irradition (plate 1)						gamma-ray irradiation (plate 2)					
	GdAzo			Azo			GdAzo			Azo		
**Exp 1**	*trans* area	*cis* area	*trans* (%)	*trans* area	*cis* area	*trans* (%)	*trans* area	*cis* area	*trans* (%)	*trans* area	*cis* area	*trans* (%)
UV	314	2601	10,8	298	1956	13,2	327	2671	10,9	289	1814	13,7
2 Gy	403	2007	16,7	442	1836	19,4	1105	1336	45,3	433	1752	19,8
5Gy	500	1828	21,5	654	1834	26,3	1422	758	65,2	633	1683	27,3
10Gy	550	1702	24,4	558	1616	25,7	1923	637	75,1	774	1483	34,3
20Gy	745	1732	30,1	765	1613	32,2	1699	344	83,2	786	1186	39,9
**Exp 2**	*trans* area	*cis* area	*trans* (%)	*trans* area	*cis* area	*trans* (%)	*trans* area	*cis* area	*trans* (%)	*trans* area	*cis* area	*trans* (%)
UV	314	2255	12,2	360	2072	14,8	295	2166	12,0	359	2005	15,2
2 Gy	476	2122	18,3	484	1941	20,0	1192	1202	49,8	500	1844	21,3
5Gy	522	1980	20,9	476	1720	21,7	1531	617	71,3	489	1549	24,0
10Gy	566	1761	24,3	545	1638	25,0	1553	394	79,8	658	1479	30,8
20Gy	684	1767	27,9	588	1572	27,2	1835	201	90,1	676	1184	36,3
**Exp 3**	*trans* area	*cis* area	*trans* (%)	*trans* area	*cis* area	*trans* (%)	*trans* area	*cis* area	*trans* (%)	*trans* area	*cis* area	*trans* (%)
UV	262	2306	10,2	265	1818	12,7	228	2440	8,5	328	2452	11,8
2 Gy	332	1961	14,5	357	1984	15,2	907	1111	44,9	479	2231	17,7
5Gy	414	1788	18,8	456	1681	21,3	1492	696	68,2	451	1625	21,7
10Gy	611	1883	24,5	616	1759	25,9	1742	416	80,7	571	1551	26,9
20Gy	636	1672	27,6	693	1680	29,2	1771	240	88,1	659	1225	35,0

b	No irradition (plate 1)						gamma-ray irradiation (plate 2)					
	GdAzo			Azo			GdAzo			Azo		
**Exp 1**	*trans* area	*cis* area	*trans* (%)	*trans* area	*cis* area	*trans* (%)	*trans* area	*cis* area	*trans* (%)	*trans* area	*cis* area	*trans* (%)
UV	516	4818	9,7	267	3722	6,7	475	4584	9,4	272	3394	7,4
2 Gy	621	4179	12,9	401	3495	10,3	2188	2809	43,8	399	3338	10,7
5Gy	748	4129	15,3	432	3245	11,7	2653	1710	60,8	599	3300	15,4
10Gy	1038	3983	20,7	745	3230	18,7	2807	1221	69,7	860	2800	23,5
20Gy	1179	4080	22,4	832	3068	21,3	3389	1098	75,5	1010	2518	28,6
**Exp 2**	*trans* area	*cis* area	*trans* (%)	*trans* area	*cis* area	*trans* (%)	*trans* area	*cis* area	*trans* (%)	*trans* area	*cis* area	*trans* (%)
UV	569	4378	11,5	307	3594	7,9	477	4331	9,9	258	3443	7,0
2 Gy	686	4388	13,5	448	3235	12,2	2059	2694	43,3	339	3347	9,2
5Gy	852	4387	16,3	627	3100	16,8	2907	1924	60,2	793	3243	19,6
10Gy	1169	4103	22,2	638	3107	17,0	3436	1343	71,9	793	2997	20,9
20Gy	1145	3745	23,4	808	3126	20,5	3591	1022	77,8	1050	2465	29,9
**Exp 3**	*trans* area	*cis* area	*trans* (%)	*trans* area	*cis* area	*trans* (%)	*trans* area	*cis* area	*trans* (%)	*trans* area	*cis* area	*trans* (%)
UV	648	5804	10,0	631	6105	9,4	541	5746	8,6	659	5973	9,9
2 Gy	760	5461	12,2	823	5878	12,3	2420	3475	41,1	905	5592	13,9
5Gy	925	5187	15,1	1047	5541	15,9	3261	2391	57,7	1163	5264	18,1
10Gy	1230	4949	19,9	1258	5387	18,9	3994	1399	74,1	1412	4845	22,6
20Gy	1517	4766	24,1	1364	5013	21,4	4063	991	80,4	1710	4429	27,9

c	No irradition (plate 1)						gamma-ray irradiation (plate 2)					
	GdAzo			Azo			GdAzo			Azo		
**Exp 1**	*trans* area	*cis* area	*trans* (%)	*trans* area	*cis* area	*trans* (%)	*trans* area	*cis* area	*trans* (%)	*trans* area	*cis* area	*trans* (%)
UV	2784	24525	10,2	2737	24516	10,0	2690	24442	9,9	2816	24557	10,3
2 Gy	4442	27093	14,1	3494	21408	14,0	10377	16973	37,9	3825	21196	15,3
5Gy	5563	25854	17,7	4492	20831	17,7	14335	12115	54,2	5321	21678	19,7
10Gy	5799	20615	22,0	5558	21574	20,5	17720	9527	65,0	6842	20115	25,4
20Gy	7186	20828	25,7	6607	20153	24,7	16504	6022	73,3	7618	17396	30,5
**Exp 2**	*trans* area	*cis* area	*trans* (%)	*trans* area	*cis* area	*trans* (%)	*trans* area	*cis* area	*trans* (%)	*trans* area	*cis* area	*trans* (%)
UV	2948	25697	10,3	2733	23638	10,4	2654	24157	9,9	2984	24584	10,8
2 Gy	4157	23724	14,9	3739	22680	14,2	10480	17803	37,1	4026	21806	15,6
5Gy	5228	22483	18,9	4817	22067	17,9	15032	12950	53,7	5503	22480	19,7
10Gy	6452	22050	22,6	5605	20472	21,5	15363	8476	64,4	6386	19514	24,7
20Gy	6697	19627	25,4	6790	21054	24,4	18747	6902	73,1	7693	17683	30,3
**Exp 3**	*trans* area	*cis* area	*trans* (%)	*trans* area	*cis* area	*trans* (%)	*trans* area	*cis* area	*trans* (%)	*trans* area	*cis* area	*trans* (%)
UV	2800	23542	10,6	2752	24003	10,3	2663	23782	10,1	2938	23959	10,9
2 Gy	3981	22578	15,0	3945	23593	14,3	9428	16553	36,3	3973	23235	14,6
5Gy	5205	21805	19,3	4576	21311	17,7	12653	11137	53,2	5009	20489	19,6
10Gy	6329	20737	23,4	5510	20351	21,3	17245	9749	63,9	6667	20519	24,5
20Gy	7000	19440	26,5	5776	17327	25,0	18601	7067	72,5	8329	18661	30,9

Table 5. Proportion of *trans*-Azo and *trans*-GdAzo upon gamma-ray radiations determined by HPLC. Assessment of *cis*-**Azo** and *cis*-**GdAzo** isomerisation upon gamma-rays at concentrations of 20 µM (**a**), 50 µM (**b**) and 200 µM (**c**). The data from the experiments without ionising radiation (control experiment, plate 1) and from the experiments upon ionising radiations (plate 2) are reported on the left and on the right respectively. Three independent experiments are reported (Exp 1, 2 and 3). The peak areas of both isomers were measured by HPLC (method C) after UV and gamma-ray radiations. The proportions of the *trans*-isomer were determined by the area ratios.

**a**	No irradition (plate 1)						gamma-ray irradiation (plate 2)					
	GdAzo			Azo			GdAzo			Azo		
**Exp 1**	*trans* area	*cis* area	*trans* (%)	*trans* area	*cis* area	*trans* (%)	*trans* area	*cis* area	*trans* (%)	*trans* area	*cis* area	*trans* (%)
UV	2286	22705	9,1	2178	21310	9,3	2002	19976	9,1	2165	19824	9,8
2 Gy	3081	19690	13,5	3275	20916	13,5	9940	18957	34,4	3439	20892	14,1
5Gy	4232	19633	17,7	4019	19253	17,3	12319	11927	50,8	4422	20075	18,1
10Gy	5260	19752	21,0	5073	19240	20,9	14471	8986	61,7	5583	17831	23,8
20Gy	6208	19177	24,5	6152	19363	24,1	15174	6228	70,9	6871	17501	28,2
**Exp 2**	*trans* area	*cis* area	*trans* (%)	*trans* area	*cis* area	*trans* (%)	*trans* area	*cis* area	*trans* (%)	*trans* area	*cis* area	*trans* (%)
UV	2084	20786	9,1	2057	21015	8,9	2027	20512	9,0	1920	19810	8,8
2 Gy	2911	19532	13,0	2975	20053	12,9	7973	14255	35,9	3129	20208	13,4
5Gy	4070	19645	17,2	3849	19672	16,4	11369	10528	51,9	3886	18690	17,2
10Gy	4725	18337	20,5	4467	18253	19,7	14419	8477	63,0	4626	16484	21,9
20Gy	5501	17724	23,7	5509	18175	23,3	15847	6231	71,8	6019	15675	27,7
**Exp 3**	*trans* area	*cis* area	*trans* (%)	*trans* area	*cis* area	*trans* (%)	*trans* area	*cis* area	*trans* (%)	*trans* area	*cis* area	*trans* (%)
UV	1687	16678	9,2	1827	18169	9,1	2063	19986	9,4	2274	20852	9,8
2 Gy	3215	20111	13,8	2970	19390	13,3	7814	14248	35,4	3104	19038	14,0
5Gy	3747	17765	17,4	3912	19259	16,9	11446	10525	52,1	3959	17717	18,3
10Gy	4950	18220	21,4	4484	17847	20,1	13476	8025	62,7	5229	17563	22,9
20Gy	5548	16784	24,8	5616	17732	24,1	15714	6265	71,5	6296	15703	28,6

**b**	No irradition (plate 1)						gamma-ray irradiation (plate 2)					
	GdAzo			Azo			GdAzo			Azo		
**Exp 1**	*trans* area	*cis* area	*trans* (%)	*trans* area	*cis* area	*trans* (%)	*trans* area	*cis* area	*trans* (%)	*trans* area	*cis* area	*trans* (%)
UV	3964	40870	8,8	3891	43154	8,3	4178	43314	8,8	4039	42088	8,8
2 Gy	5638	38139	12,9	5373	38092	12,4	14940	30973	32,5	5916	39642	13,0
5Gy	7408	36490	16,9	6463	33583	16,1	22033	22934	49,0	7336	35790	17,0
10Gy	9447	36696	20,5	8805	36865	19,3	27282	17469	61,0	9391	34430	21,4
20Gy	10400	33789	23,5	10215	34658	22,8	30951	12915	70,6	12183	33460	26,7
**Exp 2**	*trans* area	*cis* area	*trans* (%)	*trans* area	*cis* area	*trans* (%)	*trans* area	*cis* area	*trans* (%)	*trans* area	*cis* area	*trans* (%)
UV	3910	41817	8,6	3715	39463	8,6	3995	42577	8,6	3895	39816	8,9
2 Gy	5461	37558	12,7	5725	38411	13,0	14547	30383	32,4	5444	34930	13,5
5Gy	7261	36826	16,5	7390	36739	16,7	21209	22289	48,8	7963	37558	17,5
10Gy	8715	36866	19,1	8260	32245	20,4	26491	17184	60,7	9862	35485	21,7
20Gy	10313	33394	23,6	10067	32062	23,9	30428	12768	70,4	11802	31616	27,2
**Exp 3**	*trans* area	*cis* area	*trans* (%)	*trans* area	*cis* area	*trans* (%)	*trans* area	*cis* area	*trans* (%)	*trans* area	*cis* area	*trans* (%)
UV	4120	43980	8,6	4119	43578	8,6	3102	34978	8,1	3557	36044	9,0
2 Gy	6229	40380	13,4	5854	40127	12,7	12095	27149	30,8	6108	39393	13,4
5Gy	7970	38365	17,2	7625	38370	16,6	21783	24266	47,3	7993	37439	17,6
10Gy	9151	34977	20,7	9245	37299	19,9	26160	17941	59,3	9617	33849	22,1
20Gy	11352	34572	24,7	10799	34967	23,6	30040	13406	69,1	12341	34017	26,6

Table 6. Proportion of *trans*-Azo and *trans*-GdAzo upon gamma-ray radiations determined by HPLC. Assessment of *cis*-**Azo** and *cis*-**GdAzo** isomerisation upon gamma-rays at concentrations of 500 µM (**a**) and 1000 µM (**b**). The data from the experiments without ionising radiation (control experiment, plate 1) and from the experiments upon ionising radiations (plate 2) are reported on the left and on the right respectively. Three independent experiments are reported (Exp 1, 2 and 3). The peak areas of both isomers were measured by HPLC (method C) after UV and gamma-ray radiations. The proportions of the *trans*-isomer were determined by the area ratios.

Compound	Conc (µM)	Gamma-ray radiation				Control without radiation			
		2 Gy	5 Gy	10 Gy	20 Gy	Comp 2 Gy	Comp 5 Gy	Comp 10 Gy	Comp 20 Gy
cis-Azo	20	6.0 ± 0.2	10.8 ± 2.6	17.1 ± 3.1	23.5 ± 2.5	4.6 ± 1.9	9.5 ± 3.2	11.9 ± 1.6	16.0 ± 3.3
	50	3.2 ± 0.9	9.6 ± 2.7	14.2 ± 1.8	20.7 ± 2.6	3.6 ± 0.7	6.9 ± 2.0	10.3 ± 1.6	13.1 ± 1.4
	200	4.5 ± 0.8	9.0 ± 0.4	14.2 ± 0.9	19.9 ± 0.4	3.9 ± 0.2	7.5 ± 0.2	10.9 ± 0.4	14.5 ± 0.4
	500	4.3 ± 0.3	8.3 ± 0.2	13.4 ± 0.6	18.7 ± 0.3	4.1 ± 0.2	7.7 ± 0.3	11.1 ± 0.5	14.7 ± 0.4
	1000	4.4 ± 0.2	8.5 ± 0.2	12.9 ± 0.3	17.9 ± 0.4	4.2 ± 0.2	8.0 ± 0.2	11.3 ± 0.5	14.9 ± 0.5
cis-GdAzo	20	36.2 ± 1.8	57.8 ± 3.0	68.1 ± 4.0	76.6 ± 3.9	5.4 ± 1.1	9.3 ± 1.3	13.3 ± 1.2	17.4 ± 1.9
	50	33.4 ± 1.0	50.3 ± 1.2	62.6 ± 2.7	68.6 ± 2.9	2.5 ± 0.7	5.2 ± 0.5	10.5 ± 0.6	12.9 ± 1.1
	200	27.1 ± 1.0	43.8 ± 0.6	54.5 ± 0.7	63.0 ± 0.6	4.3 ± 0.4	8.2 ± 0.7	12.3 ± 0.5	15.5 ± 0.4
	500	26.1 ± 0.8	42.5 ± 0.7	53.3 ± 0.8	62.2 ± 0.6	4.3 ± 0.4	8.3 ± 0.3	11.8 ± 0.5	15.2 ± 0.6
	1000	23.4 ± 0.7	39.8 ± 0.6	51.8 ± 0.6	61.5 ± 0.5	4.3 ± 0.5	8.2 ± 0.4	11.5 ± 0.9	15.3 ± 0.8

Table 7. Molecular activation of *cis*-Azo and *cis*-GdAzo at different concentrations upon gamma-ray radiation. The molecular activation (%) was determined by HPLC (method C) and represents the increase in proportion of the *trans*-isomer in the media from PSS1 (3 independent experiments). “Comp” refers to the non-irradiated compound analysed concurrently with the irradiated compound at the mentioned dose. The means ± standard deviations are reported. Conc : Concentration.

Compound	Conc (µM)	Gamma-ray radiation			
		2 Gy	5 Gy	10 Gy	20 Gy
cis-Azo	20	0.15 ± 0.20	0.05 ± 0.04	0.11 ± 0.07	0.08 ± 0.02
	50	-0.12 ± 0.43	0.30 ± 0.12	0.21 ± 0.05	0.20 ± 0.06
	200	0.59 ± 0.86	0.63 ± 0.11	0.71 ± 0.26	0.58 ± 0.02
	500	0.57 ± 0.85	0.66 ± 0.40	1.24 ± 0.07	1.07 ± 0.15
	1000	1.25 ± 0.58	1.08 ± 0.33	1.67 ± 0.49	1.63 ± 0.21
cis-GdAzo	20	3.35 ± 0.23	2.10 ± 0.19	1.18 ± 0.09	0.63 ± 0.06
	50	8.42 ± 0.16	4.89 ± 0.11	2.81 ± 0.18	1.50 ± 0.06
	200	24.9 ± 1.3	15.4 ± 0.6	9.11 ± 0.25	5.12 ± 0.09
	500	59.3 ± 3.0	37.0 ± 1.1	22.4 ± 0.6	12.7 ± 0.3
	1000	103.8 ± 5.7	68.6 ± 2.2	43.6 ± 1.4	24.9 ± 0.7

Table 8. *G*-values of *cis*-Azo and *cis*-GdAzo at different concentrations upon gamma-ray radiation. The *G*-value defines the yield of activation upon ionising radiation (µmol/J). It was determined by HPLC (method C) and represents the molar amount of *trans*-**GdAzo** activation corrected from thermal back relaxation (activation due to thermal relaxation determined by control experiment) and divided by the energy received by the medium (3 independent experiments). The means ± standard deviations are reported. Conc : Concentration.

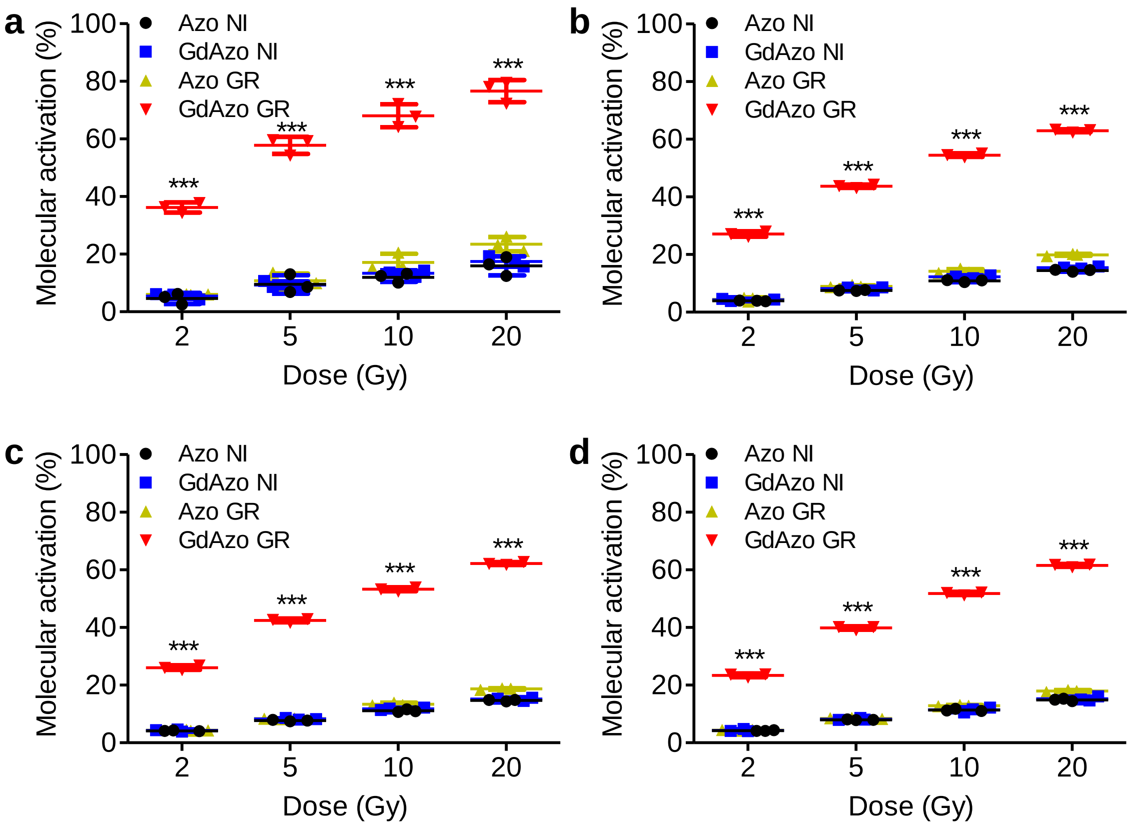

Fig. 65. Molecular activation of *cis*-Azo and *cis*-GdAzo at different concentrations upon gamma-ray radiation. Molecular activation of *cis*-**GdAzo** and *cis*-**Azo** (control molecule without Gd atom) at concentration of 20 µM (**a**), 200 µM (**b**), 500 µM (**c**) and 1000 µM (**d**) determined by HPLC (method C) and reported as the difference of *trans*-isomer proportion before and after gamma-ray (GR) irradiation (n=3). The means ± standard deviations are reported. Two-way Anova (Bonferroni post-test) was used for statistical analyses (All *vs* **GdAzo** GR). For *cis*-**GdAzo** concentration of 20 µM (**a**), *t*-values = 14.62, 23.03, 26.01 and 28.15 for **GdAzo** NI *vs* **GdAzo** GR at doses 2, 5, 10 and 20 Gy. For *cis*-**GdAzo** concentration of 200 µM (**b**), *t*-values = 50.79, 78.93, 93.85 and 105.6 for **GdAzo** NI *vs* **GdAzo** GR at doses 2, 5, 10 and 20 Gy. For *cis*-**GdAzo** concentration of 500 µM (**c**), *t*-values = 52.96, 83.01, 100.8 and 114.3 for **GdAzo** NI *vs* **GdAzo** GR at doses 2, 5, 10 and 20 Gy. For *cis*-**GdAzo** concentration of 1000 µM (**d**), *t*-values = 45.28, 75.12, 95.77 and 109.8 for **GdAzo** NI *vs* **GdAzo** GR at doses 2, 5, 10 and 20 Gy.*** $P < 0.001$. NI: non irradiated.

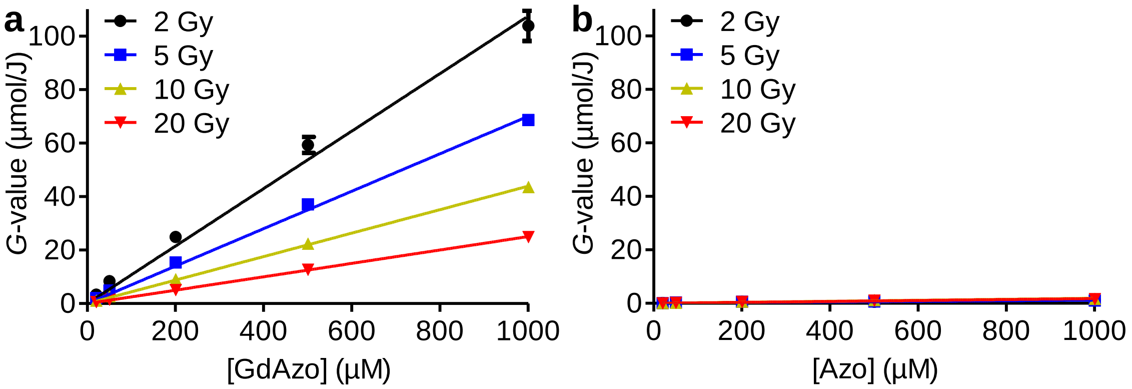

c

GdAzo	2 Gy	5 Gy	10 Gy	20 Gy
Slope	0.1075	0.0700	0.0439	0.0250
r²	0.9905	0.9966	0.9993	0.9997

Azo	2 Gy	5 Gy	10 Gy	20 Gy
Slope	0.0013	0.0012	0.0019	0.0018
r²	0.8464	0.6291	0.8465	0.9269

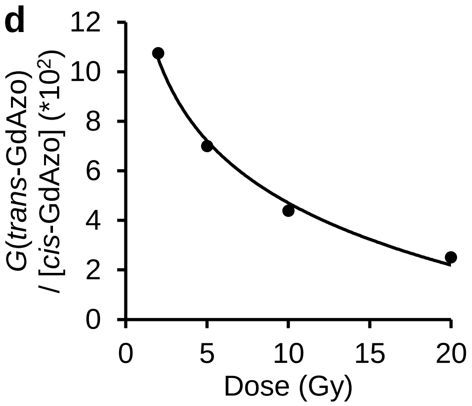

Fig. 66. Relation of *G*-values *vs cis*-GdAzo and *cis*-Azo concentrations upon gamma-ray radiation. a, b, *G*-value (µmol/J) of the activation of *cis*-**GdAzo** (**a**) and *cis*-**Azo** (**b**) upon gamma ray determined by HPLC (method C, molar amount of *trans*-**GdAzo** and *trans*-**Azo** activation corrected from thermal back relaxation) (3 independent experiments). The means ± standard deviations are reported. **c,** Parameters obtained from linear regressions in **a** and **b**, using the least-squares method (Excel 2016). **d,** Linear regression to correlate *G*(*trans*-**GdAzo**)/[*cis*-**GdAzo**] to the irradiation doses. The relation Y = -0.036*ln(X) + 0.1304 (with Y the slope of linear regressions from **a** in the unit *G*-value (µmol/J)/[**GdAzo**] (µM) and X the dose in Gy) was obtained with r^2 = 0.9925 (Excel 2016).

Supplementary Section 11

Physicochemical characterisation

General information

Nile red and phosphate buffered saline (PBS) were purchased from Sigma Aldrich (France).

11.1 - Micelle characterisation by fluorescence labelling

Critical micelle concentration (CMC) was determined using the fluorogenic properties of Nile red. It is well established that Nile red fluorescence increases by several orders of magnitude in lipophilic environment, which enables to detect the presence of micelles in aqueous media[17].

Nile red (stock solution 2 mM in MeOH, final concentration 0.5 µM) was introduced in a solution of *trans*-**GdAzo** (final concentration 0.01-4 mM, PBS) and the mixture was kept in the dark for 30 min. Fluorescence (ex: 585 nm, em 635 nm, quartz cuve, Perkin Elmer LS50B fluorescence spectrometer) was measured and reported *versus trans*-**GdAzo** concentration (Fig. 67). A sharp fluorescence increase was observed once micelles were formed. CMC was determined by the intersection of the two lines in the concentration range 0.01-0.55 mM using linear regressions (least-squares method, GraphPad Prism 5.00). The CMC of *trans*-**GdAzo** compound at rt was 0.29 mM.

Assessment of relaxivity was also used to determine the CMC of *trans*-**GdAzo** at 37 °C (0.42 mM, cf. Supplementary Section 8.2 “Relaxivity measurement”).

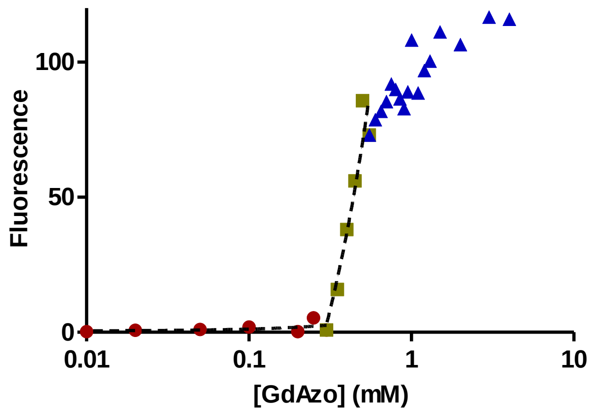

Fig. 67. Determination of CMC by fluorescence labelling. Two linear regressions (dashed lines) were performed: (i) for [**GdAzo**] between 0 and 0.3 mM (slope: 7.067; Y-intercept: 0.4779; r^2: 0.2284) and (ii) for [**GdAzo**] between 0.3 and 0.55 mM (slope: 336.3; Y-intercept: -98.01; r^2: 0.9138) (GraphPad Prism 5.00). The intersection of the two lines was used to calculate the CMC of *trans*-**GdAzo** at rt (0.29 mM). Different colours/symbols were used on the graph to distinguish the slope discontinuities.

11.2 - Relaxivity measurement

Water-proton relaxation measurements were performed on solutions with a concentration of *trans*-**GdAzo** ranging from 0.025 to 4 mM in PBS. A Bruker Minispec mq60 relaxometer (Bruker, Wissembourg, France) was used to determine the longitudinal relaxivity (r_1) of *trans*-**GdAzo** at 60 MHz (1.41 T), 37 °C (Fig. 68). Spin-lattice relaxation times (T_1) were measured by inversion-recovery method. r_1 was calculated from the slope of the regression line of $1/T_1$ *versus* the *trans*-**GdAzo** concentration (least-squares method, GraphPad Prism 5.00). The CMC of *trans*-**GdAzo** compound at 37 °C was 0.42 mM and r_1 of the monomer and micelle were 3.9 and 14.6 $mM^{-1}.s^{-1}.Gd^{-1}$ respectively.

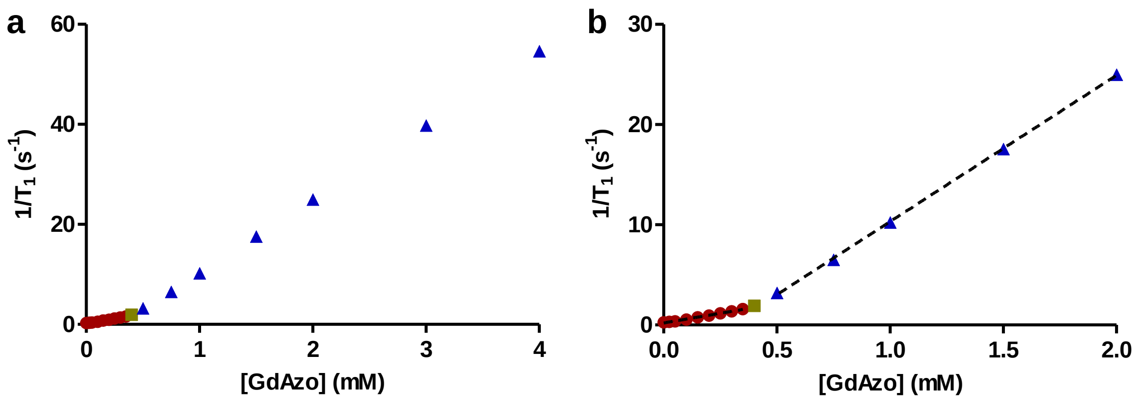

Fig. 68. Determination of the longitudinal relaxivity at 37 °C and 1.41 T. **a**, Determination of the longitudinal relaxivity (r_1) of *trans*-**GdAzo** for a concentration range of 0.025-4 mM in PBS. A slope discontinuity was detected which confirmed the aggregation of *trans*-**GdAzo** into micelles. **b**, This graph is an enlargement of the graph (**a**). Two linear regressions (dashed lines) were performed: (i) for [**GdAzo**] between 0.025 and 0.35 mM (slope: 3.864; Y-intercept: 0.1998; r^2: 0.9956) and (ii) for [**GdAzo**] between 0.5 and 2 mM (slope: 14.60; Y-intercept: -4.291; r^2: 0.9998) (GraphPad Prism 5.00). The intersection of the two lines was used to calculate the CMC of *trans*-**GdAzo** at 37 °C (0.42 mM). r_1 of the monomer and micelle were 3.9 and 14.6 $mM^{-1}.s^{-1}.Gd^{-1}$ respectively. Different colours/symbols were used on the graphs to distinguish the slope discontinuities.

Supplementary Section 12

Computational details for electronic structure calculation

General information

All calculations were performed in the framework of density functional theory (DFT)[18,19] with Becke's three-parameter Lee–Yang–Parr exchange-correlation functional (B3LYP)[20,21] using Stuttgart/Dresden (SDD) effective core potentials (ECP) with the corresponding basis set for gadolinium[22] and 6-31G* basis set for all other atoms[23], as implemented in the Gaussian 16 revision B.01 software package[24]. Ground state geometries were freely optimised and vibrational analysis within the harmonic approximation was performed at the same level of theory upon convergence to check for the absence of imaginary frequency. Geometry optimisations were carried out using polarisable continuum model (PCM) parametrised for water[25].

12.1 - Structures of GdAzo derivatives and local surface polarities

The calculations were carried out for the **GdAzo** compounds (Table 3) considering 8 diastereoisomers: (i) 2 geometries (2 couples of 2 enantiomers) from the Gd-chelate moiety (square antiprismatic (SAP) and twisted square antiprismatic (TSAP) isomers), (ii) 2 configurations from the stereogenic centre on the quaternary carbon (*R* and *S*) and (iii) 2 configurations from the azo double bond (*cis* and *trans*). Molecular modelling figures were rendered using UCSF Chimera version 1.14 (Figs. 69a, b)[26]. An in-house script was used to classify and display polar and non-polar surfaces, which were derived from electrostatic potential-mapped total electron density isodensity surfaces, using 0.01 a.u. as density value, as well as −0.028 and +0.115 a.u. as negative and positive potential thresholds respectively, as suggested by reference (Fig. 69c)[27].

Diastereoisomer	Azobenzene isomer	Gd-chelate isomer	Quaternary carbon isomer	Free enthalpy (Ha)	Φ_1 angle (°)	CNNC dihedral angle (°)	CCNN dihedral angle (°)	Φ_2 angle (°)
1	*trans*	SAP	R	−3415.651178	37.3 ± 0.2	179.866	0.361	0.544
2	*cis*	SAP	R	−3415.628616	37.11 ± 0.09	11.481	45.233	57.359
3	*trans*	TSAP	R	−3415.649787	23 ± 1	179.871	0.458	0.721
4	*cis*	TSAP	R	−3415.62608	23.0 ± 0.9	11.587	44.219	56.669
5	*trans*	SAP	S	−3415.643274	36.6 ± 0.4	179.648	0.205	0.098
6	*cis*	SAP	S	−3415.618862	36.6 ± 0.4	11.645	43.625	56.381
7	*trans*	TSAP	S	−3415.651824	24.4 ± 0.6	179.988	0.397	0.796
8	*cis*	TSAP	S	−3415.627509	24.2 ± 0.6	11.677	43.825	56.542

Table 9. Free enthalpies and geometrical parameters of *cis*-GdAzo and *trans*-GdAzo at the B3LYP/6-31G*~SDD level. Φ_1 angle is the absolute value of the dihedral angle between the parallel squares defined by the nitrogen and acetate oxygen (bound to Gd) atoms of the Gd-chelate used for the identification of SAP and TSAP isomers (means of 4 angles ± standard deviations are reported).[28] CNNC dihedral angle was measured between the two C-N bounds from either side of the azo N=N bound (absolute value). CCNN dihedral angle was measured between the CC bound (in the aryl cycle substituted by the *para*-butoxy group) next to the azo N=N bound and the azo N=N bound (absolute value). Φ_2 angle is the absolute value of the dihedral angle between the two aryl cycles of the azobenzene core. The atomic coordinates of the 8 diastereoisomers are included at the end of this section.

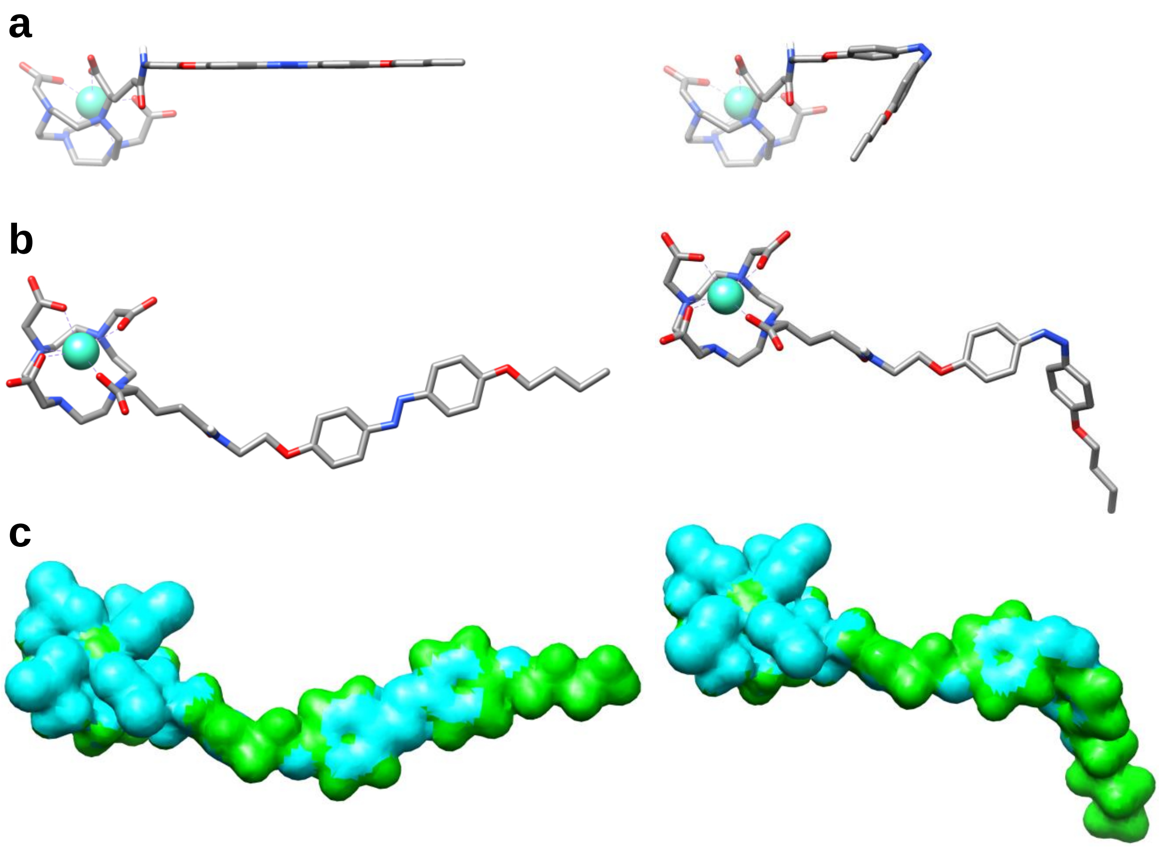

Fig. 69. *trans*-GdAzo and *cis*-GdAzo at the B3LYP/6-31G*~SDD level. a, **b**, Molecular modelling representations of *trans*-**GdAzo** (left) and *cis*-**GdAzo** (right) in different orientations. **c**, Representation of the polar (blue) and non-polar (green) surfaces of *trans*-**GdAzo** and *cis*-**GdAzo** from the orientation in (**b**). UCSF Chimera software (version 1.14) and an in-house script were used.

12.2 - Dipole moments

A set of diverse conformations of the isolated linear azo-containing moiety (**Azo-tail**) as well as of an equivalent model for a reference system (**Azo-reference**)[29,30] were geometrically optimised at the same level of theory using continuum solvation model parametrised for water, in order to compare average dipole moments of the *cis* and *trans*-isomer at 310.15 K (Fig. 70). For the **Azo-tail** model, a decrease of the dipole moment by 2.62 D upon *cis*-*trans* isomerisation was found (Table 4), whereas the equivalent value was 2.05 D for the **Azo-reference** model (Table 5).

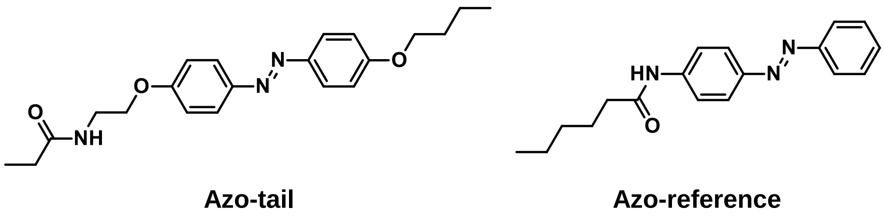

Fig. 70. Molecular structures used for the calculations of dipole moments. The linear azo-containing moieties **Azo-tail** and **Azo-reference** were extracted from the **GdAzo** structure and a polymer previously described in literature for cell-membrane permeabilisation[29,30] respectively.

Isomer	Conformation	Dipole moment (D)	Free enthalpy (Ha)	Lowest frequency (cm^{-1})	Relative free enthalpy (kcal/mol)	Population (%)
trans	1	6.13	−1206.028915	11.6964	0.00	24.1
trans	2	4.49	−1206.028537	10.5141	0.24	16.2
trans	3	4.48	−1206.028497	10.2930	0.26	15.5
trans	4	5.96	−1206.028229	11.4144	0.43	11.7
trans	5	6.07	−1206.027953	13.5452	0.60	8.7
trans	6	4.53	−1206.027933	10.8830	0.62	8.5
trans	7	5.90	−1206.027893	14.2539	0.64	8.2
trans	8	4.49	−1206.027772	10.0647	0.72	7.2
cis	1	8.32	−1206.005532	6.8621	0.00	11.7
cis	2	7.07	−1206.005503	6.3230	0.02	11.4
cis	3	10.21	−1206.005415	8.4285	0.07	10.4
cis	4	8.49	−1206.005368	7.7940	0.10	9.9
cis	5	6.01	−1206.005260	9.1481	0.17	8.8
cis	6	7.33	−1206.005164	7.8725	0.23	7.9
cis	7	10.25	−1206.005007	9.5126	0.33	6.7
cis	8	6.31	−1206.004893	9.5442	0.40	6.0
cis	9	4.92	−1206.004828	9.2757	0.44	5.6
cis	10	4.47	−1206.004557	9.1537	0.61	4.2
cis	11	10.61	−1206.004494	10.6269	0.65	3.9
cis	12	7.30	−1206.004486	9.2391	0.66	3.9
cis	13	7.03	−1206.004452	8.8587	0.68	3.7
cis	14	10.51	−1206.004004	10.3931	0.96	2.3
cis	15	11.17	−1206.003799	10.3667	1.09	1.9
cis	16	11.10	−1206.003798	10.5079	1.09	1.9

Table 10. Dipole moments, free enthalpies, lowest frequencies and Boltzmann statistics-derived populations at 310.15 K of a diverse set of conformations of the Azo-tail at the B3LYP/6-31G* level. Boltzmann-weighted average dipole moment for the *cis* isomer: 7.93 D. Same quantity for the *trans* isomer: 5.31 D.

Isomer	Conformation	Dipole moment (D)	Free enthalpy (Ha)	Lowest frequency (cm^{-1})	Relative free enthalpy (kcal/mol)	Population (%)
trans	1	4.70	−937.745404	8.4615	0.00	71.1
trans	2	4.89	−937.744552	13.8562	0.53	28.8
cis	1	6.09	−937.722962	10.2104	0.00	63.0
cis	2	8.02	−937.722458	11.0964	0.32	36.9

Table 11. Dipole moments, free enthalpies, lowest frequencies and Boltzmann statistics-derived populations at 310.15 K of a diverse set of conformations of Azo-reference at the B3LYP/6-31G* level. Boltzmann-weighted average dipole moment for the *cis* isomer: 6.80 D. Same quantity for the *trans* isomer: 4.75 D. The conformations associated with a relative free enthalpy 4 kcal/mol higher than the global minimum were omitted for clarity.

12.3 - Total electron densities

Two geometrically optimised models (one for the Gd-DOTA complex head and one for the azo-containing tail) at the same level of theory were employed to estimate total electron density for each part. A density value of $6.593 \cdot 10^{-4}$ a.u. (found to correctly reproduce the molecular volume of the water molecule at the same level of theory) was used for the definition of the electron isodensity surface used as an envelope for the molecular volume. The resulting values of 0.552 and 0.373 e/$Å^3$ (respectively for the head and tail parts) were used for fitting the SAXS-derived core-shell model (cf. Supplementary Section 13).

12.4 - Molecular models of GdAzo derivatives

Diastereoisomer 1

Electronic energy (B3LYP): −3416.41636101 Ha.
Lowest frequency: 8.539 cm^{-1}.
Free enthalpy: −3415.651178 Ha.
Atomic coordinates:

```
  1  C     6.895158   2.819710   2.013122
  2  C     7.963844   1.740552   2.201120
  3  C     9.041824  -0.224874   1.240811
  4  C     8.057107  -1.344397   1.587407
  5  C     5.920256  -2.398498   1.079099
  6  C     4.910237  -1.507213   1.808354
  7  C     3.766829   0.648968   1.834063
```

8	C	4.830213	1.586395	2.415315
9	C	4.866116	3.406570	0.778574
10	C	3.959871	2.989804	-0.399494
11	C	9.259991	1.948875	0.134460
12	C	8.530948	2.960413	-0.776507
13	C	7.708609	-2.402229	-0.593041
14	C	8.440037	-1.541006	-1.645520
15	C	3.291980	-0.913205	-0.005434
16	C	3.964327	-1.626521	-1.214738
17	H	6.698518	3.280171	2.994092
18	N	5.638804	2.310794	1.406767
19	N	8.383989	1.073447	0.946079
20	N	7.077385	-1.654260	0.518430
21	N	4.318984	-0.431091	0.975297
22	O	4.335807	1.963927	-1.083536
23	O	2.971110	3.693650	-0.640059
24	O	7.359785	2.623893	-1.196881
25	O	9.137016	3.994139	-1.084575
26	O	8.007441	-0.338655	-1.814193
27	O	9.350923	-2.075426	-2.290302
28	O	4.982397	-1.017658	-1.716674
29	O	3.491256	-2.687814	-1.645740
30	H	7.282665	3.610679	1.369028
31	H	7.597075	0.974299	2.887512
32	H	8.833395	2.204869	2.693659
33	H	9.750607	-0.123899	2.077722
34	H	9.629297	-0.510767	0.367064
35	H	8.640951	-2.244877	1.837834
36	H	7.501217	-1.079513	2.489873
37	H	6.260015	-3.171091	1.786737
38	H	5.425141	-2.924136	0.261766
39	H	4.129550	-2.155018	2.231509
40	H	5.396411	-1.031431	2.663077
41	H	3.200613	0.232749	2.680839
42	H	3.055845	1.223002	1.236970
43	H	4.319636	2.304651	3.077120
44	H	5.511774	1.017018	3.051079
45	H	5.579885	4.119986	0.355880
46	H	4.260796	3.945152	1.521063
47	H	9.834053	1.311315	-0.544467
48	H	9.977979	2.491238	0.765337
49	H	8.401385	-3.168153	-0.216849
50	H	6.915158	-2.916966	-1.143163
51	H	2.885053	0.008197	-0.437700
52	C	2.132164	-1.720085	0.607333
53	C	0.881024	-1.688166	-0.281110
54	C	-0.322839	-2.344689	0.385250
55	O	-0.569126	-2.218546	1.587489
56	N	-1.120900	-3.069754	-0.448886

57	C	-2.363612	-3.683067	-0.013772
58	C	-3.547718	-2.728403	-0.176579
59	O	-4.711136	-3.435658	0.261104
60	C	-5.921098	-2.818943	0.201621
61	C	-7.021125	-3.582971	0.626777
62	C	-8.295455	-3.036201	0.598473
63	C	-8.506562	-1.720372	0.148412
64	C	-7.400403	-0.962569	-0.273100
65	C	-6.118711	-1.500675	-0.248863
66	N	-9.844792	-1.271524	0.160745
67	N	-10.019789	-0.084941	-0.244815
68	C	-11.356143	0.366363	-0.236763
69	C	-11.560469	1.686055	-0.679595
70	C	-12.832708	2.235802	-0.714963
71	C	-13.940449	1.472222	-0.305731
72	C	-13.748176	0.149921	0.138663
73	C	-12.467994	-0.391195	0.170696
74	O	-15.144869	2.092581	-0.374894
75	C	-16.326452	1.379077	0.022543
76	C	-17.514054	2.313794	-0.157505
77	C	-18.841162	1.651198	0.235978
78	C	-20.042374	2.585883	0.058281
79	H	1.862838	-1.310147	1.586498
80	H	2.433754	-2.761629	0.759284
81	H	1.100069	-2.154023	-1.245240
82	H	0.600368	-0.643789	-0.482161
83	H	-0.887828	-3.107285	-1.431678
84	H	-2.258451	-3.960089	1.037530
85	H	-2.537451	-4.591785	-0.597192
86	H	-3.658981	-2.426765	-1.225772
87	H	-3.393853	-1.830117	0.433609
88	H	-6.850755	-4.597910	0.971675
89	H	-9.154223	-3.616305	0.923111
90	H	-7.559632	0.052393	-0.619832
91	H	-5.281741	-0.895977	-0.578103
92	H	-10.697457	2.265790	-0.993584
93	H	-12.997683	3.253274	-1.055152
94	H	-14.589106	-0.454706	0.457395
95	H	-12.314163	-1.408929	0.512010
96	H	-16.437697	0.479297	-0.597072
97	H	-16.230361	1.063982	1.070085
98	H	-17.351286	3.214405	0.448929
99	H	-17.554051	2.637676	-1.205766
100	H	-18.989715	0.745274	-0.367748
101	H	-18.787225	1.319114	1.281946
102	H	-20.975990	2.089374	0.345697
103	H	-19.937483	3.486130	0.676028
104	H	-20.141535	2.908024	-0.985489
105	Gd	6.234747	0.642893	-0.620563

Diastereoisomer 2

Electronic energy (B3LYP): −3416.39284977 Ha.
Lowest frequency: 6.0096 cm^{-1}.
Free enthalpy: −3415.628616 Ha.
Atomic coordinates:

1	C	-6.900799	0.672745	3.149511
2	C	-7.491666	1.804390	2.305586
3	C	-8.043079	2.574661	0.058518
4	C	-6.698735	3.104875	-0.445876
5	C	-4.481763	2.539854	-1.282764
6	C	-3.701200	2.218141	-0.005303
7	C	-3.339072	0.611443	1.794593
8	C	-4.509464	0.907856	2.737284
9	C	-5.475795	-1.314874	3.081345
10	C	-4.740530	-2.292232	2.139092
11	C	-9.178790	0.616025	0.989405
12	C	-9.023555	-0.892064	1.283730
13	C	-6.465685	1.939441	-2.586146
14	C	-7.634332	0.935863	-2.695736
15	C	-2.757278	-0.043969	-0.504176
16	C	-3.397001	-0.315065	-1.898307
17	H	-6.669686	1.076184	4.147876
18	N	-5.699278	0.046193	2.541799
19	N	-7.918552	1.392270	0.947696
20	N	-5.906917	2.125969	-1.227773
21	N	-3.669281	0.779169	0.355736
22	O	-4.893044	-2.100192	0.873481
23	O	-4.104656	-3.219177	2.655923
24	O	-7.919108	-1.444394	0.913878
25	O	-9.983096	-1.477068	1.801363
26	O	-7.662806	-0.019924	-1.831439
27	O	-8.429108	1.082996	-3.632833
28	O	-4.633305	-0.675114	-1.876299
29	O	-2.709273	-0.208765	-2.923596
30	H	-7.650374	-0.106916	3.293605
31	H	-6.758840	2.607861	2.203164
32	H	-8.343773	2.232198	2.858261
33	H	-8.562769	3.398803	0.572178
34	H	-8.667724	2.292618	-0.790364
35	H	-6.894508	4.004147	-1.052329
36	H	-6.091731	3.433767	0.400889
37	H	-4.396462	3.622584	-1.466003
38	H	-4.015133	2.044363	-2.135045
39	H	-2.684645	2.620871	-0.117119
40	H	-4.143643	2.758177	0.834747
41	H	-2.506663	1.267708	2.090531
42	H	-2.995134	-0.413748	1.943761
43	H	-4.140975	0.813791	3.771602

44	H	-4.820673	1.947752	2.617522
45	H	-6.455211	-1.768598	3.260084
46	H	-4.946106	-1.280487	4.043569
47	H	-9.638195	0.670181	-0.002100
48	H	-9.890079	1.047447	1.707504
49	H	-6.777995	2.897585	-3.024830
50	H	-5.673958	1.529467	-3.220518
51	H	-2.751449	-1.027694	-0.020817
52	C	-1.307457	0.467424	-0.596954
53	C	-0.329444	-0.656782	-0.966945
54	C	1.112180	-0.166175	-1.041785
55	O	1.579842	0.653302	-0.246952
56	N	1.852188	-0.706003	-2.050864
57	C	3.255254	-0.394619	-2.258758
58	C	4.172024	-1.345646	-1.487197
59	O	5.511708	-0.992498	-1.839754
60	C	6.543381	-1.707638	-1.315604
61	C	7.824426	-1.364932	-1.786311
62	C	8.949863	-2.015276	-1.305240
63	C	8.819861	-3.044355	-0.351283
64	C	7.536956	-3.417567	0.069093
65	C	6.402601	-2.741779	-0.379874
66	N	9.892919	-3.884925	0.080688
67	N	11.055725	-3.485777	0.342984
68	C	11.432354	-2.113152	0.478504
69	C	10.693634	-1.161619	1.208977
70	C	11.209696	0.107198	1.421292
71	C	12.463242	0.470111	0.892608
72	C	13.212876	-0.478608	0.181574
73	C	12.705196	-1.766305	0.009570
74	O	12.860877	1.745763	1.138422
75	C	14.125868	2.190100	0.626224
76	C	14.298157	3.651171	1.016277
77	C	15.625793	4.234759	0.514294
78	C	15.806664	5.706820	0.899471
79	H	-0.992271	0.893904	0.361204
80	H	-1.236779	1.261269	-1.347398
81	H	-0.634041	-1.112254	-1.912754
82	H	-0.365418	-1.443363	-0.199367
83	H	1.421544	-1.393041	-2.653986
84	H	3.432061	0.632138	-1.929928
85	H	3.478152	-0.459708	-3.327632
86	H	3.969970	-2.388278	-1.764105
87	H	4.014006	-1.231519	-0.407795
88	H	7.911017	-0.583669	-2.535124
89	H	9.927717	-1.743680	-1.686612
90	H	7.435289	-4.243128	0.767449
91	H	5.426367	-3.038146	-0.014904
92	H	9.727560	-1.424296	1.626069

93	H	10.656277	0.843319	1.996303
94	H	14.189404	-0.234569	-0.219002
95	H	13.293886	-2.516035	-0.511142
96	H	14.930757	1.573507	1.048714
97	H	14.142274	2.071676	-0.465637
98	H	13.457950	4.227939	0.607882
99	H	14.241754	3.735572	2.109467
100	H	16.459841	3.645082	0.919128
101	H	15.678677	4.133347	-0.578497
102	H	16.760919	6.098277	0.529389
103	H	15.004814	6.326510	0.479828
104	H	15.791402	5.835330	1.988653
105	Gd	-6.111337	-0.326301	-0.088717

Diastereoisomer 3

Electronic energy (B3LYP): −3416.41345475 Ha.
Lowest frequency: 5.4582 cm^{-1}.
Free enthalpy: −3415.649787 Ha.
Atomic coordinates:

1	C	-8.484870	2.198891	-1.152162
2	C	-8.467466	1.131278	-2.244210
3	C	-7.615689	-1.080760	-2.821834
4	C	-6.155556	-0.821431	-3.195928
5	C	-3.905919	-0.389050	-2.330657
6	C	-3.859342	1.125943	-2.088496
7	C	-4.775961	2.949968	-0.755688
8	C	-6.215860	3.091686	-1.244591
9	C	-7.438675	2.951604	0.886856
10	C	-8.092319	1.975444	1.891509
11	C	-9.241735	-0.855413	-1.040205
12	C	-8.812748	-2.021405	-0.123946
13	C	-5.019009	-2.460976	-1.773026
14	C	-4.455917	-2.732470	-0.360965
15	C	-3.138327	1.564065	0.301754
16	C	-3.772547	1.593741	1.721440
17	H	-8.781026	3.160527	-1.600083
18	N	-7.194792	2.337156	-0.434471
19	N	-8.108803	-0.207717	-1.730736
20	N	-5.220799	-1.025216	-2.066574
21	N	-4.239810	1.557594	-0.721309
22	O	-7.930126	0.716946	1.655449
23	O	-8.693252	2.459248	2.857232
24	O	-7.596601	-1.995398	0.306484
25	O	-9.661313	-2.874651	0.159628
26	O	-4.631220	-1.803842	0.518734
27	O	-3.908235	-3.822246	-0.161402

28	O	-4.910289	1.003647	1.862649
29	O	-3.143622	2.155493	2.628283
30	H	-9.248627	1.952192	-0.411401
31	H	-7.746819	1.401818	-3.019948
32	H	-9.454223	1.113245	-2.733571
33	H	-8.236651	-0.963133	-3.723585
34	H	-7.735607	-2.117176	-2.498636
35	H	-5.889291	-1.472637	-4.043680
36	H	-6.039171	0.206821	-3.548574
37	H	-3.598843	-0.573873	-3.372533
38	H	-3.175196	-0.888808	-1.698007
39	H	-2.854936	1.486632	-2.357048
40	H	-4.544801	1.615881	-2.784942
41	H	-4.134741	3.584967	-1.386559
42	H	-4.701434	3.358063	0.254265
43	H	-6.464182	4.165503	-1.259152
44	H	-6.295309	2.745365	-2.277863
45	H	-8.060559	3.854669	0.802262
46	H	-6.484135	3.255319	1.324831
47	H	-9.996268	-1.216098	-1.754421
48	H	-9.730262	-0.118428	-0.395866
49	H	-5.983117	-2.973994	-1.822241
50	H	-4.362514	-2.931713	-2.518924
51	H	-2.569040	2.499231	0.187262
52	Gd	-6.218018	-0.083544	0.240712
53	C	-2.117440	0.413248	0.209070
54	C	-0.924073	0.603975	1.157737
55	C	0.176149	-0.413278	0.869693
56	O	0.463437	-0.776539	-0.274191
57	N	0.836706	-0.882280	1.964201
58	C	1.954885	-1.804959	1.863597
59	C	3.273743	-1.071826	1.612478
60	O	4.287825	-2.076216	1.518051
61	C	5.570412	-1.701535	1.269740
62	C	6.508275	-2.744408	1.182818
63	C	7.841288	-2.456701	0.930420
64	C	8.273449	-1.129057	0.759483
65	C	7.328857	-0.092047	0.848849
66	C	5.989981	-0.369135	1.101021
67	N	9.651314	-0.957319	0.506226
68	N	10.028146	0.242353	0.358065
69	C	11.404378	0.416472	0.102006
70	C	11.835758	1.746267	-0.055892
71	C	13.167021	2.036880	-0.311709
72	C	14.106514	0.995818	-0.417346
73	C	13.686234	-0.339077	-0.261514
74	C	12.348951	-0.619131	-0.004868
75	O	15.384376	1.374997	-0.669643
76	C	16.403518	0.370399	-0.793005

77	C	17.721303	1.079131	-1.071603
78	C	18.889914	0.095956	-1.222947
79	C	20.221278	0.800963	-1.503205
80	H	-1.704118	0.363524	-0.801428
81	H	-2.607253	-0.542047	0.420553
82	H	-1.236301	0.562242	2.204076
83	H	-0.487110	1.601114	1.005456
84	H	0.583220	-0.530012	2.876783
85	H	1.762778	-2.494117	1.037504
86	H	2.020002	-2.382887	2.789300
87	H	3.497424	-0.381074	2.435128
88	H	3.212571	-0.501740	0.678025
89	H	6.167419	-3.766333	1.315339
90	H	8.575926	-3.253386	0.859864
91	H	7.658658	0.932496	0.717607
92	H	5.280516	0.447706	1.164588
93	H	11.101203	2.541845	0.027480
94	H	13.506842	3.060439	-0.434379
95	H	14.394687	-1.155347	-0.339690
96	H	12.019832	-1.645320	0.115451
97	H	16.459875	-0.211506	0.136556
98	H	16.146044	-0.315124	-1.611369
99	H	17.615225	1.677894	-1.985699
100	H	17.926341	1.780678	-0.252379
101	H	18.980201	-0.506912	-0.308807
102	H	18.670617	-0.609025	-2.036670
103	H	21.037860	0.077626	-1.606870
104	H	20.171585	1.385539	-2.429954
105	H	20.483541	1.488286	-0.689652

Diastereoisomer 4

Electronic energy (B3LYP): −3416.39012922 Ha.
Lowest frequency: 4.4524 cm^{-1}.
Free enthalpy: −3415.62608 Ha.
Atomic coordinates:

1	C	-8.126783	0.362836	1.772640
2	C	-7.665657	1.816767	1.850241
3	C	-6.110783	3.443688	0.909684
4	C	-4.775966	3.088066	1.566039
5	C	-2.875047	1.544750	1.548727
6	C	-3.326649	0.349249	2.398236
7	C	-4.899636	-1.507520	2.527168
8	C	-6.252758	-0.841967	2.769966
9	C	-7.582134	-1.840152	0.955296
10	C	-7.997704	-1.676995	-0.524224
11	C	-7.900258	2.566775	-0.469219

12	C	-7.223535	2.600411	-1.856094
13	C	-3.338001	2.888688	-0.405453
14	C	-2.860174	1.947753	-1.532397
15	C	-3.029810	-1.783184	1.060712
16	C	-3.785332	-2.579816	-0.040787
17	H	-8.662927	0.110839	2.701270
18	N	-7.024948	-0.600198	1.533311
19	N	-6.961418	2.263436	0.628989
20	N	-3.947055	2.180064	0.742059
21	N	-3.961791	-0.757180	1.641834
22	O	-7.430531	-0.717363	-1.174605
23	O	-8.812045	-2.483416	-0.987165
24	O	-6.128486	1.928144	-1.974588
25	O	-7.783892	3.233008	-2.758396
26	O	-3.399068	0.775386	-1.570439
27	O	-2.025427	2.386390	-2.331410
28	O	-4.679450	-1.938423	-0.712687
29	O	-3.469620	-3.763463	-0.223117
30	H	-8.845243	0.250095	0.957797
31	H	-6.990546	1.948490	2.699379
32	H	-8.542155	2.452599	2.052518
33	H	-6.641418	4.159873	1.556308
34	H	-5.931613	3.958088	-0.037065
35	H	-4.232007	4.021065	1.783497
36	H	-4.952524	2.602715	2.529665
37	H	-2.420666	2.286727	2.224775
38	H	-2.094316	1.235235	0.857011
39	H	-2.461642	-0.007053	2.977834
40	H	-4.054179	0.696063	3.136738
41	H	-4.427827	-1.693390	3.504527
42	H	-5.061115	-2.490768	2.080557
43	H	-6.821551	-1.476984	3.468769
44	H	-6.112815	0.117439	3.273864
45	H	-8.442508	-2.204007	1.535654
46	H	-6.820043	-2.623642	0.979801
47	H	-8.426154	3.517673	-0.299663
48	H	-8.658745	1.779303	-0.513045
49	H	-4.084629	3.549290	-0.853966
50	H	-2.497922	3.520321	-0.082628
51	H	-2.771245	-2.501489	1.853754
52	Gd	-5.417771	0.280472	-0.448836
53	C	-1.699636	-1.247910	0.495919
54	C	-0.729783	-2.370802	0.095906
55	C	0.634646	-1.801595	-0.280945
56	O	1.152520	-0.860325	0.326559
57	N	1.251974	-2.410992	-1.330291
58	C	2.567131	-2.009757	-1.800104
59	C	3.688910	-2.687415	-1.011347
60	O	4.917343	-2.209607	-1.564902

61	C	6.093125	-2.621109	-1.017799
62	C	7.257927	-2.111404	-1.620419
63	C	8.510083	-2.445724	-1.128587
64	C	8.629086	-3.320719	-0.030951
65	C	7.467802	-3.871271	0.524722
66	C	6.202909	-3.506987	0.062947
67	N	9.872884	-3.849029	0.437803
68	N	10.921249	-3.173145	0.592867
69	C	10.979688	-1.745017	0.554935
70	C	10.052522	-0.898723	1.194943
71	C	10.275561	0.468292	1.243921
72	C	11.414085	1.032439	0.637182
73	C	12.350252	0.194316	0.013498
74	C	12.139891	-1.184426	0.006657
75	O	11.521112	2.383639	0.725698
76	C	12.656310	3.032248	0.133237
77	C	12.509387	4.529212	0.366309
78	C	13.674403	5.329300	-0.231761
79	C	13.533571	6.837742	-0.001657
80	H	-1.186151	-0.650595	1.253635
81	H	-1.892496	-0.603930	-0.367251
82	H	-1.140012	-2.981012	-0.712290
83	H	-0.575449	-3.047264	0.948427
84	H	0.796455	-3.192659	-1.780459
85	H	2.657554	-0.925831	-1.693200
86	H	2.654414	-2.261584	-2.860357
87	H	3.627526	-3.779183	-1.104762
88	H	3.616370	-2.418755	0.049278
89	H	7.154376	-1.451133	-2.475943
90	H	9.397641	-2.047276	-1.607610
91	H	7.563143	-4.583252	1.339365
92	H	5.322561	-3.929696	0.532324
93	H	9.172253	-1.314933	1.672283
94	H	9.576697	1.127650	1.749479
95	H	13.245160	0.597917	-0.444671
96	H	12.876962	-1.842990	-0.443671
97	H	13.578518	2.652198	0.593209
98	H	12.690876	2.803919	-0.940625
99	H	11.560862	4.864466	-0.073336
100	H	12.443696	4.714860	1.446418
101	H	14.619860	4.978999	0.204864
102	H	13.739690	5.128166	-1.309979
103	H	14.376888	7.384991	-0.437691
104	H	12.612863	7.222699	-0.456832
105	H	13.499093	7.072830	1.069148

Diastereoisomer 5

Electronic energy (B3LYP): −3416.40734527 Ha.
Lowest frequency: 5.0274 cm^{-1}.
Free enthalpy: −3415.643274 Ha.
Atomic coordinates:

1	C	-1.967072	-0.725721	0.950155
2	C	-1.375958	-1.972138	1.628505
3	C	-0.192588	-2.533247	0.843295
4	O	-0.139538	-2.499526	-0.387993
5	N	0.784285	-3.105754	1.601796
6	C	1.970008	-3.719430	1.029846
7	C	3.142753	-2.738143	0.989887
8	O	4.255800	-3.449289	0.441950
9	C	5.459143	-2.823807	0.348328
10	C	6.515895	-3.599195	-0.158096
11	C	7.781860	-3.046433	-0.282079
12	C	8.027311	-1.713147	0.092940
13	C	6.963231	-0.943029	0.593192
14	C	5.690101	-1.486871	0.721178
15	N	9.355024	-1.261721	-0.068321
16	N	9.563002	-0.061790	0.278042
17	C	10.890314	0.390679	0.125676
18	C	11.131671	1.722962	0.508076
19	C	12.400104	2.273416	0.407749
20	C	13.466652	1.498060	-0.080972
21	C	13.236637	0.164041	-0.468796
22	C	11.960601	-0.377813	-0.364323
23	O	14.671090	2.119358	-0.140435
24	C	15.814286	1.391737	-0.616628
25	C	17.014131	2.326457	-0.559205
26	C	18.303235	1.649987	-1.044655
27	C	19.516392	2.584500	-0.989129
28	H	-1.174686	0.030528	0.924896
29	H	-2.223402	-0.928026	-0.091275
30	H	-2.114274	-2.778441	1.710169
31	H	-1.071690	-1.731015	2.654141
32	H	0.701084	-3.074031	2.608663
33	H	1.728193	-4.045777	0.016215
34	H	2.244383	-4.597254	1.622589
35	H	3.380815	-2.381121	1.999812
36	H	2.892681	-1.874296	0.361795
37	H	6.319764	-4.628142	-0.442461
38	H	8.607809	-3.635903	-0.668924
39	H	7.148281	0.085917	0.880878
40	H	4.885728	-0.872139	1.108090
41	H	10.300679	2.311492	0.885554
42	H	12.593736	3.300143	0.701899
43	H	14.045203	-0.449148	-0.849011

44	H	11.778172	-1.404938	-0.660466
45	H	15.975289	0.507431	0.014257
46	H	15.630821	1.050792	-1.644263
47	H	16.802856	3.212446	-1.172157
48	H	17.141325	2.675856	0.473796
49	H	18.499744	0.758305	-0.433494
50	H	18.162763	1.293145	-2.074283
51	H	20.422399	2.078016	-1.340401
52	H	19.362288	3.470076	-1.617675
53	H	19.701738	2.931170	0.034855
54	C	-7.756186	-1.894767	-1.067391
55	C	-8.619561	-0.973574	-0.203390
56	C	-8.865453	1.250270	0.766567
57	C	-8.012458	1.140264	2.031670
58	C	-5.761044	1.032383	2.945322
59	C	-5.352262	-0.419004	2.682529
60	C	-4.581135	-2.045138	1.047811
61	C	-5.917677	-2.476534	0.427505
62	C	-5.510704	-2.228439	-1.973734
63	C	-4.120557	-1.580128	-2.135217
64	C	-8.868831	0.975863	-1.662075
65	C	-7.962118	0.761367	-2.892684
66	C	-6.530121	3.078862	1.835785
67	C	-6.803561	3.728677	0.461597
68	C	-3.150275	-0.063762	1.699915
69	C	-3.079890	1.458202	1.377580
70	H	-8.086910	-2.931416	-0.898311
71	N	-6.299154	-1.757631	-0.811604
72	N	-8.332970	0.469644	-0.378240
73	N	-6.613070	1.601339	1.871427
74	N	-4.528520	-0.621534	1.463158
75	O	-3.993592	-0.373937	-1.698771
76	O	-3.241529	-2.234708	-2.709343
77	O	-6.692511	0.722969	-2.676217
78	O	-8.506631	0.695437	-4.001907
79	O	-6.519476	3.025951	-0.581084
80	O	-7.223299	4.892558	0.452228
81	O	-3.823343	1.902973	0.426691
82	O	-2.277262	2.137076	2.033650
83	H	-7.933959	-1.678886	-2.122081
84	H	-8.484065	-1.223608	0.851126
85	H	-9.676591	-1.182010	-0.435717
86	H	-9.889318	0.930475	1.017329
87	H	-8.930499	2.294934	0.458469
88	H	-8.511156	1.714530	2.829181
89	H	-7.990528	0.102606	2.372020
90	H	-6.279351	1.068071	3.916295
91	H	-4.872885	1.657831	3.047594
92	H	-4.811721	-0.786444	3.569590

93	H	-6.249061	-1.035047	2.598574
94	H	-4.396552	-2.714078	1.903506
95	H	-3.788473	-2.223519	0.324923
96	H	-5.851163	-3.558321	0.228516
97	H	-6.724434	-2.351544	1.152343
98	H	-6.061062	-1.963980	-2.881425
99	H	-5.396072	-3.321215	-1.960886
100	H	-8.982734	2.060506	-1.572987
101	H	-9.862289	0.556326	-1.874013
102	H	-7.195572	3.535441	2.581937
103	H	-5.504908	3.363607	2.091194
104	Gd	-5.657036	0.840201	-0.563105
105	H	-2.931209	-0.145388	2.773965

Diastereoisomer 6

Electronic energy (B3LYP): −3416.38428607 Ha.
Lowest frequency: 4.7356 cm^{-1}.
Free enthalpy: −3415.618862 Ha.
Atomic coordinates:

1	C	5.765939	2.713415	1.954372
2	C	7.099756	2.092812	1.533737
3	C	8.282259	0.770408	-0.139964
4	C	7.956557	-0.658223	0.297783
5	C	6.255482	-2.389838	0.424772
6	C	5.399217	-1.946219	1.614131
7	C	3.664700	-0.441224	2.413785
8	C	4.509027	0.789451	2.772896
9	C	3.344368	2.616309	1.628741
10	C	2.231881	2.009679	0.749124
11	C	7.317549	2.929821	-0.756126
12	C	6.000978	3.647885	-1.122577
13	C	7.088960	-1.656488	-1.762810
14	C	7.008545	-0.532467	-2.819642
15	C	3.182531	-2.163819	0.623579
16	C	3.422232	-2.326527	-0.906286
17	H	5.838189	2.991336	3.017429
18	N	4.595393	1.828198	1.718712
19	N	7.174750	1.729193	0.099273
20	N	6.776304	-1.251682	-0.373802
21	N	4.164998	-1.206805	1.245067
22	O	2.604953	1.211267	-0.191261
23	O	1.069002	2.367422	0.973380
24	O	4.943334	2.915238	-1.195346
25	O	6.057567	4.858230	-1.373908
26	O	6.191749	0.435511	-2.580542
27	O	7.689454	-0.662994	-3.844633

28	O	3.856818	-1.304063	-1.554469
29	O	3.136489	-3.422958	-1.407979
30	H	5.603914	3.639484	1.400503
31	H	7.290444	1.193424	2.123534
32	H	7.901617	2.804586	1.788556
33	H	9.196916	1.088449	0.385016
34	H	8.512473	0.783692	-1.206358
35	H	8.847622	-1.281331	0.118486
36	H	7.780115	-0.681465	1.375504
37	H	7.081778	-3.006102	0.812120
38	H	5.673317	-3.035542	-0.234460
39	H	5.143268	-2.840998	2.203892
40	H	5.997421	-1.311223	2.270161
41	H	3.627743	-1.074519	3.314453
42	H	2.646992	-0.118281	2.206648
43	H	4.080593	1.221868	3.691378
44	H	5.525192	0.483333	3.028782
45	H	3.584474	3.577448	1.165067
46	H	2.933668	2.826605	2.626087
47	H	7.746692	2.610633	-1.710672
48	H	8.009025	3.658158	-0.310036
49	H	8.077048	-2.133446	-1.827562
50	H	6.345248	-2.397096	-2.072099
51	Gd	4.832733	0.627079	-0.667549
52	H	3.357885	-3.159149	1.055613
53	C	1.674811	-1.856838	0.799480
54	C	1.049986	-2.369554	2.109537
55	C	-0.415627	-1.960621	2.218472
56	O	-0.794273	-0.813114	1.970173
57	N	-1.270707	-2.935472	2.633616
58	C	-2.694138	-2.703662	2.811263
59	C	-3.473131	-2.964804	1.520990
60	O	-4.849140	-2.716447	1.816642
61	C	-5.772792	-2.868933	0.828631
62	C	-7.108549	-2.627806	1.198758
63	C	-8.128704	-2.734417	0.265994
64	C	-7.838767	-3.109013	-1.060629
65	C	-6.514505	-3.403396	-1.406773
66	C	-5.477331	-3.257176	-0.485489
67	N	-8.836717	-3.407388	-2.041157
68	N	-9.867308	-2.716775	-2.242858
69	C	-10.090756	-1.412558	-1.701834
70	C	-9.118509	-0.393204	-1.663830
71	C	-9.469272	0.889654	-1.274020
72	C	-10.790609	1.187424	-0.889042
73	C	-11.767459	0.181887	-0.940275
74	C	-11.415130	-1.095484	-1.375095
75	O	-11.017978	2.470157	-0.504968
76	C	-12.343978	2.854642	-0.112283

77	C	-12.305740	4.326898	0.271961
78	C	-13.680467	4.850803	0.708667
79	C	-13.650579	6.332536	1.098156
80	H	1.167964	-2.376426	-0.021496
81	H	1.471761	-0.793701	0.657399
82	H	1.557962	-1.953058	2.986593
83	H	1.160143	-3.458293	2.173785
84	H	-0.914006	-3.868782	2.784055
85	H	-2.837933	-1.665218	3.118671
86	H	-3.064534	-3.356421	3.606654
87	H	-3.336016	-4.001869	1.188945
88	H	-3.123567	-2.292169	0.728344
89	H	-7.322145	-2.357057	2.228182
90	H	-9.153477	-2.550120	0.569305
91	H	-6.298379	-3.737347	-2.417428
92	H	-4.459559	-3.465564	-0.793191
93	H	-8.097098	-0.601257	-1.962556
94	H	-8.732366	1.686825	-1.258617
95	H	-12.796139	0.385917	-0.668555
96	H	-12.172607	-1.870202	-1.451392
97	H	-13.037977	2.684881	-0.946529
98	H	-12.670332	2.236451	0.734937
99	H	-11.578605	4.462427	1.083310
100	H	-11.939402	4.907148	-0.585055
101	H	-14.403356	4.700746	-0.105085
102	H	-14.044660	4.256340	1.557924
103	H	-14.643119	6.681146	1.404966
104	H	-12.960845	6.508061	1.932734
105	H	-13.321463	6.955603	0.257596

Diastereoisomer 7

Electronic energy (B3LYP): −3416.41550409 Ha.
Lowest frequency: 6.3149 cm^{-1}.
Free enthalpy: −3415.651824 Ha.
Atomic coordinates:

1	C	-8.436329	-1.423633	1.158363
2	C	-8.291721	-0.276405	2.159309
3	C	-7.408190	1.982615	2.485410
4	C	-5.924838	1.749072	2.775314
5	C	-3.778879	1.126322	1.781403
6	C	-3.855414	-0.398447	1.692969
7	C	-4.795910	-2.288956	0.455515
8	C	-6.198269	-2.400130	1.060486
9	C	-7.620876	-2.320098	-0.934220
10	C	-8.357786	-1.386753	-1.918910
11	C	-9.172608	1.619522	0.871273

12	C	-8.813166	2.718104	-0.153632
13	C	-4.841735	3.196305	1.115931
14	C	-4.337293	3.284850	-0.341312
15	C	-3.227548	-0.787949	-0.696390
16	C	-3.892489	-0.782016	-2.117182
17	H	-8.695852	-2.340925	1.709591
18	N	-7.231509	-1.644278	0.320724
19	N	-7.986179	1.020026	1.516013
20	N	-5.078136	1.807770	1.562778
21	N	-4.273126	-0.905075	0.365127
22	O	-8.149603	-0.121948	-1.770472
23	O	-9.063454	-1.907482	-2.790288
24	O	-7.626454	2.664137	-0.659254
25	O	-9.684042	3.546663	-0.440979
26	O	-4.610575	2.278673	-1.102219
27	O	-3.740750	4.311417	-0.684483
28	O	-5.155434	-0.527539	-2.166958
29	O	-3.174115	-0.981851	-3.103602
30	H	-9.272763	-1.219228	0.486519
31	H	-7.489785	-0.499790	2.867603
32	H	-9.218320	-0.211120	2.751515
33	H	-7.961435	1.952318	3.437062
34	H	-7.547957	2.986087	2.077346
35	H	-5.592915	2.490010	3.519667
36	H	-5.787760	0.768001	3.236905
37	H	-3.366335	1.397430	2.765837
38	H	-3.075988	1.505474	1.036443
39	H	-2.884228	-0.804671	2.000269
40	H	-4.575682	-0.767043	2.427273
41	H	-4.128623	-2.928234	1.053314
42	H	-4.803370	-2.705195	-0.554021
43	H	-6.461813	-3.468813	1.112266
44	H	-6.189434	-2.039233	2.091853
45	H	-8.243729	-3.204898	-0.737251
46	H	-6.718179	-2.662845	-1.447355
47	H	-9.869664	2.032571	1.614837
48	H	-9.707634	0.842244	0.318334
49	H	-5.785957	3.747184	1.153700
50	H	-4.132383	3.715028	1.777044
51	Gd	-6.298476	0.719666	-0.563570
52	H	-2.804100	0.218861	-0.600749
53	C	-2.057906	-1.800122	-0.676658
54	C	-0.969454	-1.609243	0.389981
55	C	0.318914	-2.341305	0.006176
56	O	0.710413	-2.433121	-1.159403
57	N	1.015665	-2.875186	1.047297
58	C	2.283234	-3.562575	0.867118
59	C	3.462235	-2.588622	0.830385
60	O	4.637912	-3.384557	0.656856

61	C	5.847710	-2.772573	0.563494
62	C	6.953636	-3.624265	0.401805
63	C	8.228734	-3.089566	0.295920
64	C	8.435004	-1.699087	0.347346
65	C	7.323299	-0.854470	0.510242
66	C	6.040477	-1.379927	0.618278
67	N	9.774537	-1.271309	0.227025
68	N	9.948298	-0.017793	0.266753
69	C	11.287217	0.410057	0.146256
70	C	11.492856	1.801424	0.179844
71	C	12.767574	2.335184	0.068237
72	C	13.876484	1.483209	-0.080300
73	C	13.682917	0.088834	-0.113837
74	C	12.400385	-0.435612	-0.001533
75	O	15.083234	2.094486	-0.182322
76	C	16.264936	1.293438	-0.340492
77	C	17.455777	2.237064	-0.429695
78	C	18.782341	1.484780	-0.601426
79	C	19.987345	2.427021	-0.692201
80	H	-1.579573	-1.702358	-1.652419
81	H	-2.441576	-2.826261	-0.630059
82	H	-1.292586	-1.938701	1.382343
83	H	-0.713168	-0.543533	0.477708
84	H	0.671178	-2.742069	1.987946
85	H	2.245806	-4.118525	-0.073060
86	H	2.416421	-4.275184	1.685449
87	H	3.524382	-2.016353	1.764588
88	H	3.345424	-1.889770	-0.006136
89	H	6.786486	-4.695964	0.362169
90	H	9.091833	-3.736554	0.170378
91	H	7.478775	0.217893	0.550604
92	H	5.198897	-0.708465	0.743216
93	H	10.629080	2.449602	0.294604
94	H	12.933533	3.407590	0.092550
95	H	14.524490	-0.584454	-0.226659
96	H	12.245694	-1.508583	-0.027023
97	H	16.365700	0.614479	0.516687
98	H	16.177041	0.685395	-1.250695
99	H	17.302312	2.922357	-1.273589
100	H	17.489418	2.852166	0.479077
101	H	18.921199	0.793043	0.240872
102	H	18.734737	0.863118	-1.506132
103	H	20.920346	1.865423	-0.814233
104	H	19.892460	3.108979	-1.546009
105	H	20.080137	3.038482	0.213650

Diastereoisomer 8

Electronic energy (B3LYP): −3416.39213982 Ha.
Lowest frequency: 6.2134 cm^{-1}.
Free enthalpy: −3415.627509 Ha.
Atomic coordinates:

1	C	-1.252504	0.940951	-0.888725
2	C	-0.367851	-0.302499	-1.068408
3	C	1.092684	0.084170	-1.311172
4	O	1.643928	1.002307	-0.700500
5	N	1.746602	-0.669025	-2.238817
6	C	3.157245	-0.494357	-2.539280
7	C	4.037195	-1.377484	-1.652834
8	O	5.388794	-1.141201	-2.052176
9	C	6.388007	-1.824979	-1.430248
10	C	7.688284	-1.572304	-1.904370
11	C	8.782412	-2.201339	-1.330500
12	C	8.599674	-3.119892	-0.278187
13	C	7.298238	-3.407909	0.150293
14	C	6.195953	-2.747889	-0.392849
15	N	9.643175	-3.939508	0.256303
16	N	10.798900	-3.531708	0.535141
17	C	11.184607	-2.156294	0.589720
18	C	10.417641	-1.142517	1.197720
19	C	10.942591	0.130979	1.350689
20	C	12.234856	0.436220	0.882029
21	C	13.010462	-0.572055	0.290611
22	C	12.490846	-1.861468	0.179763
23	O	12.640559	1.719437	1.064674
24	C	13.951399	2.104391	0.624354
25	C	14.132644	3.581635	0.942832
26	C	15.510152	4.105133	0.514439
27	C	15.701422	5.591735	0.833078
28	H	-0.698473	1.651738	-0.273343
29	H	-1.398480	1.430306	-1.859202
30	H	-0.717798	-0.948589	-1.879413
31	H	-0.379989	-0.907118	-0.150289
32	H	1.254276	-1.430817	-2.684614
33	H	3.412641	0.555343	-2.378556
34	H	3.331020	-0.737354	-3.591403
35	H	3.780840	-2.436323	-1.786025
36	H	3.896615	-1.112302	-0.597661
37	H	7.815496	-0.876648	-2.728076
38	H	9.777264	-1.999873	-1.712279
39	H	7.155154	-4.150405	0.929933
40	H	5.204124	-2.972789	-0.019150
41	H	9.422834	-1.359713	1.570605
42	H	10.367535	0.914872	1.833974
43	H	14.015040	-0.371486	-0.061855

44	H	13.097882	-2.655957	-0.244568
45	H	14.705980	1.496348	1.141357
46	H	14.045318	1.920993	-0.454545
47	H	13.341999	4.152439	0.438411
48	H	13.994904	3.730635	2.021812
49	H	16.294420	3.519774	1.013985
50	H	15.643402	3.941750	-0.563889
51	H	16.691342	5.939939	0.517471
52	H	14.950910	6.205624	0.320311
53	H	15.607084	5.780614	1.909416
54	C	-7.695620	0.244274	-2.378385
55	C	-7.896925	-1.137602	-1.754164
56	C	-7.572913	-2.474138	0.270638
57	C	-6.107697	-2.902550	0.186535
58	C	-3.797985	-2.132404	0.401673
59	C	-3.527831	-1.437782	-0.933506
60	C	-3.906763	0.592587	-2.249015
61	C	-5.297285	0.334984	-2.837077
62	C	-6.522208	2.347746	-2.160847
63	C	-7.356209	2.995562	-1.034606
64	C	-9.062149	-0.571295	0.329391
65	C	-8.869048	-0.143012	1.800463
66	C	-5.228248	-2.164703	2.353602
67	C	-4.627142	-0.973815	3.131620
68	C	-2.609662	0.759591	-0.168689
69	C	-3.122738	2.154920	0.331723
70	H	-7.792361	0.154420	-3.471626
71	N	-6.403584	0.881415	-2.022382
72	N	-7.832167	-1.120556	-0.276899
73	N	-5.190086	-1.977902	0.888426
74	N	-3.698179	0.033670	-0.892041
75	O	-7.438255	2.331990	0.069386
76	O	-7.856821	4.104894	-1.251004
77	O	-7.659081	0.119829	2.164514
78	O	-9.874946	-0.053279	2.513403
79	O	-4.610571	0.160815	2.516318
80	O	-4.236157	-1.176435	4.286493
81	O	-4.400370	2.324159	0.374512
82	O	-2.288435	2.995764	0.686209
83	Gd	-5.938850	0.592630	0.615325
84	H	-8.494516	0.913048	-2.050880
85	H	-7.129861	-1.825683	-2.118889
86	H	-8.862534	-1.539343	-2.100557
87	H	-8.189830	-3.225747	-0.246365
88	H	-7.893715	-2.474376	1.314640
89	H	-6.018034	-3.923117	0.591116
90	H	-5.797033	-2.959515	-0.859673
91	H	-3.546105	-3.199273	0.295761
92	H	-3.131666	-1.732155	1.168872

93	H	-2.524770	-1.726235	-1.268709
94	H	-4.214851	-1.826778	-1.688701
95	H	-3.169060	0.193037	-2.961439
96	H	-3.732839	1.669100	-2.191799
97	H	-5.318843	0.758160	-3.854263
98	H	-5.461838	-0.739159	-2.951830
99	H	-6.950425	2.625787	-3.134894
100	H	-5.524317	2.791542	-2.103693
101	H	-9.895983	-1.285683	0.266062
102	H	-9.359719	0.326523	-0.219742
103	H	-6.270817	-2.251699	2.673135
104	H	-4.714469	-3.089702	2.653315
105	H	-2.426337	0.197035	0.754348

Supplementary Section 13

Characterisation of *trans*-GdAzo aggregation and interaction with cell membrane models

13.1 - Sample preparation and methods for SAXS and DSC experiments

DPPC (1,2-dipalmitoyl-*sn*-glycero-3-phosphocholine) was purchased from Avanti Polar Lipids (Alabaster, Alabama, USA). Methanol (MeOH, HiPerSolv® CHROMANORM®) was obtained from VWR. Solutions of DPPC (27 mM, 10 wt%, MeOH) and *trans*-**GdAzo** (5 mM, MeOH) were mixed to obtain the chosen molar fractions $x_{trans\text{-}\mathbf{GdAzo}}(\%) = 100 \times n_{trans\text{-}\mathbf{GdAzo}}/(n_{trans\text{-}\mathbf{GdAzo}} + n_{\mathrm{DPPC}})$ of 0.1, 1, 3, 7, 10 or 15%. The solvent was evaporated under a nitrogen stream, after which the samples were dried overnight under vacuum to remove residual MeOH. The dry films were hydrated with phosphate buffered saline (PBS) in excess (90 wt% PBS). The suspensions were heated at 50 °C for 1 h with vortex mixing every 15 min in order to ensure homogeneous hydration of the samples. They were then allowed to equilibrate at room temperature (rt) for at least 24 h. Aliquots of the suspensions were loaded into either aluminum pans for DSC measurements or quartz capillaries for X-ray experiments.

Small-angle X-ray scattering (SAXS) experiments were performed on the SWING beamline at the SOLEIL synchrotron (St-Aubin, France). The scattering intensity I(q) was reported as a function of the scattering vector q, defined as $q = 4\,\pi\,\sin\theta/\lambda$ where 2θ is the scattering angle and λ the X-ray wavelength. Samples were loaded into quartz capillaries (1.5 mm diameter, Glass-Muller, Germany) and sealed to prevent evaporation. Capillaries were placed in a sample holder enabling temperatures to be controlled between 5 and 60 °C. Data were recorded at 12 keV in the scattering vector q-range $0.004 < q < 0.47$ Å^{-1}, using a bi-dimensional Eiger detector. The beamline software Foxtrot was used for data collection and processing. For each sample, 30 frames of 0.5 s were recorded and averaged. Buffer scattering was subtracted from the sample scattering. For all samples, the scattering intensity was normalised with respect to the incident beam intensity, acquisition time and sample transmission.

Differential scanning calorimetry (DSC) experiments were carried out using a Perkin-Elmer DSC Diamond apparatus equipped with an Intracooler 2P cooling device and connected to a Pyris Thermal Analysis Software System (Version 9.1). Aluminum pans were filled with 10 µL of suspension and sealed. The samples were first cooled from 25 to 10 °C at a constant rate $|dT/dt| = 5$ °C.min^{-1} and allowed to equilibrate for 5 min at this temperature. The samples were then heated from 10 to 60 °C at 5° C.min^{-1} and cooled down to 10 °C at the same rate. Two heating-cooling cycles were performed on each sample to assess the reversibility and reproducibility of the transitions. The transition temperatures were taken at the onset of the transitions (T_{onset}). For the main transition, the offset temperature (T_{offset}), corresponding to completion of the transition, was also reported. The enthalpy, ΔH, of each transition was obtained from the area under the corresponding peak and normalised with respect to the DPPC content.

13.2 - SAXS characterisation of *trans*-GdAzo micelles

The SAXS pattern of *trans*-**GdAzo** suspensions (24 mM in PBS) could be well described by a model of core-shell ellipsoidal aggregate, the core corresponding to the hydrophobic *trans*-azobenzene moiety and the shell to the hydrophilic Gd-chelate, which has a higher electron density (Fig. 71). The curve was fitted using the SASFit software [31]. The parameters were the core semi-axe lengths, the shell width and the electronic densities (or scattering length densities) of the core, the shell and the solvent (water). The mean electronic densities of the core and the shell were retrieved from DFT results (0.373 and 0.552 e/Å^3 for the core and the shell respectively, cf. Supplementary Section 12). The lengths for the short and long semi-axes of the core were 16.6 Å and 29 Å, respectively, and the shell thickness was 7.9 Å. These values are in reasonable agreement with the dimensions of the *trans*-**GdAzo** molecule, as shown by DFT calculation.

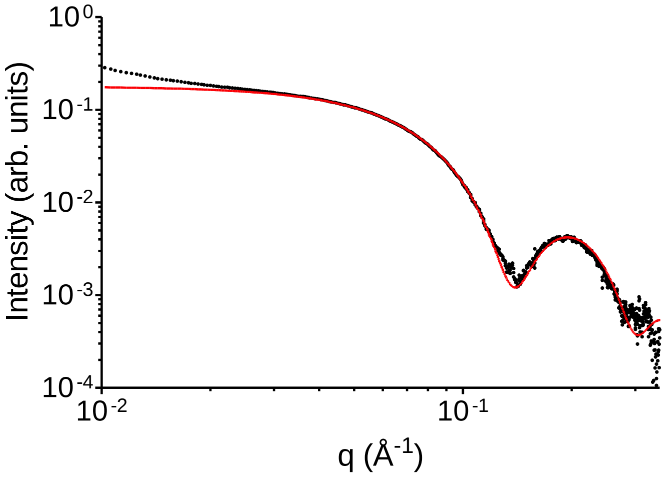

Fig. 71. SAXS pattern of the *trans*-**GdAzo** micelles recorded at rt (black). The experimental curve is fitted to that of an ellipsoidal core-shell micelle using SASFit software (red). The lengths for short and long semi-axes are 16.6 Å and 29 Å, respectively, and the shell thickness is 7.9 Å.

13.3 - Study of *trans*-GdAzo/DPPC bilayer interactions using SAXS and DSC

DPPC bilayers were used as a simple model of a target biomembrane. Any interaction of *trans*-**GdAzo** with DPPC should lead to a modification of the structural and thermal parameters of the phospholipid phase, as deduced from SAXS and DSC measurements.

In medium with excess water, the zwitterionic molecules of DPPC form bilayers whose structure and long-range organisation depend on temperature. As the temperature is increased from rt, DPPC displays two successive phase transitions: the pretransition, T_p, between the gel phase L_{β}' and the gel ripple phase P_{β}', and the main transition T_m, between P_{β}' and the liquid crystalline phase L_{α}. In the gel phases the aliphatic chains are fully extended, whereas they have a liquid-like conformation above the main transition temperature. The periodic stacking of bilayers in these lamellar phases gives rise to Bragg peaks in SAXS patterns. The lamellar spacing (d), corresponding to the sum of the bilayer and water layer thicknesses, is deduced from the peak positions using Bragg's law: $n \times d = 2\pi/q_n$ where q_n is the position of the n-order peak.

The mixing of *trans*-**GdAzo** with DPPC in excess buffer led to a progressive shift towards smaller scattering vectors q of the Bragg peaks (Fig. 72). At rt, the d-spacing increased from d = 64.3 Å in DPPC to d = 80 Å in DPPC containing 7% of *trans*-**GdAzo** and then remained constant up to 15% of *trans*-**GdAzo**. This change in d-spacing was accompanied by a broadening and a decrease in intensity of the Bragg peaks, indicating a more disordered lamellar phase involving fewer bilayers. A broad maximum centred on $q \approx 0.12$ Å^{-1}, compatible with scattering from micelles, was also clearly observed for *trans*-**GdAzo** molar fraction ≥ 3%. These results suggest the coexistence of stacked bilayers and micelles, both of which would be mixed structures containing *trans*-**GdAzo** molecules. This phase coexistence was further supported by the visible sedimentation of the lamellar phase over time. Only the broad scattering ascribed to micelles without long-range order was observed in SAXS curves recorded on the transparent supernatant of *trans*-**GdAzo**-rich mixtures (see for example Fig. 73). Heating at 55 °C, above the main transition temperature T_m of DPPC, led to the irreversible growth of the micellar phase at the expense of the lamellar phase, as indicated by the increased height of supernatant in capillaries. The intensity of the Bragg peaks, characteristic of the lamellar phase, strongly decreased for *trans*-**GdAzo** molar fraction ≥ 3% (see for example Fig. 74). Interestingly, the d-spacing did not change.

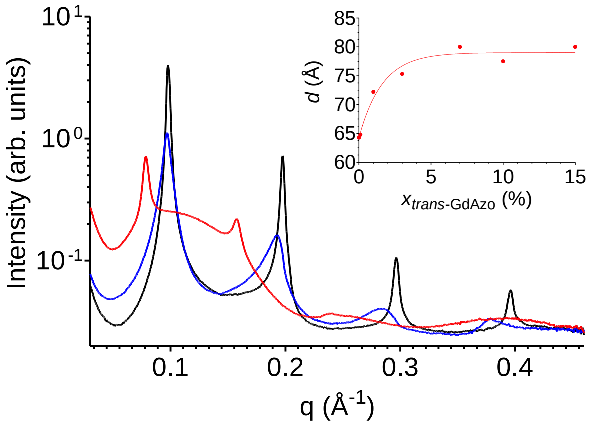

Fig. 72. Influence of the molar fraction of *trans*-GdAzo. SAXS patterns of selected samples recorded at rt (black: $x_{trans\text{-}\mathbf{GdAzo}}$ = 0% (pure DPPC), blue: $x_{trans\text{-}\mathbf{GdAzo}}$ = 0.1%, red: $x_{trans\text{-}\mathbf{GdAzo}}$ = 7%). Inset: Variation of the d-spacing as a function of *trans*-**GdAzo** molar fraction.

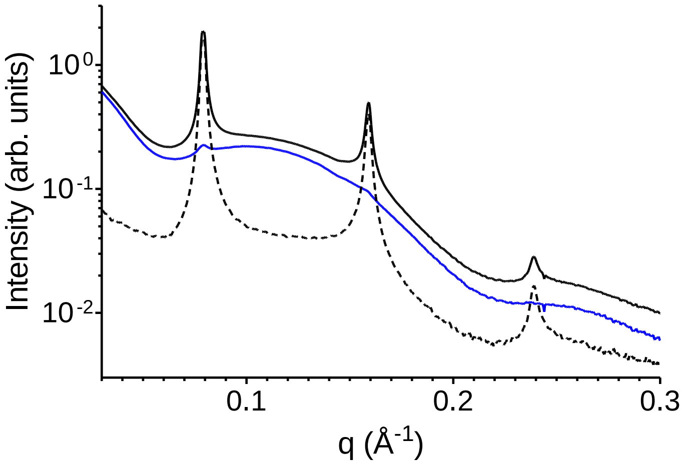

Fig. 73. Evidence of the coexistence of a lamellar phase and a micellar phase in *trans*-GdAzo/DPPC mixtures ($x_{trans\text{-}GdAzo}$ = 15%). Blue curve: SAXS pattern of the supernatant (micellar phase). Black curve: SAXS pattern of the phase at the bottom of the capillary: the Bragg peaks, indicating regular stacking of mixed bilayers, are superimposed on the broad scattering of the micellar phase, as shown by the dotted curve obtained by subtracting the pattern of the supernatant.

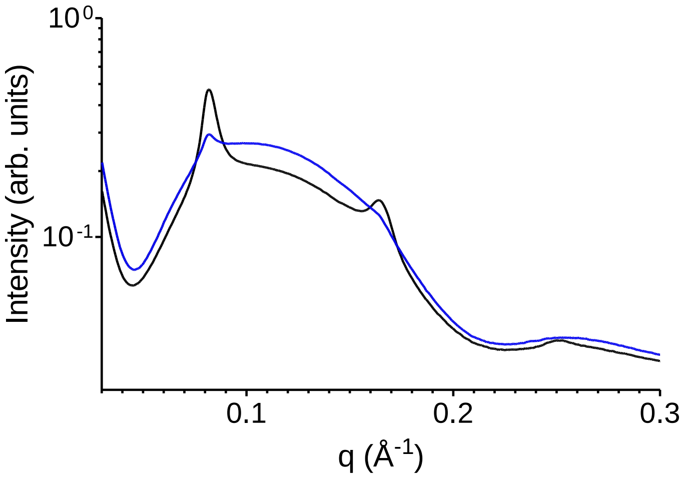

Fig. 74. Influence of heating. SAXS patterns of the DPPC/*trans*-**GdAzo** mixture ($x_{trans\text{-}GdAzo}$ = 3%) recorded at rt before (black) and after (blue) *in situ* heating at 55 °C.

The SAXS pattern of the micellar phase contained information about the shape and size of the micelles. It was characteristic of planar objects, as shown by the q^{-2} dependence of the scattered intensity in the intermediate q-range ($q \approx 0.013$-0.034 Å^{-1}) (Fig. 75). The mean

micelle diameter, D, could be deduced from the low *q* region (Guinier region). In the 0.004-0.013 Å^{-1} *q*-range, the SAXS curve could be modelled by homogeneous discs, using the SASFit software. The mean diameter of the discs was D = 34 nm for 3%, D = 28 nm for 10% and D = 26.5 nm for 15% of *trans*-**GdAzo**.

The pair-distance distribution functions, *p*(*r*), containing information about the distribution of scattering contrast over the volume of the micelles, were evaluated using the GNOM software (Fig. 75) [32,33]. They were consistent with those of DPPC nanodiscs reported in the literature which display a characteristic minimum at $r \approx 30$ Å, corresponding to the centre of the bilayer, and a broad maximum at about 60 Å, reflecting the bilayer thickness [34,35]. Indeed, the bilayer core and the polar heads have lower and higher electron densities than water, respectively.

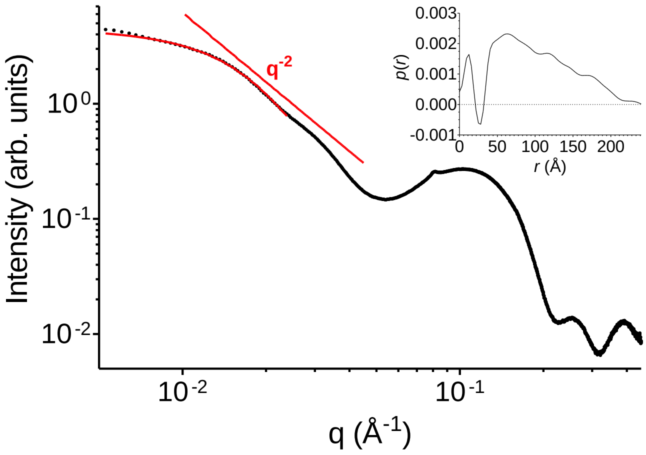

Fig. 75. **SAXS characterisation of the micellar phase**. SAXS pattern (black) of the micellar phase formed in the DPPC/*trans*-**GdAZo** mixture ($x_{trans\text{-}\mathbf{GdAzo}}$ = 10%). The first part of the curve is fitted using a homogeneous disc model 28 nm in diameter (red). In the intermediate *q*-range, the scattered intensity decreases as q^{-2}, which is characteristic of a planar object. Inset: corresponding distance distribution function determined using GNOM software.

The location of *trans*-**GdAzo** in phospholipid bilayers was further addressed by comparing the electron density profiles of bilayers of DPPC, on one hand, and DPPC with 0.1 mol% *trans*-**GdAzo** on the other hand (Fig 76). The DPPC profile reflected the electron-rich

phosphatidylcholine headgroups and electron-poor hydrocarbon chains, in agreement with previous studies[36]. The distance between the phosphate groups, d_{HH}, deduced from the position of the two maxima was 44.0 Å in pure DPPC. The addition of *trans*-**GdAzo** slightly shifted these maxima away from the bilayer centre (d_{HH}'= 46.4 Å) and increased their intensity. These results are consistent with the insertion of *trans*-**GdAzo** between DPPC chains, with the electron-rich Gd-chelate moiety slightly protruding in water. Of note, the thickness of the bilayer hydrophobic core ($2D_C$ = 34.4 and 28.5 Å at 20 and 50 °C respectively)[36] enables the insertion of the hydrophobic part of *trans*-**GdAzo** whose length can be estimated at 21.8-22.6 Å, according to the molecular model (cf. Supplementary Section 12).

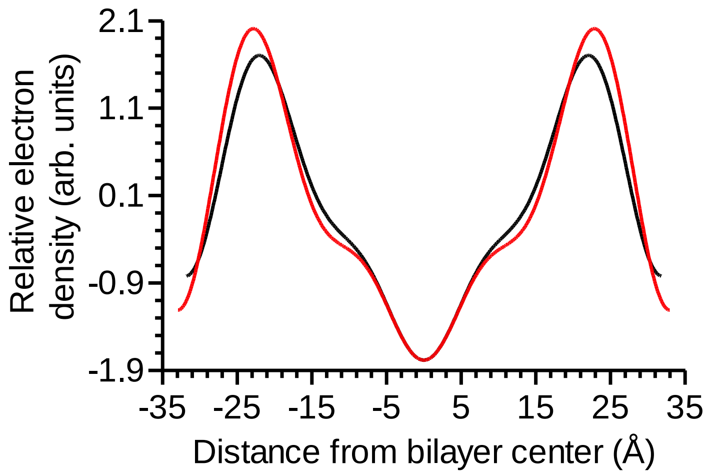

Fig. 76. Insertion of *trans*-GdAzo in DPPC model membrane. Electronic density of the DPPC model phospholipid membrane in the presence of *trans*-**GdAzo** (0.1%) at rt extracted from SAXS experiments (black: $x_{trans\text{-GdAzo}}$ = 0% (pure DPPC), red: $x_{trans\text{-GdAzo}}$ = 0.1%).

These systems were further characterised by DSC (Fig. 77). The weak endotherm corresponding to the pretransition of pure DPPC (T_{onset} = 35.0 °C, ΔH = 8.2 J.g^{-1}) shifted to higher pretransition temperatures T_p for *trans*-**GdAzo** molar fractions of 0.1 and 1% and then to slightly lower T_p upon increasing the *trans*-**GdAzo** molar fraction from 1 to 7%. The pretransition enthalpy was reduced by a factor of about 2 for molar fractions of *trans*-**GdAzo** between 0.1 and 7%; the pretransition was no longer detectable for higher molar fractions. The main transition (T_{onset} = 41.4 °C, ΔH = 49.0 J.g^{-1}), arising from the melting of the DPPC aliphatic chains, was dramatically broadened in the presence of *trans*-**GdAzo**. It moved first

towards slightly lower temperatures T_m for *trans*-**GdAzo** molar fractions between 0.1 and 1% and then to higher T_m for molar fractions higher than 1%. The main transition enthalpy was slightly reduced (by about 8%) for *trans*-**GdAzo** molar fractions higher than 7%. This behaviour demonstrates that, at temperatures below T_m of DPPC, the majority of the DPPC aliphatic chains retained an ordered state in DPPC/*trans*-**GdAzo** mixtures. The broadening of the transition indicated a decrease in the cooperativity of the gel-to-liquid phase transition. These findings are in line with previous studies showing that nanodiscs displayed broader phase transitions and melting temperatures shifted a few degrees Celsius higher than phosphatidylcholine multilamellar vesicles [35,37]. However, for *trans*-**GdAzo** molar fractions lower than 1%, only insertion of the *trans*-**GdAzo** molecule in the bilayer was observed.

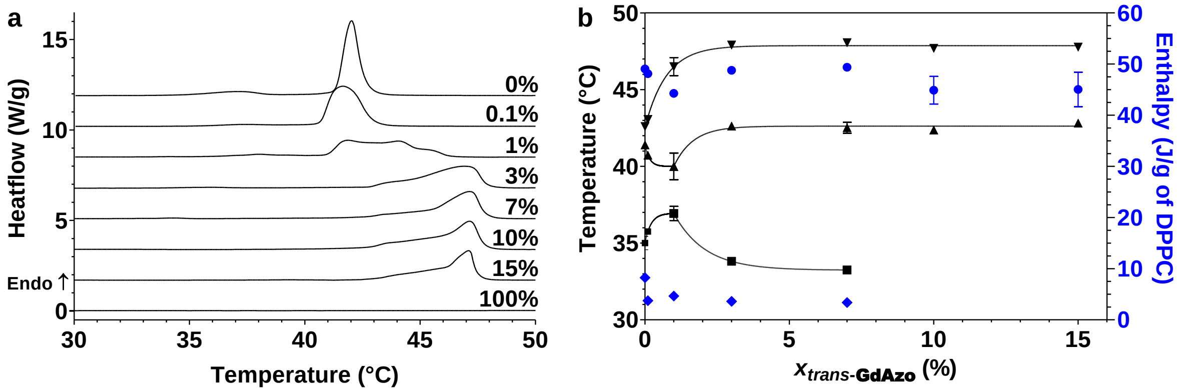

Fig. 77. DSC study of the interaction of *trans*-GdAzo with DPPC model membranes. a, DSC thermograms of fully hydrated (90 wt% PBS) DPPC/*trans*-**GdAzo** mixtures (increasing molar fraction from top to bottom, the molar fraction is expressed in %) obtained during heating at a rate of 5 °C.min^{-1}. **b**, Variation of the onset temperature of the pretransition (■) and of the onset (▲) and endset (▼) temperatures of the main transition; and of the enthalpies of the pretransition (◆) and of the main transition (●) as a function of *trans*-**GdAzo** molar fraction (n=3). The means ± standard deviations are reported.

Taken together, these results suggest that mixed micelles are disc-shaped nanostructures formed by a bilayer core whose hydrophobic edge is shielded from water by *trans*-**GdAzo** molecules, coexisting with mixed multilamellar vesicles. The solubilisation of DPPC bilayers by *trans*-**GdAzo** is likely to be a kinetic process enhanced by heating, involving the progressive formation of nanodiscs. This process may result from reorganisation within mixed bilayers.

<u>Supplementary Section 14</u>

In vitro characterisation on cancer cells

General information

Phosphate buffered saline (PBS), Dulbecco's Modified Eagle Medium-high glucose (DMEM), Roswell Park Memorial Institute medium (RPMI), penicillin, streptomycin, trypan blue solution (0.4%), dimethylsulfoxyde (DMSO), thiazolyl blue tetrazolium bromide (MTT), *S*-(4-Nitrobenzyl)-6-thioinosine (NBMPR), uranyl acetate and osmium were purchased from Sigma Aldrich (France). Fetal bovine serum (FBS) was purchased from Gibco (France), propidium iodide (PI) was purchased from Thermo Fisher Scientific (France), and gemcitabine hydrochloride (Gem) was purchased from Sequoia Research Products Ltd (UK). Epon resin and 2,2-dimethoxypropane (DMP-30) hardener were purchased from Electron Microscopy Sciences (Hatfield, UK). The radiolabelled [5'-^{3}H]-gemcitabine hydrochloride ([^{3}H]Gem) was obtained from Moravek Biochemicals (USA).

Cell lines

Human pancreatic cancer cells (PANC-1) and human acute lymphoblastic leukemia cells (CCRF-CEM) were purchased from ATCC (France) and maintained as recommended. The Gem-resistant human acute lymphoblastic leukemia cells (CCRF-CEM ARAC-8C, non-expression of hENT-1 receptor) were provided by Dr. Buddy Ullmann (Oregon Health Sciences University). Briefly, PANC-1 cells were maintained in DMEM supplemented with 10% v/v heat-inactivated FBS. CCRF-CEM and CCRF-CEM ARAC-8C cells were maintained in RPMI supplemented with 10% v/v heat-inactivated FBS. All media were further supplemented with penicillin (50 $U.mL^{-1}$) and streptomycin (50 $U.mL^{-1}$). Cells were maintained in a humid atmosphere at 37 °C with 5% CO_2. Cells were used below passage 18 after thawing and harvested at a confluence of 70–80%.

14.1 - Toxicity of *cis*-GdAzo (cell viability by MTT assay)

The toxicity of the *cis*-**GdAzo** compound on cancer cells (PANC-1) was assessed by using the MTT assay.

Cells were seeded (6,000 cells in 100 µL/well) in 96-well microplate (TPP from Thermo Fisher Scientific, France) and maintained in culture medium in a humid atmosphere at 37 °C with 5% CO_2. 24 h post-seeding, culture medium was removed before the addition of *cis*-**GdAzo** (100 µL, final concentration 0.01-500 µM, culture medium without phenol red) and cells were maintained in a humid atmosphere at 37 °C with 5% CO_2 for 1 h. Then, culture medium was discarded and replaced by fresh medium (200 µL). Cells were maintained in a humid atmosphere at 37 °C with 5% CO_2 for 4 days before addition of MTT solution (20 µL/well, stock solution 5 mg/mL in PBS). After 45 min incubation in the dark in a humid atmosphere at 37 °C with 5% CO_2, the culture medium was removed and replaced by DMSO (200 µL). After 5 min stirring in the dark, absorbance (570 nm) was recorded. The absorbance data were normalised with the absorbance of a control experiment (*cis*-**GdAzo** replaced by cell culture medium without phenol red) to obtain the cell viability percentage for each *cis*-**GdAzo** concentration (triplicate). The experiment was repeated 3 times independently (Fig. 78). *cis*-**GdAzo** was obtained by UV irradiation (365 nm, 0.817 mW.cm^{-2}, 30 min) of *trans*-**GdAzo** (105 µL, 1 mM, culture medium without phenol red, *cis*-**GdAzo** 94%) in 96-well microplate.

From this experiment, we could not detect any toxicity of the *cis*-**GdAzo** compound after 1 h exposition on cancer cells.

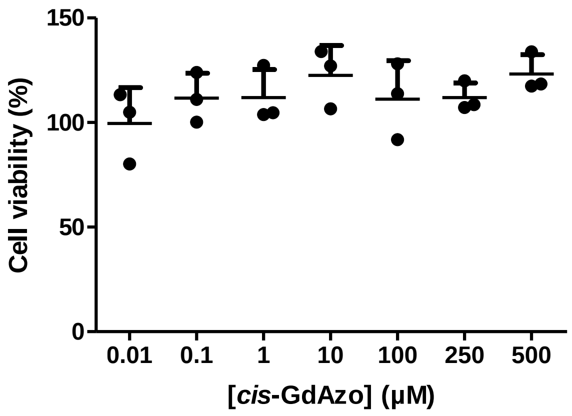

Fig. 78. *cis*-GdAzo toxicity by MTT assay. The cell viability is reported as means ± standard deviations (n=3).

14.2 - Cell permeabilisation of *trans*-GdAzo without irradiation (confocal microscopy)

PANC-1 cells were seeded (33,000 cells in 200 µL/well) in imaging chamber (micro-slide 8 well ibiTreat, Ibidi, Germany) and maintained in culture medium in a humid atmosphere at 37 °C with 5% CO_2. 24 h post-seeding, culture medium was carefully replaced by FBS and phenol red-free culture medium (95 µL) and PI (5 µL, final concentration 1 µM) was added in the medium. 15 min after addition, a first set of images was acquired. Then, *cis*-**GdAzo** or *trans*-**GdAzo** (100 µL, final concentration 0, 250, 500 or 1,000 µM, FBS and phenol red-free culture medium) was carefully introduced in the medium. The imaging chamber was kept in the dark and images were acquired each 5 min during 1 h. *cis*-**GdAzo** was obtained by UV irradiation (365 nm, 0.817 $mW.cm^{-2}$, 30 min) of *trans*-**GdAzo** (105 µL, 0.5, 1 or 2 mM, FBS and phenol red-free culture medium, *cis*-**GdAzo** 95 ± 2%) in 96-well microplate. Cells were observed with an inverted Leica TCS SP8 microscope gated-STED (Leica, Germany) using a HC PL Fluotar CS2 10x/0.30 dry objective lens. The instrument was equipped with a WLL Laser (538 nm excitation wavelength). The red fluorescence emission of PI was collected with a 560-650 nm wide emission slits and transmission images were acquired with the same laser line and a PMT-trans detector. The pinhole was set at 1.0 Airy unit (73.19 µm diameter). 16-bit numerical images were acquired with the Leica SP8 LAS X software (Version 2.0.1; Leica, Germany). The images (Figs. 79-85) were processed with ImageJ software[38] (Version 1.50i).

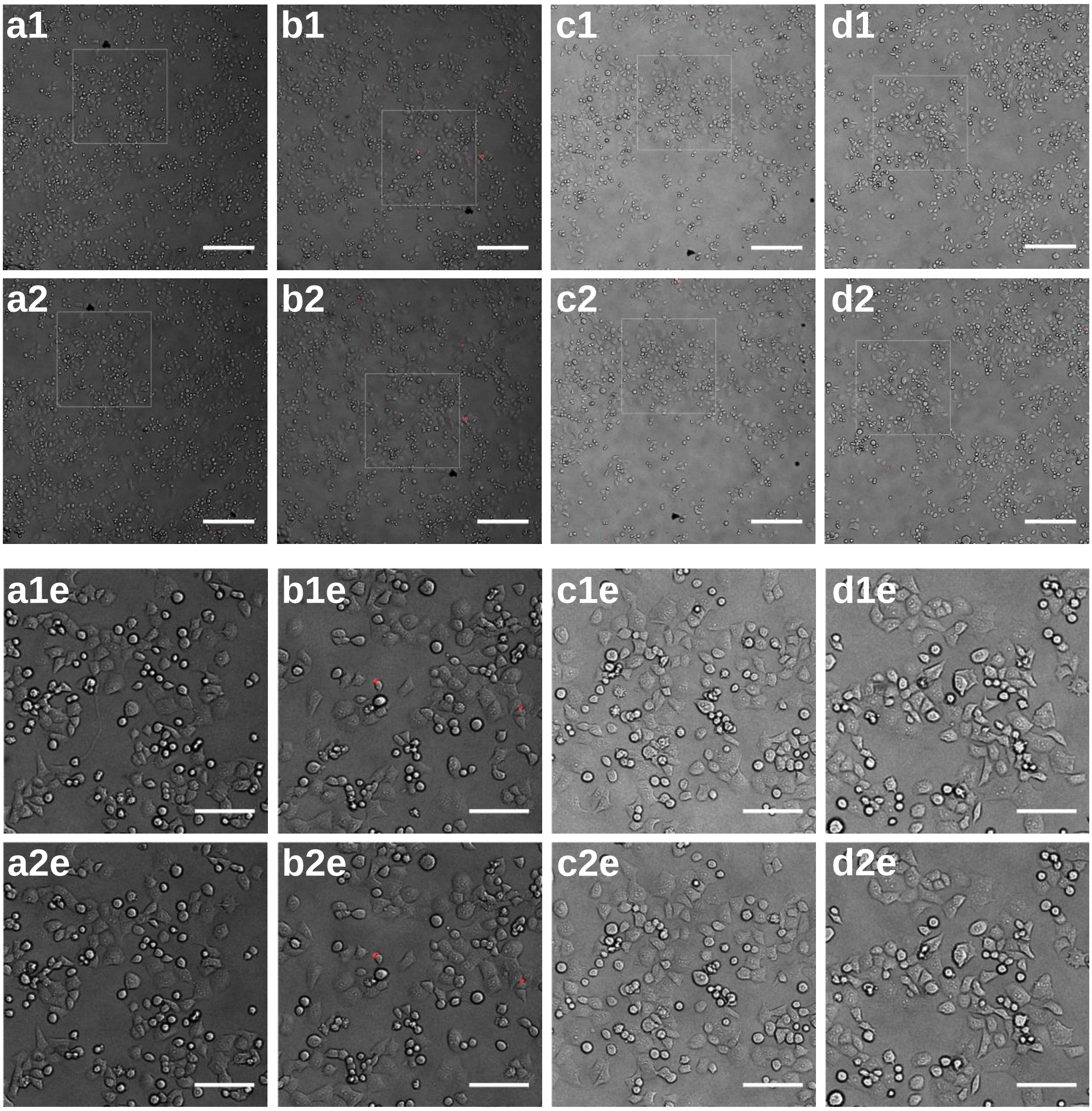

Fig. 79. Cell membrane permeabilisation in a control experiment without GdAzo using confocal microscopy. Light microscopy imaging of human pancreatic cancer cells (PANC-1) in the presence of PI (1 µM) before (**a1-d1**, quadruplicate) and 30 min after (**a2-d2**, quadruplicate) the introduction of FBS and phenol red-free culture medium. Overlap of bright field and fluorescence (ex. 538 nm, em. 560-650 nm) images is presented. PI appears in red on these images. Wide-angle images (scale bar = 300 µm) and a higher magnification on the same images (**e**, white square, scale bar = 125 µm) are presented.

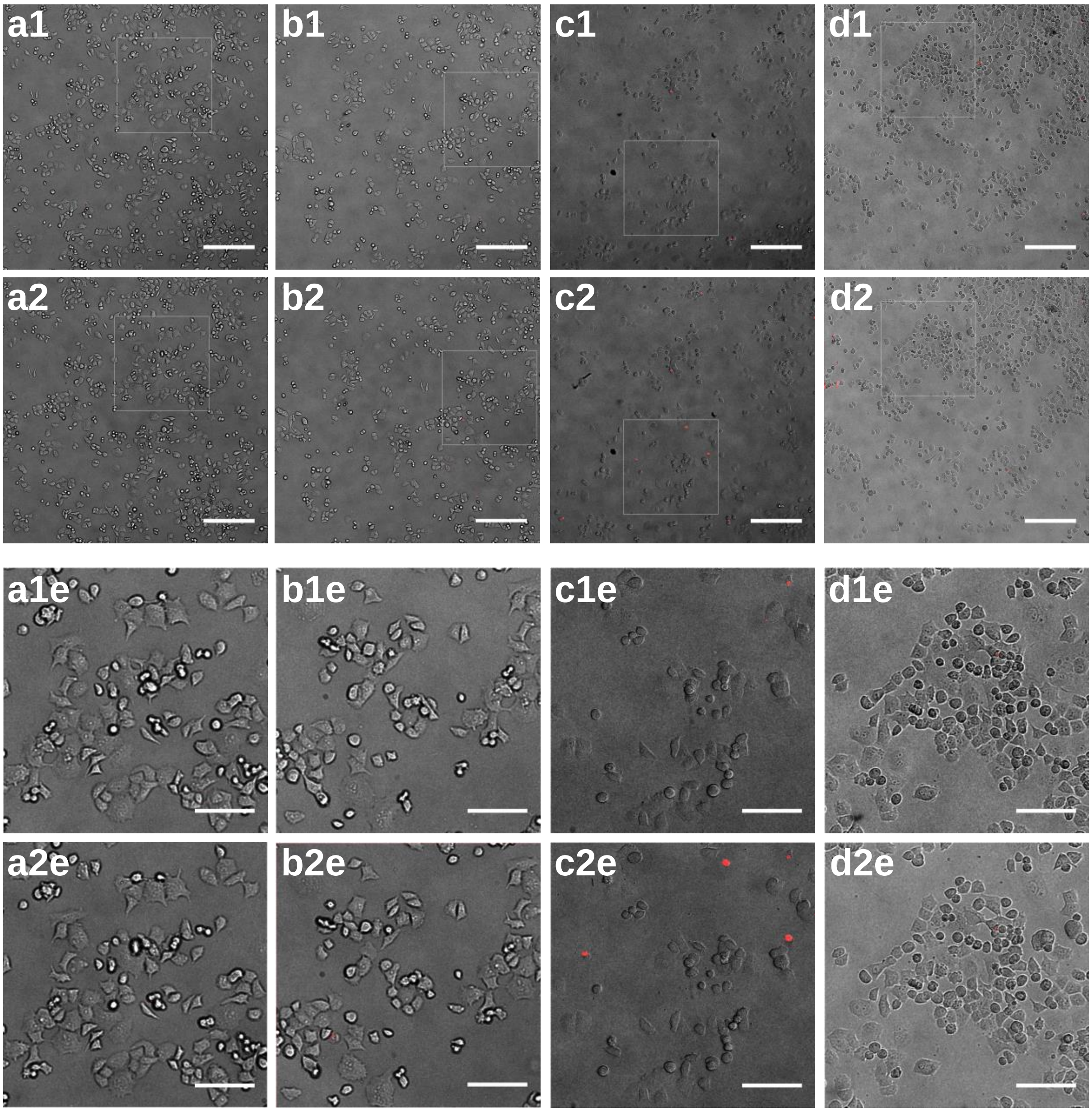

Fig. 80. Cell membrane permeabilisation induced by *cis*-GdAzo (250 µM) using confocal microscopy. Light microscopy imaging of human pancreatic cancer cells (PANC-1) in the presence of PI (1 µM) before (**a1-d1**, quadruplicate) and 30 min after (**a2-d2**, quadruplicate) the introduction of the inactive *cis*-**GdAzo** compound (final concentration 250 µM). Overlap of bright field and fluorescence (ex. 538 nm, em. 560-650 nm) images is presented. PI appears in red on these images. Wide-angle images (scale bar = 300 µm) and a higher magnification on the same images (**e**, white square, scale bar = 125 µm) are presented.

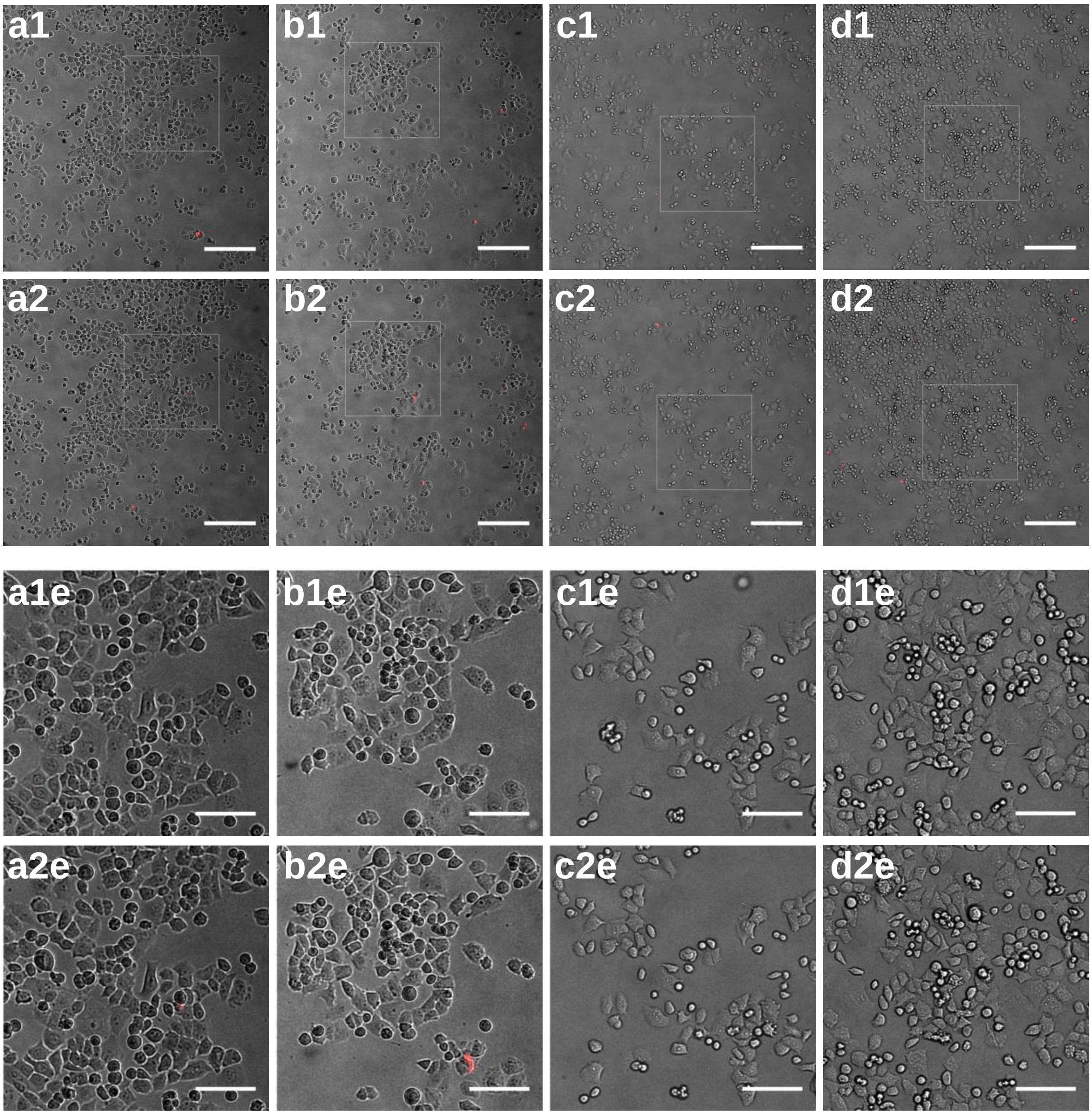

Fig. 81. Cell membrane permeabilisation induced by *cis*-GdAzo (500 µM) using confocal microscopy. Light microscopy imaging of human pancreatic cancer cells (PANC-1) in the presence of PI (1 µM) before (**a1-d1**, quadruplicate) and 30 min after (**a2-d2**, quadruplicate) the introduction of the inactive *cis*-**GdAzo** compound (final concentration 500 µM). Overlap of bright field and fluorescence (ex. 538 nm, em. 560-650 nm) images is presented. PI appears in red on these images. Wide-angle images (scale bar = 300 µm) and a higher magnification on the same images (**e**, white square, scale bar = 125 µm) are presented.

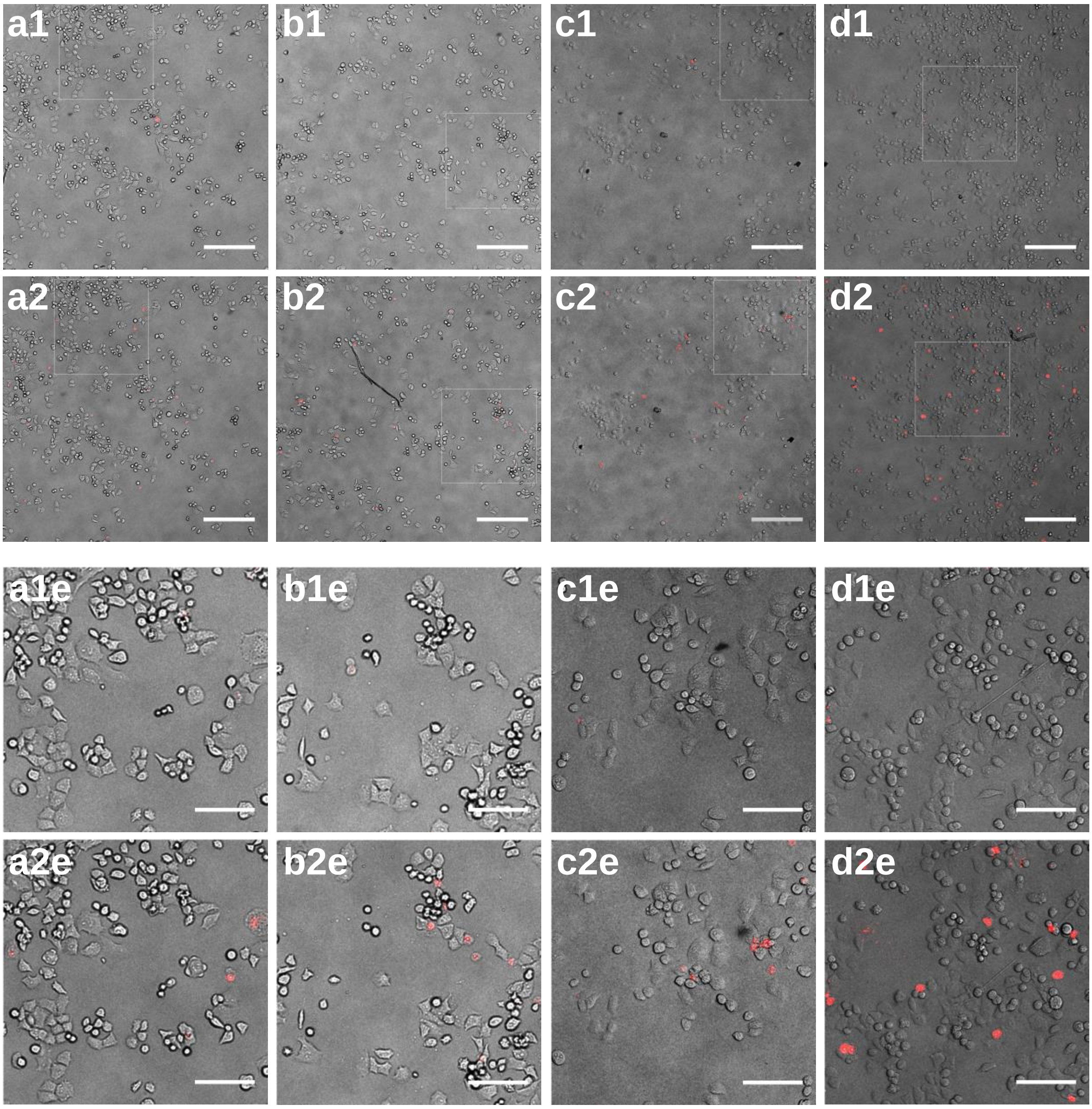

Fig. 82. Cell membrane permeabilisation induced by *cis*-GdAzo (1 mM) using confocal microscopy. Light microscopy imaging of human pancreatic cancer cells (PANC-1) in the presence of PI (1 µM) before (**a1**-**d1**, quadruplicate) and 30 min after (**a2-d2**, quadruplicate) the introduction of the inactive *cis*-**GdAzo** compound (final concentration 1 mM). Overlap of bright field and fluorescence (ex. 538 nm, em. 560-650 nm) images is presented. PI appears in red on these images. Wide-angle images (scale bar = 300 µm) and a higher magnification on the same images (**e**, white square, scale bar = 125 µm) are presented.

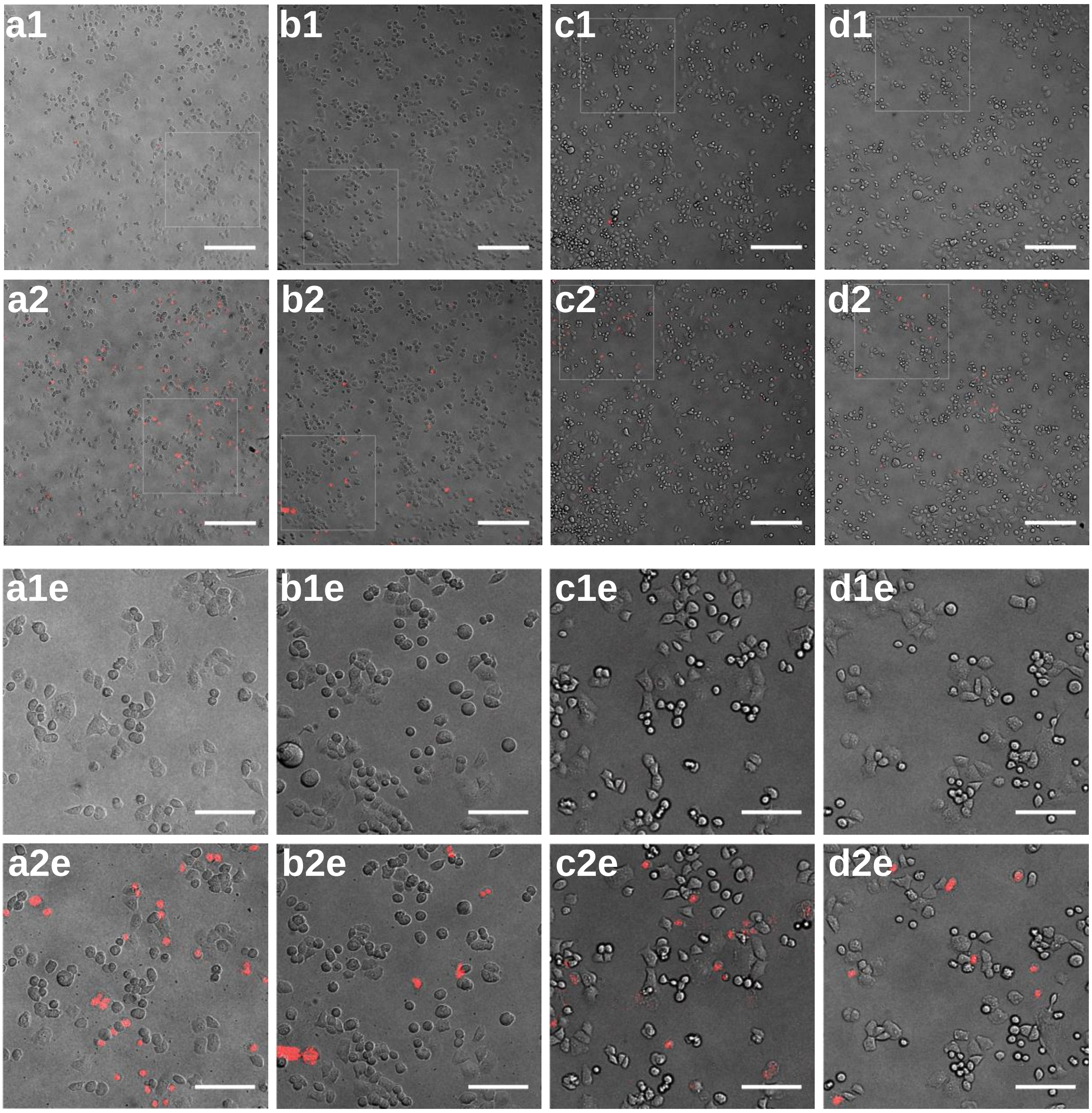

Fig. 83. Cell membrane permeabilisation induced by *trans*-GdAzo (250 µM) using confocal microscopy. Light microscopy imaging of human pancreatic cancer cells (PANC-1) in the presence of PI (1 µM) before (**a1-d1**, quadruplicate) and 30 min after (**a2-d2**, quadruplicate) the introduction of the active *trans*-**GdAzo** compound (final concentration 250 µM). Overlap of bright field and fluorescence (ex. 538 nm, em. 560-650 nm) images is presented. PI appears in red on these images. Wide-angle images (scale bar = 300 µm) and a higher magnification on the same images (**e**, white square, scale bar = 125 µm) are presented.

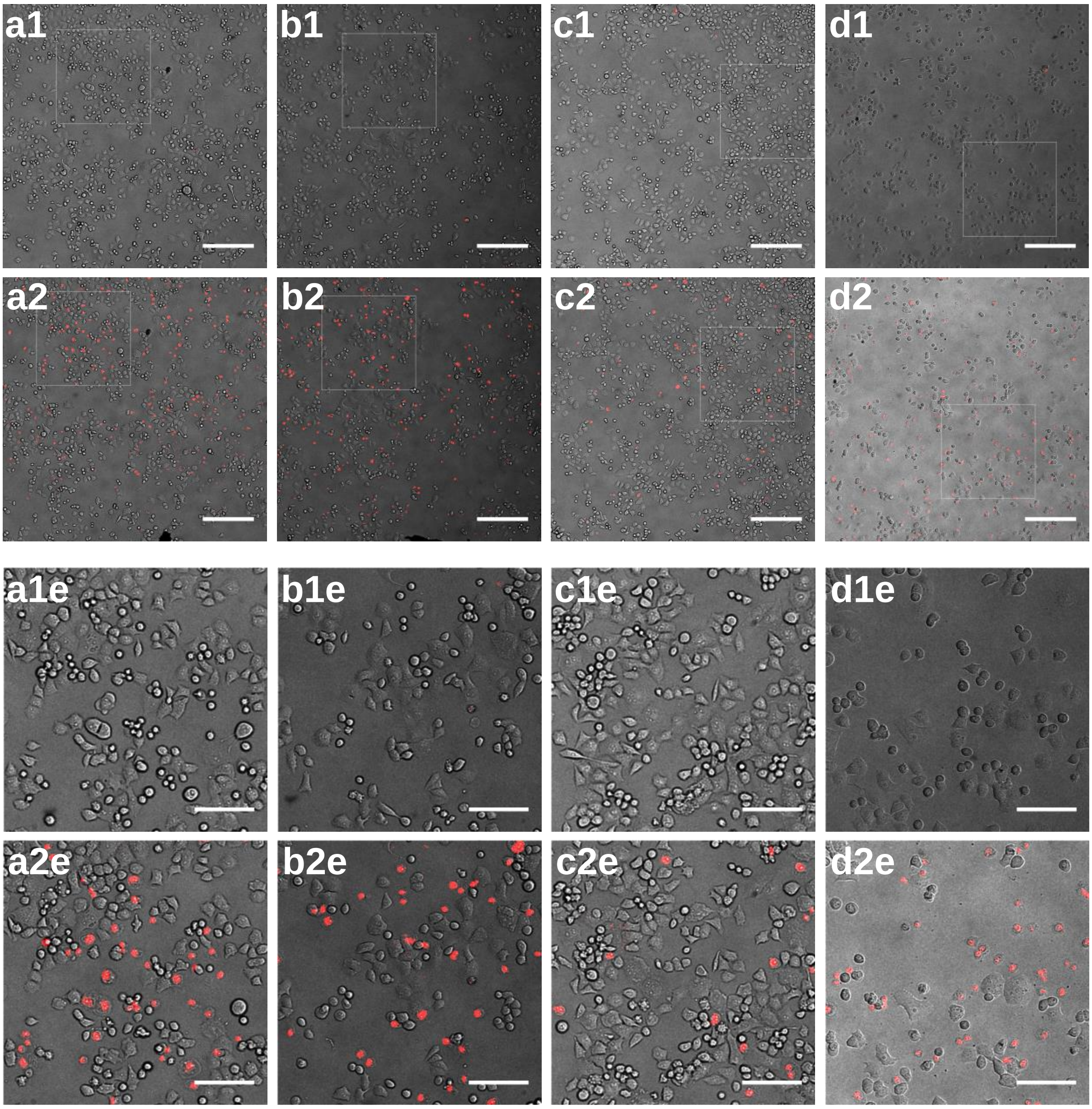

Fig. 84. Cell membrane permeabilisation induced by *trans*-GdAzo (500 µM) using confocal microscopy. Light microscopy imaging of human pancreatic cancer cells (PANC-1) in the presence of PI (1 µM) before (**a1-d1**, quadruplicate) and 30 min after (**a2-d2**, quadruplicate) the introduction of the active *trans*-**GdAzo** compound (final concentration 500 µM). Overlap of bright field and fluorescence (ex. 538 nm, em. 560-650 nm) images is presented. PI appears in red on these images. Wide-angle images (scale bar = 300 µm) and a higher magnification on the same images (**e**, white square, scale bar = 125 µm) are presented.

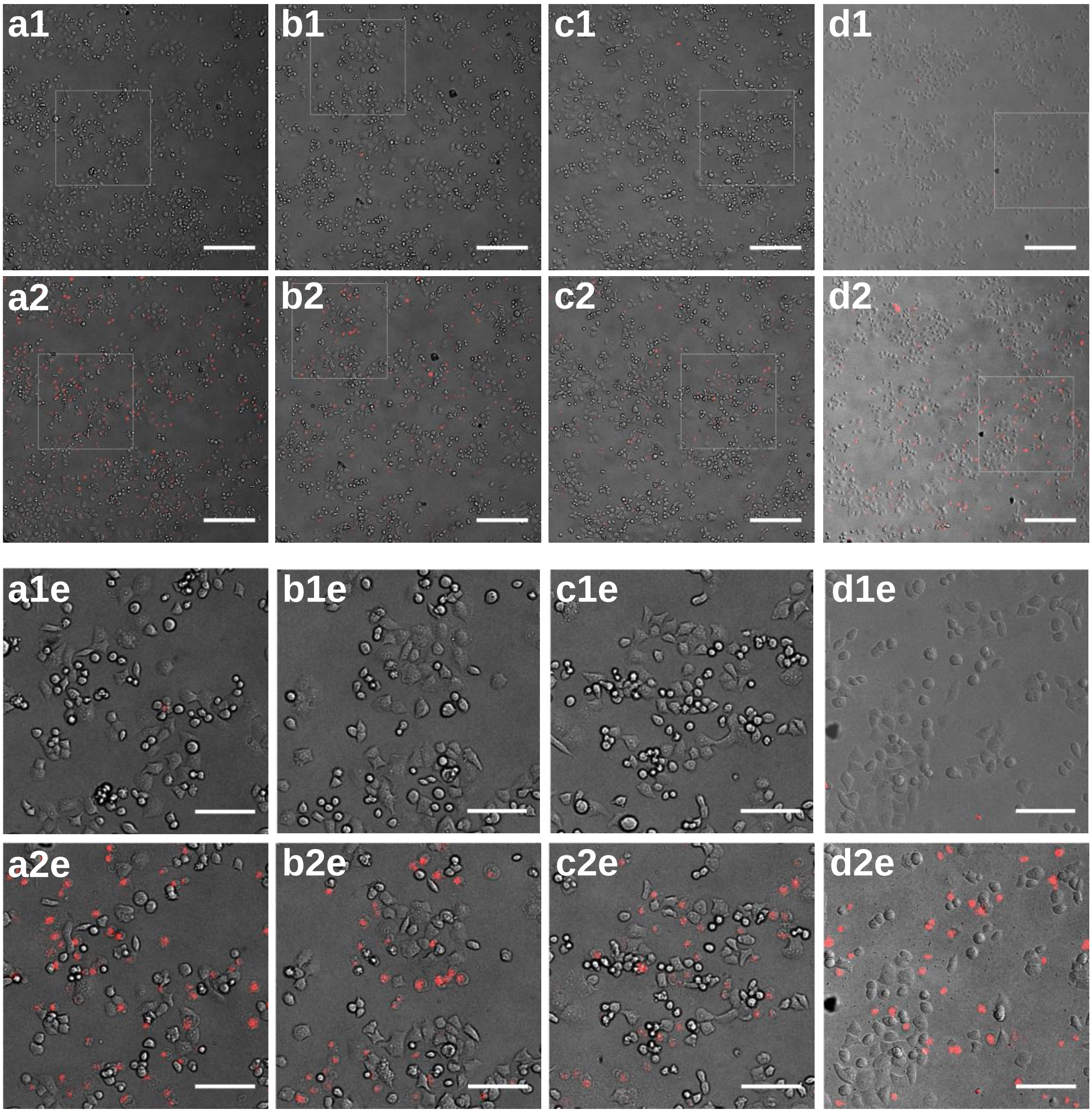

Fig. 85. Cell membrane permeabilisation induced by *trans*-GdAzo (1 mM) using confocal microscopy. Light microscopy imaging of human pancreatic cancer cells (PANC-1) in the presence of PI (1 µM) before (**a1-d1**, quadruplicate) and 30 min after (**a2-d2**, quadruplicate) the introduction of the active *trans*-**GdAzo** compound (final concentration 1 mM). Overlap of bright field and fluorescence (ex. 538 nm, em. 560-650 nm) images is presented. PI appears in red on these images. Wide-angle images (scale bar = 300 µm) and a higher magnification on the same images (**e**, white square, scale bar = 125 µm) are presented.

14.3 - Cell permeabilisation of *cis*-GdAzo upon ionising radiation (confocal microscopy)

Cells (PANC-1) were seeded (10,000 cells in 200 µL/well) in imaging chamber (Lab-Tek chamber slide system, glass, 8 well from Thermo Fisher Scientific, France) and maintained in culture medium in a humid atmosphere at 37 °C with 5% CO_2. 24 h post-seeding, culture medium was carefully replaced by PBS (95 µL) and PI (5 µL, final concentration 1 µM) was added in the medium. 15 min after addition, *cis*-**GdAzo** (100 µL, final concentration 0, 250, 500 or 850 µM, PBS) was carefully introduced in the medium. A first set of images was acquired at this stage and then the imaging chamber was irradiated (gamma-rays, 2 Gy). The imaging chamber was kept in the dark at 37 °C using a stage heater and images were acquired each 5 min for 30 min. A similar procedure was used for the non-irradiated control experiment except that the imaging chamber was not irradiated upon gamma-rays. *cis*-**GdAzo** was obtained by UV irradiation (365 nm, 0.817 mW.cm^{-2}, 30 min) of *trans*-**GdAzo** (105 µL, 0.5, 1 or 1.7 mM, PBS, *cis*-**GdAzo** 88 ± 2%) in 96-well microplate. Cells were observed with an inverted Nikon microscope (Nikon Instruments Inc., Tokyo, Japan) using a 10x dry objective lens, N.A. 0.4 and equipped with a CSU-X1 Yokogawa head. The instrument was equipped with a 561 nm laser diode for excitation wavelength. The red fluorescence emission of PI was collected with a 598-672 nm wide emission slits and transmission images were acquired with the white diode. The pinhole size was set at 50 µm and a magnification lense at 1.2. Images were acquired on an e-Volve s-CMOS camera (Photometrics). Four images per well were acquired and two wells were used for each concentration of *cis*-**GdAzo**. The experiment was repeated 3 times independently and each well was considered as an independent replicate. 16-bit numerical-images were acquired with the MetaMorph software (Version 7.10.1.161, Leica, Germany). Image analysis was performed with ImageJ software[38] (Version 1.50i completed with Adjustable Watershed plugin). The number of cells on images acquired before irradiation was manually counted and the number of fluorescent cells on images acquired before and after irradiation was automatically determined using an in-house ImageJ script (Figs. 86, 87). The cell permeabilisation was determined by the difference of the proportion of fluorescent cells before and 30 min after gamma-ray irradiation. The ImageJ script was used to quantify cell-permeabilisation efficacy by generating overlap images from the transmission and fluorescence signals and counting the number of permeabilised cells (positive fluorescent signal) from the crude data. The following parameters were selected: enhance contrast (saturation = 0.35), autothreshold (triangle dark), option black background

(false), adjustable watershed (tolerance = 2), analysed particles (size = 30-Infinity). 1 data point (over 96) had to be excluded from dataset because it could not be processed by the ImageJ script.

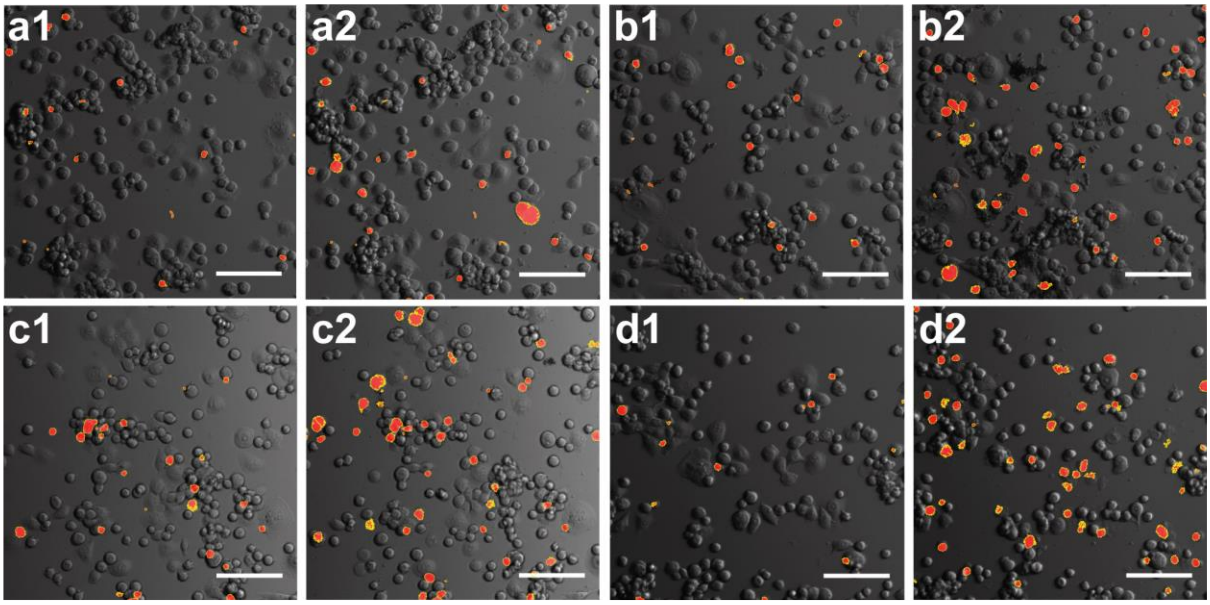

Fig. 86. Cell membrane permeabilisation induced by *cis*-GdAzo upon gamma-ray irradiation using confocal microscopy. Light microscopy imaging of human pancreatic cancer cells (PANC-1) in the presence of PI (1 µM) after introduction of the *cis*-**GdAzo** compound at the final concentration of 0 µM (**a**, introduction of PBS), 250 µM (**b**), 500 µM (**c**) and 850 µM (**d**) before (**1**) and 30 min after (**2**) after gamma-ray irradiation (2 Gy). These are examples of images generated by the ImageJ script (6 biologically independent samples). Overlap of bright field and fluorescence (ex. 561 nm, em. 598-672 nm) images is presented. PI appears in red and the counted fluorescence signals using the script are delineated in yellow. Scale bar = 125 µm.

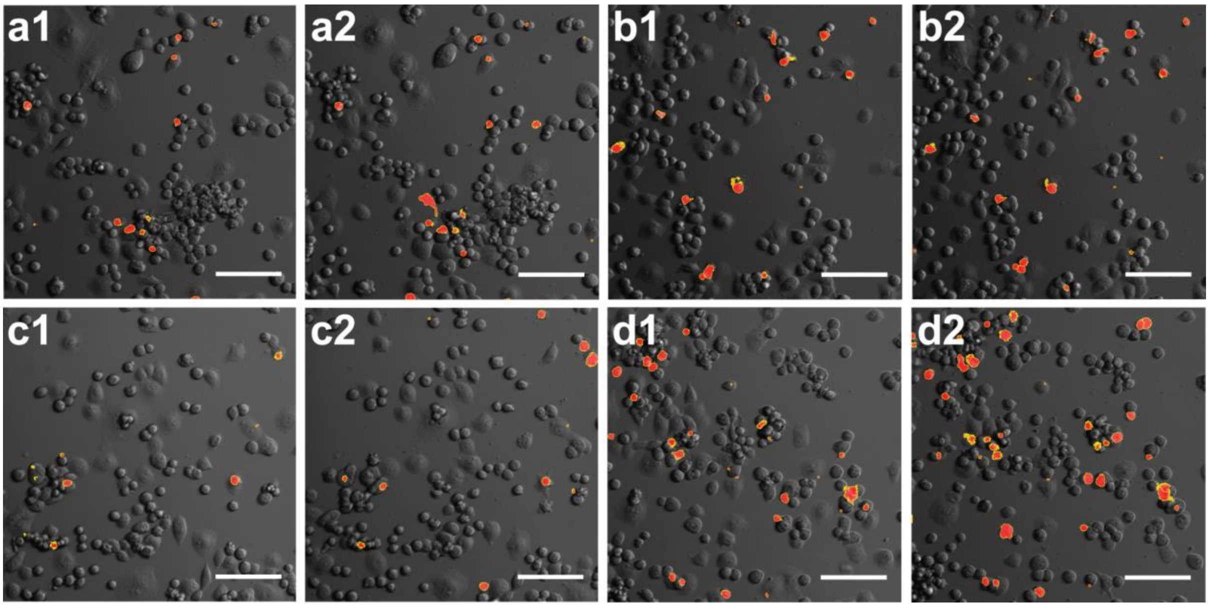

Fig. 87. Control experiment for cell membrane permeabilisation induced by *cis*-GdAzo without irradiation using confocal microscopy. Light microscopy imaging of human pancreatic cancer cells (PANC-1) in the presence of PI (1 µM) after introduction of the *cis*-**GdAzo** compound at the final concentration of 0 µM (**a**, introduction of PBS), 250 µM (**b**), 500 µM (**c**) and 850 µM (**d**) 2 min (**1**) and 30 min after (**2**) *cis*-**GdAzo** addition. In this way, the acquisitions for this control experiment were performed concurrently as those for the experiment upon gamma-ray irradiation (Fig. 86). These are examples of images generated by the ImageJ script (6 biologically independent samples). Overlap of bright field and fluorescence (ex. 561 nm, em. 598-672 nm) images is presented. PI appears in red and the counted fluorescence signals using the script are delineated in yellow. Scale bar = 125 µm.

14.4 - Cell permeabilisation of *cis*-GdAzo upon ionising radiation (flow cytometry)

Just before the experiment, cells (CCRF-CEM ARAC-8C) were dispersed in PBS and transferred (40,000 cells in 90 µL/well) in 96-well microplate (TPP from Thermo Fisher Scientific, France). *cis*-**GdAzo** (90 µL, final concentration 0, 250, 500 or 850 µM, PBS) was introduced before irradiation of the medium by GR (2 Gy). Then, PI (5 µL, final concentration 1 µM) was added and the medium was maintained in the dark at room temperature. Flow cytometry analyses were run at 15, 30 and 45 min after irradiation using a BD Accuri™ C6 Plus flow cytometer (runs of 30 µL, 100 µL/min, no threshold). *cis*-**GdAzo** was obtained by UV irradiation (365 nm, 0.817 mW.cm^{-2}, 30 min) of *trans*-**GdAzo** (105 µL, 0.5, 1 or 1.7 mM, PBS, *cis*-**GdAzo** 88 ± 2%) in 96-well microplate. A similar procedure was used for the non-irradiated control experiment except that the microplate was not irradiated upon GR. Cell membrane permeabilisation was quantified by numbering the PI-positive events (duplicate) and was represented as the relative increase in PI-positive events compared to medium without *cis*-**GdAzo** (Figs. 88, 89). The experiment was repeated 3 times independently. The data were treated using the BD Accuri C6 Plus software (version 1.0.27.1) and the representative images were obtained using the Flowjo software (version 10.7.1).

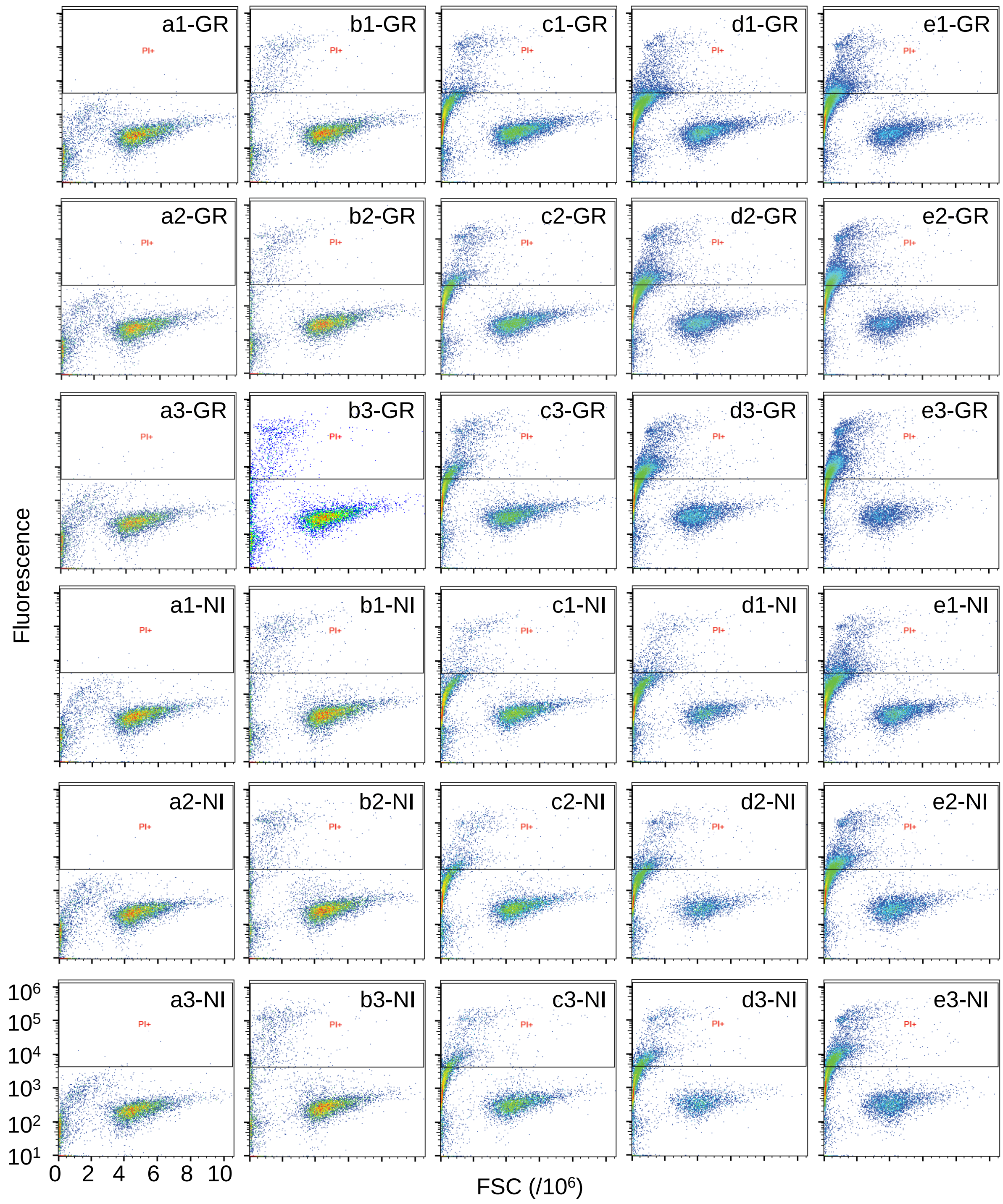

Fig. 88. Cell membrane permeabilisation induced by *cis*-GdAzo upon gamma-ray irradiation using flow cytometry. Representative flow cytometry plots of human leukemic cancer cells (CCRF-CEM ARAC-8C) in the absence (**a**) or presence (**b-e**) of PI (1 µM) after introduction of the *cis*-**GdAzo** compound at the final concentration of 0 µM (**a**, **b**, introduction of PBS), 250 µM (**c**), 500 µM (**d**) and 850 µM (**e**) at 15 min (**1**), 30 min (**2**) and 45 min (**3**) after gamma-ray irradiation (**GR**, 2 Gy) or no irradiation (**NI**). The PI+-delineation was determined by cell detection without PI (**a**). FSC: forward scatter.

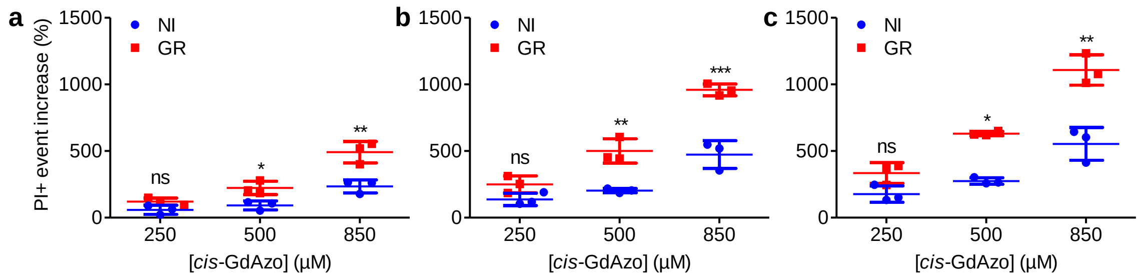

Fig. 89. Cell membrane permeabilisation induced by *cis*-GdAzo upon gamma-ray irradiation using flow cytometry. Quantification of cell permeabilisation (CCRF-CEM ARAC-8C) after treatment with *cis*-**GdAzo** upon gamma-ray irradiation (GR, 2 Gy) at 15 min (**a**), 30 min (**b**) and 45 min (**c**) after irradiation (n=3). The relative increase in PI-positive events compared to medium without *cis*-**GdAzo** (Fig. 88b) is represented. The means ± standard deviations are reported. Two-way Anova (Bonferroni post-test) was used for statistical analyses (NI *vs* GR). The *t*-values for concentrations 500 and 850 µM are 3.590 and 7.028 at 15 min (**a**), 5.254 and 8.565 at 30 min (**b**) and 4.457 and 6.935 at 45 min (**c**). ns: not significant, * $P < 0.05$, ** $P < 0.01$. *** $P < 0.001$. NI: non irradiated.

14.5 – Intracellular distribution by electron energy-loss spectroscopy transmission electron microscopy (EELS-TEM)

Cells (PANC-1) were seeded (125,000 cells in 1 mL/well) in imaging chamber (Nunc 155379 Lab-Tek II Chambered Coverglass system, 2 well from Thermo Fisher Scientific, France) and maintained in culture medium in a humid atmosphere at 37 °C with 5% CO_2. 24 h post-seeding, culture medium was removed before the addition of *trans*-**GdAzo** (490 µL, final concentration 500 µM, FBS and phenol red-free culture medium) and PI (10 µL, final concentration 1 µM) in the well. Cells were maintained in a humid atmosphere at 37 °C with 5% CO_2 for 1 h. Then, culture media was discarded and cells were washed using culture medium (2x1 mL) before the addition of fixing medium in the well (1 mL, paraformaldehyde/glutaraldehyde 1%:0.1% v/v in culture medium). Cells were maintained at 4 °C for 20 min before washing (3x1 mL, PBS) and addition of osmium (1 mL, 1% v/v in PBS). Cells were maintained at 4 °C for 40 min before washing (3x1 mL, PBS) and addition

of uranyl acetate (1 mL, 0.5% v/v in PBS). Cells were maintained at 4 °C for 30 min before washing (3x1 mL, PBS). Cells were then dried by successive washes using ethanol solutions (25%, 50%, 75%, 100% in PBS, incubation at 4 °C for 1 h for each concentration) before leaving cells in ethanol at 4 °C overnight. The cells were embedded by successive washes of Epon resin (25%, 50%, 75% and 100% in ethanol, incubation at rt for 1 h for each concentration) before leaving cells in Epon resin at 4 °C overnight. Then, the solution was replaced by Epon resin containing DMP-30 hardener (2.5% v/v) and the mixture was maintained overnight at rt before heating at 60 °C for 48 h. 100 to 200 nm-thick sections were prepared using a Leica UltraCut ultramicrotome. Sections were deposited on 200 or 300 mesh carbon-coated electron microscopy copper grids. Grids were kept in a dry and dust-free environment until observation. First, cells were observed with an inverted Leica microscope CLSM SP5 (Leica, Germany) using an HCX PL APO CS 63x/1.40 oil objective lens. The instrument was equipped with a WLL Laser (561 nm excitation wavelength). The red fluorescence emission of PI was collected with a 590-734 nm wide emission slits and transmission images were acquired with the same laser line and a PMT-trans detector. The pinhole was set at 6.28 Airy unit (600 µm). 8-bit numerical images were acquired with the Leica LAS AF software (Version 2.7.3.9723; Leica, Germany) to identify the permeabilised cells. Then, electron microscopy imaging and electron energy-loss spectroscopy (EELS) of the selected cells were performed on a 200kV JEOL 2200FS electron microscope equipped with an in-column energy filter. 32-bit numerical images were acquired with a magnification at 8,000x (pixel size 1.26 nm) and EELS spectra were obtained at 150 µm/eV by successive collection of 20 frames of 1 s exposure each. Images and spectra analyses were performed with Gatan Digital Micrograph (Version 2.31.734.0) and ImageJ software (Version 1.50i).

14.6 - Cytotoxicity of *cis*-GdAzo upon ionising radiation

Just before the experiment, cells (CCRF-CEM ARAC-8C) were dispersed in PBS and transferred (40,000 cells in 80 µL/well) in 48-well microplate (TPP from Thermo Fisher Scientific, France). Gem (20 µL, final concentration 0.1 µM) or PBS (20 µL) and *cis*-**GdAzo** (100 µL, final concentration 0, 250, 500 or 850 µM, PBS) were added before irradiation of

the medium by gamma-rays (2 Gy). The medium was maintained in the dark in a humid atmosphere at 37 °C with 5% CO_2 for 1 h. Then, culture medium (600 µL) was added in the wells and the cells were washed by 3 centrifugation cycles (300 g, 5 min, 800 µL culture medium used for each wash). Cells were dispersed in culture medium (600 µL) containing Gem (0 or 0.1 µM final concentration) and were maintained in a humid atmosphere at 37 °C with 5% CO_2 for 4 days. Living-cell number was then determined by cell counting in presence of trypan blue 1:1 v/v (triplicate). The experiment was repeated 3 times independently. Cell viability was expressed as the ratio of living-cell number after treatment to living-cell number without any treatment (non-irradiated, no Gem, no *cis*-**GdAzo**) (Figs. 90, 91). *cis*-**GdAzo** was obtained by UV irradiation (365 nm, 0.817 mW.cm^{-2}, 30 min) of *trans*-**GdAzo** (105 µL, 0.5, 1 or 1.7 mM, PBS, *cis*-**GdAzo** 88 ± 2%) in 96-well microplate. A similar procedure was used for the non-irradiated control experiment except that the 48-well microplate was not irradiated upon gamma-rays. A similar procedure was used for a control experiment upon gamma-ray irradiation by replacing the *cis*-**GdAzo** compound by Dotarem® (Fig. 92).

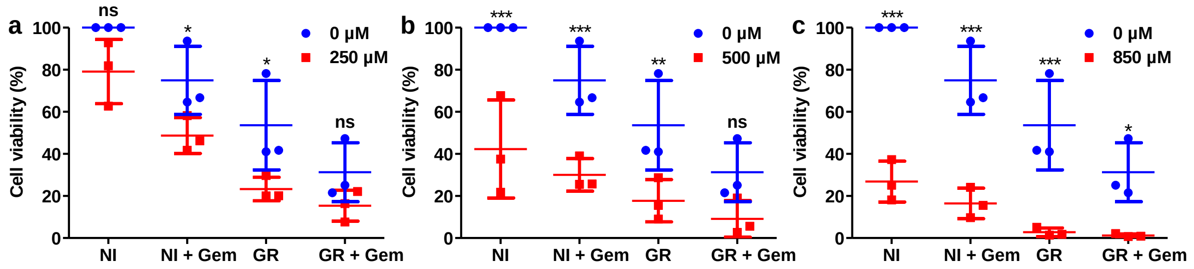

Fig. 90. Assessment of the influence of *cis*-GdAzo concentration on cell viability. Cell viability of gemcitabine (Gem)-resistant cells (human T lymphocytes, CCRF-CEM ARAC-8C) in the absence or presence of *cis*-**GdAzo** at concentrations of 250 µM (**a**), 500 µM (**b**) and 850 µM (**c**) in different conditions: no irradiation (NI), NI in the presence of Gem (0.1 µM), 2 Gy gamma-ray (GR) irradiation and 2 Gy GR irradiation in the presence of Gem (0.1 µM) (n=3). The data are represented as the means ± standard deviations. Two-way Anova (Bonferroni post-test) was used for statistical analyses (absence *vs* presence of *cis*-**GdAzo**). The *t*-values for *cis*-**GdAzo** concentration of 250 µM (**a**) are 2.797 and 3.233 for NI+Gem and GR treatments. The *t*-values for *cis*-**GdAzo** concentration of 500 µM (**b**) are 6.140, 4.781 and 3.822 for NI, NI+Gem and GR treatments. The *t*-values for *cis*-**GdAzo** concentration of 850 µM (**c**) are 7.790, 6.232, 5.416 and 3.201 for NI, NI+Gem, GR and GR+Gem treatments. ns: not significant ($P > 0.05$), *: $P < 0.05$, **: $P < 0.01$, ***: $P < 0.001$.

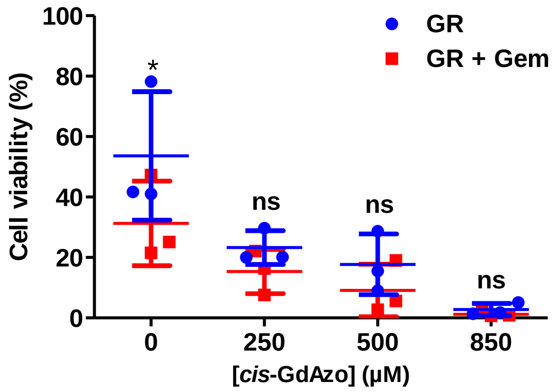

Fig. 91. Assessment of additional cytotoxic effect of Gem during treatment with *cis*-GdAzo upon ionising radiation. Cell viability of gemcitabine (Gem)-resistant cells (human T lymphocytes, CCRF-CEM ARAC-8C) in the absence or presence of Gem (0-0.1 µM) and *cis*-**GdAzo** (0, 250, 500, 850 µM) upon 2 Gy gamma-ray (GR) irradiation (n=3). The data are represented as the means ± standard deviations. Two-way Anova (Bonferroni post-test) was used for statistical analyses (GR *vs* GR + Gem, the *t*-value without *cis*-**GdAzo** is 3.301). ns: not significant ($P > 0.05$), *: $P < 0.05$. No influence of Gem on cell viability in the presence of *cis*-**GdAzo** upon 2 Gy GR irradiation could be detected in these conditions.

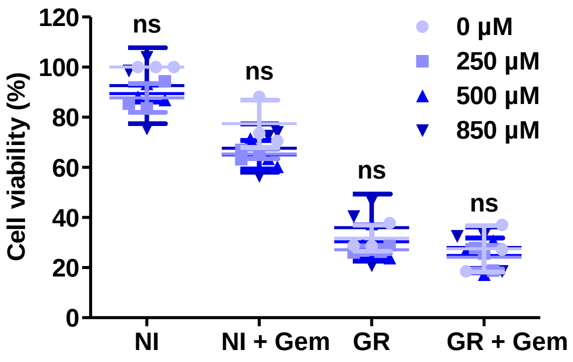

Fig. 92. Cytotoxic effect of Dotarem® upon ionising radiation. Cell viability of gemcitabine (Gem)-resistant cells (human T lymphocytes, CCRF-CEM ARAC-8C) in the absence or presence of Gem (0-0.1 µM) and Dotarem® (0, 250, 500, 850 µM) upon 2 Gy gamma-ray (GR) irradiation or without irradiation (NI) (n=3). The data are represented as the means ± standard deviations. Two-way Anova (Bonferroni post-test) was used for statistical analyses. No effect of Dotarem® on cell viability could be detected in these conditions (with or without GR irradiation, and in the absence or presence of Gem). ns: not significant ($P > 0.05$).

14.7 - Gem cellular uptake in the presence of *trans*-GdAzo

Just before the experiment, cells (CCRF-CEM or CCRF-CEM ARAC-8C) were dispersed (120,000 cells in 150 µL/well) in FBS-free culture medium and transferred in 24-well microplate (TPP from Thermo Fisher Scientific, France). NBMPR (50 µL, final concentration 0 or 100 µM, FBS-free culture medium) was introduced in the wells and cells were maintained in a humid atmosphere at 37 °C with 5% CO_2 for 15 min. A mixture of Gem and [^{3}H]Gem (50 µL, 0.1 µCi/well, final Gem concentration 10 µM, FBS-free culture medium) and *trans*-**GdAzo** (50 µL, final concentration 0 or 500 µM, FBS-free culture medium) were added in the wells and cells were maintained in a humid atmosphere at 37 °C with 5% CO_2 for 1 h. Then, culture medium (600 µL) was added in the wells and cells were washed by 2 centrifugation cycles (200 g, 5 min, 1 mL culture medium used for each wash) and were dispersed in red phenol-free culture medium (1 ml). The living-cell number was then determined by cell counting in presence of trypan blue 1:1 v/v (duplicate). The amount of internalised [^{3}H]Gem was determined using a β-scintillation counter (Beckman Coulter LS6500). Briefly, cellular suspensions were first solubilised with a mixture of Soluene-350® (Perkin Elmer) and isopropanol 1:1 v/v at 50-60 °C overnight before the addition of Hionic-Fluor scintillation cocktail® (Perkin Elmer). Then, samples were vigorously vortexed for 1 min and kept aside for 2 h before counting. The experiment was repeated 3 times independently.

This experiment confirmed that *trans*-**GdAzo** does not increase Gem penetration inside the resistant cancer cells (Fig. 93).

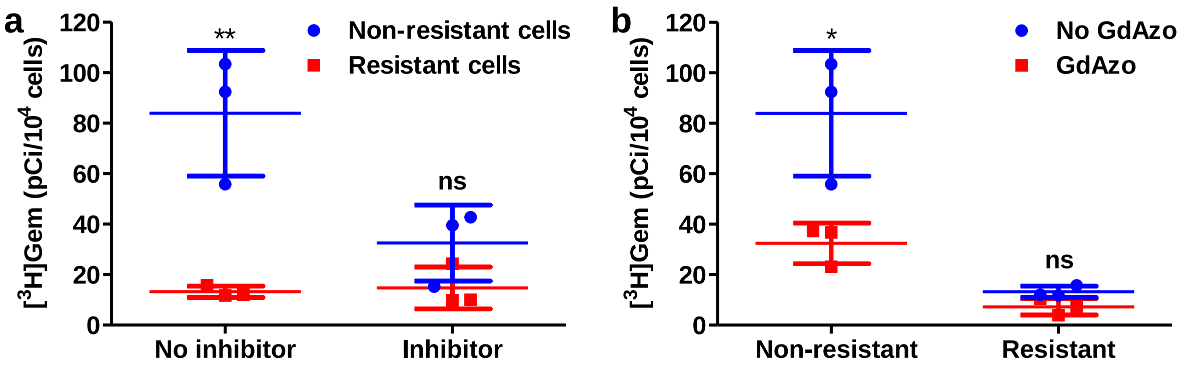

Fig. 93. Gemcitabine uptake by cancer cells. **a**, Radiolabelled-gemcitabine ([^{3}H]Gem) uptake in non-resistant control cancer cells (human T lymphocytes, CCRF-CEM) and Gem-resistant cancer cells (CCRF-CEM ARAC-8C, cell line which does not express the hENT1 receptor) in the absence or presence of an inhibitor of the hENT1 receptors (NBMPR) (n=3). **b**, [^{3}H]Gem uptake in the non-resistant and Gem-resistant cancer cells in the absence or presence of *trans*-**GdAzo** (n=3). The data are represented as the means ± standard deviations. Two-way Anova (Bonferroni post-test) was used for statistical analyses (**a**, non-resistant *vs* resistant cells, t-value = 6.314; **b**, without *vs* with *trans*-**GdAzo**, t-value = 4.095). ns: not significant ($P > 0.05$), * $P < 0.05$, ** $P < 0.01$. The reduced uptake of Gem for the resistant cells was confirmed (**a**) and no influence of *trans*-**GdAzo** on Gem-uptake by the resistant cancer cells could be detected in these conditions (**b**).

Abbreviations

CMC, critic micelle concentration; d, doublet; DFT, density functional theory; DMEM, dulbecco's modified eagle medium-high glucose; DSC, differential scanning calorimetry; E, electron beam; EELS-TEM, electron energy loss spectroscopy coupled to transmission electronic microscopy; FBS, fetal bovine serum; GR, gamma-ray; HPLC, high-performance liquid chromatography; HRMS, high-resolution mass spectrometry; IR, ionising radiation; LC-MS, mass spectrometry coupled to high-performance liquid chromatography; m, multiplet; MRI, magnetic resonance imaging; NI, non-irradiated; NMR, nuclear magnetic resonance; OD, optical density; PBS, phosphate buffered saline; PSS, photostationary state; ROS, reactive oxygen species; RPMI, roswell park memorial institute medium; rt, room temperature; SAXS, small angle X-rays scattering; SHE, standard hydrogen electrode; t, triplet; T_1, spin–lattice relaxation time; $t_{1/2}$, thermal half-life; TLC, thin layer chromatography; UV, ultraviolet; XR, X-ray.

Chemical entities:

Azo, 4-(*N*-(1,4,7,10-tetraazacyclododecane-1,4,7-triacetic acid-10-glutaryl)-ethoxyamide)-4'-butoxyazobenzene; $CDCl_3$, deuterated chloroform; $Cd(ClO_4)_2$, cadmium perchlorate; DCM, dichloromethane; DMF, *N*,*N*-dimethylformamide; DMSO, dimethylsulfoxyde; DOTA, 1,4,7,10-tetraazacyclododecane-1,4,7,10-tetraacetic acid; DOTAGA anhydride, 2,2′,2”-(10-(2,6-dioxotetrahydro-2H-pyran-3-yl)-1,4,7,10-tetraazacyclododecane-1,4,7-triyl)triacetic acid; EtOH, ethanol; Gd, gadolinium; GdAzo, gadolinium 4-(*N*-(1,4,7,10-tetraazacyclododecane-1,4,7-triacetic acid-10-glutaryl)-ethoxyamide)-4'-butoxyazobenzene; $GdCl_3.6H_2O$, gadolinium chloride hexahydrate; Gem, gemcitabine; $H^•$, hydrogen radicals; H_2O_2, hydrogen peroxide; HCl, hydrochloric acid; $HO^•$, hydroxyl radical; MeOD, deuterated methanol; MeOH, methanol; $MgSO_4$, magnesium sulfate; MTT, thiazolyl blue tetrazolium bromide; NaN_3, sodium azide; $NaNO_2$, sodium nitrite; NaOH, sodium hydroxide; Na_2SeO_4, sodium selenate; NBMPR, *S*-(4-nitrobenzyl)-6-thioinosine; PI, propidium iodide; tBuOH, *tert*-butanol; TFA, trifluoroacetic acid.

References

1. Francesc, S. in *PENELOPE-2014 - A Code System for Monte Carlo Simulation of Electron and Photon Transport.* NEA/NSC/DOC(2015)3 (Workshop Barcelona, Spain, 2015).

2. Loh, Z.-H. *et al.* Observation of the fastest chemical processes in the radiolysis of water. *Science* **367,** 179-182 (2020).

3. Bobrowski, K. *Applications of Ionizing Radiation in Materials Processing* Vol. 1 (eds Yongxia Sun & Andrzej Grzegorz Chmielewski) Ch. 4, p. 81-116 (Institute of Nuclear Chemistry and Technology, Warszawa, 2017).

4. Hiroki, A., Pimblott, S. M. & LaVerne, J. A. Hydrogen peroxide production in the radiolysis of water with high radical scavenger concentrations. *J. Phys. Chem. A* **106,** 9352-9358 (2002).

5. Pastina, B. & LaVerne, J. A. Scavenging of the precursor to the hydrated electron by the selenate ion. *J. Phys. Chem. A* **103,** 209-212 (1999).

6. Getoff, N. Pulse radiolysis studies of β-carotene in oxygenated DMSO solution. Formation of β-carotene radical cation. *Radiat. Res.* **154,** 692-696 (2000).

7. Singh, A., Koroll, G. W. & Cundall, R. B. Pulse radiolysis of aqueous solutions of sodium azide: Reactions of azide radical with tryptophan and tyrosine. *Radiat. Phys. Chem.* **19,** 137-146 (1982).

8. Et Taouil, A. *et al.* How protein structure affects redox reactivity: example of Human centrin 2. *Phys. Chem. Chem. Phys.* **16,** 24493-24498 (2014).

9. Singh, T. S., Madhava Rao, B. S., Mohan, H. & Mittal, J. P. A pulse radiolysis study of coumarin and its derivatives. *J. Photochem. Photobiol.* **153,** 163-171 (2002).

10. Louit, G. *et al.* The reaction of coumarin with the OH radical revisited: hydroxylation product analysis determined by fluorescence and chromatography. *Radiat. Phys. Chem.* **72,** 119-124 (2005).

11. Sicard-Roselli, C. *et al.* A New Mechanism for Hydroxyl Radical Production in Irradiated Nanoparticle Solutions. *Small* **10,** 3338-3346 (2014).

12. Louit, G. *et al.* Determination of hydroxyl rate constants by a high-throughput fluorimetric assay: towards a unified reactivity scale for antioxidants. *Analyst* **134,** 250-255 (2009).

13. Náfrádi, M. *et al.* Application of coumarin and coumarin-3-carboxylic acid for the determination of hydroxyl radicals during different advanced oxidation processes. *Radiat. Phys. Chem.* **170,** 108610 (2020).

14. Brun, E., Girard, H. A., Arnault, J.-C., Mermoux, M. & Sicard-Roselli, C. Hydrogen plasma treated nanodiamonds lead to an overproduction of hydroxyl radicals and solvated electrons in solution under ionizing radiation. *Carbon* **162,** 510-518 (2020).

15. Fulford, J., Bonner, P., Goodhead, D. T., Hill, M. A. & O'Neill. Experimental determination of the dependence of OH radical yield on photon energy: A comparison with theoretical simulations. *J. Phys. Chem. A* **103,** 11345-11349 (1999).

16. Buxton, G. V., Greenstock, C. L., Phillips Helman, W. & Ross, A. B. Critical Review of rate constants for reactions of hydrated electrons, hydrogen atoms and hydroxyl radicals (·OH/·O−) in Aqueous Solution. *J. Phys. Chem. Ref. Data* **17,** 513-886 (1988).

17. Krishna, M. M. G. Excited-state kinetics of the hydrophobic probe Nile red in membranes and micelles. *J. Phys. Chem. A* **103,** 3589-3595 (1999).

18. Hohenberg, P. & Kohn, W. Inhomogeneous electron gas. *Phys. Rev.* **136,** B864-B871 (1964).

19. Kohn, W. & Sham, L. J. Self-consistent equations including exchange and correlation effects. *Phys. Rev.* **140,** A1133-A1138 (1965).

20. Lee, C., Yang, W. & Parr, R. G. Development of the Colle-Salvetti correlation-energy formula into a functional of the electron density. *Phys. Rev. B* **37,** 785-789 (1988).

21. Becke, A. D. Density-functional thermochemistry. III. The role of exact exchange. *J. Chem. Phys.* **98,** 5648-5652 (1993).

22. Dolg, M., Stoll, H. & Preuss, H. Energy-adjusted ab initio pseudopotentials for the rare earth elements. *J. Chem. Phys.* **90,** 1730-1734 (1989).

23. Hehre, W. J., Radom, L., Schleyer, P. V. R. & Pople, J. A. *Ab Initio Molecular Orbital Theory*. (John Wiley, New York, 1986).

24. [gaussian] Gaussian 16, v. revision B.01, Frisch, M. J. *et al.* (Gaussian, Inc., Wallingford CT, 2016).

25. Tomasi, J., Mennucci, B. & Cammi, R. Quantum mechanical continuum solvation models. *Chem. Rev.* **105,** 2999-3094 (2005).

26. Pettersen, E. F. *et al.* UCSF Chimera—A visualization system for exploratory research and analysis. *J. Comput. Chem.* **25,** 1605-1612 (2004). (UCSF Chimera is developed by the Resource for Biocomputing, Visualization, and Informatics at the University of California, San Francisco, with support from NIH P41-GM103311).

27. Schaftenaar, G. & de Vlieg, J. Quantum mechanical polar surface area. *J. Comput. Aided Mol. Des.* **26,** 311-318 (2012).

28. Cosentino, U. *et al.* Conformational characterization of lanthanide(III)−DOTA complexes by ab initio investigation in vacuo and in aqueous solution. *J. Am. Chem. Soc.* **124,** 4901-4909 (2002).

29. Sebai, S. C., Cribier, S., Karimi, A., Massotte, D. & Tribet, C. Permeabilization of lipid membranes and cells by a light-responsive copolymer. *Langmuir* **26,** 14135-14141 (2010).

30. Sebai, S. C. *et al.* Photocontrol of the translocation of molecules, peptides, and quantum dots through cell and lipid membranes doped with azobenzene copolymers. *Angew. Chem. Int. Ed.* **51,** 2132-2136 (2012).

31. Bressler, I., Kohlbrecher, J. & Thunemann, A. F. SASfit: a tool for small-angle scattering data analysis using a library of analytical expressions. *J. Appl. Crystallogr.* **48,** 1587-1598 (2015).

32. Svergun, D. I., Semenyuk, A. V. & Feigin, L. A. Small-angle-scattering-data treatment by the regularization method. *Acta Cryst.* **44,** 244-250 (1988).

33. Svergun, D. Determination of the regularization parameter in indirect-transform methods using perceptual criteria. *J. Appl. Cryst.* **25,** 495-503 (1992).

34. Denisov, I. G., Grinkova, Y. V., Lazarides, A. A. & Sligar, S. G. Directed self-assembly of monodisperse phospholipid bilayer nanodiscs with controlled size. *J. Am. Chem. Soc.* **126,** 3477-3487 (2004).

35. Denisov, I. G., McLean, M. A., Shaw, A. W., Grinkova, Y. V. & Sligar, S. G. Thermotropic phase transition in soluble nanoscale lipid bilayers. *J. Phys. Chem. B* **109,** 15580-15588 (2005).

36. Nagle, J. F. & Tristram-Nagle, S. Structure of lipid bilayers. *Biochim. Biophys. Acta* **1469,** 159-195 (2000).

37. Shaw, A. W., McLean, M. A. & Sligar, S. G. Phospholipid phase transitions in homogeneous nanometer scale bilayer discs. *FEBS Lett.* **556,** 260-264 (2004).

38. Schneider, C. A., Rasband, W. S. & Eliceiri, K. W. NIH Image to ImageJ: 25 years of image analysis. *Nat. Methods* **9,** 671 (2012).
