# Supplementary material for: Breaking photoswitch activation depth limit using ionising radiation stimuli adapted to clinical application
Source: Nat Commun. 2022 Jul 14;13:4102. doi: 10.1038/s41467-022-30917-0 (PMC9283480; doi:10.1038/s41467-022-30917-0)
Supplement: Supplementary file 2 — Description of Additional Supplementary Files [file 41467_2022_30917_MOESM2_ESM.docx]

**Description of Additional Supplementary Files**

**Supplementary Video 1. Cell permeabilisation upon gamma-rays (2 Gy) (control experiment).** Time-course montage of human pancreatic cancer cell (PANC-1) permeabilisation obtained by live-cell confocal imaging in the presence of propidium iodide (PI, 1 µM). Cell permeabilisation was imaged every 5 min over 30 min after irradiation by gamma-rays (2 Gy). The images are overlap of bright field and fluorescence of PI (ex. 561 nm, em. 598-672 nm). PI appears in red and reveals cell permeabilisation. Scale bar = 125 µm.

**Supplementary Video 2. Cell permeabilisation by *cis*-GdAzo without irradiation (control experiment).** Time-course montage of human pancreatic cancer cell (PANC-1) permeabilisation obtained by live-cell confocal imaging in the presence of propidium iodide (PI, 1 µM) and *cis*-**GdAzo** (850 µM). Cell permeabilisation was imaged every 5 min over 30 min after the introduction of *cis*-**GdAzo**. The images are overlap of bright field and fluorescence of PI (ex. 561 nm, em. 598-672 nm). PI appears in red and reveals cell permeabilisation. Scale bar = 125 µm.

**Supplementary Video 3. Cell permeabilisation by *cis*-GdAzo upon gamma-rays (2 Gy).** Time-course montage of human pancreatic cancer cell (PANC-1) permeabilisation obtained by live-cell confocal imaging in the presence of propidium iodide (PI, 1 µM) and *cis*-**GdAzo** (850 µM). Cell permeabilisation was imaged every 5 min over 30 min after irradiation by gamma-rays (2 Gy, carried out 2 min after the introduction of *cis*-**GdAzo**). The images are overlap of bright field and fluorescence of PI (ex. 561 nm, em. 598-672 nm). PI appears in red and reveals cell permeabilisation. The arrows show a loss of integrity for some cells leading to collapse. Scale bar = 125 µm.
