# Supplementary material for: Breaking photoswitch activation depth limit using ionising radiation stimuli adapted to clinical application
Source: Nat Commun. 2022 Jul 14;13:4102. doi: 10.1038/s41467-022-30917-0 (PMC9283480; doi:10.1038/s41467-022-30917-0)
Supplement: Supplementary file 7 — Reporting Summary [file 41467_2022_30917_MOESM7_ESM.pdf]

nature portfolio

Corresponding author(s): Guillaume BORT

Last updated by author(s): 29.03.2022

Reporting Summary

Nature Portfolio wishes to improve the reproducibility of the work that we publish. This form provides structure for consistency and transparency in reporting. For further information on Nature Portfolio policies, see our Editorial Policies and the Editorial Policy Checklist.

Statistics

For all statistical analyses, confirm that the following items are present in the figure legend, table legend, main text, or Methods section.

n/a	Confirmed	
☐	☒	The exact sample size (*n*) for each experimental group/condition, given as a discrete number and unit of measurement
☐	☒	A statement on whether measurements were taken from distinct samples or whether the same sample was measured repeatedly
☐	☒	The statistical test(s) used AND whether they are one- or two-sided<br>*Only common tests should be described solely by name; describe more complex techniques in the Methods section.*
☐	☒	A description of all covariates tested
☐	☒	A description of any assumptions or corrections, such as tests of normality and adjustment for multiple comparisons
☐	☒	A full description of the statistical parameters including central tendency (e.g. means) or other basic estimates (e.g. regression coefficient) AND variation (e.g. standard deviation) or associated estimates of uncertainty (e.g. confidence intervals)
☐	☒	For null hypothesis testing, the test statistic (e.g. *F*, *t*, *r*) with confidence intervals, effect sizes, degrees of freedom and *P* value noted<br>*Give P values as exact values whenever suitable.*
☒	☐	For Bayesian analysis, information on the choice of priors and Markov chain Monte Carlo settings
☒	☐	For hierarchical and complex designs, identification of the appropriate level for tests and full reporting of outcomes
☒	☐	Estimates of effect sizes (e.g. Cohen's *d*, Pearson's *r*), indicating how they were calculated

Our web collection on statistics for biologists contains articles on many of the points above.

Software and code

Policy information about availability of computer code

Data collection	Commercial or open source software was used to collect the data. Omega (version 5.70 R2) and MARS software (version 4.01 R2) were used to collect absorbance data from microplate (FLUOstar OMEGA plate-reader). Empower or MassLynx software was used for the HPLC and LC-MS experiments (mentioned in the Supplementary Information Section 1). The molecular models of GdAzo derivatives were obtained by calculations performed in the framework of density functional theory using Gaussian 16 revision B.01 software package. The small-angle X-ray scattering experiments were performed on the SWING beamline at the SOLEIL synchrotron (St-Aubin, France) and the data were collected with Foxtrot software. The data from differential scanning calorimetry experiments were collected with Pyris Thermal Analysis software system (version 9.1). The data from light-microscopy experiments were collected with Leica SP8 LAS X software (version 2.0.1), MetaMorph software (version 7.10.1.161) or Leica LAS AF software (version 2.7.3.9723) (mentioned in the Supplementary Information Section 14) and the data from electron microscopy imaging and electron energy-loss spectroscopy were collected with Gatan Digital Micrograph (version 2.31.734.0). The data from flow cytometry were collected using a BD Accuri™ C6 Plus flow cytometer and the BD Accuri C6 Plus software (version 1.0.27.1).

Data analysis	Commercial or open source software was used to analyse the data, as well as two in-house scripts (deposited in the public repository). Statistical analyses were performed with GraphPad Prism software (version 5.00). The molecular modelling figures of GdAzo derivatives (from density functional theory calculations) were rendered using UCSF Chimera (version 1.14). Gaussian 16 revision B.01 software package was used to calculate the dipole moments and the total electron densities and an in-house script (deposited in the public repository, to be used with UCSF Chimera version 1.14) was used to classify and display polar and non-polar surfaces. The data from the small-angle X-ray scattering experiments were processed with Foxtrot software and data analyses were performed using SASFit and GNOM software (mentioned in the Supplementary Information Section 13). ImageJ software (version 1.50i) was used to process the images from light microscopy experiments and to generate the videos. An in-house script for ImageJ software (version 1.50i completed with Adjustable Watershed plugin) was used to identity the permeabilised cells in the presence of cis-GdAzo upon ionising radiation (Supplementary Information Section 14.3, deposited in the public repository). Gatan Digital Micrograph (Version 2.31.734.0) and ImageJ software (Version 1.50i) were used to analyse images and spectra from electron microscopy imaging and electron energy-loss spectroscopy. The data from flow cytometry were treated using the BD Accuri C6 Plus software (version 1.0.27.1) and the representative images were obtained using the Flowjo software (version 10.7.1).

For manuscripts utilizing custom algorithms or software that are central to the research but not yet described in published literature, software must be made available to editors and reviewers. We strongly encourage code deposition in a community repository (e.g. GitHub). See the Nature Portfolio guidelines for submitting code & software for further information.

Data

Policy information about availability of data

All manuscripts must include a data availability statement. This statement should provide the following information, where applicable:

- Accession codes, unique identifiers, or web links for publicly available datasets
- A description of any restrictions on data availability
- For clinical datasets or third party data, please ensure that the statement adheres to our policy

All characterisation data and experimental protocols to evaluate the conclusions in the paper are available in the manuscript and/or the supplementary information. The main raw data and custom codes related to this paper are available in a public repository.

Field-specific reporting

Please select the one below that is the best fit for your research. If you are not sure, read the appropriate sections before making your selection.

☒ Life sciences ☐ Behavioural & social sciences ☐ Ecological, evolutionary & environmental sciences

For a reference copy of the document with all sections, see nature.com/documents/nr-reporting-summary-flat.pdf

Life sciences study design

All studies must disclose on these points even when the disclosure is negative.

Sample size	Statistical analysis was performed when experiment was repeated three times independently, which is a standard size for these types of experiments given prior reports with similar systems.
Data exclusions	One data point had to be excluded from the microscopy experiment assessing the efficacy of cis-GdAzo permeabilization upon ionising radiation (Supplementary Information Section 14.3). These crude data were automatically processed with an in-house script for ImageJ software (version 1.50i completed with Adjustable Watershed plugin) to determine the number of fluorescent cells (i.e. permeabilised cells) on images. For this experiment, four replicates were used for each cis-GdAzo concentration and the experiment was repeated six times independently. For the concentration of 250 µM, one data point could not be processed by the ImageJ script and had to be excluded for this reason (mentioned in Supplementary Information Section 14.3).
Replication	Key experiments were reproduced at least three times independently to confirm reproducibility and all replicates performed were successful.
Randomization	Randomization was not relevant for our study since all the experiments were carried out in vitro and no animal groups were used. Nevertheless, the sample allocations were random when appropriate, and the control experiments were run concurrently to other experiments to avoid any statistical bias.
Blinding	Some procedures were undertaken to have objective interpretations of the most important cell-biology experiments. The cell permeabilisation of cis-GdAzo upon ionising radiation (Supplementary Information Section 14.3) was assessed automatically from the crude data using an in-house script for ImageJ software (version 1.50i completed with Adjustable Watershed plugin, available in public repository) to process all the images in exactly the same way and to avoid any statistical bias. Assessment of cis-GdAzo cytotoxicity upon ionising radiation (Supplementary Information Section 14.6) was performed by two different investigators. The first one prepared the solutions of cis-GdAzo and introduced them in the microplates whereas the second one irradiated the samples, performed cell handling and counting. The last investigator did not know about the amounts of cis-GdAzo introduced in the microplate wells. Cell counting was the most relevant method to assess the effect of treatment since the determination of metabolic activity or protein expression can be altered upon ionising radiation and finally be unrelated to cell viability. We believe that these procedures were helpful to avoid any statistical bias even if proper blind experiments could not be implemented because of lack of human resources.

Reporting for specific materials, systems and methods

We require information from authors about some types of materials, experimental systems and methods used in many studies. Here, indicate whether each material, system or method listed is relevant to your study. If you are not sure if a list item applies to your research, read the appropriate section before selecting a response.

Materials & experimental systems

n/a	Involved in the study
☒	☐ Antibodies
☐	☒ Eukaryotic cell lines
☒	☐ Palaeontology and archaeology
☒	☐ Animals and other organisms
☒	☐ Human research participants
☒	☐ Clinical data
☒	☐ Dual use research of concern

Methods

n/a	Involved in the study
☒	☐ ChIP-seq
☐	☒ Flow cytometry
☒	☐ MRI-based neuroimaging

Eukaryotic cell lines

Policy information about cell lines

Cell line source(s)	The human pancreatic cancer cells (PANC-1) and human acute lymphoblastic leukemia cells (CCRF-CEM) were purchased from American Type Culture Collection (ATCC) and the gemcitabine-resistant human acute lymphoblastic leukemia cells (CCRF-CEM ARAC-8C, non-expression of hENT-1 receptor) were provided by Dr. Buddy Ullmann (Oregon Health Sciences University).
Authentication	No authentication of the cell lines provided by ATCC was performed. The gemcitabine uptake by CCRF-CEM ARAC-8C cells was assessed using radiolabelled gemcitabine (Supplementary Fig. 93). The lower gemcitabine uptake was confirmed for the resistant cells (vs the CCRF-CEM control cells) and was related to the hENT-1 receptor by using a specific inhibitor (S-(4-Nitrobenzyl)-6-thioinosine, NBMPR).
Mycoplasma contamination	All cell lines used in this study were tested negative for mycoplasma contamination.
Commonly misidentified lines (See ICLAC register)	All cell lines used in this study were identified.

Flow Cytometry

Plots

Confirm that:

☒ The axis labels state the marker and fluorochrome used (e.g. CD4-FITC).

☒ The axis scales are clearly visible. Include numbers along axes only for bottom left plot of group (a 'group' is an analysis of identical markers).

☒ All plots are contour plots with outliers or pseudocolor plots.

☒ A numerical value for number of cells or percentage (with statistics) is provided.

Methodology

Sample preparation	The accurate methodology to prepare the sample is described in the Supplementary Information section 14.4. The cells (CCRF-CEM ARAC-8C) were provided by Dr. Buddy Ullmann (Oregon Health Sciences University). After treatment with cis-GdAzo upon ionising radiation, propidium iodide (final concentration 1 µM) was introduced in the medium at least 10 min before running flow cytometry analyses using a BD Accuri™ C6 Plus flow cytometer (runs of 30 µL, 100 µL/min, no threshold).
Instrument	The data from flow cytometry were collected using a BD Accuri™ C6 Plus flow cytometer.
Software	The data from flow cytometry were collected and treated using the BD Accuri C6 Plus software (version 1.0.27.1) and the representative images were obtained using the Flowjo software (version 10.7.1).
Cell population abundance	The data were acquired from a starting population of 40,000 cells in PBS. The events were counted in 30 µL at 100 µL/min flow rate for each sample. The lowest numbers of counted events were 735 and 1013 in the control (no cis-GdAzo) and treated (with cis-GdAzo) samples respectively.
Gating strategy	No gating was applied to consider the cells as well as the cell debris in order to run a relevant experiment comparable to the confocal microscopy carried out in similar conditions (Supplementary Information section 14.3). The non-irradiated control experiments were performed at the same cell and cis-GdAzo concentrations. The boundaries between "positive" and "negative" cell populations were defined using the non-irradiated samples containing cells and propidium iodide without cis-GdAzo.

☒ Tick this box to confirm that a figure exemplifying the gating strategy is provided in the Supplementary Information.
